# Redox-neutral decarboxylative coupling of fluoroalkyl carboxylic acids *via* dual metal photoelectrocatalysis†

**DOI:** 10.1039/d4sc06057a

**Published:** 2024-10-12

**Authors:** Yaxing Wu, Xiuling Wang, Zhenyu Wang, Chao Chen

**Affiliations:** a Key Laboratory of Bioorganic Phosphorus Chemistry & Chemical Biology (Ministry of Education), Department of Chemistry, Tsinghua University China chenchao01@mails.tsinghua.edu.cn; b Key Laboratory of Systems Bioengineering, Ministry of Education, Department of Pharmaceutical Engineering, School of Chemical Engineering and Technology, Tianjin University China

## Abstract

Given the importance and beneficial characteristics of aliphatic CF_3_ chiral compounds in modern chemistry, efficient strategies for their synthesis are highly sought after. While α-CF_3_ carboxylic acid is an emerging and easily accessible CF_3_-containing synthon, its use as a source of fluoroalkyl is highly challenging due to its high oxidation potential. Herein, we disclose a photoelectrocatalytic method for the direct and enantioselective decarboxylative cross-coupling of α-CF_3_ carboxylic acids. Key to our approach is the strategic integration of the LMCT-induced decarboxylative process with classical nickel catalysis. This strategy enables the efficient synthesis of aliphatic chiral CF_3_ compounds with a broad range of substrates.

## Abstract 2

A photoelectrocatalytic method for the decarboxylative cross-coupling of α-CF_3_ carboxylic acids has been established. Key to our approach is the strategic integration of the LMCT-induced decarboxylative process with classical nickel catalysis.

## Graphical abstract

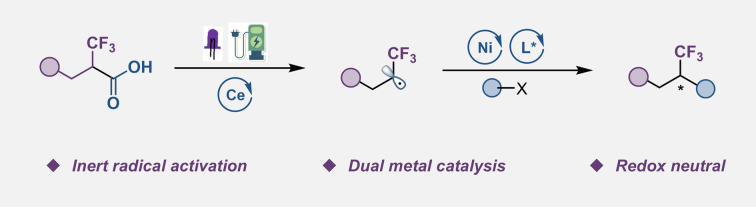

## Introduction

**Fig. 1:**
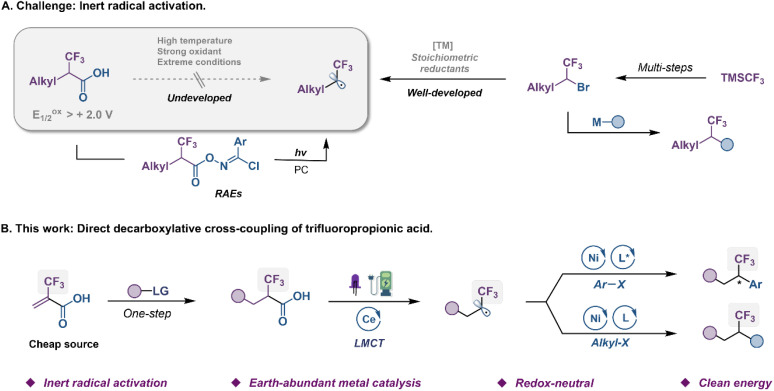
Origin of the reaction design. (A) Challenge: inert radical activation. (B) This work: direct decarboxylative cross-coupling of trifluoropropionic acid.

The trifluoromethyl group (CF_3_) represents one of the prominent functional groups in developing pharmaceuticals, agrochemicals, and advanced functional materials.^1–6^ The incorporation of CF_3_ into given lead compounds, known as the fluorine effect to modulate their biological and physiological activities, provides highly attractive opportunities in drug design.^7,8^ Numerous efforts from research groups around the world have been devoted to this field, and a plethora of sophisticated trifluoromethylating methods have been reported in the past two decades.^9–11^ While significant advancements have been made in the development of general, catalytic methods that access aryl-CF_3_ compounds, protocols for the formation of alkyl-CF_3_ bonds remain limited.^12,13^ To address these issues, an alternative strategy of trifluoromethylation by selective coupling of α-CF_3_ alkyl electrophiles with the corresponding coupling partners has recently been developed as a complementary approach (Fig. 1A, right).^14–25^ For instance, Ni-catalyzed enantioselective cross-couplings between α-CF_3_ alkyl bromides and aryl zinc or titanium species, have been established by Fu and Gandelman groups.^14,20^ However, such indirect fluoroalkylation was still hampered by the relatively poor robustness and narrow substrate scope due to the use of nucleophilic metal species, which thus limited their use for the late-stage fluoroalkylation of complex bioactive compounds. Methods based on carbon–halogen bond cleavage *via* single-electron transfer (SET) have proven to be reliable in accessing carbon radicals from organic halides,^26^ generating an open-shell intermediate for use of the complex C–C coupling reaction. Nevertheless, the activation of α-CF_3_ alkyl bromides to produce trifluoroethyl radicals needed transition-metal and strong reductants that often caused β-fluoride elimination of the M–F moiety.^21^ Recently, Xu groups established a photoredox-catalyzed enantioselective reductive cross-coupling of aryl halides with α-CF_3_ alkyl bromides.^27^

α-CF_3_ carboxylic acids, which are readily accessed from hydroalkylation or carbometallation of commercially available 2-(trifluoromethyl)acrylic acid (TFMAA), appear to be attractive radical sources from both economic and sustainability perspectives, as they are inexpensive, and produce only CO_2_ as a byproduct in the reaction (Fig. 1A, left).^28–30^ However, the high oxidation potential (*E*^ox^_p/2_ > 2.0 V *vs.* SCE) of fluoroalkyl carboxylic acids has presented significant challenges in their use as a source of the trifluoroethyl radical. These moieties have been proven difficult to decarboxylate under strong oxidants, photoredox or heat due to their unique electronic properties. In 2018, the Wang group revealed an elegant photo-catalyzed decarboxylative reaction of α-CF_3_ carboxylic acids;^31^ however, this transformation necessitated the utilization of complex *N*-hydroxybenzimidoyl chloride ester (redox-active ester, RAE) precursors. Evidently, the direct decarboxylation of unactivated carboxylic acids as a convenient source of trifluoroethyl radicals, avoiding the use of prefunctionalized substrates, remains synthetically desirable and challenging.

Ligand-to-metal charge transfer (LMCT) is an inner-sphere single electron transfer process that has different mechanisms from the photo-driven redox process that requires matching redox potential.^32–36^ In LMCT excited states, metal-based acceptor orbitals are populated and leave behind oxidized ligand-based orbitals, imparting unique properties to the excited state. This provides an effective protocol for direct decarboxylation of fluoroalkyl carboxylic acids with high oxidation potential.^37–40^ Generally, visible-light absorption triggers a M^(*n*)+^–O homolytic cleavage leading to M^(*n*−1)+^ species and a carboxylate radical that decarboxylates to a fluoroalkyl radical. In this communication, we envision a new strategy of dual metal electrophotochemical decarboxylative functionalization of α-CF_3_ carboxylic acids in a mild and green fashion (Fig. 1B). Specifically, the Ce photocatalyst that functions at the anode *via* an inner-sphere LMCT pathway engages in the net photooxidation of challenging substrates under visible-light irradiation. Concurrently, cathodic reduction of Ni catalyst is designed to activate aryl or alkyl halides producing aryl or alkyl Ni(ii) species for radical-based cross-coupling.

## Results and discussion

**Fig. 2:**
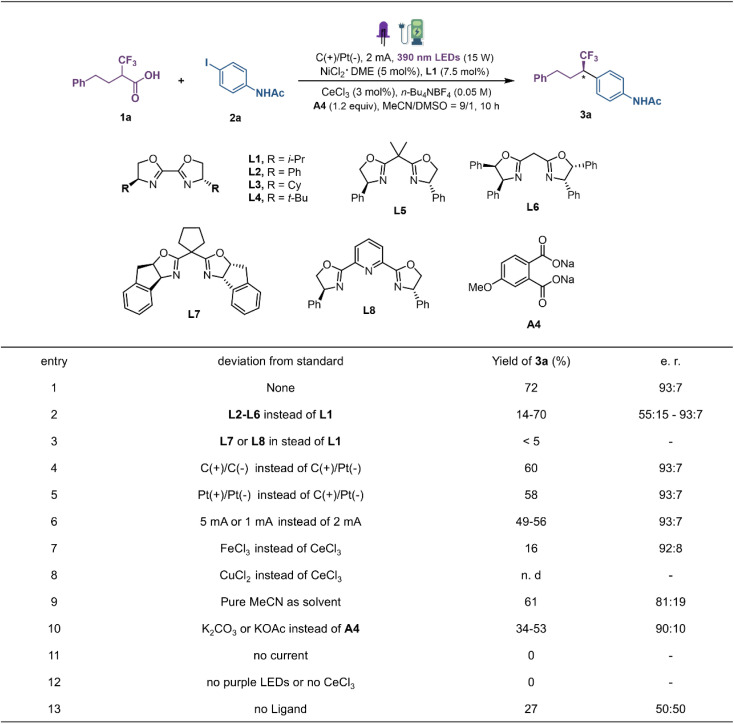
Reaction optimization. A photo-electricity reactor equipped with a LED module (15 W power, purple LED 390 nm and constant current was used). Yields were determined by GC-MS analysis *vs. n*-dodecane as an internal standard. The enantiomeric ratio was determined by chiral HPLC. See the ESI† for detailed conditions.

Our investigation was initiated with 4-phenyl-2-(trifluoromethyl)-butanoic acid (1a) and *N*-(4-iodophenyl)acetamide (2a) as the model substrate to explore the reaction conditions for decarboxylative coupling (Fig. 2). Optimal results were obtained in CH_3_CN/DMSO with a constant current of 2 mA in an undivided cell equipped with a graphite plate anode and a platinum plate cathode, under near-ultraviolet light irradiation (15 W, 390 nm light-emitting diodes (LEDs)) at room temperature. And the combination of CeCl_3_ (3 mol%), NiCl_2_·DME (DME, dimethoxyethane; 5 mol%), chiral ligand L1 (7.5 mol%) and additive A4 (1.2 equiv.) provided product 3a in 72% yield and a 93 : 7 enantiomeric ratio (e.r). The examination of different ligands revealed that the steric nature of the chiral oxazoline template has a noticeable effect on the reaction efficiency (L1–L8). In general, the sterically demanding substituents on the side chain are beneficial for imparting stereocontrol. By comparison, i-Pr-bis(oxazoline) (i-Pr-Biox) L1 exhibited the best compromise between reactivity and enantioselectivity (Fig. 2, entries 1–3). Changing either the anode material to platinum or the cathode material to graphite decreased the yields of 3a to 60% and 58%, respectively (entries 4 and 5). Both increasing and decreasing the constant current would lead to decreased yields under the same reaction conditions (entry 6). Different LMCT catalysts such as FeCl_3_ and CuCl_2_ delivered the product with sharply decreased yield (entries 7 and 8). Furthermore, replacing the optimal solvent with pure MeCN resulted in decreased yield and enantioselectivity (entry 9). Control experiments showed that the role of disodium phthalate cannot be explained simply as a base as the reaction with K_2_CO_3_ or KOAc afforded the product 3a in only 34–53% yield (entry 10). The examination of different additives revealed that the benzoate-bearing electron-donating groups on the aromatic ring result in higher yield, indicating that carboxylate salts may act as a ligand to the metal to regulate the catalytic process (see the ESI†). A series of control experiments omitting each individual component highlighted the essential roles played by electricity, metal, light and ligand in promoting this new decarboxylative coupling protocol (entries 11–13).

**Fig. 3:**
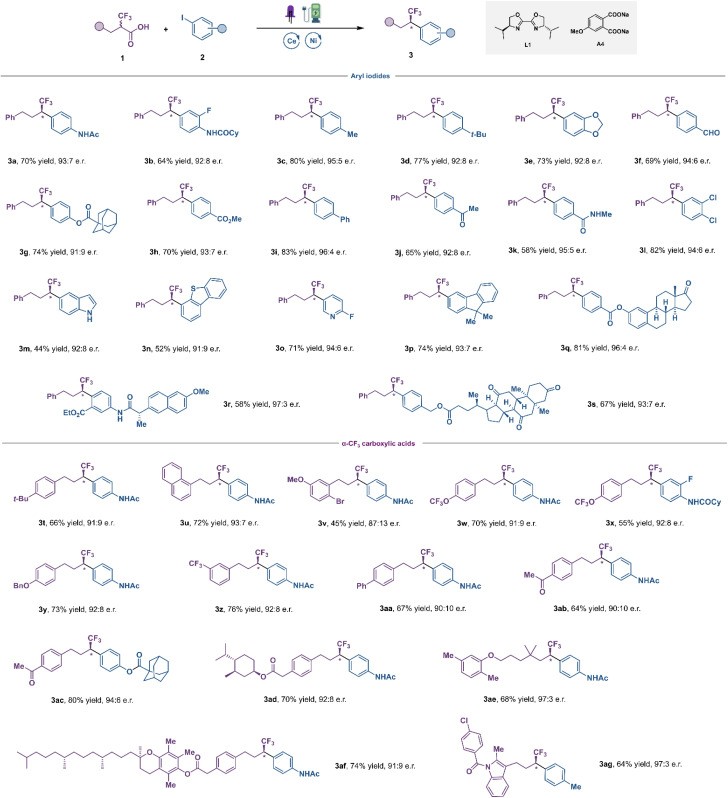
Substrate scope. The reaction conditions were conducted with α-CF_3_ carboxylic acids 1 (0.20 mmol, 1.0 equiv.), aryl iodides 2 (0.24 mmol, 1.2 equiv.), CeCl_3_ (3 mol%), NiCl_2_·diglyme (5 mol%), L1 (7.5 mol%), A4 (0.24 mmol, 1.2 equiv), *n*-Bu_4_NBF_4_ (0.10 mmol, 0.5 equiv.), C(+) and Pt(−) in degassed MeCN/DMSO (9 : 1, 2 mL) under 15 W 390 nm purple LEDs with 2 mA constant current.

With the optimized conditions in hand, the scope of this transformation was investigated next (Fig. 3). We initially focused on examining a diverse array of aryl iodides. Aryl iodides bearing acylamino (3a, 3b and 3k), alkyl (3c, 3d), alkoxy (3e), aldehyde (3f), acyloxy (3g), ester (3h), phenyl (3i), ketone (3j), chlorine (3l) and fluorine (3o) groups were all successfully converted to the corresponding fluoroalkylation products in good yields and high enantioselectivities. In general, electron-deficient aryl iodides exhibited improved efficacy compared to electron-neutral and electron-rich ones. Satisfyingly, diverse heterocycles, such as indole (3m), dibenzothiophene (3n) and pyridine (3o) were all well compatible with this catalytic system, with good yield and high e.r. This is noteworthy as this LMCT strategy allowed us to introduce fluoroalkyl groups to various electron-rich (hetero)aromatics with low oxidation potential, without the interference of undesired bimolecular redox processes. This is highly challenging under direct oxidative decarboxylation settings, in which selectivity is at the mercy of redox thermodynamics, resulting in degradation of the substrates. Moreover, aryl iodides derived from estrone (3q), naproxen (3r) and dehydrocholic acid (3s) furnished the desired coupling products in moderate to good yields with excellent diastereocontrol. The scope of α-CF_3_ carboxylic acids was explored next. As shown in Fig. 3, numerous carboxylic acids 1 were successfully arylated in the reaction, and various functional groups in the aromatic ring, including electron-donating groups (3t–3y) and electron-withdrawing groups (**3z–3ac**), were well tolerated with good yield and high e.r. To demonstrate the robustness and further synthetic utility of this new LMCT-induced decarboxylative coupling method, the late-stage functionalization of various pharmaceutical agents and biologically active compounds was carried out, such as DL–menthol (3ad), gemfibrozil (3ae), vitamin E (3af), and indometacin (3ag). To our delight, good yields and enantioselectivities were afforded in all cases, demonstrating the great potential of this mild decarboxylative coupling for efficient construction of chiral fluoroalkyl-containing derivatives of drugs or drug candidates.

**Fig. 4:**
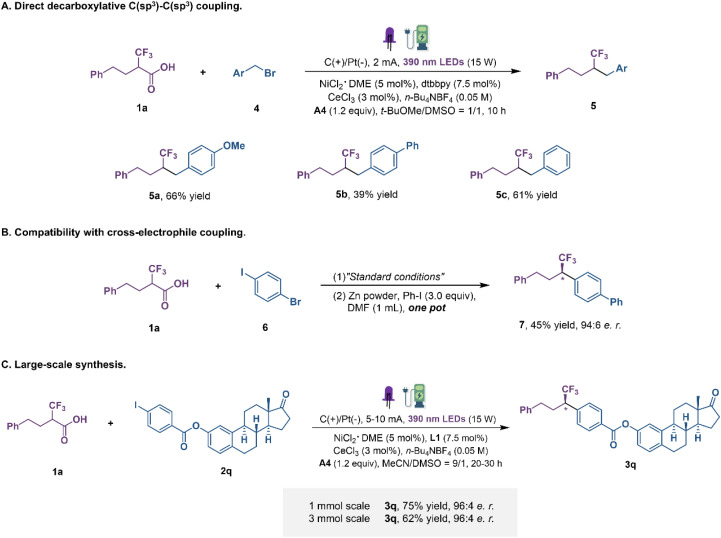
Other transformations. (A) Direct decarboxylative C(sp^3^)–C(sp^3^) coupling. (B) Compatibility with cross-electrophile coupling. (C) Large-scale synthesis.

In an attempt to extend the reaction scope to alkyl halides, we set up the reactions using alkyl bromides as the alkyl source in DMSO and *t*-BuOMe with a constant current under the irradiation of purple LEDs (Fig. 4A). Generally, C(sp^3^)–C(sp^3^) constructions are particularly difficult due to the relative weakness of the Ni–C(sp^3^) bond and the tendency of radical rebound.^41–43^ We were delighted to find that alkyl bromides participated in this transformation under modified reaction conditions. Benzyl bromides with electron-donating and electron-withdrawing groups were transformed into coupling products in moderate yields. Subsequently, the enantioenriched fluoroalkyl-containing coupling products obtained by the developed method can also proceed directly to the next step of Ni-catalytic cross-electrophile coupling without purification (Fig. 4B) and, more notably, without any detectable erosion of enantiopurity in the course, which will further enrich the chemical space of this protocol. Finally, to demonstrate the applicability of this dual transition metal catalytic decarboxylative coupling, large-scale reactions were performed (Fig. 4C). When the reaction was conducted on a 1 mmol-scale, product 3q was obtained in 75% yield, slightly reduced but maintaining the same level of enantioselectivity. Further scaling up to a 3 mmol-scale still gave a comparable outcome. These examples clearly highlight the potential synthetic value of this protocol, despite the absence of flow-chemistry equipment.

**Fig. 5:**
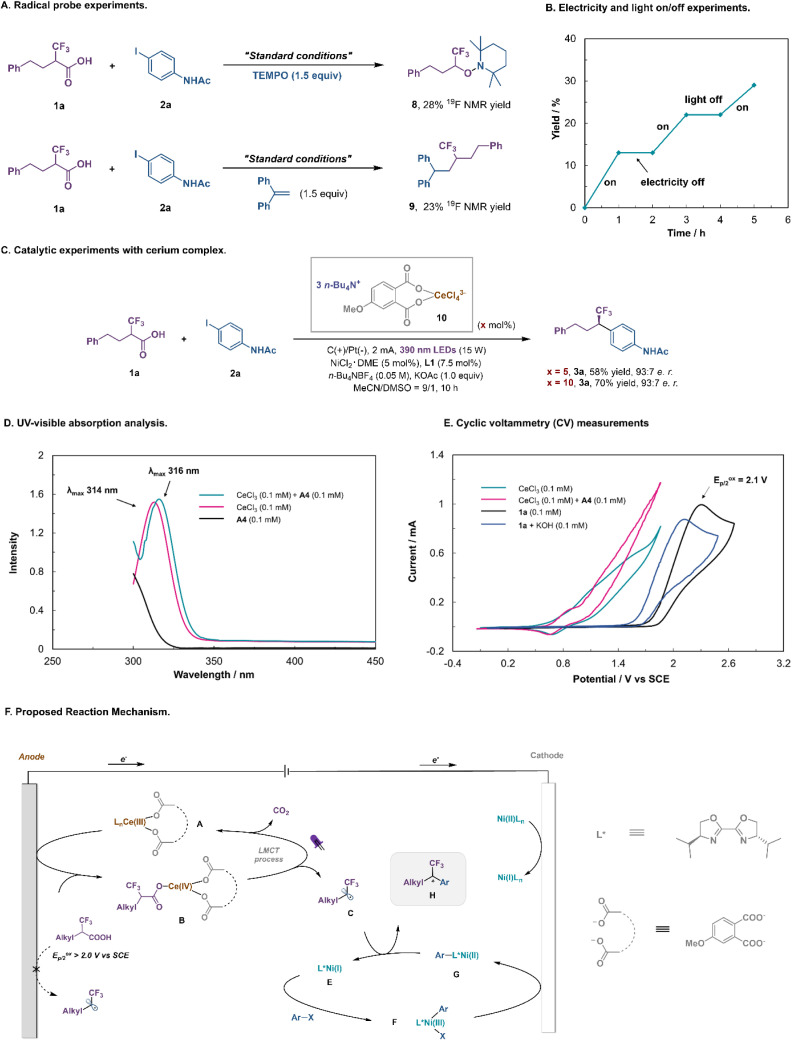
Mechanistic studies. (A) Radical probe experiments. (B) Electricity and light on/off experiments. (C) Catalytic experiments with the cerium complex. (D) UV-visible absorption analysis. (E) Cyclic voltammetry (CV) measurements. (F) Proposed reaction mechanism.

To give insights into the mechanism, a series of experiments were conducted (Fig. 5). The addition of TEMPO ((2,2,6,6-tetramethylpiperidin-1-yl)oxyl) quenched the decarboxylative coupling and TEMPO-fluoroalkyl adduct 8 was detected by ^19^F NMR spectroscopy (Fig. 5A), while the reaction with 1,1-diphenylethylene gave rise to the fluoroalkyl-incorporated product 9. The light or electricity on/off experiment revealed that the reaction was initiated by photoelectrochemical catalysis (Fig. 5B). Subsequently, the reactivity and catalytic efficiency of presynthesized (*n*-Bu_4_N)_3_(ArCOO)_2_–CeCl_4_ complex 10 were further investigated. Using 5–10 mol% of complex 10 instead of CeCl_3_/A4 as a catalyst, product 3a was obtained in 58–70% yield with an identical e.r. (93 : 7; Fig. 5C). UV-visible analysis showed that the cerium catalyst substantially absorbed near-ultraviolet light. Importantly, the addition of A4 resulted in a slight red shift in the maximum absorption wavelength of Ce(iii), indicating that new cerium species were generated under these conditions (Fig. 5D). To support our proposed LMCT activation mode, we conducted cyclic voltammetry (CV) measurements of Ce salts and α-CF_3_ carboxylic acid (Fig. 5E). While CeCl_3_ alone displayed an almost irreversible oxidative wave with an onset potential of ∼0.80 V (*vs.* SCE), its mixture with the additive A4 led to the Ce(iv)/Ce(iii) redox couple being more reversible, indicating that more stable cerium species were generated under this condition. Cyclic voltammograms indicated that the mixture of CeCl_3_ and A4 was oxidized at a much lower potential than the fluoroalkyl carboxylic acid 1a or potassium fluoroalkyl carboxylate (*E*^ox^_p/2_ = 1.8–2.1 V *vs.* SCE). Hence the integration of visible light and electricity allows the LMCT-induced decarboxylation to proceed at an anode potential much lower than that needed for the direct anodic oxidation of the fluoroalkyl carboxylic acid.

On the basis of the aforementioned mechanistic studies and precedents in the literature, a plausible mechanism for this Ce/Ni dual-catalyzed enantioselective decarboxylative cross-coupling can be proposed (Fig. 5F).^37,38,44–48^ First, the coordination of sodium phthalate to the Ce center is beneficial for both catalytic cycles. At the anode, CeCl_3_ is converted to Ce(iii) complex A, which is oxidized to Ce(iv) species B. The coordination of the α-CF_3_ carboxylic acid, followed by photoinduced ligand to metal charge transfer (LMCT), regenerates Ce(iii) salt and produces the key fluoroalkyl radical intermediate C after decarboxylation. Concurrently, reduction of the precatalyst Ni(ii)L_*n*_ at the cathode would initiate the Ni-catalytic cycle. Oxidative addition of aryl iodides to L*Ni(i) E would afford the adduct L*Ni(iii)–Ar F, which can be rapidly reduced to L*Ni(ii)–Ar G at the cathode. Subsequently, the fluoroalkyl radical C reacts with L*Ni(ii)–Ar G to produce a Ni(iii) intermediate, which undergoes reductive elimination to release the final coupled product H and L*Ni(i) E.

## Conclusions

In summary, we have developed a dual metal photoelectrocatalytic method for the direct decarboxylative coupling of unactivated α-CF_3_ carboxylic acid. Key to the success is the strategic integration of the photoelectrocatalytic LMCT process with classical asymmetric nickel catalysis. Applications of the photoelectrocatalytic LMCT protocol to decarboxylative transformations of other fluoroalkyl carboxylic acids are ongoing in our laboratory.

## Data availability

All data generated or analysed during this study are included in this article and its ESI files.†

## Author contributions

C. C. and Y. W. conceived and designed the experiments. C. C. directed the project. Y. W. and X. W. performed the experiments. Y. W. wrote the paper. All the authors provided helpful discussion on this project and contributed to manuscript writing.

## Conflicts of interest

There are no conflicts to declare.

## Supplementary Material

SC-OLF-D4SC06057A-s001
