# Supplementary material for: Redox-neutral decarboxylative coupling of fluoroalkyl carboxylic acids via dual metal photoelectrocatalysis
Source: Chem Sci. 2024 Oct 12;15(44):18497–503. doi: 10.1039/d4sc06057a (PMC11485129; doi:10.1039/d4sc06057a)
Supplement: SC-015-D4SC06057A-s001 [file SC-015-D4SC06057A-s001.pdf]

Supplementary Information (SI) for Chemical Science.
This journal is © The Royal Society of Chemistry 2024

SUPPORTING INFORMATION

Redox-neutral decarboxylative coupling of fluoroalkyl carboxylic acids via a dual metal photoelectrocatalysis

Yaxing Wu,[1] Xiuling Wang,[1,2] Zhenyu Wang,[1] and Chao Chen*[1]

1. Key Laboratory of Bioorganic Phosphorus Chemistry & Chemical Biology (Ministry of Education), Department of Chemistry, Tsinghua University, Haidian District, Beijing, 100084 (P. R. China)
2. Key Laboratory of Systems Bioengineering, Ministry of Education, Department of Pharmaceutical Engineering, School of Chemical Engineering and Technology, Tianjin University, Jingnan District, Tianjin, 300350 (P. R. China)

* E-mail: chenchao01@mails.tsinghua.edu.cn. Tel: +86-10-62773684.

TABLE OF CONTENTS

TABLE OF CONTENTS …… S2

MATERIALS AND METHODS …… S3

EXPERIMENTAL DATA …… S5

- General procedure and reaction optimization …… S5
  - General procedure for the decarboxylative fluoroalkylation …… S5
  - Optimization of reaction conditions …… S7
- Preparation of the substrates …… S14
- Substrate cope …… S23
- Direct decarboxylative C(sp^3)-C(sp^3) coupling. …… S44
- Compatibility with cross-electrophile coupling …… S47
- Large-scale synthesis …… S47
- Mechanistic investigations …… S50
  - Radical-trapping experiments …… S50
  - Electricity and light on/off experiments …… S51
  - Catalytic experiments with cerium complex …… S52
  - UV-visible absorption analysis …… S55
  - Cyclic voltammetry (CV) measurements …… S56
- Determination of abusolute configuration …… S58
- The copies of HPLC spectra …… S59
- The copies of NMR spectra …… S92

REFERENCES OF SUPPORITNG INFORMATION …… S200

MATERIALS AND METHODS

All reactions were carried out under ambient atmosphere unless otherwise stated. Concentration under reduced pressure was performed by rotary evaporation at 25-45 °C at an appropriate pressure. Purified compounds were further dried under vacuum (10^{-4}-10^{-1} KPa). Isolated yields refer to purified and spectroscopically pure compounds or mixtures of constitutional isomers. All air- and moisture-sensitive manipulations were performed using standard Schlenk- or glove-box techniques under an atmosphere of argon or dinitrogen.

Starting materials

All substrates and materials were used as received from commercial suppliers, or prepared according to published procedures, respectively, unless otherwise stated. Nickel catalysts was purchased from Energy-Chemical, and stored in an anhydrous brown bottle. Various photocatalysts was purchased from Sigma-Aldrich, Bidepharm and Energy-Chemical.

Solvents

Dry THF, DCM, DMSO and DME were purchased from Innochem (water content < 50 ppm). Dry MeCN and DMF were purchased from Energy-Chemical (water content < 30 ppm). Dry ether was obtained by post-treatment with 4 Å molecular sieve, which activated at 300 °C in muffle furnace. All deuterated solvents were purchased from Sigma-Aldrich. EtOAc and petroleum ether (boiling range 60-90 °C) was purchased from Tansoole.

Spectroscopy and instruments

NMR spectra were recorded on a JEOL AL-400MHz spectrometer operating at 400 MHz, 101 MHz, and 376 MHz, for 1H, 13C, and 19F acquisitions. Chemical shifts are reported in ppm with the solvent residual peak as the internal standard. For ^{1}H NMR: chloroform-*d*, δ 7.26; dimethyl sulfoxide-d_6, δ 2.50. For ^{13}C NMR: chloroform-*d*, δ 77.16; dimethyl sulfoxide-d_6, δ 39.52. ^{19}F NMR spectra were referenced using a unified chemical shift scale based on the ^{1}H NMR resonance of tetramethylsilane (1% (v/v) solution in the respective solvent). Data is reported as follows: s = singlet, d = doublet, t = triplet, q = quartet, m = multiplet, br = broad; coupling constants in Hz; integration. HRMS spectra were obtained using Bruker Esquire ion trap mass spectrometer in positive mode. UV-vis absorption spectra were recorded on an UV 2700.

Chromatography

Thin layer chromatography (TLC) was performed using TLC plates pre-coated with 200 μm thickness silica gel F254 plates and visualized by fluorescence quenching under 254 nm UV light,

phosphomolybdic Acid, and $KMnO_4$ stain. Column chromatography was performed on silica gel (particle size 10-40 µm, Ocean Chemical Factory of Qingdao, China). Gas chromatography-mass spectrometry (GC-MS) was performed on a Shimadzu GCMS-QP2010 SE Series chromatograph with split-mode capillary injection and FID detection. Enantiopurity was assessed *via* high performance liquid chromatography (HPLC) analysis using a Shimadzu LC-20AT Chiral HPLC. Infinity chromatograph with chiral columns as noted for each compound using a hexane/isopropanol liquid phase.

Photochemistry

All reactions with visible light were carried out using a photoreactor equipped with a LED module (15 W Power Purple LED 390 nm, purchased from GreeThink), consisting out of 50 LED-chips. The power of the LED was adjusted using a linear regulator.

EXPERIMENTAL DATA

General procedure and reaction optimization

General procedure for the decarboxylative coupling of α-CF_3 carboxylic acids

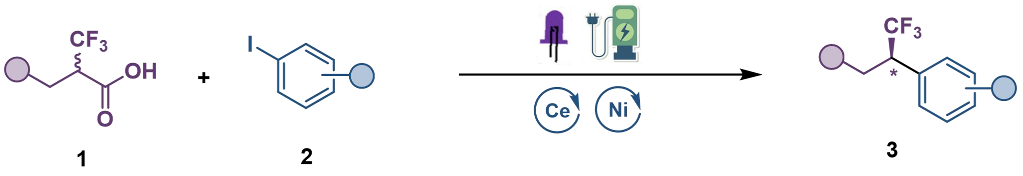

Scheme S1. General procedure for the decarboxylative coupling of α-CF_3 carboxylic acids.

An oven-dried screw-cap vial was equipped with a stir bar, a threaded Teflon cap fitted with electrical feedthroughs, a graphite plate anode (2.5 cm * 0.5 cm), and a platinum plate cathode (2.5 cm * 0.5 cm). Under nitrogen atmosphere, *n*-Bu_4NBF_4 (66 mg, 0.10 mmol, 0.5 eq.), sodium phthalate (**A4**) (58 mg, 0.24 mmol, 1.2 eq.), α-CF_3 carboxylic acid (0.20 mmol, 1.0 eq.), aryl iodide (0.24 mmol, 1.2 eq.), $CeCl_3$ (1.5 mg, 0.006 mmol, 3 mol%), $NiCl_2$ DME (2.2 mg, 0.01 mmol, 5 mol%), and chiral ligand (**L1**) (3.4 mg, 0.015 mmol, 7.5 mol%) were added to this reaction vessel. After that, the mixture of MeCN and DMSO (9:1(v:v), 2.0 mL) were added via syringe. The reaction was irradiated with purple LEDs (15 W, 390 nm) under the vessel and electrolysis was initiated at a constant current of 2.0 mA, The temperature was kept at approximately 30°C through the use of a cooling fan. After 10 hours, the photolysis and electrolysis were terminated and the reaction was quenched with 10 mL water, then extracted with DCM (3 x 10 mL). The combined organic phase was dried with anhydrous Na_2SO_4 and concentrated under vacuum. The residue was further purified by column chromatography to afford corresponding coupling products, which was characterized by GC-MS analysis, ^{1}H NMR, ^{13}C NMR and ^{19}F NMR spectroscopy. The enantioselectivity was determined via HPLC analysis with chiral columns.

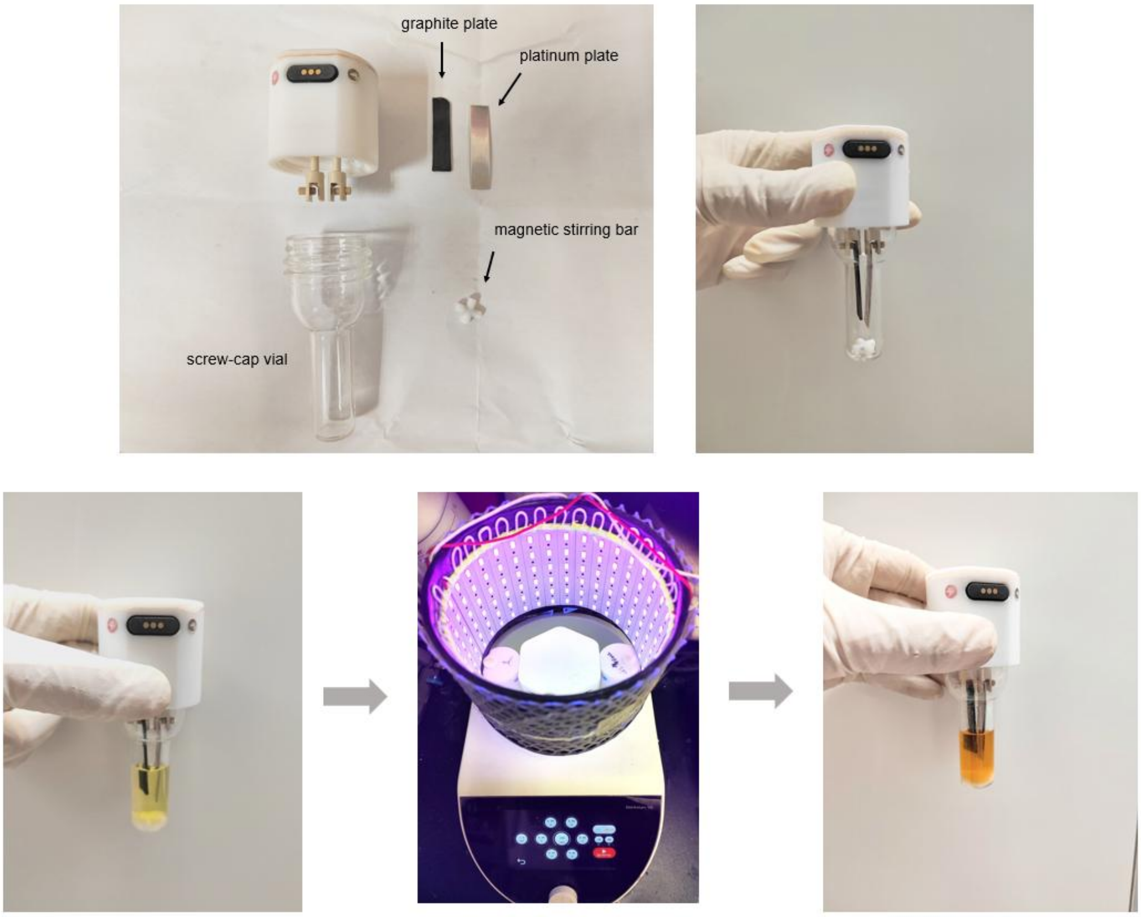

Figure S1. Components of the electrolysis setup (top left). Assembled reaction vessel (top right). Reaction before (bottom left), during (bottom middle) and after (bottom right).

Optimization of reaction conditions

Table S1. Screening of metal catalysts.

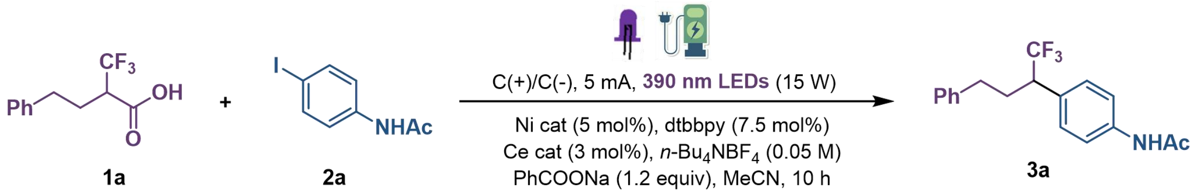

Entry	Ni cat.	Ce cat.	Yield (%)
1	$NiCl_2 \cdot DME$	$CeCl_3$	32
2	$NiCl_2 \cdot DME$	$Ce(OTf)_3$	21
3	$NiCl_2 \cdot DME$	$CeCl_3 \cdot 7H_2O$	15
4	$NiCl_2 \cdot DME$	$Ce(NO_3)_3$	30
5	$NiCl_2 \cdot DME$	$FeCl_3$ instead of [Ce]	< 5
6	$NiCl_2 \cdot DME$	$CuCl_2$ instead of [Ce]	n. d
7	$Ni(COD)_2$	$CeCl_3$	< 5
8	$Ni(COD)_2$	$Ce(NO_3)_3$	< 5
9	$Ni(COD)_2$	$FeCl_3$ instead of [Ce]	n. d
10	$Ni(acac)_2$	$CeCl_3$	20
11	$Ni(acac)_2$	$Ce(NO_3)_3$	13
12	$NiBr_2$	$CeCl_3$	29
13	$NiBr_2$	$Ce(NO_3)_3$	32

An oven-dried screw-cap vial was equipped with a stir bar, a threaded Teflon cap fitted with electrical feedthroughs, a graphite plate anode (2.5 cm * 0.5 cm), and a graphite plate cathode (2.5 cm * 0.5 cm). Under nitrogen atmosphere, *n*-Bu_4NBF_4 (66 mg, 0.10 mmol, 0.5 eq.), sodium benzoate (35 mg, 0.24 mmol, 1.2 eq.), 4-phenyl-2-(trifluoromethyl)butanoic acid (46.4 mg, 0.20 mmol, 1.0 eq.), *N*-(4-iodophenyl)acetamide (62.6 mg, 0.24 mmol, 1.2 eq.), Ce cat. (0.006 mmol, 3 mol%), Ni cat. (0.01 mmol, 5 mol%), and dtbbpy (4.1 mg, 0.015 mmol, 7.5 mol%) were added to this reaction vessel. After that, MeCN (2.0 mL) were added via syringe. The reaction was irradiated with purple LEDs (15 W, 390 nm) under the vessel and electrolysis was initiated at a constant current of 2.0 mA, The temperature was kept at approximately 30°C through the use of a cooling fan. After 10 hours, the photolysis and electrolysis were terminated and the reaction was quenched with 10 mL water, then extracted with DCM (3 x 10 mL). The combined organic phase was dried with anhydrous Na_2SO_4 and concentrated under vacuum. The crude reaction mixture was filtered through a plug of silica then subjected to GC-MS analysis.

Table S2. Screening of electrodes, current and reaction time.

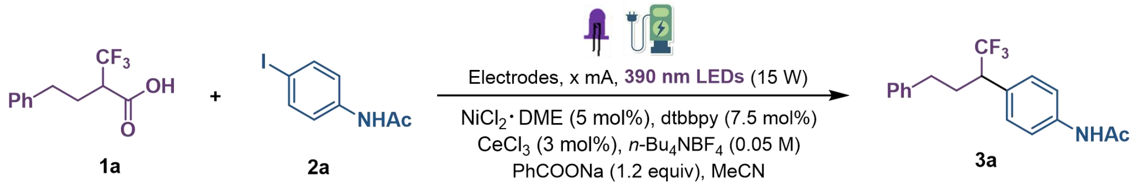

Entry	Electrodes	Current and reaction time	Yield (%)
1	C(+)/C(-)	5 mA, 10 h	32
2	C(+)/C(-)	5 mA, 2 h	11
3	C(+)/C(-)	5 mA, 12 h	30
4	C(+)/C(-)	2 mA, 24 h	28
5	C(+)/C(-)	2 mA, 10 h	35
6	C(+)/C(-)	2 mA, 3 h	8
7	Pt(+)/C(-)	5 mA, 2 h	< 5
8	Pt(+)/C(-)	2 mA, 10 h	26
9	Pt(+)/Pt(-)	5 mA, 2 h	7
10	Pt(+)/Pt(-)	2 mA, 10 h	20
11	C(+)/Pt(-)	5 mA, 2 h	13
12	C(+)/Pt(-)	2 mA, 10 h	40
13	C(+)/Pt(-)	2 mA, 5 h	22
14	C(+)/Pt(-)	2 mA, 12 h	39

An oven-dried screw-cap vial was equipped with a stir bar, a threaded Teflon cap fitted with electrical feedthroughs, an anode (2.5 cm * 0.5 cm), and a cathode (2.5 cm * 0.5 cm). Under nitrogen atmosphere, *n*-Bu_4NBF_4 (66 mg, 0.10 mmol, 0.5 eq.), sodium benzoate (35 mg, 0.24 mmol, 1.2 eq.), 4-phenyl-2-(trifluoromethyl)butanoic acid (46.4 mg, 0.20 mmol, 1.0 eq.), *N*-(4-iodophenyl)acetamide (62.6 mg, 0.24 mmol, 1.2 eq.), $CeCl_3$ (1.5 mg, 0.006 mmol, 3 mol%), $NiCl_2$ DME (2.2 mg, 0.01 mmol, 5 mol%) and dtbbpy (4.1 mg, 0.015 mmol, 7.5 mol%) were added to this reaction vessel. After that, MeCN (2.0 mL) were added via syringe. The reaction was irradiated with purple LEDs (15 W, 390 nm) under the vessel and electrolysis was initiated at a constant current, The temperature was kept at approximately 30°C through the use of a cooling fan. After the reaction, the photolysis and electrolysis were terminated and the reaction was quenched with 10 mL water, then extracted with DCM (3 x 10 mL). The combined organic phase was dried with anhydrous Na_2SO_4 and concentrated under vacuum. The crude reaction mixture was filtered through a plug of silica then subjected to GC-MS analysis.

Table S3. Screening of additives and solvent.

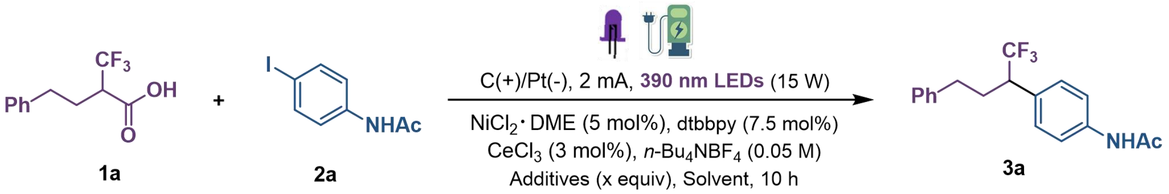

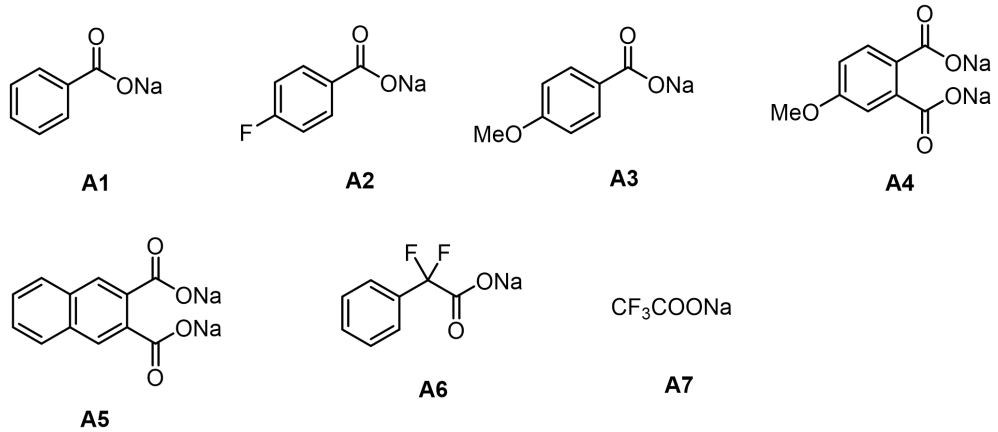

Entry	Additives	Solvent	Yield (%)
1	**A1** (1.2 equiv)	MeCN	40
2	**A2** (1.2 equiv)	MeCN	28
3	**A3** (1.2 equiv)	MeCN	46
4	**A4** (0.6 equiv)	MeCN	49
5	**A5** (0.6 equiv)	MeCN	41
6	**A4** (1.2 equiv)	MeCN	53
7	**A6** (1.2 equiv)	MeCN	10
8	**A7** (1.2 equiv)	MeCN	7
9	**A3** (1.2 equiv)	DMSO	54
10	**A4** (1.2 equiv)	DMSO	57
11	**A5** (1.2 equiv)	DMSO	40
12	**A3** (1.2 equiv)	DMA	17
13	**A4** (1.2 equiv)	DMA	15
14	**A5** (1.2 equiv)	DMA	20
15	**A4** (1.2 equiv)	MeCN/DMSO = 1/1	54
16	**A4** (1.2 equiv)	MeCN/DMSO = 9/1	60
17	**A4** (1.2 equiv)	MeCN/H_2O = 9/1	trace
18	**A3** (1.2 equiv)	MeCN/DMSO = 9/1	42

An oven-dried screw-cap vial was equipped with a stir bar, a threaded Teflon cap fitted with electrical feedthroughs, a graphite plate anode (2.5 cm * 0.5 cm), and a platinum plate cathode (2.5 cm * 0.5 cm). Under nitrogen atmosphere, *n*-Bu_4NBF_4 (66 mg, 0.10 mmol, 0.5 eq.), additives (0.24 mmol, 1.2 eq.), 4-phenyl-2-(trifluoromethyl)butanoic acid (46.4 mg, 0.20 mmol, 1.0 eq.), *N*-(4-iodophenyl)acetamide (62.6 mg, 0.24 mmol, 1.2 eq.), $CeCl_3$ (1.5 mg, 0.006 mmol, 3 mol%), $NiCl_2$ DME (2.2 mg, 0.01 mmol, 5 mol%), and dtbbpy (4.1 mg, 0.015 mmol, 7.5 mol%) were added to this reaction vessel. After that, the solvent (2.0 mL) were added via syringe. The reaction was irradiated with purple LEDs (15 W, 390 nm) under the vessel and electrolysis was initiated at a constant current of 2.0 mA, The temperature was kept at approximately 30°C through the use of a cooling fan. After 10 hours, the photolysis and electrolysis were terminated and the reaction was quenched with 10 mL water, then extracted with DCM (3 x 10 mL). The combined organic phase was dried with anhydrous Na_2SO_4 and concentrated under vacuum. The crude reaction mixture was filtered through a plug of silica then subjected to GC-MS analysis.

Table S4. Screening of chiral ligands.

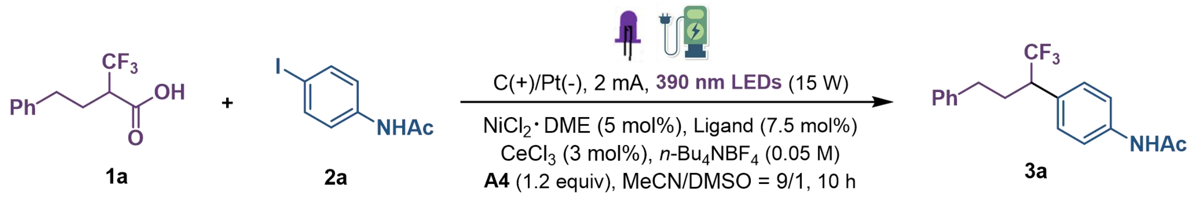

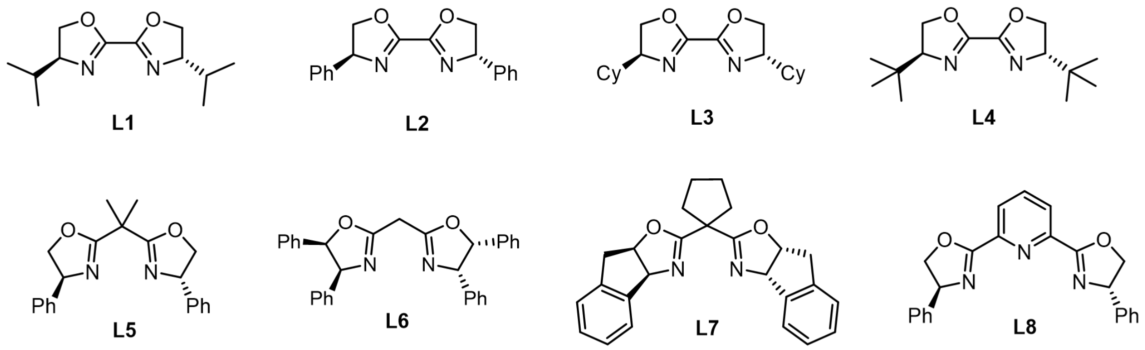

Entry	Ligands	e. r.	Yield (%)
1	**L1**	93 : 7	72
2	**L2**	55 : 15	47
3	**L3**	91 : 9	65
4	**L4**	93 : 7	70
5	**L5**	61 : 39	64
6	**L6**	76 : 24	14
7	**L7**	-	< 5
8	**L8**	-	< 5

An oven-dried screw-cap vial was equipped with a stir bar, a threaded Teflon cap fitted with electrical feedthroughs, a graphite plate anode (2.5 cm * 0.5 cm), and a platinum plate cathode (2.5 cm * 0.5 cm). Under nitrogen atmosphere, *n*-Bu_4NBF_4 (66 mg, 0.10 mmol, 0.5 eq.), sodium phthalate (**A4**) (58 mg, 0.24 mmol, 1.2 eq.), 4-phenyl-2-(trifluoromethyl)butanoic acid (46.4 mg, 0.20 mmol, 1.0 eq.), *N*-(4-iodophenyl)acetamide (62.6 mg, 0.24 mmol, 1.2 eq.), $CeCl_3$ (1.5 mg, 0.006 mmol, 3 mol%), $NiCl_2$ DME (2.2 mg, 0.01 mmol, 5 mol%), and chiral ligand (**L1-L8**) (0.015 mmol, 7.5 mol%) were added to this reaction vessel. After that, the mixture of MeCN and DMSO (9:1(v:v), 2.0 mL) were added via syringe. The reaction was irradiated with purple LEDs (15 W, 390 nm) under the vessel and electrolysis was initiated at a constant current of 2.0 mA, The temperature was kept at approximately 30°C through the use of a cooling fan. After 10 hours, the photolysis and electrolysis were terminated and the reaction was quenched with 10 mL water, then extracted with DCM (3 x 10 mL). The combined organic phase was dried with anhydrous

Na_2SO_4 and concentrated under vacuum. The crude reaction mixture was filtered through a plug of silica then subjected to GC-MS analysis. The enantioselectivity was determined via HPLC analysis with chiral columns.

Table S5. Variation from standard conditions.

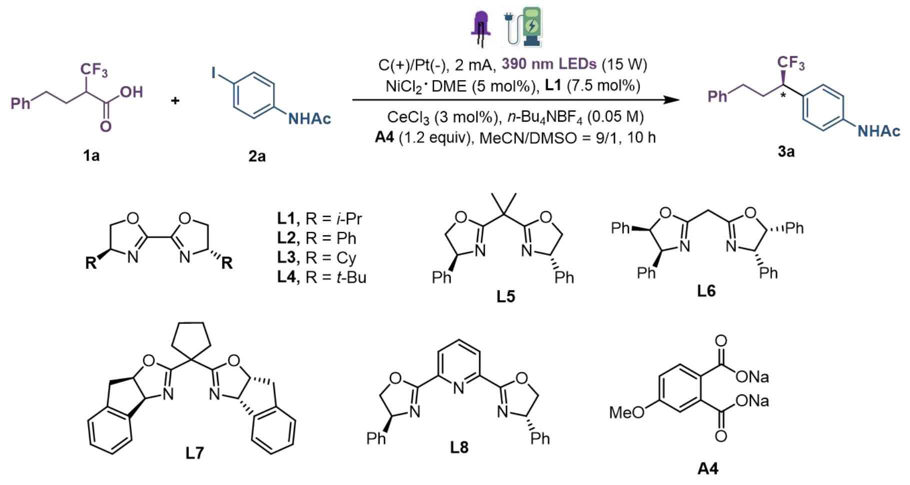

entry	deviation from standard	Yield of **3a** (%)	e. r.
1	None	72	93:7
2	**L2-L6** instead of **L1**	14-70	55:15 - 93:7
3	**L7** or **L8** in stead of **L1**	< 5	-
4	C(+)/C(-) instead of C(+)/Pt(-)	60	93:7
5	Pt(+)/Pt(-) instead of C(+)/Pt(-)	58	93:7
6	5 mA or 1 mA instead of 2 mA	49-56	93:7
7	$FeCl_3$ instead of $CeCl_3$	16	92:8
8	$CuCl_2$ instead of $CeCl_3$	n. d	-
9	Pure MeCN as solvent	61	81:19
10	K_2CO_3 or KOAc instead of **A4**	34-53	90:10
11	no **A4**	11	92:8
12	1.0 equiv KOAc and 5 mol% **A4** instead of **A4**	58	93:7
13	no current	0	-
14	no purple LEDs or no $CeCl_3$	0	-
15	no Ligand	27	50:50

Preparation of the substrates (Method 1)

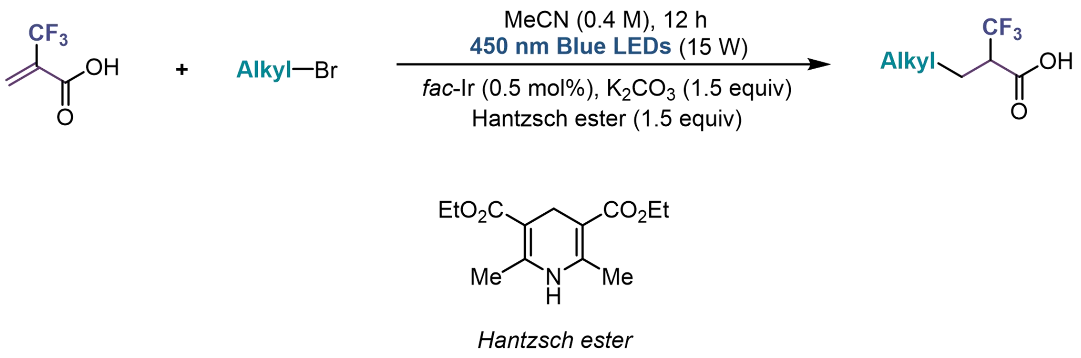

Scheme S2. Preparation of α-CF_3 carboxylic acids.

A reaction tube equipped with a magnetic stir bar was charged with *fac*-Ir(ppy)$_3$ (6.5 mg, 0.5 mol %), Hantzsch ester 1 (675 mg, 3.0 mmol, 1.5 equiv), 3,3,3-trifluoropropionic acid (280 mg, 2.0 mmol, 1.0 equiv), alkyl bromide (3.0 mmol, 1.5 equiv) K_2CO_3 (3.0 mmol, 1.5 equiv) and MeCN (5 mL) under nitrogen atmosphere. The mixture was stirred under irradiation of 15 W blue LEDs at room temperature for 12 hours. Then the mixture was diluted with H_2O and the resulting mixture was extracted with DCM for 3 times. The combined organic layers were washed with brine, dried over Na_2SO_4, filtered, and concentrated under reduced pressure. The residue was purified by chromatography on silica gel to afford corresponding products, which was characterized by GC-MS analysis, ^{1}H NMR, ^{13}C NMR and ^{19}F NMR spectroscopy.

4-phenyl-2-(trifluoromethyl)butanoic acid (**S1**)

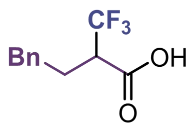

The title compound was purified with silica gel chromatography (ethyl acetate: petroleum ether, 1:30 (v:v)) as a colorless oil (90% yield).

R*f* = 0.25 (ethyl acetate: petroleum ether, 1:5 (v:v)).

NMR Spectroscopy:

^{1}H NMR (400 MHz, Chloroform-*d*, 298 K) δ 7.39 - 7.16 (m, 5H), 3.25 - 3.06 (m, 1H), 2.88 - 2.75 (m, 1H), 2.75 - 2.57 (m, 1H), 2.38 - 2.19 (m, 1H), 2.19 - 2.07 (m, 1H).

^{13}C NMR (101 MHz, Chloroform-*d*, 298 K) δ 171.55, 139.86, 128.85, 128.61, 126.70, 123.30 (q, *J* = 281.0 Hz), 49.80 (q, *J* = 27.0 Hz), 32.84, 29.86.

^{19}F NMR (376 MHz, Chloroform-*d*, 298 K) δ -68.03 (d, *J* = 8.3 Hz).

The spectra data are consistent with those reported in literature.

GC-MS: 232.

4-(4-(*tert*-butyl)phenyl)-2-(trifluoromethyl)butanoic acid (**S2**)

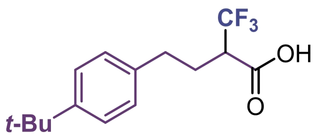

The title compound was purified with silica gel chromatography (ethyl acetate: petroleum ether, 1:30 (v:v)) as a colorless oil (84% yield).

R*f* = 0.28 (ethyl acetate: petroleum ether, 1:5 (v:v)).

NMR Spectroscopy:

^{1}**H NMR** (400 MHz, Chloroform-*d*, 298 K) δ 11.01 (br, 1H), 7.31 (d, *J* = 8.3 Hz, 2H), 7.13 (d, *J* = 8.3 Hz, 2H), 3.23 - 3.09 (m, 1H), 2.83 - 2.72 (m, 1H), 2.70 - 2.58 (m, 1H), 2.34 - 2.20 (m, 1H), 2.16 - 2.03 (m, 1H), 1.30 (s, 9H).

^{13}**C NMR** (101 MHz, Chloroform-*d*, 298 K) δ 165.48, 149.29, 137.15, 128.19, 125.55, 125.09 (q, *J* = 281.0 Hz), 49.97 (q, *J* = 27.2 Hz), 34.43, 32.33, 31.37, 27.99.

^{19}**F NMR** (376 MHz, Chloroform-*d*, 298 K) δ -67.94 (d, *J* = 8.5 Hz).

GC-MS: 288.

HRMS (ESI) m/z $[M+H]^+$: Calculated for $C_{15}H_{20}F_3O_2^+$: 289.1410. Found: 289.1412.

4-(4-(trifluoromethoxy)phenyl)-2-(trifluoromethyl)butanoic acid (**S3**)

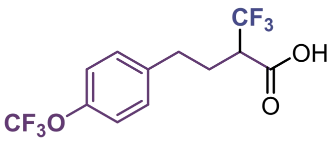

The title compound was purified with silica gel chromatography (ethyl acetate: petroleum ether, 1:30 (v:v)) as a colorless oil (80% yield).

R*f* = 0.25 (ethyl acetate: petroleum ether, 1:5 (v:v)).

NMR Spectroscopy:

^{1}**H NMR** (400 MHz, Chloroform-*d*, 298 K) δ 8.33 (br, 1H), 7.43 - 7.03 (m, 4H), 3.21 - 3.05 (m, 2H), 2.81 - 2.64 (m, 2H), 2.26 - 2.09 (m, 1H).

^{13}**C NMR** (101 MHz, Chloroform-*d*, 298 K) δ 148.02, 138.54, 129.83, 128.56, 124.23 (q, *J* = 281.0 Hz), 121.26, 120.15 (q, *J* = 278.0 Hz), 49.45 (q, *J* = 27.6 Hz), 32.04, 27.73.

^{19}**F NMR** (376 MHz, Chloroform-*d*, 298 K) δ -57.94, -67.94 (d, *J* = 8.4 Hz).

GC-MS: 316.

HRMS (ESI) m/z $[M+H]^+$: Calculated for $C_{12}H_{11}F_6O_3^+$: 317.0607. Found: 317.0601.

4-(3-(benzyloxy)phenyl)-2-(trifluoromethyl)butanoic acid (**S4**)

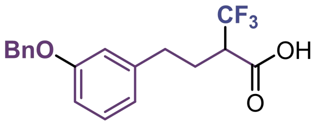

The title compound was purified with silica gel chromatography (ethyl acetate: petroleum ether, 1:20 (v:v)) as a colorless oil (81% yield).

R*f* = 0.25 (ethyl acetate: petroleum ether, 1:5 (v:v)).

NMR Spectroscopy:

^{1}**H NMR** (400 MHz, Chloroform-*d*, 298 K) δ 10.23 (br, 1H), 7.53 - 7.34 (m, 5H), 7.33 - 7.23 (m, 1H), 6.94 - 6.83 (m, 3H), 5.10 (s, 2H), 3.29 - 3.15 (m, 1H), 2.90 - 2.78 (m, 1H), 2.77 - 2.65 (m, 1H), 2.40 - 2.26 (m, 1H), 2.26 - 2.12 (m, 1H).

^{13}**C NMR** (101 MHz, Chloroform-*d*, 298 K) δ 173.42, 159.19, 141.38, 137.09, 129.89, 128.75, 128.16, 127.70, 124.63 (q, *J* = 280.2 Hz), 121.31, 115.48, 113.00, 70.15, 49.52 (q, *J* = 27.7 Hz), 32.79, 27.76.

^{19}**F NMR** (376 MHz, Chloroform-*d*, 298 K) δ -67.68 (d, *J* = 8.1 Hz).

GC-MS: 338.

HRMS (ESI) m/z $[M+H]^+$: Calculated for $C_{18}H_{18}F_3O_3^+$: 339.1203. Found: 339.1200.

4-(naphthalen-1-yl)-2-(trifluoromethyl)butanoic acid (**S5**)

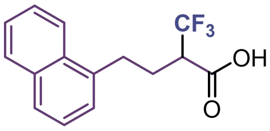

The title compound was purified with silica gel chromatography (ethyl acetate: petroleum ether, 1:30 (v:v)) as a colorless oil (65% yield).

R*f* = 0.30 (ethyl acetate: petroleum ether, 1:5 (v:v)).

NMR Spectroscopy:

^{1}**H NMR** (400 MHz, Chloroform-*d*, 298 K) δ 10.28 (br, 1H), 8.03 (d, *J* = 8.3 Hz, 1H), 7.89 (d, *J* = 8.0 Hz, 1H), 7.77 (d, *J* = 8.1 Hz, 1H), 7.64 - 7.31 (m, 4H), 3.42 - 3.24 (m, 2H), 3.22 - 3.11 (m, 1H), 2.54 - 2.38 (m, 1H), 2.38 - 2.24 (m, 1H).

^{13}**C NMR** (101 MHz, Chloroform-*d*, 298 K) δ 165.08, 135.99, 134.11, 131.71, 129.08, 127.60, 126.54, 126.39, 125.85, 125.63, 124.41 (q, *J* = 280.0 Hz), 123.35, 49.99 (q, *J* = 27.7 Hz), 30.20, 27.17.

^{19}**F NMR** (376 MHz, Chloroform-*d*, 298 K) δ -67.55 (d, *J* = 8.4 Hz).

GC-MS: 282.

HRMS (ESI) m/z $[M+H]^+$: Calculated for $C_{15}H_{14}F_3O_2^+$: 283.0940. Found: 283.0939.

4-(2-bromo-5-methoxyphenyl)-2-(trifluoromethyl)butanoic acid (**S6**)

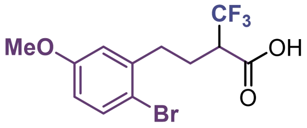

The title compound was purified with silica gel chromatography (ethyl acetate: petroleum ether, 1:20 (v:v)) as a yellow oil (72% yield).

R*f* = 0.25 (ethyl acetate: petroleum ether, 1:5 (v:v)).

NMR Spectroscopy:

^{1}**H NMR** (400 MHz, Chloroform-*d*, 298 K) δ 11.10 (br, 1H), 7.38 (d, *J* = 8.4 Hz, 1H), 6.77 - 6.71 (m, 2H), 3.81 (s, 2H), 3.19 - 3.06 (m, 1H), 2.90 - 2.69 (m, 1H), 2.12 - 1.93 (m, 1H).

^{13}**C NMR** (101 MHz, Chloroform-*d*, 298 K) δ 174.70, 159.52, 140.04, 133.26, 125.11 (q, *J* = 278.1 Hz), 116.96, 115.65, 115.33, 55.30, 46.05 (q, *J* = 27.1 Hz), 33.01, 25.13.

^{19}**F NMR** (376 MHz, Chloroform-*d*, 298 K) δ -68.26 (d, *J* = 8.2 Hz).

GC-MS: 340.

HRMS (ESI) m/z $[M+H]^+$: Calculated for $C_{12}H_{13}BrF_3O_3^+$: 340.9995. Found: 340.9991.

2-(trifluoromethyl)-4-(3-(trifluoromethyl)phenyl)butanoic acid (**S7**)

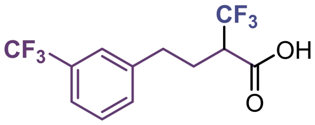

The title compound was purified with silica gel chromatography (ethyl acetate: petroleum ether, 1:30 (v:v)) as a colorless oil (81% yield).

R*f* = 0.2 (ethyl acetate: petroleum ether, 1:10 (v:v)).

NMR Spectroscopy:

^{1}**H NMR** (400 MHz, Chloroform-*d*, 298 K) δ 10.70 (br, 1H), 7.59 - 7.33 (m, 4H), 3.21 – 3.58 (m, 1H), 2.97 - 2.67 (m, 2H), 2.36 - 2.07 (m, 2H).

^{13}**C NMR** (101 MHz, Chloroform-*d*, 298 K) δ 162.18, 140.78, 131.95, 129.20, 127.14 (q, *J* = 279.2 Hz), 125.22, 124.10 (q, *J* = 281.1 Hz), 123.61, 122,09 (q, *J* = 27.6 Hz), 45.05 (q, *J* = 28.0 Hz), 32.63, 27.65.

^{19}**F NMR** (376 MHz, Chloroform-*d*, 298 K) δ -62.56, -67.84 (d, *J* = 7.7 Hz).

GC-MS: 300.

HRMS (ESI) m/z $[M+H]^+$: Calculated for $C_{12}H_{11}F_6O_2^+$: 301.0658. Found: 301.0660.

4-([1,1'-biphenyl]-4-yl)-2-(trifluoromethyl)butanoic acid (**S8**)

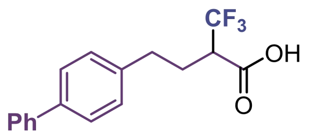

The title compound was purified with silica gel chromatography (ethyl acetate: petroleum ether, 1:20 (v:v)) as a colorless oil (75% yield).

R*f* = 0.3 (ethyl acetate: petroleum ether, 1:5 (v:v)).

NMR Spectroscopy:

^{1}**H NMR** (400 MHz, Chloroform-*d*, 298 K) δ 10.21 (br, 1H), 7.59 (dd, *J* = 14.2, 7.9 Hz, 4H), 7.50 - 7.41 (m, 2H), 7.39 - 7.33 (m, 1H), 7.33 - 7.27 (m, 2H), 3.30 - 3.16 (m, 1H), 2.93 - 2.82 (m, 1H), 2.81 - 2.69 (m, 1H), 2.41 - 2.28 (m, 1H), 2.28 - 2.15 (m, 1H).

^{13}**C NMR** (101 MHz, Chloroform-*d*, 298 K) δ 173.04, 140.91, 139.71, 138.81, 129.03, 128.90, 127.50, 127.36, 127.13, 124.62 (q, *J* = 280.6 Hz), 49.55 (q, *J* = 28.1 Hz), 32.41, 27.84.

^{19}**F NMR** (376 MHz, Chloroform-*d*, 298 K) δ -67.72 (d, *J* = 8.0 Hz).

GC-MS: 308.

HRMS (ESI) m/z $[M+H]^+$: Calculated for $C_{17}H_{16}F_3O_2^+$: 309.1097. Found: 309.1092.

4-(4-acetylphenyl)-2-(trifluoromethyl)butanoic acid (**S9**)

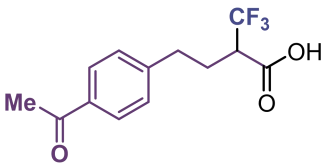

The title compound was purified with silica gel chromatography (ethyl acetate: petroleum ether, 1:20 (v:v)) as a colorless oil (83% yield).

R*f* = 0.2 (ethyl acetate: petroleum ether, 1:5 (v:v)).

NMR Spectroscopy:

^{1}**H NMR** (400 MHz, Chloroform-*d*, 298 K) δ 8.92 (br, 1H), 7.90 (d, *J* = 8.3 Hz, 2H), 7.29 (d, *J* = 8.2 Hz, 2H), 3.20 - 3.07 (m, 1H), 2.91 - 2.80 (m, 1H), 2.78 - 2.69 (m, 1H), 2.59 (s, 3H), 2.35 - 2.23 (m, 1H), 2.19 - 2.07 (m, 1H).

^{13}**C NMR** (101 MHz, Chloroform-*d*, 298 K) δ 198.93, 171.36, 145.82, 135.59, 129.02, 128.84, 124.54 (q, *J* = 280.1 Hz), 49.41 (q, *J* = 28.4, 28.0 Hz), 32.72, 27.40, 26.57.

^{19}**F NMR** (376 MHz, Chloroform-*d*, 298 K) δ -67.85 (d, *J* = 8.5 Hz).

GC-MS: 274.

HRMS (ESI) m/z $[M+H]^+$: Calculated for $C_{13}H_{14}F_3O_3^+$: 275.0890. Found: 275.0881.

4-(4-(2-(((1R,3R,4S)-4-isopropyl-3-methylcyclohexyl)oxy)-2-oxoethyl)phenyl)-2-(trifluoromethyl)butanoic acid (**S10**)

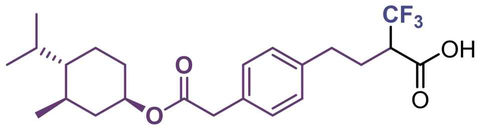

The title compound was purified with silica gel chromatography (ethyl acetate: petroleum ether, 1:30 (v:v)) as a colorless oil (83% yield).

R*f* = 0.3 (ethyl acetate: petroleum ether, 1:5 (v:v)).

NMR Spectroscopy:

^{1}**H NMR** (400 MHz, Chloroform-*d*, 298 K) δ 7.44 (br, 1H), 7.24 - 7.11 (m, 3H), 3.58 (s, 2H), 3.20 - 3.06 (m, 1H), 2.83 - 2.73 (m, 1H), 2.71 - 2.59 (m, 1H), 2.30 - 2.18 (m, 1H), 2.17 - 2.05 (m, 1H), 1.96 (d, *J* = 12.2 Hz, 1H), 1.77 - 1.60 (m, 3H), 1.49 - 1.30 (m, 2H), 1.05 - 0.93 (m, 2H), 0.91 - 0.78 (m, 8H), 0.68 (d, *J* = 6.9 Hz, 3H).

^{13}**C NMR** (101 MHz, Chloroform-*d*, 298 K) δ 173.51, 171.79, 138.44, 132.67, 129.58, 128.71, 124.58 (q, *J* = 279.1 Hz), 75.09, 49.23 (q, *J* = 28.0 Hz), 47.10, 41.45, 40.83, 34.30, 32.35, 31.44, 27.80, 26.24, 23.49, 22.06, 20.73, 16.31.

^{19}**F NMR** (376 MHz, Chloroform-*d*, 298 K) δ -67.88 (d, *J* = 8.2 Hz).

HRMS (ESI) m/z $[M+H]^+$: Calculated for $C_{23}H_{32}F_3O_4^+$: 429.2247. Found: 429.2240.

4-(4-(2-oxo-2-(((R)-2,5,7,8-tetramethyl-2-((4R,8R)-4,8,12-trimethyltridecyl)chroman-6-yl)oxy)ethyl)phenyl)-2-(trifluoromethyl)butanoic acid (**S11**)

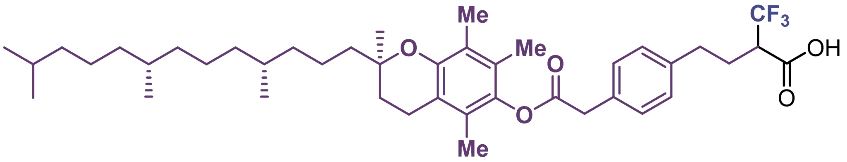

The title compound was purified with silica gel chromatography (ethyl acetate: petroleum ether, 1:20 (v:v)) as a colorless oil (70% yield).

R*f* = 0.35 (ethyl acetate: petroleum ether, 1:3 (v:v)).

NMR Spectroscopy:

^{1}**H NMR** (400 MHz, Chloroform-*d*, 298 K) δ 8.82 (br, 1H), 7.40 (d, *J* = 7.7 Hz, 2H), 7.22 (d, *J* = 7.7 Hz, 2H), 3.90 (s, 2H), 3.22 - 3.08 (m, 1H), 2.90 - 2.79 (m, 1H), 2.76 - 2.64 (m, 1H), 2.58 (t, *J* = 6.9 Hz, 2H), 2.37 - 2.23 (m, 1H), 2.22 - 2.11 (m, 4H), 1.92 (s, 3H), 1.87 (s, 3H), 1.84 - 1.72 (m, 2H), 1.44 - 1.08 (m, 21H), 0.99 - 0.82 (m, 12H).

^{13}**C NMR** (101 MHz, Chloroform-*d*, 298 K) δ 172.38, 170.62, 149.58, 140.58, 138.85, 132.18, 129.95, 128.92, 126.78, 125.02, 124.62 (q, *J* = 278.4 Hz), 123.23, 117.55, 75.19, 49.34 (q, *J* = 27.8 Hz), 41.08, 39.51, 37.55, 37.42, 32.92, 32.82, 32.37, 31.22, 28.10, 27.84, 24.93, 24.57, 22.84, 22.75, 21.13, 20.67, 19.88, 19.77, 12.84, 11.97, 11.88.

^{19}F NMR (376 MHz, Chloroform-*d*, 298 K) δ -67.79 (d, *J* = 8.3 Hz).

HRMS (ESI) m/z $[M+H]^+$: Calculated for $C_{42}H_{62}F_3O_5^+$: 703.4544. Found: 703.4539.

Preparation of the substrates (Method 2) [1, 2]

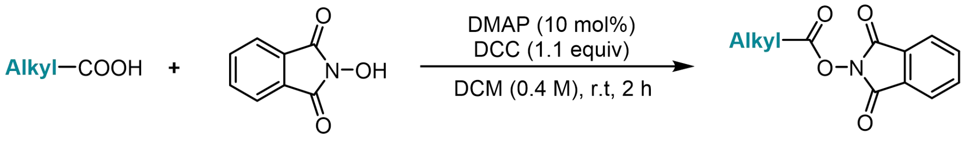

A reaction tube equipped with a magnetic stir bar was charged with alkyl carboxylic acid (4 mmol, 1.0 equiv), *N*-hydroxylphthalimide (4.4 mmol, 1.1 equiv), DMAP (280 mg, 0.4 mmol, 10 mol%) and DCM (5 mL) under nitrogen atmosphere. Then, a solution of DCC (4.4 mmol) in 5 mL DCM was added slowly at room temperature. The mixture was stirred at room temperature for 2 hours. the white precipitate was filtered off and the solution was concentrated on a rotary evaporator. The residue was purified by chromatography on silica gel to afford corresponding products, which was characterized by ^{1}H NMR and ^{13}C NMR spectroscopy.

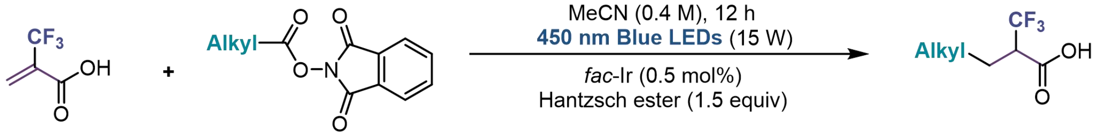

A reaction tube equipped with a magnetic stir bar was charged with *fac*-Ir(ppy)$_3$ (6.5 mg, 0.5 mol %), Hantzsch ester 1 (675 mg, 3.0 mmol, 1.5 equiv), 3,3,3-trifluoropropionic acid (280 mg, 2.0 mmol, 1.0 equiv), redox-active esters (3.0 mmol, 1.5 equiv) and MeCN (5 mL) under nitrogen atmosphere. The mixture was stirred under irradiation of 15 W blue LEDs at room temperature for 12 hours. Then the mixture was diluted with H_2O and the resulting mixture was extracted with DCM for 3 times. The combined organic layers were washed with brine, dried over Na_2SO_4, filtered, and concentrated under reduced pressure. The residue was purified by chromatography on silica gel to afford corresponding products, which was characterized by GC-MS analysis, ^{1}H NMR, ^{13}C NMR and ^{19}F NMR spectroscopy.

1,3-dioxoisoindolin-2-yl 5-(2,5-dimethylphenoxy)-2,2-dimethylpentanoate (**S12**)

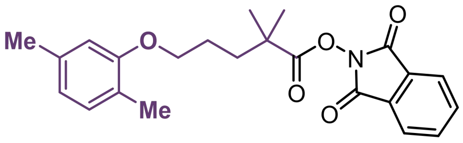

The title compound was purified with silica gel chromatography (ethyl acetate: petroleum ether,

1:20 (v:v)) as a colorless oil (90% yield).

R_f = 0.35 (ethyl acetate: petroleum ether, 1:3 (v:v)).

NMR Spectroscopy:

^{1}H NMR (400 MHz, Chloroform-*d*, 298 K) δ 7.91 - 7.84 (m, 2H), 7.82 - 7.74 (m, 2H), 7.00 (d, *J* = 6.0 Hz, 1H), 6.68 - 6.59 (m, 2H), 4.06 - 3.95 (m, 2H), 2.31 (s, 3H), 2.19 (s, 3H), 2.02 - 1.87 (m, 4H), 1.45 (s, 6H).

^{13}C NMR (101 MHz, Chloroform-*d*, 298 K) δ 173.87, 162.20, 157.06, 136.58, 134.78, 130.36, 129.15, 123.97, 123.70, 120.79, 112.10, 67.82, 42.06, 37.48, 25.22, 25.08, 21.48, 15.87.

HRMS (ESI) m/z $[M+H]^+$: Calculated for $C_{23}H_{26}NO_5^+$: 396.1805. Found: 396.1800.

7-(2,5-dimethylphenoxy)-4,4-dimethyl-2-(trifluoromethyl)heptanoic acid (**S13**)

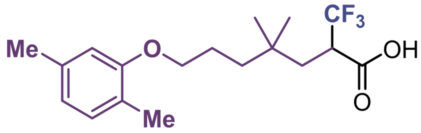

The title compound was purified with silica gel chromatography (ethyl acetate: petroleum ether, 1:30 (v:v)) as a colorless oil (73% yield).

R_f = 0.35 (ethyl acetate: petroleum ether, 1:5 (v:v)).

NMR Spectroscopy:

^{1}H NMR (400 MHz, Chloroform-*d*, 298 K) δ 9.50 (br, 1H), 7.00 - 6.94 (m, 1H), 6.72 - 6.68 (m, 1H), 6.65 (d, *J* = 2.4 Hz, 1H), 3.96 (t, *J* = 5.6 Hz, 2H), 3.30 - 3.21 (m, 3H), 2.30 (s, 3H), 2.18 (s, 3H), 1.93 - 1.85 (m, 1H), 1.80 - 1.71 (m, 1H), 1.71 - 1.59 (m, 2H), 1.40 - 1.24 (m, 2H), 0.98 (s, 3H), 0.93 (s, 3H).

^{13}C NMR (101 MHz, Chloroform-*d*, 298 K) δ 174.53, 157.42, 137.21, 130.45, 124.92, 124.21 (q, *J* = 278.0 Hz), 121.82, 113.17, 69.90, 46.90 (q, *J* = 26.9 Hz), 39.59, 37.71, 32.61, 29.38, 24.75, 21.63, 15.87.

^{19}F NMR (376 MHz, Chloroform-*d*, 298 K) δ -67.52 (d, *J* = 8.2 Hz).

HRMS (ESI) m/z $[M+H]^+$: Calculated for $C_{18}H_{26}F_3O_3^+$: 347.1829. Found: 347.1833.

1,3-dioxoisoindolin-2-yl 2-(1-(4-chlorobenzoyl)-5-methoxy-2-methyl-1H-indol-3-yl)acetate (**S14**)

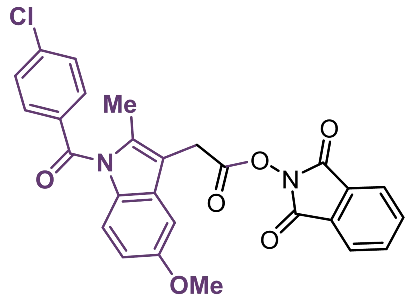

The title compound was purified with silica gel chromatography (ethyl acetate: petroleum ether, 1:20 (v:v)) as a colorless oil (87% yield).

R_f = 0.45 (ethyl acetate: petroleum ether, 1:2 (v:v)).

NMR Spectroscopy:

^{1}H NMR (400 MHz, Chloroform-*d*, 298 K) δ 7.94 - 7.89 (m, 2H), 7.67 (d, *J* = 8.5 Hz, 2H), 7.46 (d, *J* = 8.6 Hz, 2H), 7.42 - 7.31 (m, 1H), 7.02 (d, *J* = 2.5 Hz, 1H), 6.92 (d, *J* = 9.0 Hz, 1H), 6.74 - 6.60 (m, 2H), 3.88 (s, 3H), 2.41 (s, 3H), 2.03 (s, 2H).

^{13}C NMR (101 MHz, Chloroform-*d*, 298 K) δ 163.81, 162.52, 161.73, 156.25, 152.00, 139.62, 136.24, 134.93, 134.82, 131.40, 131.32, 129.27, 129.17, 124.11, 124.06, 116.82, 112.30, 55.82, 29.04, 27.27.

HRMS (ESI) m/z $[M+H]^+$: Calculated for $C_{27}H_{20}{}^{35}ClN_2O_6{}^+$: 503.1004. Found: 503.1010.

4-(1-(4-chlorobenzoyl)-5-methoxy-2-methyl-1H-indol-3-yl)-2-(trifluoromethyl)butanoic acid (**S15**)

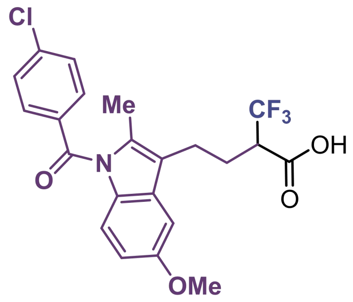

The title compound was purified with silica gel chromatography (ethyl acetate: petroleum ether, 1:20 (v:v)) as a colorless oil (87% yield).

R_f = 0.45 (ethyl acetate: petroleum ether, 1:2 (v:v)).

NMR Spectroscopy:

^{1}H NMR (400 MHz, Chloroform-*d*, 298 K) δ 8.17 (br, 1H), 7.64 (d, *J* = 6.2 Hz, 2H), 7.46 (d, *J* = 6.3 Hz, 2H), 6.93 - 6.84 (m, 2H), 6.67 (d, *J* = 9.0 Hz, 1H), 3.82 (s, 3H), 3.29 - 3.18 (m, 1H), 2.83 - 2.72 (m, 2H), 2.35 - 2.21 (m, 4H), 2.20 - 2.08 (m, 1H).

^{13}C NMR (101 MHz, Chloroform-*d*, 298 K) δ 172.29, 168.58, 156.11, 139.37, 134.81, 134.02, 131.24, 131.11, 130.62, 129.28, 124.52 (q, *J* = 279.8 Hz), 117.51, 115.27, 111.52, 101.14, 55.76, 49.68 (q, *J* = 27.6 Hz), 25.94, 21.32, 13.20.

^{19}F NMR (376 MHz, Chloroform-*d*, 298 K) δ -67.54 (d, *J* = 8.3 Hz).

HRMS (ESI) m/z $[M+H]^+$: Calculated for $C_{22}H_{20}{}^{35}ClF_3NO_4{}^+$: 454.1027. Found: 454.1030.

The substrate scope of the decarboxylative cross-coupling

(*R*)-*N*-(4-(1,1,1-trifluoro-4-phenylbutan-2-yl)phenyl)acetamide (**3a**)

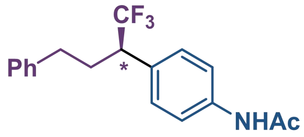

The product was obtained as a white solid (44.9 mg, 70 % yield).

R*f* = 0.25 (ethyl acetate: petroleum ether, 1:2 (v:v)).

NMR Spectroscopy:

^{1}H NMR (400 MHz, Chloroform-*d*, 298 K) δ 7.64 (s, 1H), 7.54 (d, *J* = 7.5 Hz, 2H), 7.31 - 7.15 (m, 5H), 7.06 (d, *J* = 7.9 Hz, 2H), 3.25 - 3.10 (m, 1H), 2.62 - 2.51 (m, 1H), 2.44 - 2.22 (m, 2H), 2.21 - 2.06 (m, 4H).

^{13}C NMR (101 MHz, Chloroform-*d*, 298 K) δ 168.74, 140.61, 138.07, 130.18, 129.88, 128.62, 128.49, 126.35, 126.96 (q, *J* = 281.5 Hz), 120.13, 48.60 (q, *J* = 26.3 Hz), 32.48, 30.09, 24.65.

^{19}F NMR (376 MHz, Chloroform-*d*, 298 K) δ -69.75 (d, *J* = 9.4 Hz).

GC-MS: 321.

HRMS (ESI) m/z $[M+H]^+$: Calculated for $C_{18}H_{19}F_3NO^+$: 322.1413. Found: 322.1404.

HPLC analysis: The enantiomeric ratio was determined on a Daicel Chiralpak OD-H Column, *n*-Hexane: *i*-PrOH = 85: 15, 1.0 mL/min, T = 25 ℃, λ = 254 nm. Retention time t_1 = 9.610 min (major), t_2 = 11.070 min (minor), er = 93:7.

$[\alpha]_D^{20}$ = -31.2° (c = 0.2, $CHCl_3$)

(R)-N-(2-fluoro-4-(1,1,1-trifluoro-4-phenylbutan-2-yl)phenyl)cyclohexanecarboxamide (**3b**)

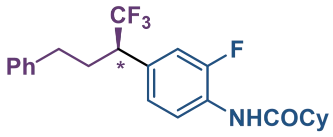

The product was obtained as a white solid (52.1 mg,64 % yield).

R*f* = 0.25 (ethyl acetate: petroleum ether, 1:2 (v:v)).

NMR Spectroscopy:

^{1}H NMR (400 MHz, Chloroform-*d*, 298 K) δ 8.41 (dt, *J* = 9.5, 4.8 Hz, 1H), 7.45 (s, 1H), 7.31 - 7.17 (m, 3H), 7.11 - 7.01 (m, 4H), 3.24 - 3.08 (m, 1H), 2.65 - 2.54 (m, 1H), 2.45 - 2.25 (m, 3H), 2.20 - 2.10 (m, 1H), 1.98 (d, *J* = 12.5 Hz, 2H), 1.85 (d, *J* = 12.6 Hz, 2H), 1.72 (d, *J* = 9.6 Hz, 1H), 1.63 - 1.49 (m, 2H), 1.40 - 1.23 (m, 3H).

^{13}C NMR (101 MHz, Chloroform-*d*, 298 K) δ 174.59, 152.35 (d, *J* = 243.0 Hz), 140.34, 130.48 (d, *J* = 6.4 Hz), 128.68, 128.48, 126.72 (d, *J* = 10.1 Hz), 126.71 (q, *J* = 281.1 Hz),

126.45, 125.86, 121.75, 115.20 (d, J = 20.2 Hz), 48.52 (q, J = 26.6 Hz), 46.63, 32.40, 30.05, 29.71, 25.68.

^{19}F NMR (376 MHz, Chloroform-*d*, 298 K) δ -69.78 (d, J = 8.8 Hz), -130.65.

GC-MS: 407.

HRMS (ESI) m/z $[M+H]^+$: Calculated for $C_{23}H_{26}F_4NO^+$: 408.1945. Found: 408.1944.

HPLC analysis: The enantiomeric ratio was determined on a Daicel Chiralpak OD-H Column, *n*-Hexane: *i*-PrOH = 93: 7, 1.0 mL/min, T = 25 ℃, λ = 254 nm. Retention time t_1 = 7.507 min (major), t_2 = 8.349 min (minor), er = 92:8.

$[\alpha]_D^{20}$ = -38.5° (c = 0.2, $CHCl_3$).

Melt point: 78.4 - 79.1℃.

(R)-1-methyl-4-(1,1,1-trifluoro-4-phenylbutan-2-yl)benzene (**3c**)

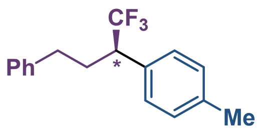

The product was obtained as a colorless oil (44.5 mg, 80 % yield).

R_f = 0.70 (ethyl acetate: petroleum ether, 1:20 (v:v)).

NMR Spectroscopy:

^{1}H NMR (400 MHz, Chloroform-*d*, 298 K) δ 7.31 - 7.18 (m, 7H), 7.11 - 7.05 (m, 2H), 3.24 - 3.10 (m, 1H), 2.63 - 2.52 (m, 1H), 2.45 - 2.27 (m, 5H), 2.24 - 2.14 (m, 1H).

^{13}C NMR (101 MHz, Chloroform-*d*, 298 K) δ 140.80, 138.11, 131.41, 129.57, 129.14, 128.59, 128.52, 126.69 (q, J = 281.0 Hz), 126.29, 48.81 (q, J = 26.3 Hz), 32.57, 30.17, 21.24.

^{19}F NMR (376 MHz, Chloroform-*d*, 298 K) δ -69.75 (d, J = 9.4 Hz).

GC-MS: 278.

HRMS (ESI) m/z $[M+H]^+$: Calculated for $C_{17}H_{18}F_3^+$: 279.1355. Found: 279.1355.

HPLC analysis: The enantiomeric ratio was determined on a Daicel Chiralpak IC Column, *n*-Hexane: *i*-PrOH = 99: 1, 1.0 mL/min, T = 25 ℃, λ = 254 nm. Retention time t_1 = 6.322 min (minor), t_2 = 8.819 min (major), er = 95:5.

$[\alpha]_D^{20}$ = -52.4° (c = 0.2, $CHCl_3$)

(R)-1-(tert-butyl)-4-(1,1,1-trifluoro-4-phenylbutan-2-yl)benzene (**3d**)

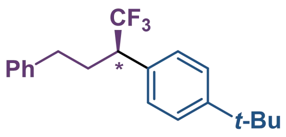

The product was obtained as a colorless oil (49.1 mg, 77 % yield).

R_f = 0.75 (ethyl acetate: petroleum ether, 1:20 (v:v)).

NMR Spectroscopy:

^{1}H NMR (400 MHz, Chloroform-*d*, 298 K) δ 7.43 - 7.37 (m, 2H), 7.33 - 7.18 (m, 5H), 7.15 - 7.09 (m, 2H), 3.29 - 3.11 (m, 1H), 2.65 - 2.55 (m, 1H), 2.50 - 2.39 (m, 1H), 2.38 - 2.28 (m, 1H), 2.28 - 2.15 (m, 1H), 1.38 (s, 9H).

^{13}C NMR (101 MHz, Chloroform-*d*, 298 K) δ 151.20, 150.02, 140.89, 138.31, 128.88, 128.59, 127.50 (q, *J* = 284.4 Hz), 126.79, 125.75, 48.80 (q, *J* = 26.2 Hz), 34.65, 32.70, 31.51, 30.29.

^{19}F NMR (376 MHz, Chloroform-*d*, 298 K) δ -69.56 (d, *J* = 8.7 Hz).

GC-MS: 320.

HRMS (ESI) m/z $[M+H]^+$: Calculated for $C_{20}H_{24}F_3^+$: 321.1825. Found: 321.1829.

HPLC analysis: The enantiomeric ratio was determined on a Daicel Chiralpak OJ-H Column, *n*-Hexane: *i*-PrOH = 99: 1, 1.0 mL/min, T = 25 ℃, λ = 254 nm. Retention time t_1 = 4.743 min (major), t_2 = 6.306 min (minor), er = 92:8.

$[\alpha]_D^{20}$ = -44.5° (c = 0.2, $CHCl_3$)

(R)-5-(1,1,1-trifluoro-4-phenylbutan-2-yl)benzo[d][1,3]dioxole (**3e**)

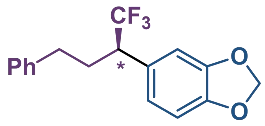

The product was obtained as a colorless oil (44.9 mg, 73 % yield).

R_f = 0.45 (ethyl acetate: petroleum ether, 1:20 (v:v)).

NMR Spectroscopy:

^{1}H NMR (400 MHz, Chloroform-*d*, 298 K) δ 7.31 - 7.07 (m, 5H), 6.85 - 6.78 (m, 2H), 6.76 - 6.70 (m, 1H), 5.99 (s, 2H), 3.12 (ddq, *J* = 18.5, 9.3, 4.7, 3.9 Hz, 1H), 2.60 (ddd, *J* = 13.9, 9.0, 4.9 Hz, 1H), 2.47 - 2.36 (m, 1H), 2.30 (dtd, *J* = 12.5, 8.4, 3.8 Hz, 1H), 2.19 - 2.06 (m, 1H).

^{13}C NMR (101 MHz, Chloroform-*d*, 298 K) δ 148.17, 147.65, 140.67, 128.62, 128.51, 126.35, 126.43 (q, *J* = 281.0 Hz), 123.18, 108.98, 108.50, 101.36, 48.88 (q, *J* = 26.7 Hz), 32.51, 30.26.

^{19}F NMR (376 MHz, Chloroform-*d*, 298 K) δ -69.93 (d, *J* = 9.1 Hz).

GC-MS: 308.

HRMS (ESI) m/z $[M+H]^+$: Calculated for $C_{17}H_{16}F_3O_2^+$: 309.1097. Found: 309.1100.

HPLC analysis: The enantiomeric ratio was determined on a Daicel Chiralpak AD-H Column, *n*-Hexane: *i*-PrOH = 99: 1, 1.0 mL/min, T = 25 ℃, λ = 254 nm. Retention time t_1 = 8.725 min (minor), t_2 = 11.336 min (major), er = 92:8.

$[\alpha]_D^{20}$ = -42.2° (c = 0.2, $CHCl_3$)

(R)-4-(1,1,1-trifluoro-4-phenylbutan-2-yl)benzaldehyde (**3f**)

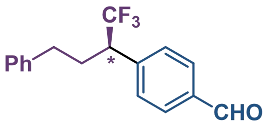

The product was obtained as a white solid (40.3 mg, 69 % yield).

R_f = 0.30 (ethyl acetate: petroleum ether, 1:5 (v:v)).

NMR Spectroscopy:

^{1}H NMR (400 MHz, Chloroform-*d*, 298 K) δ 10.06 (s, 1H), 8.19 - 8.13 (m, 1H), 7.96 - 7.89 (m, 1H), 7.50 - 7.40 (m, 2H), 7.31 - 7.18 (m, 3H), 7.11 - 7.04 (m, 2H), 3.38 - 3.26 (m, 1H), 2.65 - 2.53 (m, 1H), 2.48 - 2.36 (m, 2H), 2.33 - 2.19 (m, 1H).

^{13}C NMR (101 MHz, Chloroform-*d*, 298 K) δ 191.85, 136.41, 130.76, 130.19, 130.05, 129.54, 128.73, 128.44, 126.59 (q, *J* = 281.0 Hz), 126.55, 49.37 (q, *J* = 26.5 Hz), 32.48, 30.11.

^{19}F NMR (376 MHz, Chloroform-*d*, 298 K) δ -69.13 (d, *J* = 9.4 Hz).

GC-MS: 292.

HRMS (ESI) m/z $[M+H]^+$: Calculated for $C_{17}H_{16}F_3O^+$: 293.1148. Found: 293.1146.

HPLC analysis: The enantiomeric ratio was determined on a Daicel Chiralpak OJ-H Column, *n*-Hexane: *i*-PrOH = 94: 6, 1.0 mL/min, T = 25 ℃, λ = 254 nm. Retention time t_1 = 16.771 min (major), t_2 = 20.950 min (minor), er = 94:6.

$[\alpha]_D^{20}$ = -81.3° (c = 0.2, $CHCl_3$)

(R)-4-(1,1,1-trifluoro-4-phenylbutan-2-yl)benzaldehyde (**3g**)

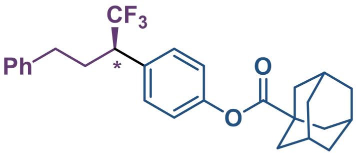

The product was obtained as a white solid (65.1 mg, 74 % yield).

R_f = 0.55 (ethyl acetate: petroleum ether, 1:10 (v:v)).

NMR Spectroscopy:

^{1}H NMR (400 MHz, Chloroform-*d*, 298 K) δ 7.32 - 7.16 (m, 5H), 7.14 - 7.05 (m, 4H), 3.28 - 3.15 (m, 1H), 2.66 - 2.56 (m, 1H), 2.47 - 2.28 (m, 2H), 2.25 - 2.13 (m, 1H), 2.14 - 2.02 (m, 9H), 1.83 - 1.73 (m, 6H).

^{13}C NMR (101 MHz, Chloroform-*d*, 298 K) δ 176.11, 151.15, 140.54, 131.65, 130.20, 128.64, 128.51, 126.10 (q, *J* = 281.2 Hz), 126.37, 121.99, 48.63 (q, *J* = 26.4 Hz), 41.17, 38.86, 36.54, 32.49, 30.19, 28.00.

^{19}F NMR (376 MHz, Chloroform-*d*, 298 K) δ -69.69 (d, *J* = 9.3 Hz).

GC-MS: 442.

HRMS (ESI) m/z $[M+H]^+$: Calculated for $C_{27}H_{30}F_3O_2^+$: 443.2192. Found: 443.2189.

HPLC analysis: The enantiomeric ratio was determined on a Daicel Chiralpak OJ-H Column, *n*-Hexane: *i*-PrOH = 95: 5, 1.0 mL/min, T = 25 ℃, λ = 254 nm. Retention time t_1 = 7.849 min (minor), t_2 = 9.887 min (major), er = 91:9.

$[\alpha]_D^{20}$ = -51.1° (c = 0.2, $CHCl_3$).

Melt point: 65.2 - 67.0℃.

Methyl (R)-4-(1,1,1-trifluoro-4-phenylbutan-2-yl)benzoate (**3h**)

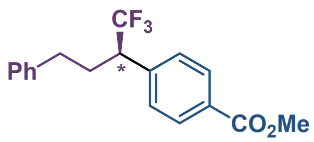

The product was obtained as a colorless oil (45.0 mg, 70 % yield).

R_f = 0.60 (ethyl acetate: petroleum ether, 1:10 (v:v)).

NMR Spectroscopy:

^{1}H NMR (400 MHz, Chloroform-*d*, 298 K) δ 8.08 (dd, *J* = 8.2, 2.7 Hz, 2H), 7.38 (d, *J* = 5.6 Hz, 2H), 7.32 - 7.17 (m, 3H), 7.08 (d, *J* = 6.6 Hz, 2H), 3.95 (s, 3H), 3.37 - 3.23 (m, 1H), 2.64 - 2.52 (m, 1H), 2.45 - 2.35 (m, 2H), 2.32 - 2.17 (m, 1H).

^{13}C NMR (101 MHz, Chloroform-*d*, 298 K) δ 166.76, 140.27, 139.65, 130.36, 130.12, 129.40, 128.70, 128.47, 126.70 (q, *J* = 281.1 Hz), 126.49, 52.30, 49.21 (q, *J* = 26.7 Hz), 32.48, 30.12.

^{19}F NMR (376 MHz, Chloroform-*d*, 298 K) δ -69.25 (d, *J* = 9.3 Hz).

GC-MS: 322.

HRMS (ESI) m/z $[M+H]^+$: Calculated for $C_{18}H_{18}F_3O_2^+$: 323.1253. Found: 323.1250.

HPLC analysis: The enantiomeric ratio was determined on a Daicel Chiralpak OJ-H Column, *n*-Hexane: *i*-PrOH = 94: 6, 1.0 mL/min, T = 25 ℃, λ = 254 nm. Retention time t_1 = 9.269 min (minor), t_2 = 10.290 min (major), er = 93:7.

$[\alpha]_D^{20}$ = -58.0° (c = 0.2, $CHCl_3$)

(R)-4-(1,1,1-trifluoro-4-phenylbutan-2-yl)-1,1'-biphenyl (**3i**)

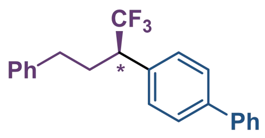

The product was obtained as a white solid (56.5 mg, 83 % yield).

R_f = 0.40 (ethyl acetate: petroleum ether, 1:20 (v:v)).

NMR Spectroscopy:

^{1}H NMR (400 MHz, Chloroform-*d*, 298 K) δ 7.69 - 7.63 (m, 4H), 7.54 - 7.45 (m, 2H), 7.44 - 7.38 (m, 3H), 7.37 - 7.29 (m, 2H), 7.29 - 7.23 (m, 1H), 7.19 - 7.12 (m, 2H), 3.38 - 3.24 (m,

1H), 2.73 - 2.62 (m, 1H), 2.55 - 2.37 (m, 2H), 2.36 - 2.23 (m, 1H).

^{13}C NMR (101 MHz, Chloroform-*d*, 298 K) δ 141.30, 140.73, 140.63, 133.54, 129.73, 128.98, 128.68, 128.58, 127.65, 127.60, 127.25, 127.10 (q, *J* = 281.1 Hz), 126.40, 48.97 (q, *J* = 26.6 Hz), 32.65, 30.26.

^{19}F NMR (376 MHz, Chloroform-*d*, 298 K) δ -69.42 (d, *J* = 9.5 Hz).

GC-MS: 340.

HRMS (ESI) m/z $[M+H]^+$: Calculated for $C_{22}H_{20}F_3^+$: 341.1512. Found: 341.1519.

HPLC analysis: The enantiomeric ratio was determined on a Daicel Chiralpak OJ-H Column, *n*-Hexane: *i*-PrOH = 94: 6, 1.0 mL/min, T = 25 ℃, λ = 254 nm. Retention time t_1 = 10.735 min (minor), t_2 = 12.286 min (major), er = 96:4.

$[\alpha]_D^{20}$ = -66.9° (c = 0.2, $CHCl_3$).

(R)-1-(4-(1,1,1-trifluoro-4-phenylbutan-2-yl)phenyl)ethan-1-one (**3j**)

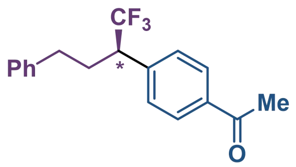

The product was obtained as a white solid (39.8 mg, 65 % yield).

R*f* = 0.30 (ethyl acetate: petroleum ether, 1:10 (v:v)).

NMR Spectroscopy:

^{1}H NMR (400 MHz, Chloroform-*d*, 298 K) δ 7.98 (dd, *J* = 8.4, 2.4 Hz, 2H), 7.43 - 7.37 (m, 2H), 7.32 - 7.17 (m, 3H), 7.10 - 7.04 (m, 2H), 3.37 - 3.23 (m, 1H), 2.66 - 2.53 (m, 4H), 2.45 - 2.32 (m, 2H), 2.32 - 2.17 (m, 1H).

^{13}C NMR (101 MHz, Chloroform-*d*, 298 K) δ 197.71, 140.23, 139.82, 137.17, 129.60, 128.86, 128.70, 128.46, 126.66 (q, *J* = 281.1 Hz), 126.51, 49.17 (q, *J* = 26.9 Hz), 32.46, 30.08, 26.75.

^{19}F NMR (376 MHz, Chloroform-*d*, 298 K) δ -69.24 (d, *J* = 9.6 Hz).

GC-MS: 306.

HRMS (ESI) m/z $[M+H]^+$: Calculated for $C_{18}H_{18}F_3O^+$: 307.1304. Found: 307.1300.

HPLC analysis: The enantiomeric ratio was determined on a Daicel Chiralpak OD-H Column, *n*-Hexane: *i*-PrOH = 97: 3, 1.0 mL/min, T = 25 ℃, λ = 254 nm. Retention time t_1 = 10.816 min (major), t_2 = 12.041 min (minor), er = 92:8.

$[\alpha]_D^{20}$ = -57.5° (c = 0.2, $CHCl_3$)

(R)-N-methyl-4-(1,1,1-trifluoro-4-phenylbutan-2-yl)benzamide (**3k**)

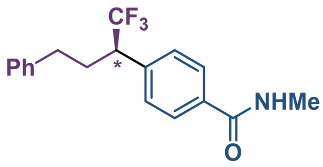

The product was obtained as a yellow solid (37.2 mg, 58 % yield).

R*f* = 0.45 (ethyl acetate: petroleum ether, 1:1 (v:v)).

NMR Spectroscopy:

^{1}H NMR (400 MHz, Chloroform-*d*, 298 K) δ 7.79 (d, *J* = 8.3 Hz, 2H), 7.36 - 7.16 (m, 5H), 7.05 (d, *J* = 7.7 Hz, 2H), 6.74 (s, 1H), 3.32 - 3.18 (m, 1H), 2.98 (d, *J* = 4.8 Hz, 3H), 2.60 - 2.48 (m, 1H), 2.43 - 2.28 (m, 2H), 2.27 - 2.13 (m, 1H).

^{13}C NMR (101 MHz, Chloroform-*d*, 298 K) δ 168.08, 140.31, 137.89, 134.82, 129.45, 128.66, 128.44, 127.51, 126.76 (q, *J* = 281.5 Hz), 126.45, 49.04 (q, *J* = 26.8 Hz), 32.44, 30.06, 26.92.

^{19}F NMR (376 MHz, Chloroform-*d*, 298 K) δ -69.33 (d, *J* = 9.4 Hz).

GC-MS: 321.

HRMS (ESI) m/z $[M+H]^+$: Calculated for $C_{18}H_{19}F_3NO^+$: 322.1413. Found: 322.1412.

HPLC analysis: The enantiomeric ratio was determined on a Daicel Chiralpak AD-H Column, *n*-Hexane: *i*-PrOH = 95: 5, 1.0 mL/min, T = 25 ℃, λ = 254 nm. Retention time t_1 = 30.227 min (major), t_2 = 40.835 min (minor), er = 95:5.

$[\alpha]_D^{20}$ = -43.0° (c = 0.2, $CHCl_3$)

Melt point: 72.4 - 73.8℃.

(R)-1,2-dichloro-4-(1,1,1-trifluoro-4-phenylbutan-2-yl)benzene (**3l**)

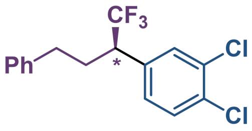

The product was obtained as a white solid (54.3 mg, 82 % yield).

R*f* = 0.70 (ethyl acetate: petroleum ether, 1:20 (v:v)).

NMR Spectroscopy:

^{1}H NMR (400 MHz, Chloroform-*d*, 298 K) δ 7.64 - 7.59 (m, 2H), 7.55 - 7.44 (m, 3H), 7.39 - 7.33 (m, 3H), 3.22 - 3.11 (m, 1H), 2.65 - 2.56 (m, 1H), 2.46 - 2.29 (m, 2H), 2.25 - 2.08 (m, 1H).

^{13}C NMR (101 MHz, Chloroform-*d*, 298 K) δ 138.82, 132.57, 131.22, 131.06, 130.87, 128.90, 128.74, 128.43, 127.15 (q, *J* = 288.2 Hz), 126.57, 126.25, 48.67 (q, *J* = 26.7 Hz), 32.41, 29.95.

^{19}F NMR (376 MHz, Chloroform-*d*, 298 K) δ -69.58 (d, *J* = 9.3 Hz).

GC-MS: 332.

HRMS (ESI) m/z $[M+H]^+$: Calculated for $C_{16}H_{14}{}^{35}Cl\ Cl_2F_3{}^+$: 333.0419. Found: 333.0421.

HPLC analysis: The enantiomeric ratio was determined on a Daicel Chiralpak OJ-H Column, *n*-Hexane: *i*-PrOH = 99: 1, 1.0 mL/min, T = 25 ℃, λ = 254 nm. Retention time t_1 = 10.717 min (minor), t_2 = 17.706 min (major), er = 94:6.

$[\alpha]_D^{20}$ = -43.0° (c = 0.2, $CHCl_3$)

(R)-5-(1,1,1-trifluoro-4-phenylbutan-2-yl)-1H-indole (**3m**)

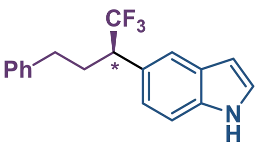

The product was obtained as a white solid (26.6 mg, 44 % yield).

R_f = 0.40 (ethyl acetate: petroleum ether, 1:3 (v:v)).

NMR Spectroscopy:

^{1}**H NMR** (400 MHz, Chloroform-*d*, 298 K) δ 8.18 (s, 1H), 7.60 (s, 1H), 7.41 (d, *J* = 8.5 Hz, 1H), 7.31 - 7.08 (m, 7H), 6.58 (s, 1H), 3.39 - 3.26 (m, 1H), 2.65 - 2.55 (m, 1H), 2.51 - 2.26 (m, 3H).

^{13}**C NMR** (101 MHz, Chloroform-*d*, 298 K) δ 141.14, 135.69, 128.85, 128.56, 128.20, 126.40 (q, *J* = 281.0 Hz), 126.21, 125.76, 124.98, 123.04, 121.70, 111.38, 102.84, 49.35 (q, *J* = 26.4 Hz), 32.72, 30.62.

^{19}**F NMR** (376 MHz, Chloroform-*d*, 298 K) δ -69.65 (d, *J* = 9.5 Hz).

GC-MS: 303.

HRMS (ESI) m/z $[M+H]^+$: Calculated for $C_{18}H_{17}F_3N^+$: 304.1308. Found: 304.1319.

HPLC analysis: The enantiomeric ratio was determined on a Daicel Chiralpak AD-H Column, *n*-Hexane: *i*-PrOH = 96: 4, 1.0 mL/min, T = 25 ℃, λ = 254 nm. Retention time t_1 = 12.010 min (major), t_2 = 22.837 min (minor), er = 92:8.

$[\alpha]_D^{20}$ = -50.2° (c = 0.2, $CHCl_3$)

(R)-4-(1,1,1-trifluoro-4-phenylbutan-2-yl)dibenzo[b,d]thiophene (**3n**)

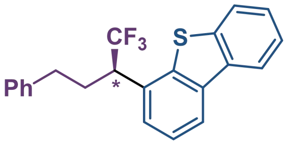

The product was obtained as a colorless solid (38.5 mg, 52 % yield).

R_f = 0.50 (ethyl acetate: petroleum ether, 1:5 (v:v)).

NMR Spectroscopy:

^{1}**H NMR** (400 MHz, Chloroform-*d*, 298 K) δ 8.18 (dd, *J* = 5.4, 3.4 Hz, 2H), 7.88 (dd, *J* = 5.9, 3.3 Hz, 1H), 7.59 - 7.45 (m, 4H), 7.34 - 7.17 (m, 3H), 7.11 - 7.05 (m, 2H), 3.73 - 3.62 (m, 1H),

2.60 - 2.24 (m, 4H).

^{13}C NMR (101 MHz, Chloroform-*d*, 298 K) δ 141.73, 140.58, 138.82, 136.32, 136.04, 129.38, 128.63, 128.52, 127.20, 126.52 (q, *J* = 281.2 Hz), 126.38, 125.57, 125.29, 124.79, 122.84, 121.92, 121.60, 48.35 (q, J = 27.2 Hz), 32.69, 30.70.

^{19}F NMR (376 MHz, Chloroform-*d*, 298 K) δ -69.58 (d, *J* = 9.3 Hz).

GC-MS: 370.

HRMS (ESI) m/z $[M+H]^+$: Calculated for $C_{22}H_{18}F_3S^+$: 371.1076. Found: 371.1071.

HPLC analysis: The enantiomeric ratio was determined on a Daicel Chiralpak IA Column, *n*-Hexane: *i*-PrOH = 94: 6, 1.0 mL/min, T = 25 ℃, λ = 254 nm. Retention time t_1 = 5.162 min (major), t_2 = 9.189 min (minor), er = 91:9.

$[\alpha]_D^{20}$ = -51.7° (c = 0.2, $CHCl_3$)

Melt point: 75.0 - 75.9℃.

(R)-2-fluoro-5-(1,1,1-trifluoro-4-phenylbutan-2-yl)pyridine (**3o**)

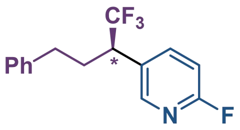

The product was obtained as a colorless oil (40.1 mg, 71 % yield).

R_f = 0.65 (ethyl acetate: petroleum ether, 1:2 (v:v)).

NMR Spectroscopy:

^{1}H NMR (400 MHz, Chloroform-*d*, 298 K) δ 8.10 (s, 1H), 7.74 (t, *J* = 8.1 Hz, 1H), 7.33 - 7.18 (m, 3H), 7.06 (dd, *J* = 7.8, 2.6 Hz, 2H), 7.02 - 6.95 (m, 1H), 3.33 - 3.19 (m, 1H), 2.68 - 2.56 (m, 1H), 2.47 - 2.35 (m, 2H), 2.26 - 2.10 (m, 1H).

^{13}C NMR (101 MHz, Chloroform-*d*, 298 K) δ 163.68 (d, *J* = 240.9 Hz), 148.79 (d, *J* = 14.9 Hz), 141.20 (d, *J* = 8.0 Hz), 139.72, 128.82, 128.40, 128.08, 126.67, 126.45 (q, *J* = 281.0 Hz), 110.06 (d, *J* = 37.6 Hz), 46.01 (q, *J* = 27.4 Hz), 32.33, 29.75.

^{19}F NMR (376 MHz, Chloroform-*d*, 298 K) δ -68.08, -69.84 (d, *J* = 9.8 Hz).

GC-MS: 283.

HRMS (ESI) m/z $[M+H]^+$: Calculated for $C_{15}H_{14}F_4N^+$: 284.1057. Found: 284.1069.

HPLC analysis: The enantiomeric ratio was determined on a Daicel Chiralpak OD-H Column, *n*-Hexane: *i*-PrOH = 98: 2, 1.0 mL/min, T = 25 ℃, λ = 254 nm. Retention time t_1 = 9.714 min (major), t_2 = 16.948 min (minor), er = 94:6.

$[\alpha]_D^{20}$ = -59.3° (c = 0.2, $CHCl_3$)

(R)-9,9-dimethyl-3-(1,1,1-trifluoro-4-phenylbutan-2-yl)-9H-fluorene (**3p**)

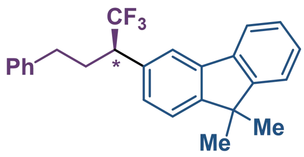

The product was obtained as a white solid (56.3 mg, 74 % yield).

R_f = 0.55 (ethyl acetate: petroleum ether, 1:20 (v:v)).

NMR Spectroscopy:

^{1}**H NMR** (400 MHz, Chloroform-*d*, 298 K) δ 7.74 (dd, *J* = 7.5, 2.4 Hz, 2H), 7.49 - 7.42 (m, 1H), 7.38 - 7.20 (m, 7H), 7.10 (d, *J* = 6.9 Hz, 2H), 3.37 - 3.19 (m, 1H), 2.70 - 2.59 (m, 1H), 2.49 - 2.36 (m, 2H), 2.35 - 2.21 (m, 1H), 1.52 (s, 6H).

^{13}**C NMR** (101 MHz, Chloroform-*d*, 298 K) δ 154.24, 153.87, 140.72, 139.49, 138.76, 133.44, 128.61, 128.59, 128.01, 127.59, 127.16, 126.52 (q, *J* = 280.5 Hz), 126.35, 123.69, 122.75, 120.26, 120.21, 49.37 (q, *J* = 26.5 Hz), 46.97, 32.60, 30.35, 27.21.

^{19}**F NMR** (376 MHz, Chloroform-*d*, 298 K) δ -69.43 (d, *J* = 9.3 Hz).

GC-MS: 380.

HRMS (ESI) m/z $[M+H]^+$: Calculated for $C_{25}H_{24}F_3$: 381.1825. Found: 381.1824.

HPLC analysis: The enantiomeric ratio was determined on a Daicel Chiralpak OD-H Column, *n*-Hexane: *i*-PrOH = 99: 1, 1.0 mL/min, T = 25 ℃, λ = 254 nm. Retention time t_1 = 4.761 min (major), t_2 = 5.874 min (minor), er = 93:7.

$[\alpha]_D^{20}$ = -49.5° (c = 0.2, $CHCl_3$)

Melt point: 60.1 - 61.4℃.

(8R,9S,13S,14S)-13-methyl-17-oxo-7,8,9,11,12,13,14,15,16,17-decahydro-6H-cyclopenta[a]phenanthren-2-yl 4-((R)-1,1,1-trifluoro-4-phenylbutan-2-yl)benzoate (**3q**)

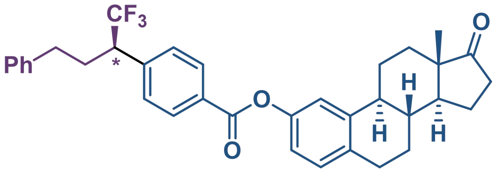

The product was obtained as a white solid (90.7 mg, 81 % yield).

R_f = 0.30 (ethyl acetate: petroleum ether, 1:10 (v:v)).

NMR Spectroscopy:

^{1}**H NMR** (400 MHz, Chloroform-*d*, 298 K) δ 8.23 (d, *J* = 8.4 Hz, 2H), 7.45 (d, *J* = 8.1 Hz, 2H), 7.35 (d, *J* = 8.5 Hz, 1H), 7.33 - 7.27 (m, 2H), 7.25 - 7.19 (m, 1H), 7.09 (d, *J* = 6.6 Hz, 2H), 7.04 - 6.94 (m, 2H), 3.41 - 3.26 (m, 1H), 2.98 - 2.92 (m, 2H), 2.65 - 2.37 (m, 5H), 2.34 - 1.94 (m, 6H), 1.72 - 1.40 (m, 6H), 0.93 (s, 3H).

^{13}**C NMR** (101 MHz, Chloroform-*d*, 298 K) δ 165.07, 148.90, 140.35, 140.22, 138.27, 137.70,

130.70, 129.87, 129.60, 128.73, 128.49, 126.68 (q, J = 281.0 Hz), 126.64, 126.54, 121.78, 118.94, 50.54, 49.25 (q, J = 26.9 Hz), 48.05, 44.29, 38.14, 35.96, 32.49, 31.69, 30.13, 29.55, 26.46, 25.90, 21.70, 13.94.

^{19}F NMR (376 MHz, Chloroform-*d*, 298 K) δ -69.15 (d, J = 9.2 Hz).

HRMS (ESI) m/z $[M+H]^+$: Calculated for $C_{35}H_{36}F_3O_3$: 561.2611. Found: 561.2609.

HPLC analysis: The enantiomeric ratio was determined on a Daicel Chiralpak AD-H Column, *n*-Hexane: *i*-PrOH = 85: 15, 1.0 mL/min, T = 25 ℃, λ = 254 nm. Retention time t_1 = 23.444 min (major), t_2 = 68.301 min (minor), er = 96:4.

$[\alpha]_D^{20}$ = -31.7° (c = 0.2, $CHCl_3$)

Melt point: 97.2 - 98.5℃.

Ethyl 5-((S)-2-(6-methoxynaphthalen-2-yl)propanamido)-2-((R)-1,1,1-trifluoro-4-phenylbutan-2-yl)benzoate (**3r**)

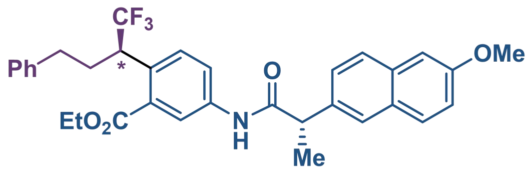

The product was obtained as a white solid (65.1 mg, 58 % yield).

R_f = 0.25 (ethyl acetate: petroleum ether, 1:1 (v:v)).

NMR Spectroscopy:

^{1}H NMR (400 MHz, Chloroform-*d*, 298 K) δ 11.26 (s, 1H), 8.82 (d, J = 8.7 Hz, 1H), 7.91 - 7.82 (m, 2H), 7.80 - 7.72 (m, 2H), 7.54 (d, J = 8.6 Hz, 1H), 7.46 (d, J = 9.4 Hz, 1H), 7.31 - 7.24 (m, 2H), 7.24 - 7.10 (m, 3H), 7.09 - 7.03 (m, 2H), 4.37 - 4.26 (m, 2H), 4.00 - 3.88 (m, 4H), 3.26 - 3.12 (m, 1H), 2.62 - 2.51 (m, 1H), 2.43 - 2.29 (m, 2H), 2.26 - 2.11 (m, 1H), 1.74 (d, J = 7.1 Hz, 3H), 1.36 (t, J = 6.4 Hz, 3H).

^{13}C NMR (101 MHz, Chloroform-*d*, 298 K) δ 173.51, 167.78, 157.80, 141.72, 140.37, 136.12, 134.92, 133.98, 131.73, 129.48, 129.25, 128.66, 128.51, 128.32, 127.60, 126.70 (q, J = 280.6 Hz), 126.43, 126.35, 126.31, 120.85, 119.12, 115.73, 105.71, 61.65, 55.38, 49.16, 48.47 (q, J = 27.0 Hz), 32.42, 29.92, 18.37, 14.24.

^{19}F NMR (376 MHz, Chloroform-*d*, 298 K) δ -69.72 (d, J = 8.9 Hz).

HRMS (ESI) m/z $[M+H]^+$: Calculated for $C_{33}H_{33}F_3NO_4$: 564.2356. Found: 564.2349.

HPLC analysis: The enantiomeric ratio was determined on a Daicel Chiralpak AD-H Column, *n*-Hexane: *i*-PrOH = 85: 15, 1.0 mL/min, T = 25 ℃, λ = 254 nm. Retention time t_1 = 6.669 min (major), t_2 = 7.654 min (minor), er = 97:3.

$[\alpha]_D^{20}$ = -20.5° (c = 0.2, $CHCl_3$).

Melt point: 100.0 - 102.5℃.

4-((R)-1,1,1-trifluoro-4-phenylbutan-2-yl)benzyl (4R)-4-((5S,8S,9S,10R,13R,14R)-5,10-dimethyl-3,7,12-trioxohexadecahydro-1H-cyclopenta[a]phenanthren-17-yl)pentanoate (**3s**)

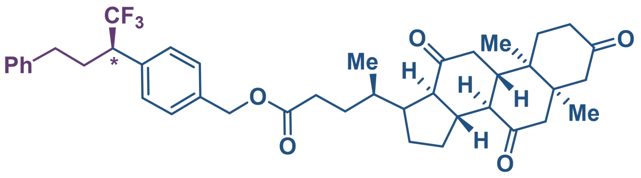

The product was obtained as a white solid (91.0 mg, 67 % yield).

R*f* = 0.30 (ethyl acetate: petroleum ether, 1:2 (v:v)).

NMR Spectroscopy:

^{1}H NMR (400 MHz, Chloroform-*d*, 298 K) δ 7.39 - 7.16 (m, 7H), 7.09 - 7.03 (m, 2H), 5.11 (s, 2H), 3.30 - 3.10 (m, 1H), 2.93 - 2.75 (m, 3H), 2.56 - 2.13 (m, 13H), 2.03 - 1.77 (m, 8H), 1.40 - 1.18 (m, 7H), 1.01 (s, 3H), 0.83 (d, *J* = 4.5 Hz, 3H).

^{13}C NMR (101 MHz, Chloroform-*d*, 298 K) δ 212.03, 209.16, 208.80, 173.92, 140.55, 136.31, 134.46, 129.55, 128.59, 128.24, 126.74 (q, *J* = 280.0 Hz), 126.32, 125.52, 65.67, 60.48, 56.96, 51.84, 49.06 (q, *J* = 26.8 Hz), 46.91, 45.73, 45.60, 45.00, 42.87, 38.70, 36.56, 36.08, 35.54, 35.33, 34.02, 32.54, 31.62, 30.51, 30.17, 27.71, 25.18, 21.94, 18.70, 11.86.

^{19}F NMR (376 MHz, Chloroform-*d*, 298 K) δ -69.49 (d, *J* = 8.8 Hz).

HRMS (ESI) m/z $[M+H]^+$: Calculated for $C_{41}H_{50}F_3O_5$: 679.3605. Found: 679.3600.

HPLC analysis: The enantiomeric ratio was determined on a Daicel Chiralpak OJ-H Column, *n*-Hexane: *i*-PrOH = 85: 15, 1.0 mL/min, T = 25 ℃, λ = 254 nm. Retention time t_1 = 13.878 min (major), t_2 = 15.777 min (minor), er = 93:7.

$[\alpha]_D^{20}$ = +14.8° (c = 0.2, $CHCl_3$).

Melt point: 91.4 - 92.7℃.

(R)-N-(4-(4-(4-(tert-butyl)phenyl)-1,1,1-trifluorobutan-2-yl)phenyl)acetamide (**3t**)

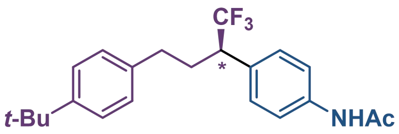

The product was obtained as a white solid (49.7 mg, 66 % yield).

R*f* = 0.20 (ethyl acetate: petroleum ether, 1:2 (v:v)).

NMR Spectroscopy:

^{1}H NMR (400 MHz, Chloroform-*d*, 298 K) δ 7.91 (s, 1H), 7.57 (d, *J* = 6.4 Hz, 2H), 7.31 (d, *J* = 8.6 Hz, 2H), 7.25 (d, *J* = 6.5 Hz, 2H), 7.03 (d, *J* = 8.6 Hz, 2H), 3.27 - 3.14 (m, 1H), 2.60 - 2.47 (m, 1H), 2.43 - 2.27 (m, 2H), 2.23 - 2.13 (m, 4H), 1.31 (s, 9H).

^{13}C NMR (101 MHz, Chloroform-*d*, 298 K) δ 168.94, 149.19, 138.11, 137.56, 130.33, 129.89,

128.14, 127.03 (q, J = 281.5 Hz), 125.52, 120.23, 48.73 (q, J = 26.7 Hz), 34.49, 32.00, 31.49, 30.13, 24.61.

^{19}F NMR (376 MHz, Chloroform-*d*, 298 K) δ -69.65 (d, J = 9.2 Hz).

GC-MS: 377.

HRMS (ESI) m/z $[M+H]^+$: Calculated for $C_{22}H_{27}F_3NO^+$: 378.2039. Found: 378.2040.

HPLC analysis: The enantiomeric ratio was determined on a Daicel Chiralpak OD-H Column, *n*-Hexane: *i*-PrOH = 85: 15, 1.0 mL/min, T = 25 ℃, λ = 254 nm. Retention time t_1 = 7.040 min (major), t_2 = 8.145 min (minor), er = 91:9.

$[\alpha]_D^{20}$ = -50.2° (c = 0.2, $CHCl_3$).

Melt point: 70.1 - 72.3℃.

(R)-N-(4-(1,1,1-trifluoro-4-(naphthalen-1-yl)butan-2-yl)phenyl)acetamide (**3u**)

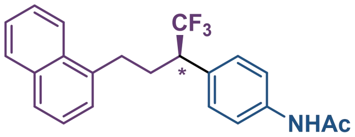

The product was obtained as a colorless oil (53.4 mg, 72 % yield).

R_f = 0.30 (ethyl acetate: petroleum ether, 1:1 (v:v)).

NMR Spectroscopy:

^{1}H NMR (400 MHz, Chloroform-*d*, 298 K) δ 8.01 - 7.54 (m, 12H), 3.39 - 3.20 (m, 1H), 3.07 - 2.94 (m, 1H), 2.92 - 2.83 (m, 1H), 2.51 - 2.39 (m, 1H), 2.35 - 2.24 (m, 1H), 2.16 (s, 3H).

^{13}C NMR (101 MHz, Chloroform-*d*, 298 K) δ 168.74, 138.36, 136.91, 136.69, 136.11, 132.17, 129.90, 128.99, 127.21, 126.23 (q, J = 278.3 Hz), 126.20, 126.11, 125.60, 124.37, 123.48, 120.22, 49.27 (q, J = 26.4 Hz), 29.96, 29.67, 24.62.

^{19}F NMR (376 MHz, Chloroform-*d*, 298 K) δ -69.60 (d, J = 9.2 Hz).

GC-MS: 371.

HRMS (ESI) m/z $[M+H]^+$: Calculated for $C_{22}H_{21}F_3NO^+$: 372.1570. Found: 372.1577.

HPLC analysis: The enantiomeric ratio was determined on a Daicel Chiralpak OD-H Column, *n*-Hexane: *i*-PrOH = 90:10, 1.0 mL/min, T = 25 ℃, λ = 254 nm. Retention time t_1 = 16.110 min (major), t_2 = 17.946 min (minor), er = 93:7.

$[\alpha]_D^{20}$ = -45.5° (c = 0.2, $CHCl_3$).

(R)-N-(4-(4-(2-bromo-5-methoxyphenyl)-1,1,1-trifluorobutan-2-yl)phenyl)acetamide (**3v**)

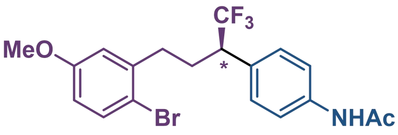

The product was obtained as a white solid (38.6 mg, 45 % yield).

R*f* = 0.25 (ethyl acetate: petroleum ether, 1:1 (v:v)).

NMR Spectroscopy:

^{1}H NMR (400 MHz, Chloroform-*d*, 298 K) δ 7.80 (s, 1H), 7.55 (d, *J* = 8.3 Hz, 2H), 7.45 - 7.33 (m, 1H), 7.27 (d, *J* = 8.1 Hz, 2H), 6.65 - 6.58 (m, 2H), 3.73 (s, 3H), 3.31 - 3.13 (m, 1H), 2.62 - 2.43 (m, 2H), 2.38 - 2.22 (m, 1H), 2.21 - 2.09 (m, 4H).

^{13}C NMR (101 MHz, Chloroform-*d*, 298 K) δ 168.84, 159.06, 141.16, 138.21, 133.57, 129.89, 129.61, 126.83 (q, *J* = 281.5 Hz), 120.14, 116.22, 114.76, 113.61, 55.50, 49.16 (q, *J* = 26.9 Hz), 33.66, 28.55, 24.60.

^{19}F NMR (376 MHz, Chloroform-*d*, 298 K) δ -69.78 (d, *J* = 9.0 Hz).

GC-MS: 429.

HRMS (ESI) m/z $[M+H]^+$: Calculated for $C_{19}H_{20}{}^{79}BrF_3NO_2^+$: 430.0624. Found: 430.0619.

HPLC analysis: The enantiomeric ratio was determined on a Daicel Chiralpak OD-H Column, *n*-Hexane: *i*-PrOH = 93: 7, 1.0 mL/min, T = 25 ℃, λ = 254 nm. Retention time t_1 = 10.880 min (major), t_2 = 12.751 min (minor), er = 87:13.

$[\alpha]_D^{20}$ = -53.0° (c = 0.2, $CHCl_3$).

Melt point: 74.3 - 75.0℃.

(R)-N-(4-(1,1,1-trifluoro-4-(4-(trifluoromethoxy)phenyl)butan-2-yl)phenyl)acetamide (**3w**)

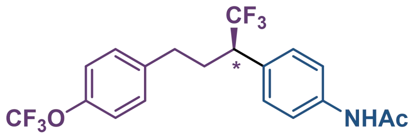

The product was obtained as a white solid (56.5 mg, 70 % yield).

R*f* = 0.25 (ethyl acetate: petroleum ether, 1:2 (v:v)).

NMR Spectroscopy:

^{1}H NMR (400 MHz, Chloroform-*d*, 298 K) δ 7.96 (s, 1H), 7.56 (d, *J* = 6.4 Hz, 2H), 7.21 (d, *J* = 8.0 Hz, 2H), 7.08 (q, *J* = 8.6 Hz, 4H), 3.22 - 3.09 (m, 1H), 2.59 - 2.48 (m, 1H), 2.46 - 2.35 (m, 1H), 2.33 - 2.23 (m, 1H), 2.22 - 2.09 (m, 4H).

^{13}C NMR (101 MHz, Chloroform-*d*, 298 K) δ 169.02, 147.76, 139.36, 138.24, 129.96, 129.76, 129.71, 126.86 (q, *J* = 280.1 Hz), 121.15, 120.59 (q, *J* = 257.8 Hz), 120.31, 48.70 (q, *J* = 26.6 Hz), 31.89, 30.02, 24.52.

^{19}F NMR (376 MHz, Chloroform-*d*, 298 K) δ -57.83, -69.78 (d, *J* = 9.1 Hz).

GC-MS: 405.

HRMS (ESI) m/z $[M+H]^+$: Calculated for $C_{19}H_{18}F_6NO_2^+$: 406.1236. Found: 406.1233.

HPLC analysis: The enantiomeric ratio was determined on a Daicel Chiralpak OD-H Column, *n*-

Hexane: *i*-PrOH = 93: 7, 1.0 mL/min, T = 25 ℃, λ = 254 nm. Retention time t_1 = 11.100 min (major), t_2 = 12.948 min (minor), er = 91:9.

$[\alpha]_D^{20}$ = -58.3° (c = 0.2, $CHCl_3$).

Melt point: 66.9 - 67.6℃.

(R)-N-(2-fluoro-4-(1,1,1-trifluoro-4-(4-(trifluoromethoxy)phenyl)butan-2-yl)phenyl)cyclohexanecarboxamide (**3x**)

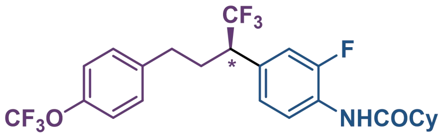

The product was obtained as a white solid (54.0 mg, 55 % yield).

R_f = 0.40 (ethyl acetate: petroleum ether, 1:1 (v:v)).

NMR Spectroscopy:

^{1}H NMR (400 MHz, Chloroform-*d*, 298 K) δ 8.39 (t, *J* = 8.2 Hz, 1H), 7.44 (s, 1H), 7.15 - 6.99 (m, 6H), 3.22 - 3.08 (m, 1H), 2.62 - 2.50 (m, 1H), 2.48 - 2.36 (m, 1H), 2.36 - 2.23 (m, 2H), 2.19 - 2.05 (m, 1H), 2.01 - 1.93 (m, 2H), 1.88 - 1.80 (m, 2H), 1.74 - 1.67 (m, 1H), 1.62 - 1.48 (m, 2H), 1.40 - 1.21 (m, 3H).

^{13}C NMR (101 MHz, Chloroform-*d*, 298 K) δ 174.58, 153.57, 151.15, 147.84, 139.08, 129.70 (d, *J* = 20.6 Hz), 126.83 (d, *J* = 10.0 Hz), 126.58 (q, *J* = 281.1 Hz), 125.72 (d, *J* = 17.9 Hz), 121.83 (d, *J* = 22.7 Hz), 121.20 (d, *J* = 21.7 Hz), 120.68 (d, *J* = 280.0 Hz), 115.12 (t, *J* = 19.7 Hz), 48.67 (q, *J* = 27.8 Hz), 46.62, 31.85, 30.02, 29.70, 25.68.

^{19}F NMR (376 MHz, Chloroform-*d*, 298 K) δ -57.85, -69.85 (d, *J* = 8.9 Hz), -130.50 (t, *J* = 9.3 Hz).

GC-MS: 491.

HRMS (ESI) m/z $[M+H]^+$: Calculated for $C_{24}H_{25}F_7NO_2^+$: 492.1768. Found: 492.1770.

HPLC analysis: The enantiomeric ratio was determined on a Daicel Chiralpak OD-H Column, *n*-Hexane: *i*-PrOH = 95: 5, 1.0 mL/min, T = 25 ℃, λ = 254 nm. Retention time t_1 = 5.131 min (minor), t_2 = 5.678 min (major), er = 92:8.

$[\alpha]_D^{20}$ = -40.6° (c = 0.2, $CHCl_3$).

Melt point: 68.2 - 69.9℃.

(R)-N-(4-(4-(4-(benzyloxy)phenyl)-1,1,1-trifluorobutan-2-yl)phenyl)acetamide (**3y**)

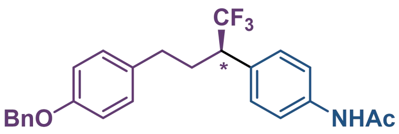

The product was obtained as a yellow oil (62.1 mg, 73 % yield).

R$_f$ = 0.30 (ethyl acetate: petroleum ether, 1:1 (v:v)).

NMR Spectroscopy:

^{1}**H NMR** (400 MHz, Chloroform-*d*, 298 K) δ 7.58 (s, 1H), 7.53 (d, *J* = 8.3 Hz, 2H), 7.45 - 7.36 (m, 4H), 7.35 - 7.30 (m, 1H), 7.25 - 7.16 (m, 3H), 6.82 (dd, *J* = 8.2, 2.6 Hz, 1H), 6.73 - 6.65 (m, 2H), 5.03 (s, 2H), 3.25 - 3.10 (m, 1H), 2.60 - 2.48 (m, 1H), 2.42 - 2.24 (m, 2H), 2.23 - 2.09 (m, 4H).

^{13}**C NMR** (101 MHz, Chloroform-*d*, 298 K) δ 168.69, 159.06, 142.32, 138.05, 137.11, 129.88, 129.64, 128.70, 128.09, 127.62, 126.95 (q, *J* = 281.0 Hz), 121.19, 120.15, 115.27, 112.53, 70.04, 48.61 (q, *J* = 26.6 Hz), 32.53, 29.99, 24.63.

^{19}**F NMR** (376 MHz, Chloroform-*d*, 298 K) δ -69.70 (d, *J* = 9.3 Hz).

GC-MS: 427.

HRMS (ESI) m/z [M+H]$^+$: Calculated for $C_{25}H_{25}F_3NO_2^+$: 428.1832. Found: 428.1829.

HPLC analysis: The enantiomeric ratio was determined on a Daicel Chiralpak AD-H Column, *n*-Hexane: *i*-PrOH = 90: 10, 1.0 mL/min, T = 25 ℃, λ = 254 nm. Retention time t_1 = 23.465 min (major), t_2 = 35.555 min (minor), er = 92:8.

[α]$_D^{20}$ = -59.0° (c = 0.2, $CHCl_3$).

(R)-N-(4-(1,1,1-trifluoro-4-(3-(trifluoromethyl)phenyl)butan-2-yl)phenyl)acetamide (**3z**)

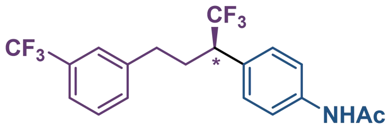

The product was obtained as a colorless oil (59.1 mg, 76 % yield).

R$_f$ = 0.20 (ethyl acetate: petroleum ether, 1:2 (v:v)).

NMR Spectroscopy:

^{1}**H NMR** (400 MHz, Chloroform-*d*, 298 K) δ 7.58 - 7.42 (m, 5H), 7.38 (t, *J* = 7.6 Hz, 2H), 7.31 - 7.19 (m, 8H), 3.22 - 3.07 (m, 1H), 2.66 - 2.55 (m, 1H), 2.53 - 2.41 (m, 1H), 2.37 - 2.26 (m, 1H), 2.23 - 2.13 (m, 1H).

^{13}**C NMR** (101 MHz, Chloroform-*d*, 298 K) δ 168.66, 141.52, 138.20, 131.86, 130.88 (q, J = 31.9 Hz), 129.81, 129.08, 126.77 (q, *J* = 282.0 Hz), 125.21 (q, *J* = 3.9 Hz), 124.21 (q, *J* = 280.6 Hz), 123.29 (q, *J* = 4.0 Hz), 120.21, 48.77 (q, *J* = 26.7 Hz), 32.44, 29.93, 24.67.

^{19}**F NMR** (376 MHz, Chloroform-*d*, 298 K) δ -62.49, -69.80 (d, *J* = 9.0 Hz).

GC-MS: 389.

HRMS (ESI) m/z [M+H]$^+$: Calculated for $C_{19}H_{18}F_6NO^+$: 390.1287. Found: 390.1287.

HPLC analysis: The enantiomeric ratio was determined on a Daicel Chiralpak OD-H Column, *n*-Hexane: *i*-PrOH = 90: 10, 1.0 mL/min, T = 25 ℃, λ = 254 nm. Retention time t_1 = 7.734 min

(major), t_2 = 8.946 min (minor), er = 92:8.

$[\alpha]_D^{20}$ = -44.5° (c = 0.2, $CHCl_3$).

(R)-N-(4-(4-([1,1'-biphenyl]-4-yl)-1,1,1-trifluorobutan-2-yl)phenyl)acetamide (**3aa**)

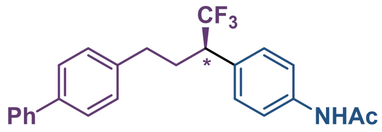

The product was obtained as a white solid (53.3 mg, 67 % yield).

R_f = 0.30 (ethyl acetate: petroleum ether, 1:1 (v:v)).

NMR Spectroscopy:

^{1}H NMR (400 MHz, Chloroform-*d*, 298 K) δ 7.63 - 7.47 (m, 7H), 7.46 - 7.39 (m, 2H), 7.37 - 7.29 (m, 1H), 7.29 - 7.23 (m, 2H), 7.18 - 7.12 (m, 2H), 3.29 - 3.15 (m, 1H), 2.67 - 2.54 (m, 1H), 2.50 - 2.31 (m, 2H), 2.26 - 2.15 (m, 4H).

^{13}C NMR (101 MHz, Chloroform-*d*, 298 K) δ 168.72, 140.99, 139.72, 139.33, 138.09, 130.22, 129.91, 128.89, 127.34, 127.09, 126.99 (q, *J* = 280.6 Hz), 120.20, 48.70 (q, *J* = 26.5 Hz), 32.17, 30.09, 24.64.

^{19}F NMR (376 MHz, Chloroform-*d*, 298 K) δ -69.66 (d, *J* = 9.6 Hz).

GC-MS: 397.

HRMS (ESI) m/z $[M+H]^+$: Calculated for $C_{24}H_{23}F_3NO^+$: 398.1726. Found: 398.1730.

HPLC analysis: The enantiomeric ratio was determined on a Daicel Chiralpak OD-H Column, *n*-Hexane: *i*-PrOH = 85: 15, 1.0 mL/min, T = 25 ℃, λ = 254 nm. Retention time t_1 = 11.586 min (minor), t_2 = 19.102 min (major), e.r = 90:10.

$[\alpha]_D^{20}$ = -50.0° (c = 0.2, $CHCl_3$).

Melt point: 76.4 - 78.0℃.

(R)-N-(4-(4-(4-acetylphenyl)-1,1,1-trifluorobutan-2-yl)phenyl)acetamide (**3ab**)

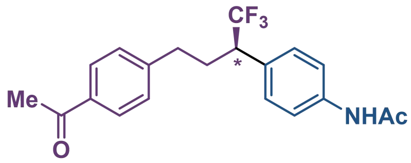

The product was obtained as a white solid (46.4 mg, 64 % yield).

R_f = 0.20 (ethyl acetate: petroleum ether, 1:1 (v:v)).

NMR Spectroscopy:

^{1}H NMR (400 MHz, Chloroform-*d*, 298 K) δ 7.86 (d, *J* = 6.0 Hz, 2H), 7.69 (s, 1H), 7.55 (d, *J* = 6.3 Hz, 2H), 7.21 (d, *J* = 6.3 Hz, 2H), 7.15 (d, *J* = 6.1 Hz, 2H), 3.21 – 3.06 (m, 1H), 2.66 - 2.53 (m, 4H), 2.51 - 2.39 (m, 1H), 2.37 - 2.26 (m, 1H), 2.23 - 2.12 (m, 4H).

^{13}C NMR (101 MHz, Chloroform-*d*, 298 K) δ 198.08, 168.74, 146.44, 138.26, 135.51, 129.79, 128.78, 128.73, 126.65 (q, *J* = 280.0 Hz), 120.21, 48.67 (q, *J* = 27.2 Hz), 32.55, 29.73, 26.68, 24.64.

^{19}F NMR (376 MHz, Chloroform-*d*, 298 K) δ -69.78 (d, *J* = 9.5 Hz).

GC-MS: 363.

HRMS (ESI) m/z $[M+H]^+$: Calculated for $C_{20}H_{21}F_3NO_2^+$: 364.1519. Found: 364.1520.

HPLC analysis: The enantiomeric ratio was determined on a Daicel Chiralpak OD-H Column, *n*-Hexane: *i*-PrOH = 85: 15, 1.0 mL/min, T = 25 ℃, λ = 254 nm. Retention time t_1 = 13.995 min (major), t_2 = 16.062 min (minor), er = 90:10.

$[\alpha]_D^{20}$ = -47.4° (c = 0.2, $CHCl_3$).

Melt point: 72.0 - 73.8℃.

4-((R)-4-(4-acetylphenyl)-1,1,1-trifluorobutan-2-yl)phenyl (3R,5R,7R)-adamantane-1-carboxylate (**3ac**)

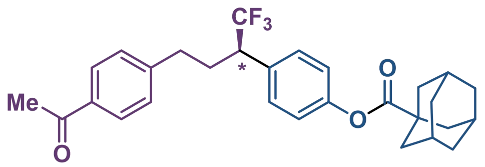

The product was obtained as a white solid (77.5 mg, 80 % yield).

R_f = 0.40 (ethyl acetate: petroleum ether, 1:10 (v:v)).

NMR Spectroscopy:

^{1}H NMR (400 MHz, Chloroform-*d*, 298 K) δ 7.87 (d, *J* = 8.4 Hz, 2H), 7.28 (d, *J* = 8.6 Hz, 2H), 7.16 (d, *J* = 8.3 Hz, 2H), 7.09 (d, *J* = 8.7 Hz, 2H), 3.26 - 3.11 (m, 1H), 2.69 - 2.59 (m, 4H), 2.54 - 2.42 (m, 1H), 2.41 - 2.29 (m, 1H), 2.25 - 2.14 (m, 1H), 2.11 - 2.03 (m, 10H), 1.84 - 1.70 (m, 5H).

^{13}C NMR (101 MHz, Chloroform-*d*, 298 K) δ 197.88, 176.13, 151.24, 146.26, 135.59, 130.11, 128.81, 128.73, 128.11, 126.39 (q, *J* = 280.2 Hz), 122.09, 48.70 (q, *J* = 26.8 Hz), 41.15, 38.85, 36.53, 32.54, 29.82, 27.96, 26.65.

^{19}F NMR (376 MHz, Chloroform-*d*, 298 K) δ -69.73 (d, *J* = 8.7 Hz).

GC-MS: 484.

HRMS (ESI) m/z $[M+H]^+$: Calculated for $C_{29}H_{32}F_3O_3^+$: 485.2298. Found: 485.2301.

HPLC analysis: The enantiomeric ratio was determined on a Daicel Chiralpak OD-H Column, *n*-Hexane: *i*-PrOH = 98: 2, 1.0 mL/min, T = 25 ℃, λ = 254 nm. Retention time t_1 = 7.006 min (major), t_2 = 12.563 min (minor), er = 94:6.

$[\alpha]_D^{20}$ = -46.5° (c = 0.2, $CHCl_3$).

Melt point: 64.1 - 65.5℃.

(1R,3R,4S)-4-isopropyl-3-methylcyclohexyl 2-(4-((R)-3-(4-acetamidophenyl)-4,4,4-trifluorobutyl)phenyl)acetate (**3ad**)

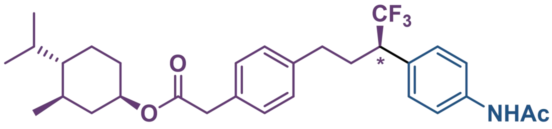

The product was obtained as a white solid (72.2 mg, 70 % yield).

R_f = 0.25 (ethyl acetate: petroleum ether, 1:1 (v:v)).

NMR Spectroscopy:

^{1}**H NMR** (400 MHz, Chloroform-*d*, 298 K) δ 7.60 (s, 1H), 7.55 - 7.48 (m, 2H), 7.23 - 7.13 (m, 4H), 7.03 - 6.97 (m, 2H), 4.65 (td, *J* = 10.9, 4.3 Hz, 1H), 3.55 (s, 2H), 3.22 - 3.07 (m, 1H), 2.59 - 2.49 (m, 1H), 2.42 - 2.22 (m, 2H), 2.20 - 2.10 (m, 4H), 1.95 (d, *J* = 12.5 Hz, 1H), 1.79 - 1.58 (m, 4H), 1.49 - 1.30 (m, 3H), 1.04 - 0.81 (m, 7H), 0.67 (d, *J* = 6.9 Hz, 3H).

^{13}**C NMR** (101 MHz, Chloroform-*d*, 298 K) δ 171.50, 168.60, 139.28, 138.08, 132.35, 130.08, 129.84, 129.40, 128.59, 127.72 (q, *J* = 280.6 Hz), 120.11, 74.86, 48.62 (q, *J* = 26.5 Hz), 47.08, 41.46, 40.89, 34.31, 32.11, 31.46, 30.05, 26.24, 24.62, 23.51, 22.09, 20.75, 16.35.

^{19}**F NMR** (376 MHz, Chloroform-*d*, 298 K) δ -69.76 (d, *J* = 9.8 Hz).

HRMS (ESI) m/z $[M+H]^+$: Calculated for $C_{30}H_{39}F_3NO_3^+$: 518.2877. Found: 518.2890.

HPLC analysis: The enantiomeric ratio was determined on a Daicel Chiralpak OD-H Column, *n*-Hexane: *i*-PrOH = 90: 10, 1.0 mL/min, T = 25 ℃, λ = 254 nm. Retention time t_1 = 9.703 min (major), t_2 = 12.111 min (minor), er = 92:8.

$[\alpha]_D^{20}$ = +8.5° (c = 0.2, $CHCl_3$).

Melt point: 80.0 - 81.7℃.

(R)-N-(4-(7-(2,5-dimethylphenoxy)-1,1,1-trifluoro-4,4-dimethylheptan-2-yl)phenyl)acetamide (**3ae**)

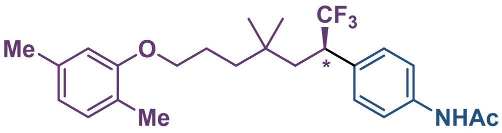

The product was obtained as a white solid (59.0 mg, 68 % yield).

R_f = 0.20 (ethyl acetate: petroleum ether, 1:2 (v:v)).

NMR Spectroscopy:

^{1}**H NMR** (400 MHz, Chloroform-*d*, 298 K) δ 7.51 - 7.45 (m, 3H), 7.30 - 7.25 (m, 2H), 7.04 - 6.97 (m, 1H), 6.69 - 6.63 (m, 1H), 6.58 (s, 1H), 3.81 - 3.70 (m, 2H), 3.37 - 3.25 (m, 1H), 2.31 (d, *J* = 3.8 Hz, 3H), 2.15 (dd, *J* = 17.2, 3.8 Hz, 6H), 1.93 (d, *J* = 5.1 Hz, 2H), 1.81 - 1.64 (m, 1H), 1.63 - 1.52 (m, 1H), 1.38 - 1.20 (m, 2H), 0.80 (s, 6H).

^{13}C NMR (101 MHz, Chloroform-*d*, 298 K) δ 168.60, 157.12, 137.85, 136.59, 132.48, 130.37, 130.03, 127.31 (q, *J* = 281.1 Hz), 123.63, 120.75, 119.96, 112.12, 68.35, 46.14 (q, *J* = 26.1 Hz), 39.86, 38.26, 33.17, 27.69, 27.52, 24.59, 24.16, 21.48, 15.89.

^{19}F NMR (376 MHz, Chloroform-*d*, 298 K) δ -70.21 (d, *J* = 9.9 Hz).

GC-MS: 435.

HRMS (ESI) m/z $[M+H]^+$: Calculated for $C_{25}H_{33}F_3NO_2^+$: 436.2458. Found: 436.2466.

HPLC analysis: The enantiomeric ratio was determined on a Daicel Chiralpak OJ-H Column, *n*-Hexane: *i*-PrOH = 90: 10, 1.0 mL/min, T = 25 ℃, λ = 254 nm. Retention time t_1 = 23.026 min (minor), t_2 = 27.623 min (major), er = 97:3.

$[\alpha]_D^{20}$ = -58.0° (c = 0.2, $CHCl_3$).

Melt point: 78.5 - 79.5℃.

(R)-2,5,7,8-tetramethyl-2-((4R,8R)-4,8,12-trimethyltridecyl)chroman-6-yl 2-(4-((R)-3-(4-acetamidophenyl)-4,4,4-trifluorobutyl)phenyl)acetate (**3af**)

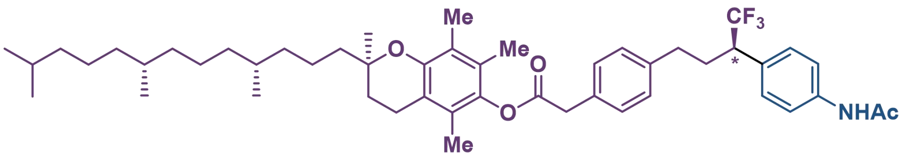

The product was obtained as a colorless oil (117.4 mg, 74 % yield).

R_f = 0.15 (ethyl acetate: petroleum ether, 1:1 (v:v)).

NMR Spectroscopy:

^{1}H NMR (400 MHz, Chloroform-*d*, 298 K) δ 7.81 (s, 1H), 7.50 (d, *J* = 5.7 Hz, 2H), 7.33 (d, *J* = 5.1 Hz, 2H), 7.20 (d, *J* = 5.9 Hz, 2H), 7.06 (d, *J* = 5.4 Hz, 2H), 3.86 (s, 2H), 3.24 - 3.04 (m, 1H), 2.62 - 2.49 (m, 3H), 2.43 - 2.27 (m, 2H), 2.24 - 2.14 (m, 1H), 2.09 (s, 3H), 2.06 (s, 3H), 1.91 (s, 3H), 1.85 (s, 3H), 1.81 - 1.68 (m, 2H), 1.58 - 1.48 (m, 3H), 1.44 - 1.00 (m, 23H), 0.91 - 0.83 (m, 10H).

^{13}C NMR (101 MHz, Chloroform-*d*, 298 K) δ 170.57, 168.86, 149.57, 140.55, 139.73, 138.23, 131.77, 129.93, 129.79, 128.86, 128.77, 126.98 (q, *J* = 280.6 Hz), 126.75, 124.99, 123.20, 120.19, 117.56, 75.18, 48.59 (q, *J* = 25.8 Hz), 41.08, 39.48, 37.54, 37.39, 32.90, 32.80, 32.12, 31.17, 30.03, 28.08, 24.91, 24.55, 24.43, 22.84, 22.74, 21.10, 20.65, 19.86, 19.75, 12.88, 12.00, 11.87.

^{19}F NMR (376 MHz, Chloroform-*d*, 298 K) δ -69.70 (d, *J* = 8.9 Hz).

HRMS (ESI) m/z $[M+H]^+$: Calculated for $C_{49}H_{69}F_3NO_4^+$: 792.5173. Found: 792.5170.

HPLC analysis: The enantiomeric ratio was determined on a Daicel Chiralpak OD-H Column, *n*-Hexane: *i*-PrOH = 95: 5, 1.0 mL/min, T = 25 ℃, λ = 254 nm. Retention time t_1 = 26.735 min (minor), t_2 = 30.206 min (major), er = 91:9.

[α]$_D^{20}$ = +18.7° (c = 0.2, $CHCl_3$).

(R)-(4-chlorophenyl)(2-methyl-3-(4,4,4-trifluoro-3-(p-tolyl)butyl)-1H-indol-1-yl)methanone (**3ag**)

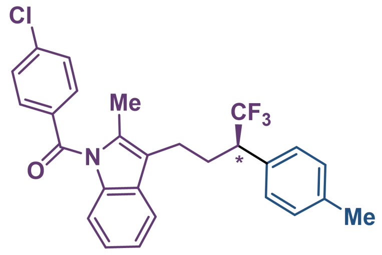

The product was obtained as a white solid (60.0 mg, 64 % yield).

R$_f$ = 0.40 (ethyl acetate: petroleum ether, 1:1 (v:v)).

NMR Spectroscopy:

^{1}**H NMR** (400 MHz, Chloroform-*d*, 298 K) δ 7.68 - 7.61 (m, 2H), 7.50 - 7.43 (m, 2H), 7.30 - 7.20 (m, 4H), 6.98 - 6.87 (m, 2H), 6.77 - 6.73 (m, 1H), 6.72 - 6.64 (m, 1H), 3.39 - 3.23 (m, 1H), 2.62 - 2.53 (m, 2H), 2.43 - 2.29 (m, 5H), 2.26 - 2.14 (m, 3H).

^{13}**C NMR** (101 MHz, Chloroform-*d*, 298 K) δ 168.34, 156.05, 139.17, 138.32, 134.42, 134.29, 131.42, 131.19, 131.11, 130.88, 129.64, 129.20, 127.06 (q, *J* = 280.0 Hz), 118.45, 115.18, 111.43, 101.20, 55.71, 49.33 (q, *J* = 26.7 Hz), 28.46, 21.23, 13.30.

^{19}**F NMR** (376 MHz, Chloroform-*d*, 298 K) δ -69.67 (d, *J* = 9.5 Hz).

HRMS (ESI) m/z $[M+H]^+$: Calculated for $C_{27}H_{24}{}^{35}ClF_3NO^+$: 470.1493. Found: 470.1481.

HPLC analysis: The enantiomeric ratio was determined on a Daicel Chiralpak IC Column, *n*-Hexane: *i*-PrOH = 96: 4, 1.0 mL/min, T = 25 ℃, λ = 254 nm. Retention time t_1 = 49.563 min (major), t_2 = 92.971 min (minor), er = 97:3.

[α]$_D^{20}$ = -41.4° (c = 0.2, $CHCl_3$).

Melt point: 89.0 - 91.0℃.

Direct decarboxylative C(sp^3)-C(sp^3) coupling.

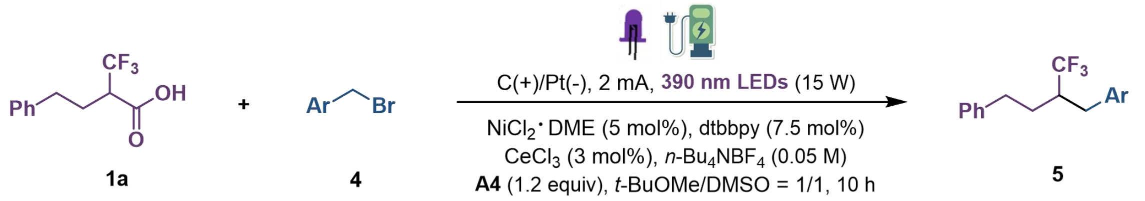

Scheme S3. General procedure for the decarboxylative C(sp^3)-C(sp^3) coupling of α-CF_3 carboxylic acids.

An oven-dried screw-cap vial was equipped with a stir bar, a threaded Teflon cap fitted with electrical feedthroughs, a graphite plate anode (2.5 cm * 0.5 cm), and a platinum plate cathode (2.5 cm * 0.5 cm). Under nitrogen atmosphere, *n*-Bu_4NBF_4 (66 mg, 0.10 mmol, 0.5 eq.), sodium phthalate (**A4**) (58 mg, 0.24 mmol, 1.2 eq.), α-CF_3 carboxylic acid (0.20 mmol, 1.0 eq.), benzyl bromide (0.24 mmol, 1.2 eq.), $CeCl_3$ (1.5 mg, 0.006 mmol, 3 mol%), $NiCl_2$ DME (2.2 mg, 0.01 mmol, 5 mol%), and dtbbpy (4.1 mg, 0.015 mmol, 7.5 mol%) were added to this reaction vessel. After that, the mixture of *t*-BuOMe and DMSO (1:1(v:v), 2.0 mL) were added via syringe. The reaction was irradiated with purple LEDs (15 W, 390 nm) under the vessel and electrolysis was initiated at a constant current of 2.0 mA, The temperature was kept at approximately 30°C through the use of a cooling fan. After 10 hours, the photolysis and electrolysis were terminated and the reaction was quenched with 10 mL water, then extracted with DCM (3 x 10 mL). The combined organic phase was dried with anhydrous Na_2SO_4 and concentrated under vacuum. The residue was further purified by column chromatography to afford corresponding coupling products, which was characterized by GC-MS analysis, ^{1}H NMR, ^{13}C NMR and ^{19}F NMR spectroscopy.

1-methoxy-4-(4-phenyl-2-(trifluoromethyl)butyl)benzene (**5a**)

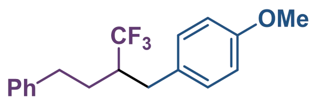

The product was obtained as a colorless oil (40.6 mg, 66 % yield).

R*f* = 0.65 (ethyl acetate: petroleum ether, 1:5 (v:v)).

NMR Spectroscopy:

^{1}H NMR (400 MHz, Chloroform-*d*, 298 K) δ 7.26 - 7.20 (m, 2H), 7.20 – 7.14 (m, 1H), 7.09 - 6.99 (m, 4H), 6.83 (d, *J* = 8.6 Hz, 2H), 3.80 (s, 3H), 3.00 (dd, *J* = 14.2, 4.6 Hz, 1H), 2.72 - 2.49 (m, 3H), 2.36 (dtq, *J* = 14.2, 9.4, 4.9, 4.3 Hz, 1H), 1.96 - 1.82 (m, 1H), 1.81 - 1.68 (m, 1H).

^{13}C NMR (101 MHz, Chloroform-*d*, 298 K) δ 158.42, 141.15, 130.15, 130.12, 128.51, 128.45 (q, *J* = 282.0 Hz), 128.43, 126.14, 114.07, 55.35, 44.01 (q, *J* = 24.6 Hz), 33.43, 33.07, 29.05.

^{19}F NMR (376 MHz, Chloroform-*d*, 298 K) δ -69.84 (d, *J* = 9.1 Hz).

GC-MS: 308

HRMS (ESI) m/z $[M+H]^+$: Calculated for $C_{18}H_{20}F_3O^+$: 309.1461. Found: 309.1455.

4-(4-phenyl-2-(trifluoromethyl)butyl)-1,1'-biphenyl (**5b**)

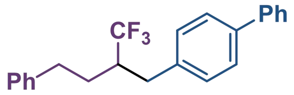

The product was obtained as a colorless oil (27.5 mg, 39 % yield).

R*f* = 0.60 (ethyl acetate: petroleum ether, 1:5 (v:v)).

NMR Spectroscopy:

^{1}**H NMR** (400 MHz, Chloroform-*d*, 298 K) δ 7.62 (d, *J* = 8.2 Hz, 2H), 7.55 (d, *J* = 8.0 Hz, 2H), 7.50 - 7.43 (m, 2H), 7.41 - 7.33 (m, 1H), 7.29 - 7.15 (m, 5H), 7.05 (d, *J* = 7.7 Hz, 2H), 3.12 (dd, *J* = 14.1, 4.3 Hz, 1H), 2.78 - 2.67 (m, 2H), 2.66 - 2.57 (m, 1H), 2.53 - 2.40 (m, 1H), 2.03 - 1.90 (m, 1H), 1.88 - 1.76 (m, 1H).

^{13}**C NMR** (101 MHz, Chloroform-*d*, 298 K) δ 141.04, 140.87, 139.67, 137.29, 129.61, 128.93, 128.57, 128.48, 128.46 (q, *J* = 281.5 Hz), 127.39, 127.13, 126.20, 43.81 (q, *J* = 24.5 Hz), 34.00, 33.07, 29.14.

^{19}**F NMR** (376 MHz, Chloroform-*d*, 298 K) δ -69.81 (d, *J* = 9.3 Hz).

GC-MS: 354

HRMS (ESI) m/z $[M+H]^+$: Calculated for $C_{23}H_{22}F_3^+$: 355.1668. Found: 355.1666.

(2-(trifluoromethyl)butane-1,4-diyl)dibenzene (**5c**)

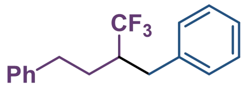

The product was obtained as a colorless oil (34.0 mg, 61 % yield).

R*f* = 0.55 (ethyl acetate: petroleum ether, 1:10 (v:v)).

NMR Spectroscopy:

^{1}**H NMR** (400 MHz, Chloroform-*d*, 298 K) δ 7.28 - 7.12 (m, 4H), 7.11 - 6.95 (m, 4H), 6.86 – 6.79 (m, 2H), 3.05 – 2.96 (m, 1H), 2.73 - 2.49 (m, 3H), 2.44 - 2.26 (m, 1H), 1.97 - 1.83 (m, 1H), 1.82 - 1.68 (m, 1H).

^{13}**C NMR** (101 MHz, Chloroform-*d*, 298 K) δ 156.73, 141.18, 130.14, 130.00, 128.51, 128.47 (q, *J* = 281.5 Hz), 128.44, 126.08, 116.26, 43.98 (q, *J* = 26.9 Hz), 33.47, 33.07, 29.04.

^{19}**F NMR** (376 MHz, Chloroform-*d*, 298 K) δ -69.88 (d, *J* = 9.5 Hz).

GC-MS: 278

HRMS (ESI) m/z $[M+H]^+$: Calculated for $C_{17}H_{18}F_3^+$: 279.1355. Found: 279.135.

The C(sp^3)-C(sp^3) coupling of unactivated alkyl halide.

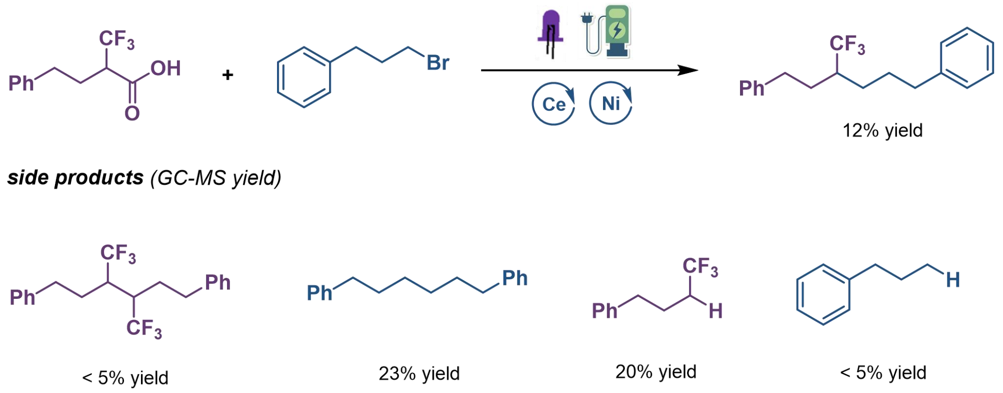

The reaction between C(sp^2) halides and alkyl radical precursors are typical strategies, which proceeded by Ni-catalyzed hybridization-dependent cross-coupling. However, the radical-radical cross coupling for the construction of C(sp^3)-C(sp^3) bonds remains an important yet elusive objective for coupling reactions, which mainly attributed to the potential competitive radical side reactions and the difficulty in regulating cross-selectivity caused by similar reactivity and addition rate to metal center of two radicals. In fact, we have also conducted the reaction of 1-bromo-3-phenylpropane with α-CF_3 carboxylic acids under standard conditions, but it exhibited poor cross selectivity. Applications of the photo-electrocatalytic LMCT protocol to chanllenging C(sp^3)-C(sp^3) coupling are ongoing in our laboratory.

Compatibility with cross-electrophile coupling.

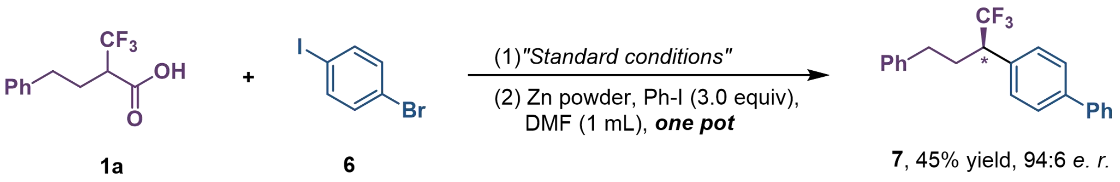

Scheme S4. Compatibility with cross-electrophile coupling.

An oven-dried screw-cap vial was equipped with a stir bar, a threaded Teflon cap fitted with electrical feedthroughs, a graphite plate anode (2.5 cm * 0.5 cm), and a platinum plate cathode (2.5 cm * 0.5 cm). Under nitrogen atmosphere, *n*-Bu_4NBF_4 (66 mg, 0.10 mmol, 0.5 eq.), sodium phthalate (**A4**) (58 mg, 0.24 mmol, 1.2 eq.), α-CF_3 carboxylic acid (0.20 mmol, 1.0 eq.), 4-bromoiodobenzene (**6**, 0.30 mmol, 1.5 eq.) and $CeCl_3$ (1.5 mg, 0.006 mmol, 3 mol%) were added to this reaction vessel. After that, the mixture of MeCN and DMSO (9:1(v:v), 2.0 mL) were added via syringe. The reaction was irradiated with purple LEDs (15 W, 390 nm) under the vessel and electrolysis was initiated at a constant current of 2.0 mA, The temperature was kept at approximately 30°C through the use of a cooling fan. After 10 hours, photolysis and electrolysis were terminated. Iodine benzene (3.0 equiv, 0.6 mmol), Zn powder (3.0 equiv, 0.6 mmol), and 1mL of dry DMF were added to the reaction flask, and stirred for an additional 12 hours. After this, the reaction was quenched with 10 mL water, then extracted with DCM (3 x 10 mL). The combined organic phase was dried with anhydrous Na_2SO_4 and concentrated under vacuum. The residue was further purified by column chromatography to afford corresponding coupling products, which was characterized by GC-MS analysis, ^{1}H NMR, ^{13}C NMR and ^{19}F NMR spectroscopy.

Large-scale synthesis.

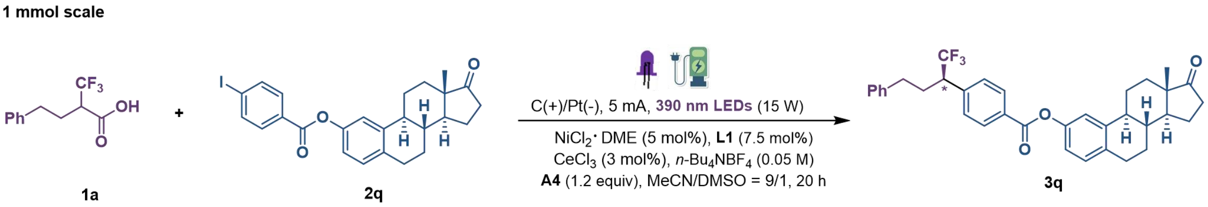

Scheme S5. Large-scale synthesis (1 mmol scale).

An oven-dried screw-cap vial was equipped with a stir bar, a threaded Teflon cap fitted with electrical feedthroughs, a graphite plate anode (2.5 cm * 0.5 cm), and a platinum plate cathode (2.5 cm * 0.5 cm). Under nitrogen atmosphere, *n*-Bu_4NBF_4 (330 mg, 0.50 mmol, 0.5 eq.), sodium phthalate (**A4**) (290 mg, 1.2 mmol, 1.2 eq.), α-CF_3 carboxylic acid **1a** (232 mg, 1.0 mmol, 1.0 eq.), aryl iodide **2q** (600 mg, 1.2 mmol, 1.2 eq.), $CeCl_3$ (7.5 mg, 0.03 mmol, 3 mol%), $NiCl_2$ DME (11.0

mg, 0.05 mmol, 5 mol%), and chiral ligand (**L1**) (17.0 mg, 0.075 mmol, 7.5 mol%) were added to this reaction vessel. After that, the mixture of MeCN and DMSO (9:1(v:v), 10 mL) were added via syringe. The reaction was irradiated with purple LEDs (15 W, 390 nm) under the vessel and electrolysis was initiated at a constant current of 5.0 mA, The temperature was kept at approximately 30°C through the use of a cooling fan. After 20 hours, the photolysis and electrolysis were terminated and the reaction was quenched with 20 mL water, then extracted with DCM (3 x 20 mL). The combined organic phase was dried with anhydrous Na_2SO_4 and concentrated under vacuum. The residue was further purified by column chromatography to afford corresponding coupling products.

(8R,9S,13S,14S)-13-methyl-17-oxo-7,8,9,11,12,13,14,15,16,17-decahydro-6H-cyclopenta[a]phenanthren-2-yl 4-((R)-1,1,1-trifluoro-4-phenylbutan-2-yl)benzoate (**3q**)

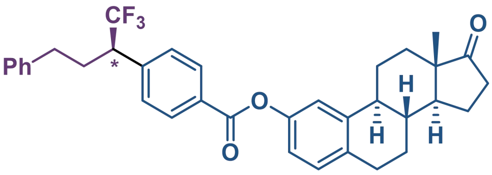

The product was obtained as a white solid (420.1 mg, 75 % yield).

HPLC analysis: The enantiomeric ratio was determined on a Daicel Chiralpak AD-H Column, *n*-Hexane: *i*-PrOH = 85: 15, 1.0 mL/min, T = 25 ℃, λ = 254 nm. Retention time t_1 = 23.464 min (major), t_2 = 68.430 min (minor), er = 96:4.

3 mmol scale

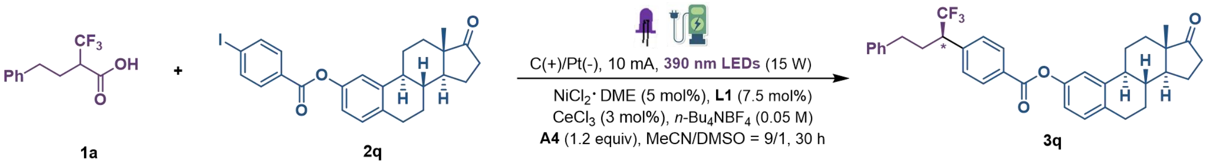

1a 2q 3q

Scheme S6. Large-scale synthesis (3 mmol scale).

An oven-dried screw-cap vial was equipped with a stir bar, a threaded Teflon cap fitted with electrical feedthroughs, a graphite plate anode (2.5 cm * 0.5 cm), and a platinum plate cathode (2.5 cm * 0.5 cm). Under nitrogen atmosphere, *n*-Bu_4NBF_4 (0.99 g, 1.5 mmol, 0.5 eq.), sodium phthalate (**A4**) (0.87 g, 3.6 mmol, 1.2 eq.), α-CF_3 carboxylic acid **1a** (0.87 g, 3.0 mmol, 1.0 eq.), aryl iodide **2q** (1.5 g, 3.6 mmol, 1.2 eq.), $CeCl_3$ (22.5 mg, 0.09 mmol, 3 mol%), $NiCl_2$ DME (33.0 mg, 0.15 mmol, 5 mol%), and chiral ligand (**L1**) (51.0 mg, 0.225 mmol, 7.5 mol%) were added to this reaction vessel. After that, the mixture of MeCN and DMSO (9:1(v:v), 15 mL) were added via syringe. The reaction was irradiated with purple LEDs (15 W, 390 nm) under the vessel and

electrolysis was initiated at a constant current of 10 mA, The temperature was kept at approximately 30°C through the use of a cooling fan. After 30 hours, the photolysis and electrolysis were terminated and the reaction was quenched with 30 mL water, then extracted with DCM (3 x 40 mL). The combined organic phase was dried with anhydrous Na_2SO_4 and concentrated under vacuum. The residue was further purified by column chromatography to afford corresponding coupling products.

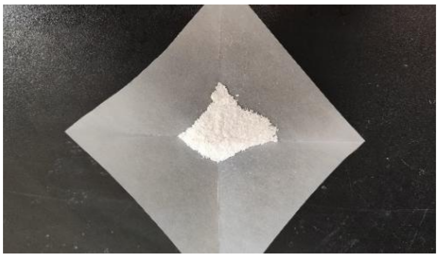

3q (1.04 g)

(8R,9S,13S,14S)-13-methyl-17-oxo-7,8,9,11,12,13,14,15,16,17-decahydro-6H-cyclopenta[a]phenanthren-2-yl 4-((R)-1,1,1-trifluoro-4-phenylbutan-2-yl)benzoate (**3q**)

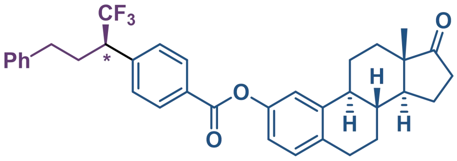

The product was obtained as a white solid (1.04 g, 62 % yield).

HPLC analysis: The enantiomeric ratio was determined on a Daicel Chiralpak AD-H Column, *n*-Hexane: *i*-PrOH = 85: 15, 1.0 mL/min, T = 25 ℃, λ = 254 nm. Retention time t_1 = 23.439 min (major), t_2 = 68.411 min (minor), er = 96:4.

Mechanistic Investigations

Radical-trapping experiments [3]

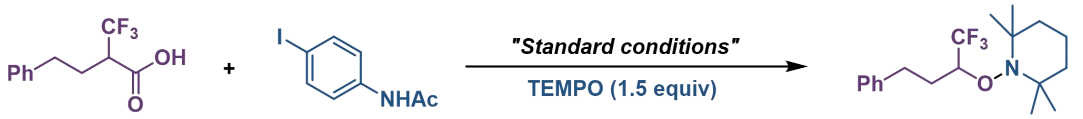

1a 2a 8, 28% ^{19}F NMR yield

Scheme S7. Radical trap reaction of TEMPO.

TEMPO (1.5 equiv) were subjected to the standard conditions. TEMPO-fluoroalkyl adduct **8** was detected by ^{1}H NMR, ^{19}F NMR spectroscopy and HRMS. 28% ^{19}F NMR yield (4-fluoro-iodobenzene as internal standard).

HRMS (ESI) m/z $[M+H]^+$: Calculated for $C_{19}H_{29}F_3NO^+$: 344.2196. Found: 344.2201.

^{1}H NMR (400 MHz, Chloroform-d, 298 K) 7.29 - 7.13 (m, 5H), 4.41 - 4.29 (m, 1H), 2.69 (dt, J = 12.7, 11.8, 2H), 2.05 - 1.89 (m, 2H), 1.83 - 1.74 (m, 2H), 1.68 - 1.46 (m, 4H), 1.21 (s, 12H).

^{19}F NMR (376 MHz, Chloroform-*d*, 298 K) δ -73.29 (d, *J* = 6.7 Hz, C*F*$_3$).

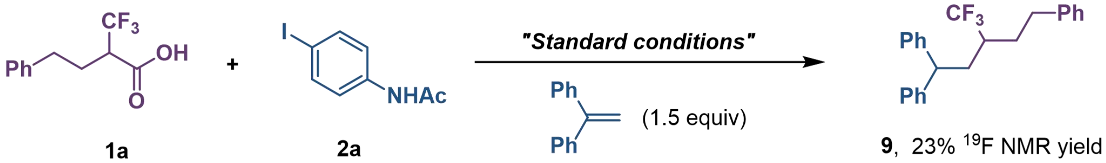

1a 2a 9, 23% ^{19}F NMR yield

Scheme S8. Radical trap reaction of 1,1-diphenylethylene.

1,1-Diphenylethylene (1.5 equiv) were subjected to the standard conditions. hydrofluoroalkylation product **9** was detected ^{1}H NMR, ^{19}F NMR spectroscopy and GC-MS. 23% ^{19}F NMR yield (4-fluoro-iodobenzene as internal standard).

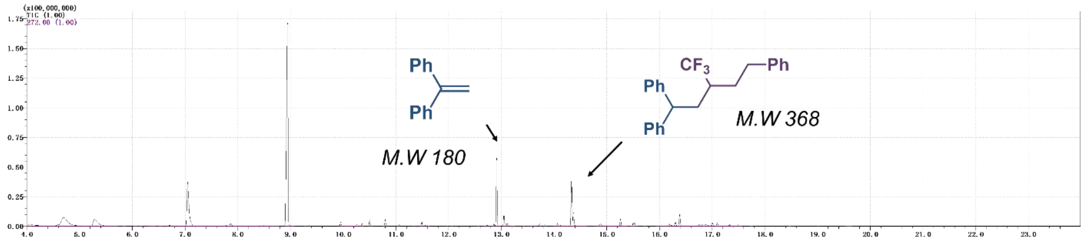

Figure S2. GC-MS spectra of radical trapping product **9**.

^{1}H NMR (400 MHz, Chloroform-d, 298 K) 7.34 - 7.16 (m, 15H), 3.93 (dt, *J* = 11.6, 10.5, Hz, 1H), 2.70 - 2.60 (m, 2H), 2.41 - 2.16 (m, 1H), 2.09 - 1.92 (m, 2H), 1.77 - 1.63 (m, 2H).

^{19}F NMR (376 MHz, Chloroform-*d*, 298 K) δ -71.02 (d, *J* = 8.2 Hz, C*F*$_3$).

Electricity and light on/off experiments [4]

An oven-dried screw-cap vial was equipped with a stir bar, a threaded Teflon cap fitted with electrical feedthroughs, a graphite plate anode (2.5 cm * 0.5 cm), and a platinum plate cathode (2.5 cm * 0.5 cm). Under nitrogen atmosphere, *n*-Bu_4NBF_4 (66 mg, 0.10 mmol, 0.5 eq.), sodium phthalate (**A4**) (58 mg, 0.24 mmol, 1.2 eq.), α-CF_3 carboxylic acid **1a** (0.20 mmol, 1.0 eq.), aryl iodide **2a** (0.24 mmol, 1.2 eq.), $CeCl_3$ (1.5 mg, 0.006 mmol, 3 mol%), $NiCl_2$ DME (2.2 mg, 0.01 mmol, 5 mol%), and chiral ligand (**L1**) (3.4 mg, 0.015 mmol, 7.5 mol%) were added to this reaction vessel. After that, the mixture of MeCN and DMSO (9:1(v:v), 2.0 mL) were added via syringe. Turn on the light and irradiate for 1 hour under constant 2 mA current, then the reaction mixture (0.1 mL) were taken with a syringe. The product yield was then measured by ^{19}F-NMR analysis with CF_3-DMA as an internal standard, after which the reaction mixture was kept in dark or without current for 1 hour and further analyzed by ^{19}F-NMR with CF_3-DMA as an internal standard. These on-off measurements were repeated, which showed that the product is formed only upon constant irradiation and electrolyzation and the reaction was not a chain reaction.

Time	1 h	2 h (electricity off)	3 h	4 h (light off)	5 h
Yield	13%	13%	22%	22%	28%

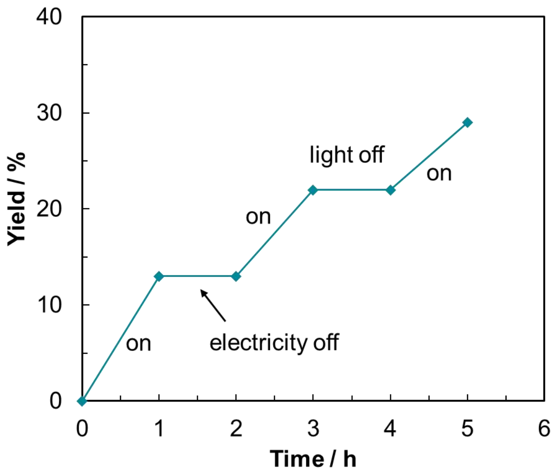

Figure S3. Electricity and light on/off experiments.

Catalytic experiments with cerium complex.

Preparation of cerium complex.

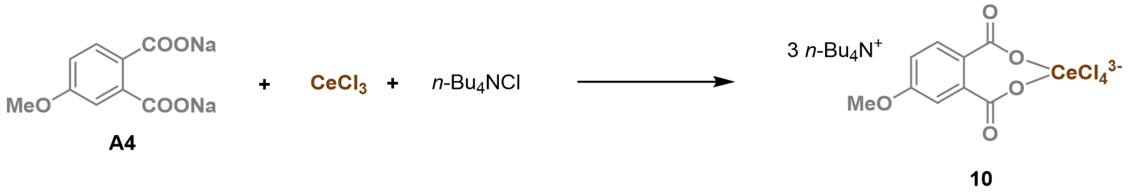

Scheme S9. Preparation of cerium complex.

To the solution of **A4** (72.0 mg, 0.30 mmol, 1.0 equiv.) in H_2O (5 mL), $CeCl_3$ (74.0 mg, 0.30 mmol, 1.0 equiv.) and *n*-Bu_4NCl (416.9 mg, 1.5 mmol, 5.0 equiv.) was added slowly. After stirring for 2 hours at room temperature, the resulting white solid was filtered over a sintered-glass funnel and washed with a small amount of cold H_2O (10-20 mL). The solid was dried for 6 h under high vacuum to give *n*-$Bu_4N)_3(ArCOO)_2$-$CeCl_4$ complex **10** as white solid in 51% yield, which was characterized by ^{1}H NMR and HRMS.

^{1}H NMR (400 MHz, DMSO-*d6*, 298 K) δ 7.70 (d, *J* = 8.5 Hz, 1H), 7.10 - 6.93 (m, 2H), 3.17 - 3.08 (m, 1H), 1.59 - 1.47 (m, 1H), 1.27 (h, *J* = 7.4 Hz, 1H), 0.90 (t, *J* = 7.3 Hz, 1H).

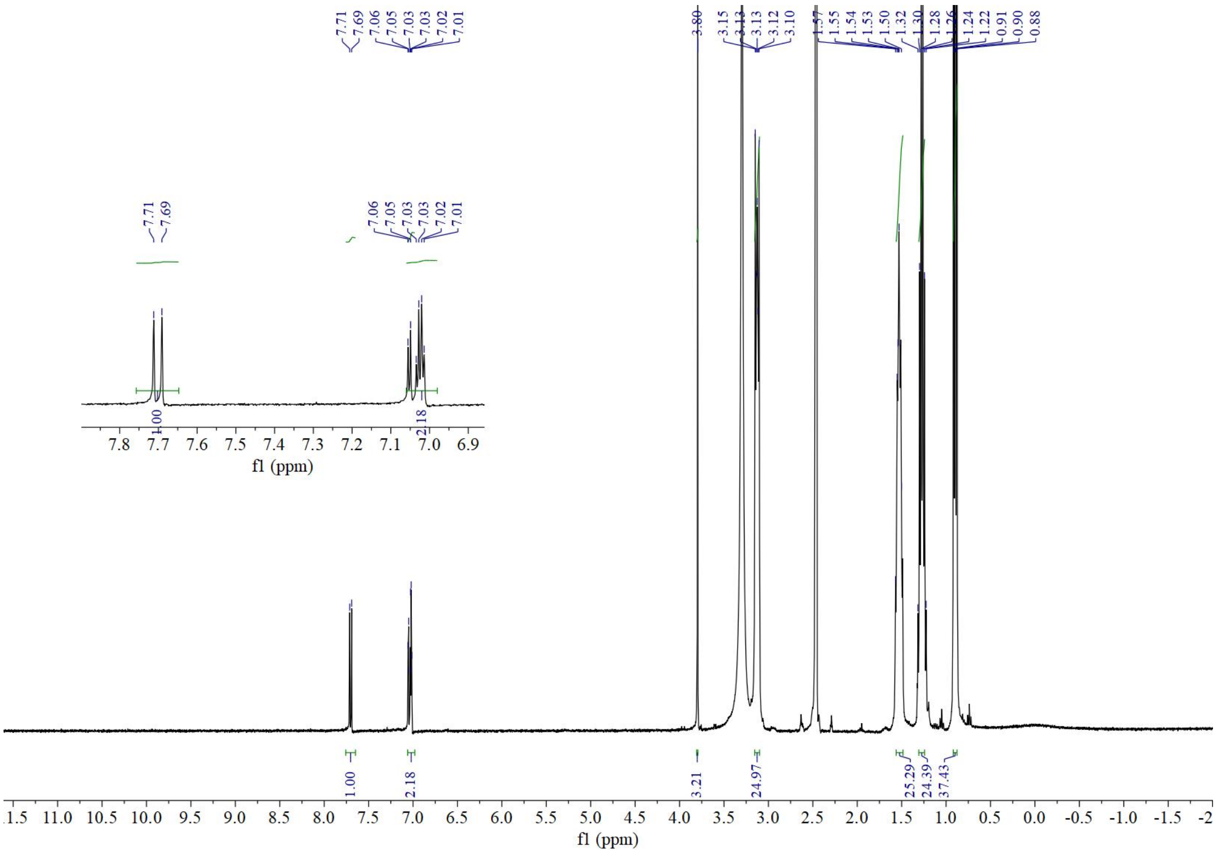

Figure S4. 1H NMR spectrum of *n*-$Bu_4N)_3(ArCOO)_2$-$CeCl_4$ complex **10**.

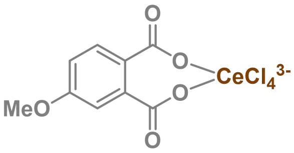

HRMS (ESI) m/z: Calculated for $C_9H_6CeCl_4O_5^{3-}$: 158.6004. Found: 158.6019.

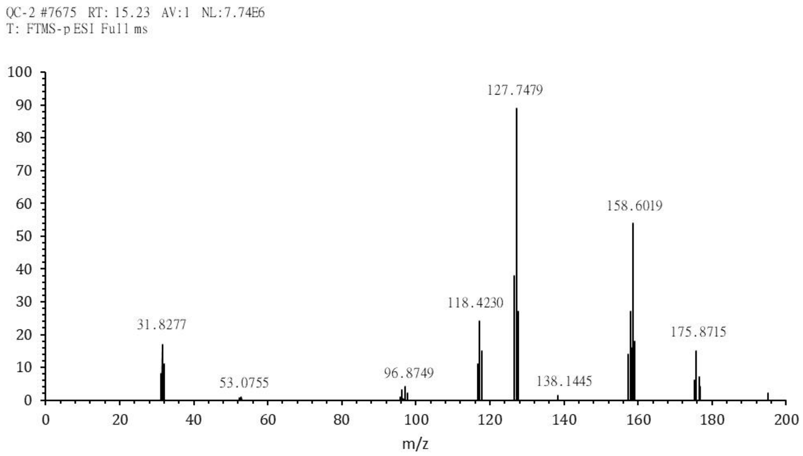

Figure S5. HRMS spectrum of *n*-$Bu_4N)_3(ArCOO)_2$-$CeCl_4$ complex **10**.

Catalytic experiments with cerium complex.

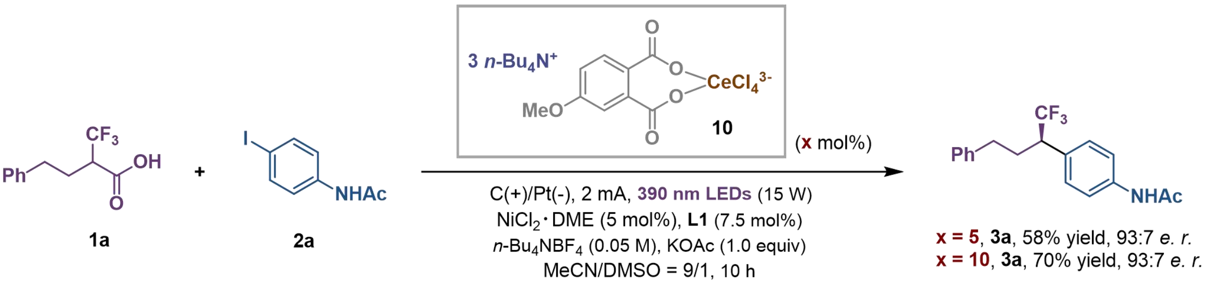

Scheme S10. Catalytic experiments with cerium complex.

An oven-dried screw-cap vial was equipped with a stir bar, a threaded Teflon cap fitted with electrical feedthroughs, a graphite plate anode (2.5 cm * 0.5 cm), and a platinum plate cathode (2.5 cm * 0.5 cm). Under nitrogen atmosphere, *n*-Bu_4NBF_4 (66 mg, 0.10 mmol, 0.5 eq.), cerium complex (**10**, 0.01 or 0.02 mmol, 5 or 10 mol%), α-CF_3 carboxylic acid (**1a**, 0.20 mmol, 1.0 eq.), aryl iodide (**2a**, 0.24 mmol, 1.2 eq.), $NiCl_2$ DME (2.2 mg, 0.01 mmol, 5 mol%), KOAc (0.2 mmol, 1.0 equiv.) and chiral ligand (**L1**) (3.4 mg, 0.015 mmol, 7.5 mol%) were added to this reaction vessel. After that, the mixture of MeCN and DMSO (9:1(v:v), 2.0 mL) were added via syringe. The reaction was irradiated with purple LEDs (15 W, 390 nm) under the vessel and electrolysis was initiated at a constant current of 2.0 mA, The temperature was kept at approximately 30°C through the use of a cooling fan. After 10 hours, the photolysis and electrolysis were terminated and the reaction was quenched with 10 mL water, then extracted with DCM (3 x 10 mL). The

combined organic phase was dried with anhydrous Na_2SO_4 and concentrated under vacuum. The residue was further purified by column chromatography to afford corresponding coupling products. The enantioselectivity was determined via HPLC analysis with chiral columns.

UV-visible absorption analysis.

UV-visible absorption spectra were recorded on an UV-2700 spectrophotometer, equipped with a temperature control unit at 25 °C. The samples were measured in Starna Fluorometer Microquartz cuvettes (volume: 1.8 ml, path length: 10 mm) equipped with a PTFE-stopper. The spectra were aquired from 300 to 800 nm using 1.0 nm steps. All measurements were performed in mixture of MeCN and DMSO (9:1) at the following concentrations: pure $CeCl_3$ (0.1 mM), pure A4 (0.1 mM), a mixture of $CeCl_3$ (0.1 mM) and pure A4 (0.1 mM).

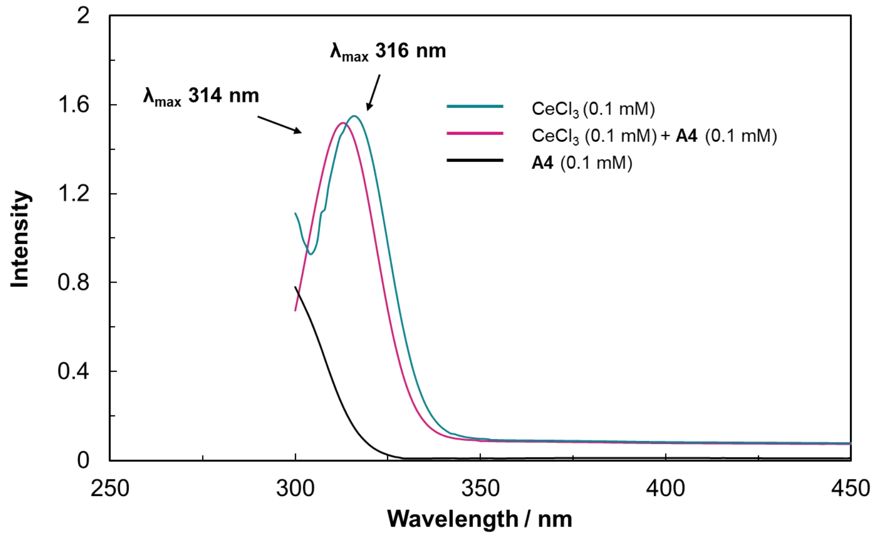

Figure S6. UV-visible absorption spectrums.

Cyclic voltammetry (CV) measurements [5-7]

General information: Cyclic voltammetry (CV) studies were performed in MeCN or MeCN/DMSO with *n*-Bu_4NBF_4 (0.1 M) as supporting electrolyte using a glassy carbon working electrode (2.0 mm in diameter), a SCE reference electrode, and a platinum plate counter electrode. The solution of determinand was sparged with nitrogen for 5 minutes before data collection and the scan rate was 100 mV/s.

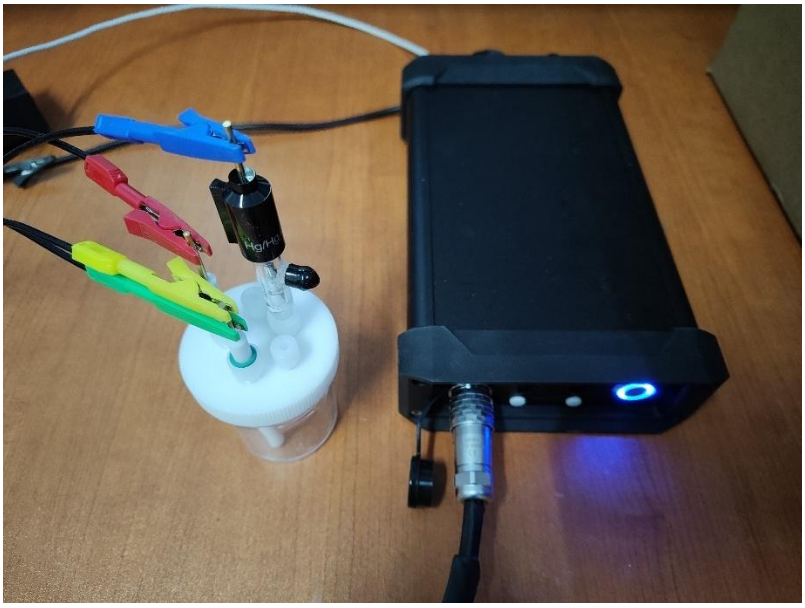

Figure S7. Cyclic voltammetry (CV) setup.

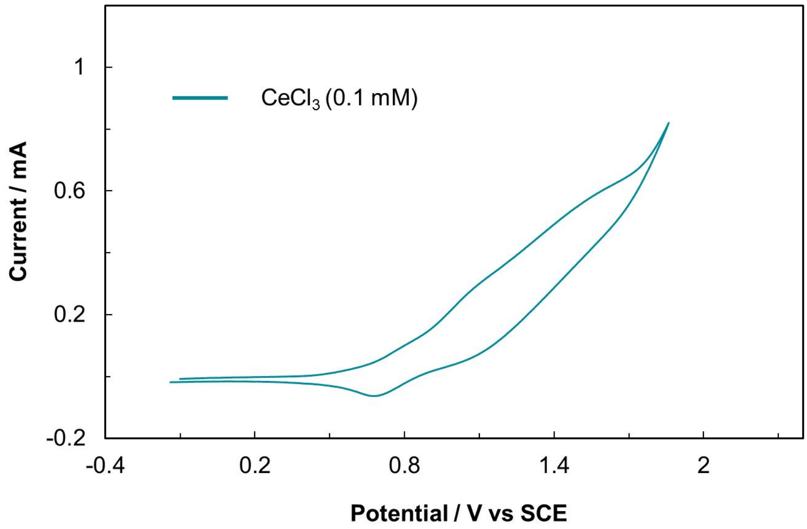

Figure S8. Cyclic voltammogram of $CeCl_3$ in DMSO/MeCN, Conditions: DMSO/MeCN (1:9, 10 mL), *n*-Bu_4BF_4 (0.10 M), $CeCl_3$ (0.1 mM).

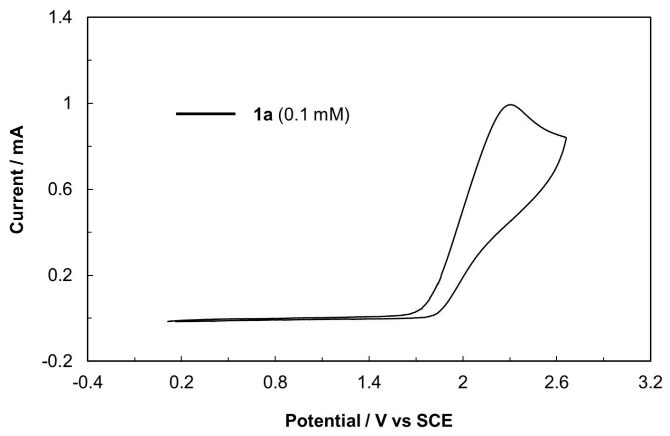

Figure S9. Cyclic voltammogram of **1a** in MeCN. Conditions: MeCN (10 mL), *n*-Bu_4BF_4 (0.10 M), **1a** (0.1 mM).

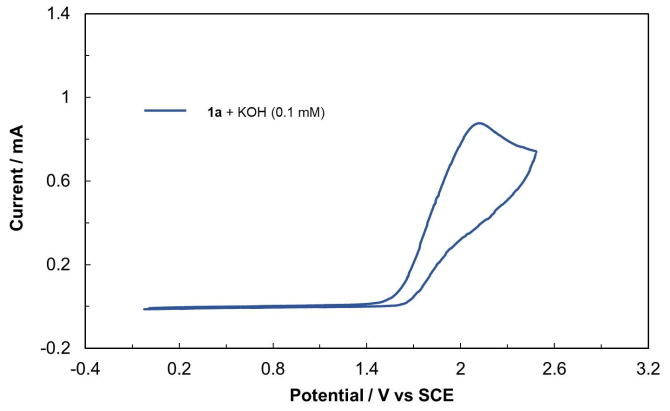

Figure S10. Cyclic voltammogram of **1a** in MeCN. Conditions: MeCN (10 mL), *n*-Bu_4BF_4 (0.10 M), **1a** (0.1 mM) + KOH (0.1 mM) + 4A MS (100 mg).

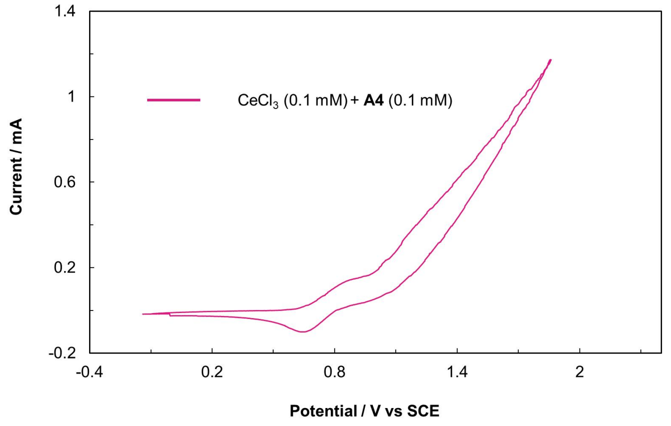

Figure S11. Cyclic voltammogram of **A4** and $CeCl_3$ in DMSO/MeCN. Conditions: DMSO/MeCN (1:9, 10 mL), *n*-Bu_4BF_4 (0.10 M), **A4** (0.1 mM) and $CeCl_3$ (0.1 mM).

Determination of abusolute configuration [8,9]

The product **3m** has been reported by Wang`s group [8], in whose work **3m** of *S*-configuration was synthesized. And we made the following comparation to determinate our product **3m** as *R*-configuration.

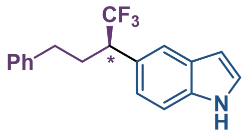

3m

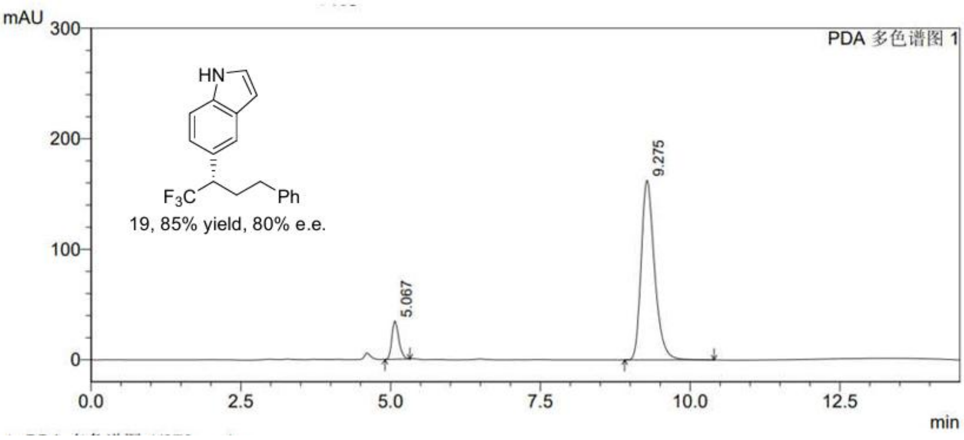

HPLC data of ***S*-3m** (90:10 er) in Wang`s work

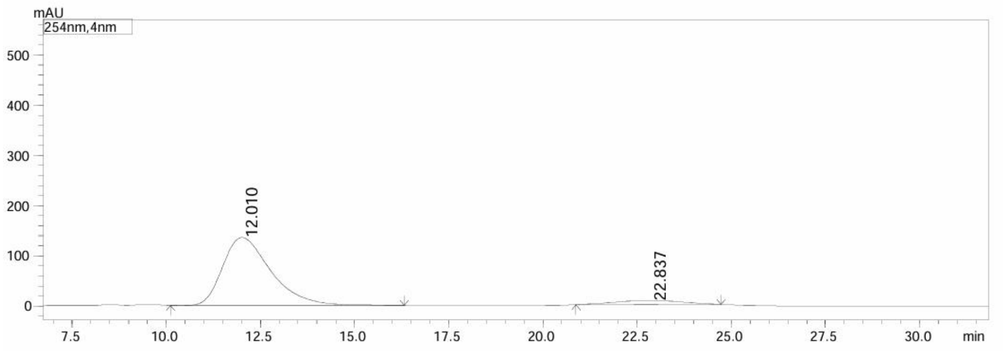

HPLC data of ***R*-3m** (92:8 er) in our work

HPLC Spectra

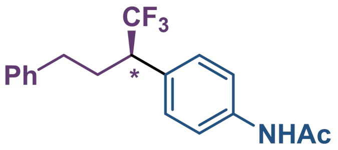

3a

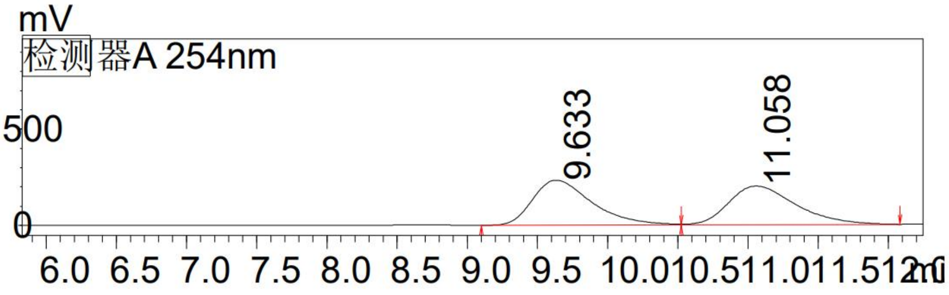

Peak#	Ret. Time	Area	Height	Area%	Height%
1	9.633	6865246	233594	50.693	53.762
2	11.058	6677522	200899	49.307	46.238
Total		13542767	434493	100.000	100.000

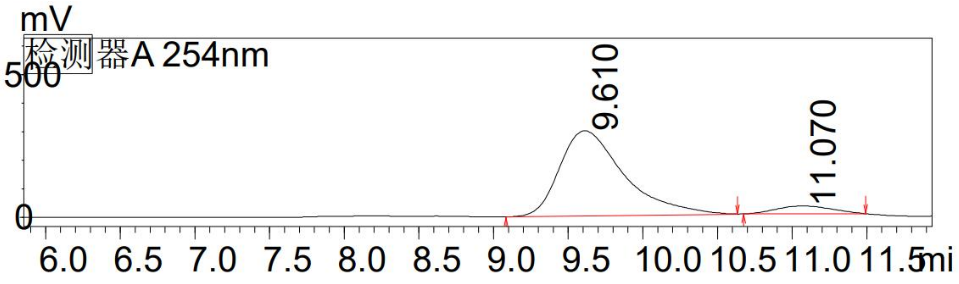

Peak#	Ret. Time	Area	Height	Area%	Height%
1	9.610	8938333	298116	92.614	91.557
2	11.070	712864	27492	7.386	8.443
Total		9651197	325608	100.000	100.000

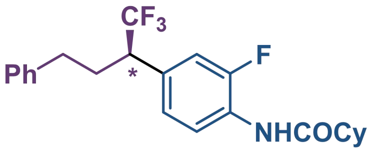

3b

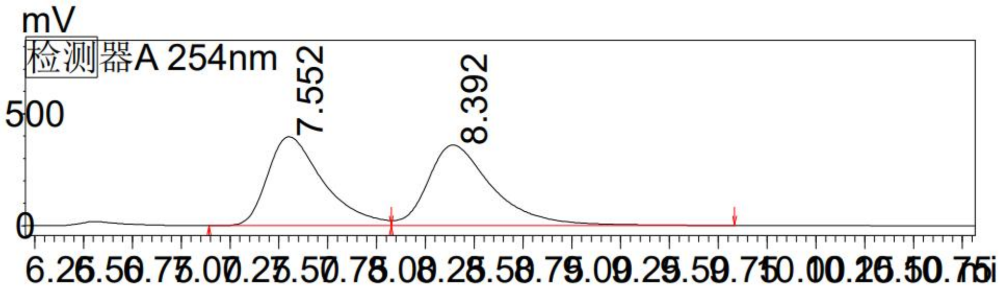

Peak#	Ret. Time	Area	Height	Area%	Height%
1	7.552	7862218	396816	48.765	52.467
2	8.392	8260582	359495	51.235	47.533
Total		16122800	756311	100.000	100.000

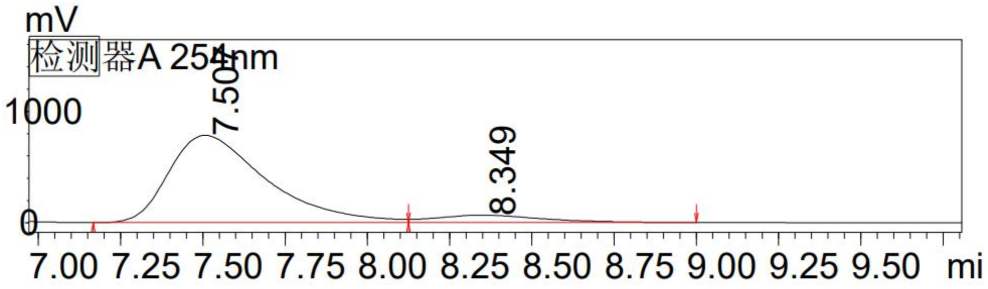

Peak#	Ret. Time	Area	Height	Area%	Height%
1	7.507	16081870	780806	91.783	92.265
2	8.349	1439669	65461	8.217	7.735
Total		17521539	846267	100.000	100.000

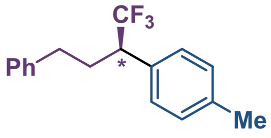

3c

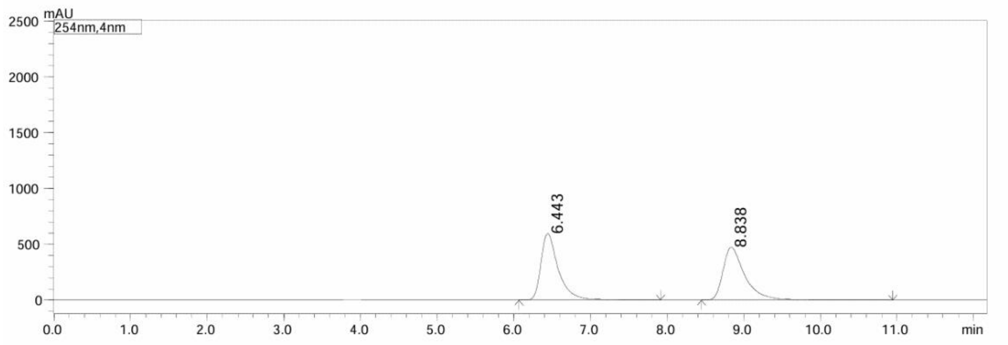

Peak#	Ret. Time	Area	Height	Area%	Height%
1	6.443	9423396	595380	50.086	55.738
2	8.838	9391172	472792	49.914	44.262
Total		18814568	1068172	100.000	100.000

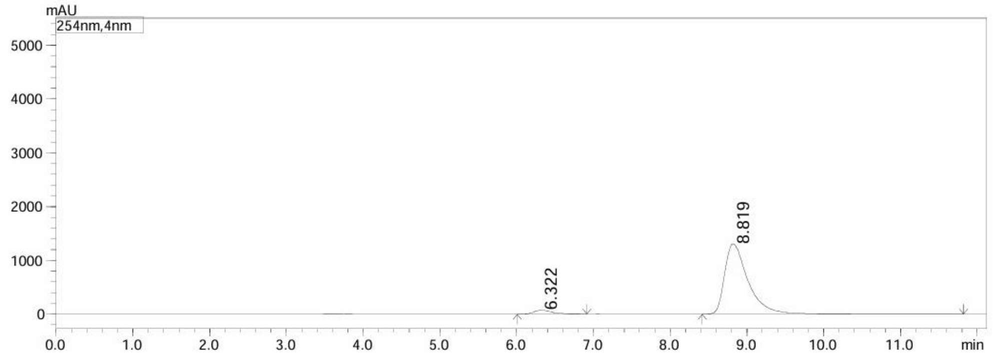

Peak#	Ret. Time	Area	Height	Area%	Height%
1	6.322	1207148	74511	4.160	5.385
2	8.819	27813321	1309056	95.840	94.615
Total		29020468	1383567	100.000	100.000

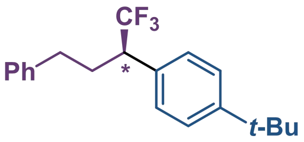

3d

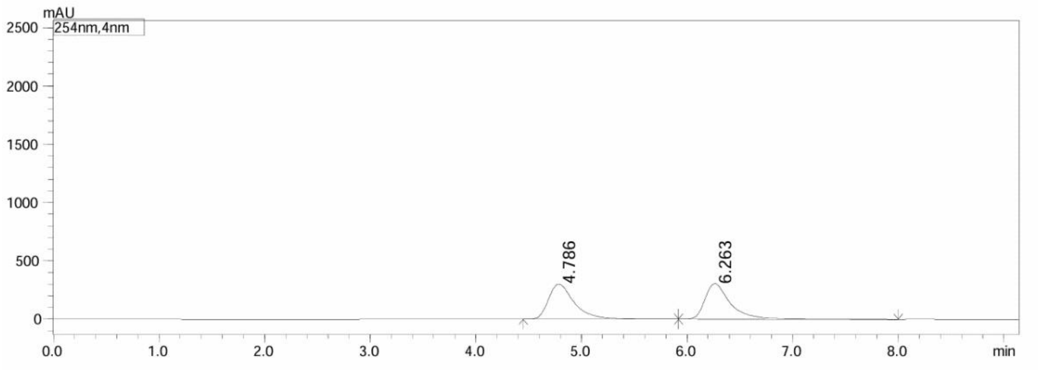

Peak#	Ret. Time	Area	Height	Area%	Height%
1	4.786	5108101	299746	50.047	49.551
2	6.263	5098554	305184	49.953	50.449
Total		10206655	604930	100.000	100.000

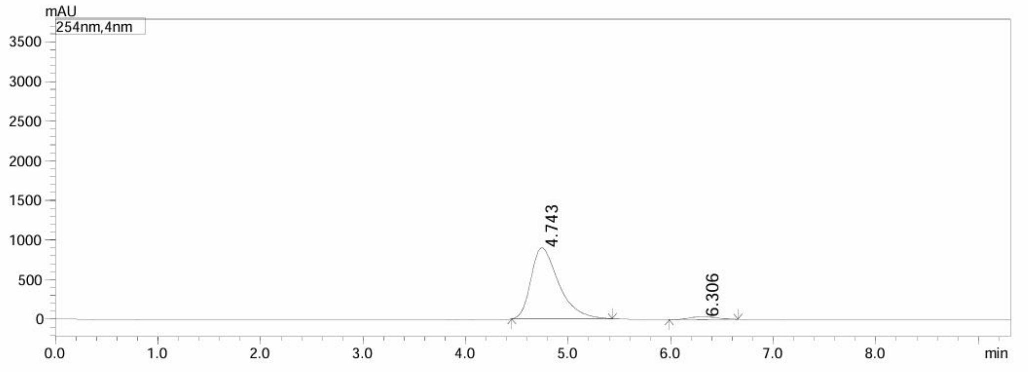

Peak#	Ret. Time	Area	Height	Area%	Height%
1	4.743	14813494	659440	92.308	91.173
2	6.306	1234458	63844	7.692	8.827
Total		16047952	723284	100.000	100.000

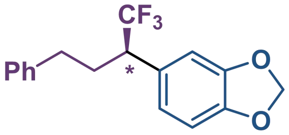

3e

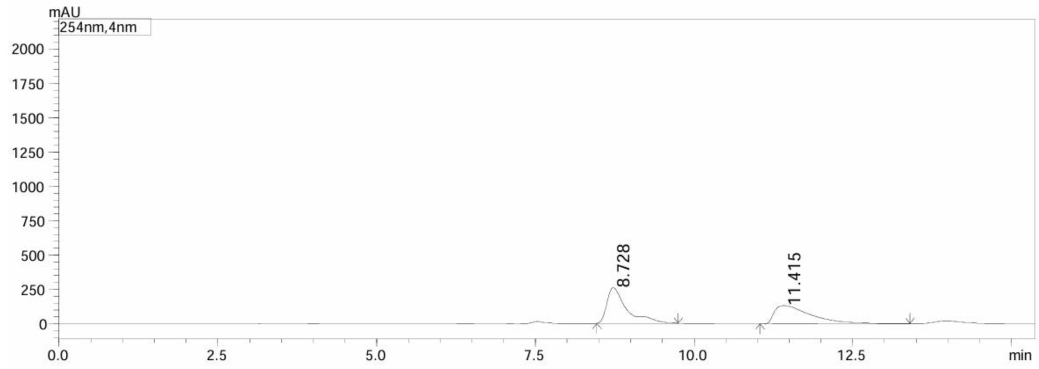

Peak#	Ret. Time	Area	Height	Area%	Height%
1	8.728	5641162	259830	51.730	66.247
2	11.415	5263889	132382	48.270	33.753
Total		10905051	392211	100.000	100.000

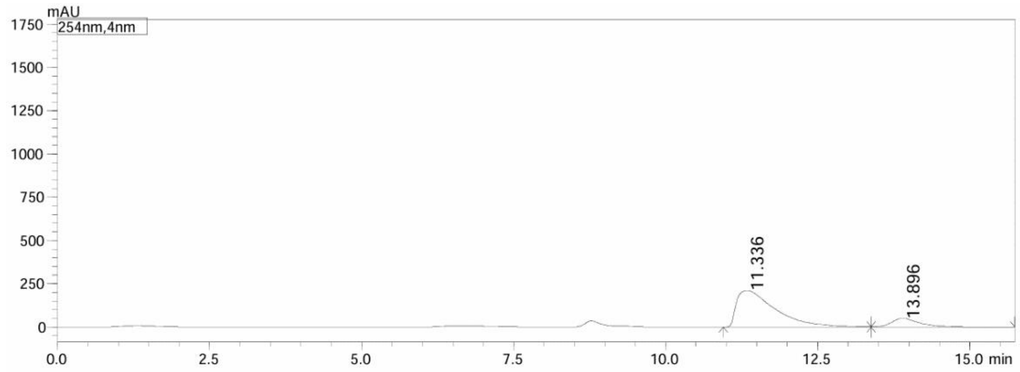

Peak#	Ret. Time	Area	Height	Area%	Height%
1	8.725	805873	28363	7.962	11.845
2	11.336	9315642	211085	92.038	88.155
Total		10121515	239448	100.000	100.000

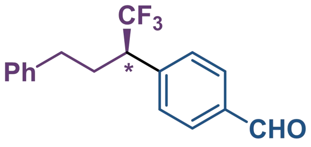

3f

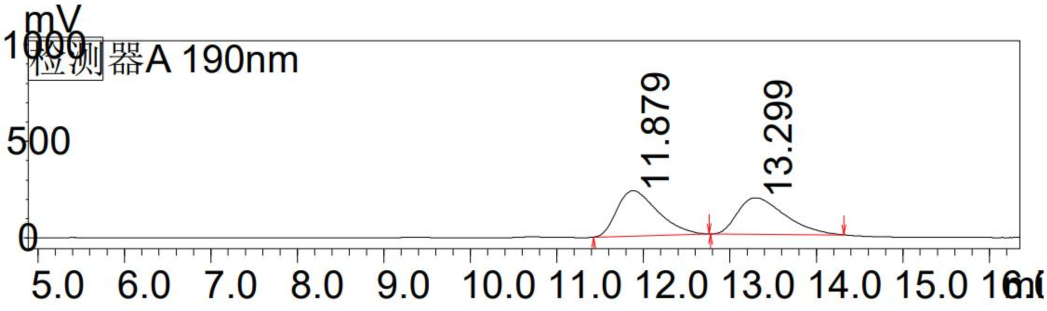

Peak#	Ret. Time	Area	Height	Area%	Height%
1	11.879	7729796	234973	51.606	55.305
2	13.299	7248637	189893	48.394	44.695
Total		14978433	424866	100.000	100.000

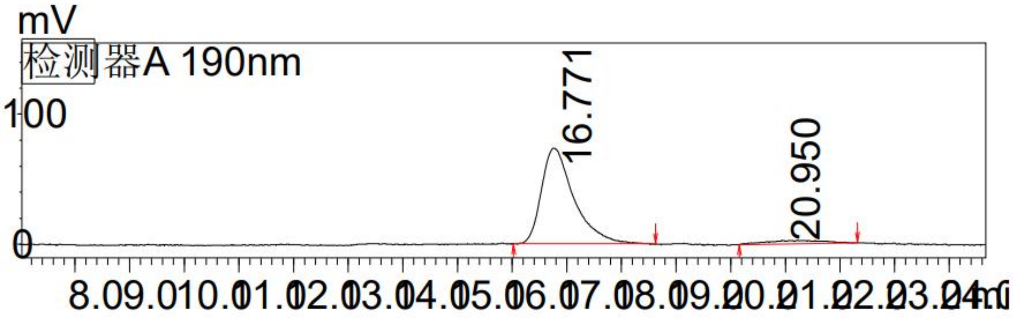

Peak#	Ret. Time	Area	Height	Area%	Height%
1	16.771	3040788	73458	94.310	96.465
2	20.950	183469	2692	5.690	3.535
Total		3224257	76149	100.000	100.000

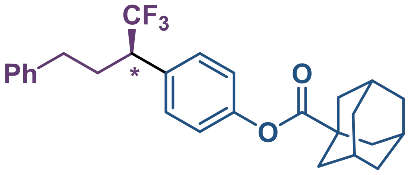

3g

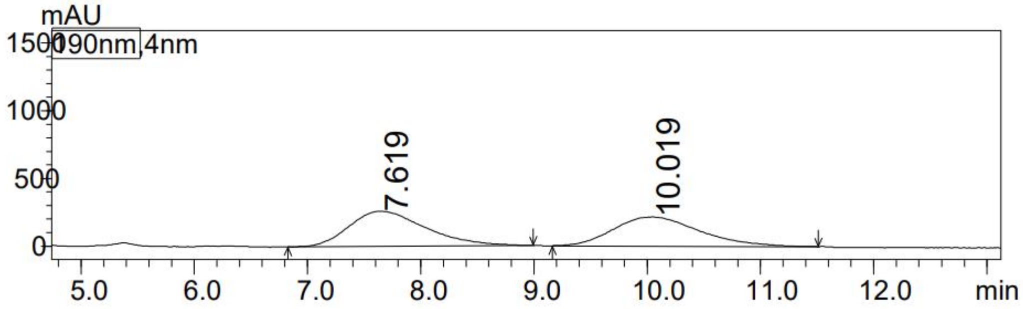

Peak#	Ret. Time	Area	Height	Area%	Height%
1	7.619	12336330	260117	51.886	54.781
2	10.019	11439501	214716	48.114	45.219
Total		23775831	474833	100.000	100.000

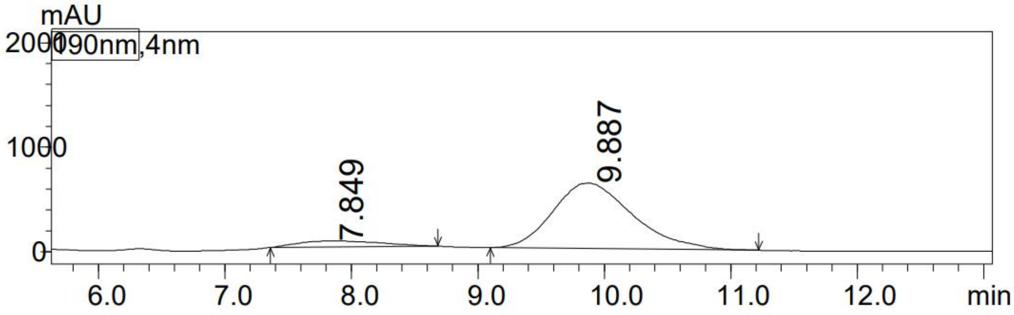

Peak#	Ret. Time	Area	Height	Area%	Height%
1	7.849	2656313	60168	8.817	8.746
2	9.887	27469731	627794	91.183	91.254
Total		30126044	687962	100.000	100.000

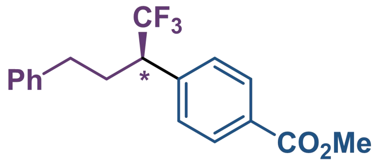

3h

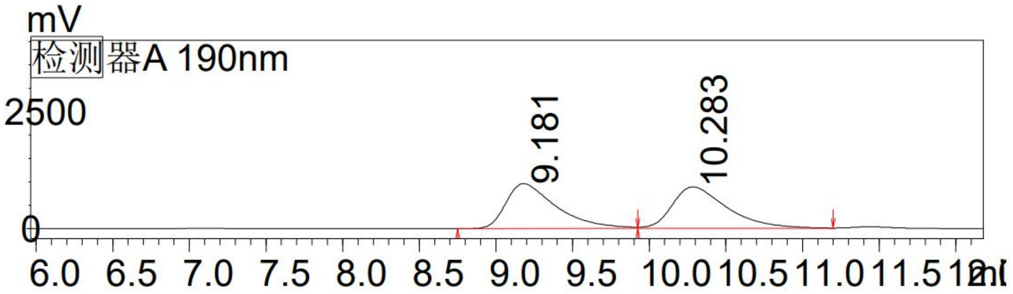

Peak#	Ret. Time	Area	Height	Area%	Height%
1	9.181	21910118	959007	50.010	51.983
2	10.283	21901324	885828	49.990	48.017
Total		43811441	1844834	100.000	100.000

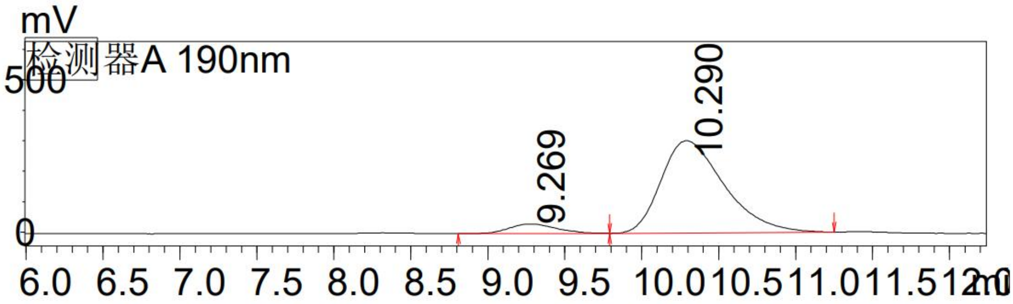

Peak#	Ret. Time	Area	Height	Area%	Height%
1	9.269	661551	31483	7.103	9.411
2	10.290	8651578	303042	92.897	90.589
Total		9313129	334525	100.000	100.000

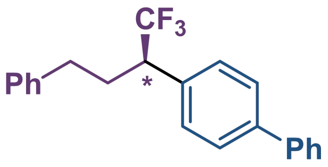

3i

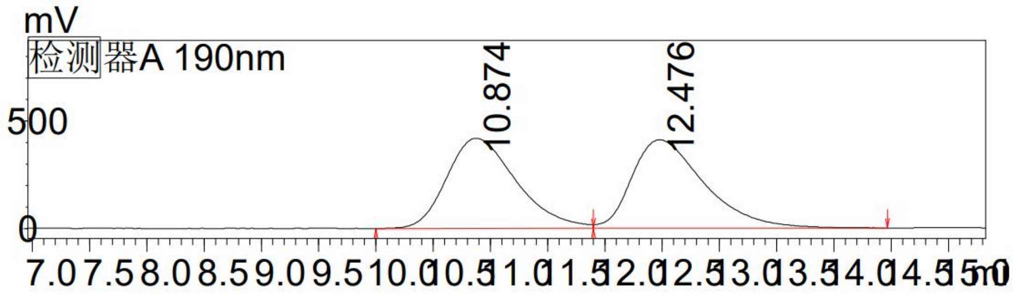

Peak#	Ret. Time	Area	Height	Area%	Height%
1	10.874	17637039	418243	49.384	50.458
2	12.476	18077036	410647	50.616	49.542
Total		35714075	828890	100.000	100.000

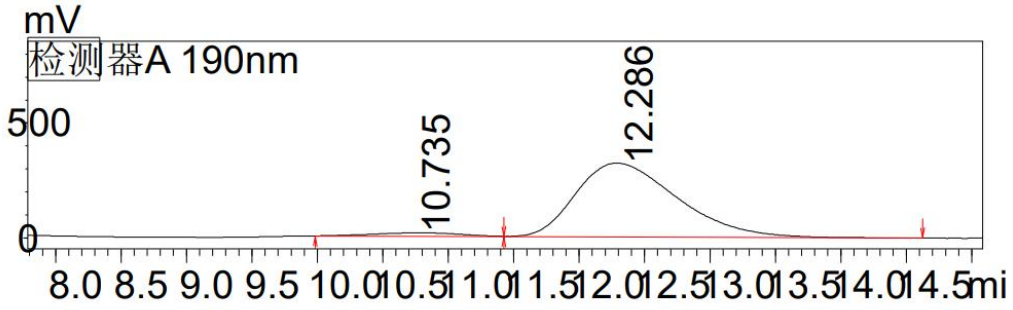

Peak#	Ret. Time	Area	Height	Area%	Height%
1	10.735	725434	15595	4.002	4.627
2	12.286	17400117	321484	95.998	95.373
Total		18125550	337079	100.000	100.000

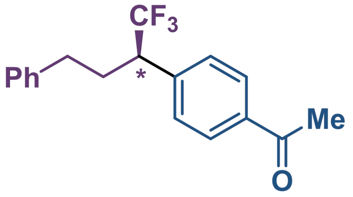

3j

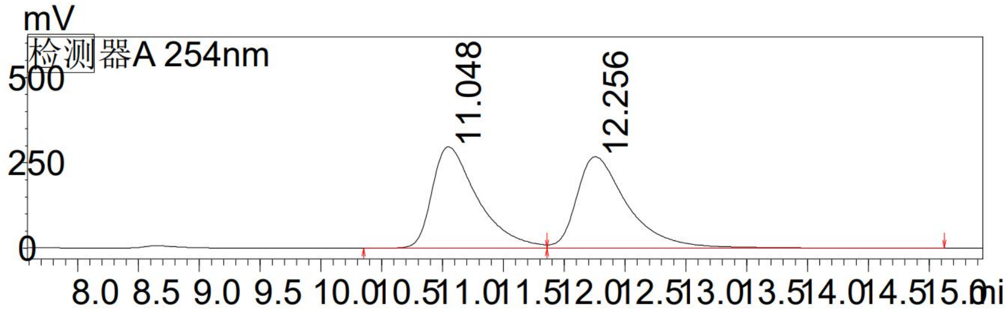

Peak#	Ret. Time	Area	Height	Area%	Height%
1	11.048	7735513	296646	49.782	52.590
2	12.256	7803181	267428	50.218	47.410
Total		15538694	564074	100.000	100.000

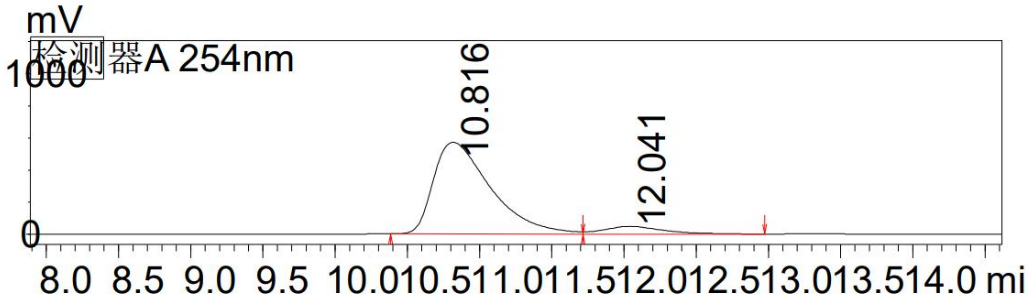

Peak#	Ret. Time	Area	Height	Area%	Height%
1	10.816	15753223	570279	92.117	92.344
2	12.041	1348018	47279	7.883	7.656
Total		1760653	63584	100.000	100.000

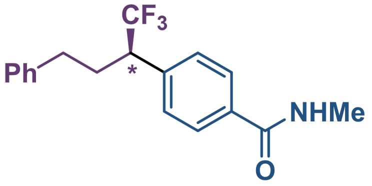

3k

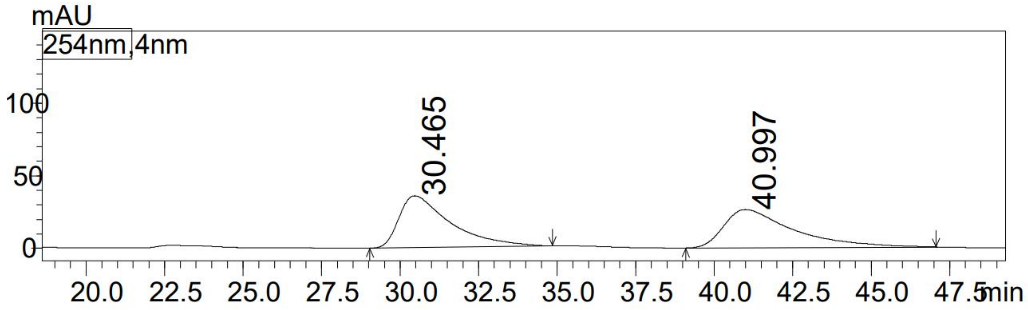

Peak#	Ret. Time	Area	Height	Area%	Height%
1	30.465	4053445	35737	50.077	57.609
2	40.997	4040948	26296	49.923	42.391
Total		8094393	62033	100.000	100.000

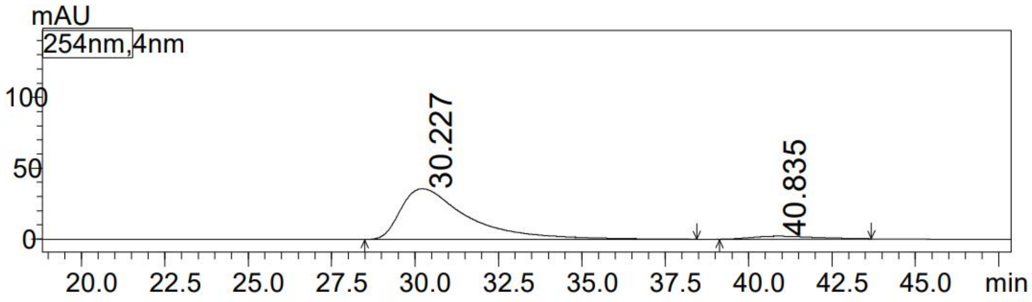

Peak#	Ret. Time	Area	Height	Area%	Height%
1	30.227	5020960	35788	95.002	94.686
2	40.835	264123	2008	4.998	5.314
Total		5285083	37796	100.000	100.000

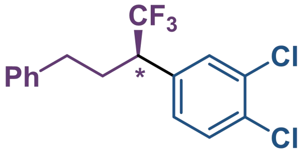

3l

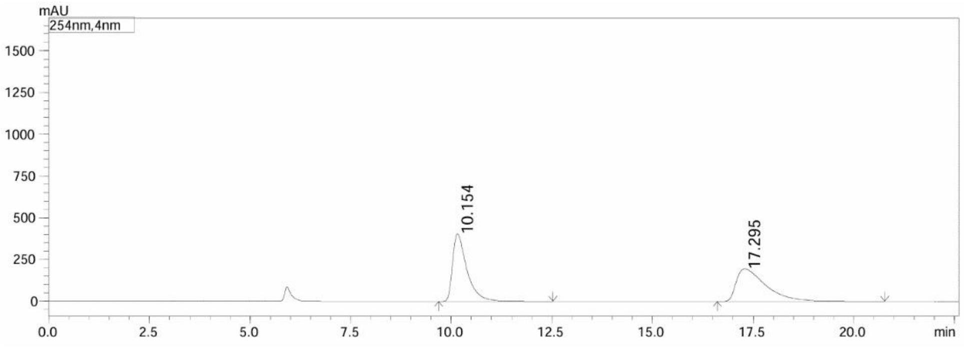

Peak#	Ret. Time	Area	Height	Area%	Height%
1	10.154	10030065	403851	49.965	67.517
2	17.295	10044058	194300	50.035	32.483
Total		20074123	598150	100.000	100.000

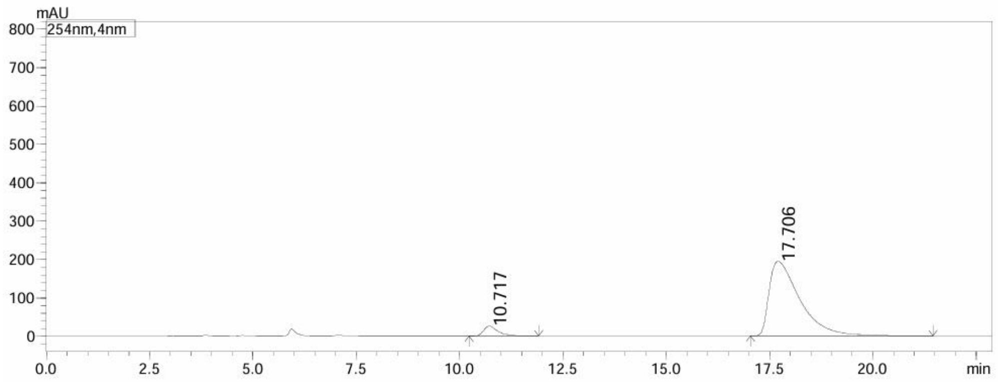

Peak#	Ret. Time	Area	Height	Area%	Height%
1	10.717	631638	25864	5.903	11.761
2	17.706	10069447	194050	94.097	88.239
Total		10701085	219913	100.000	100.000

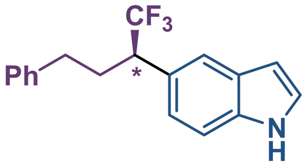

3m

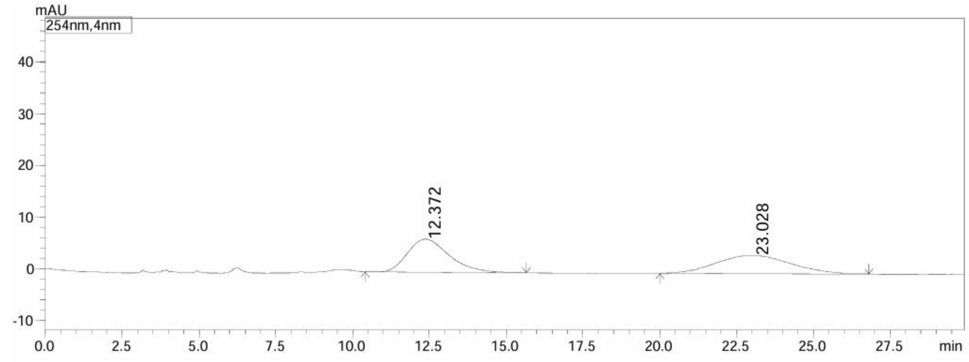

Peak#	Ret. Time	Area	Height	Area%	Height%
1	12.372	599826	6385	50.066	64.309
2	23.028	598255	3544	49.934	43.521
Total		1198081	9928	100.000	100.000

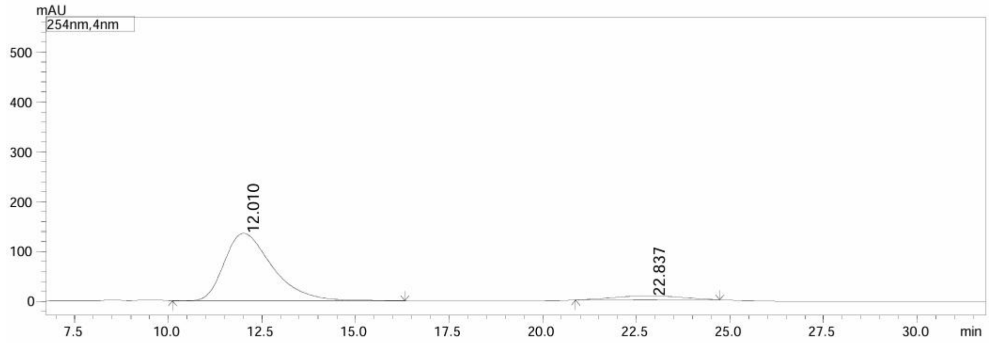

Peak#	Ret. Time	Area	Height	Area%	Height%
1	12.010	12111348	135354	91.858	94.257
2	22.837	1073541	8247	8.142	5.743
Total		13184889	143602	100.000	100.000

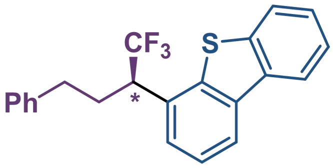

3n

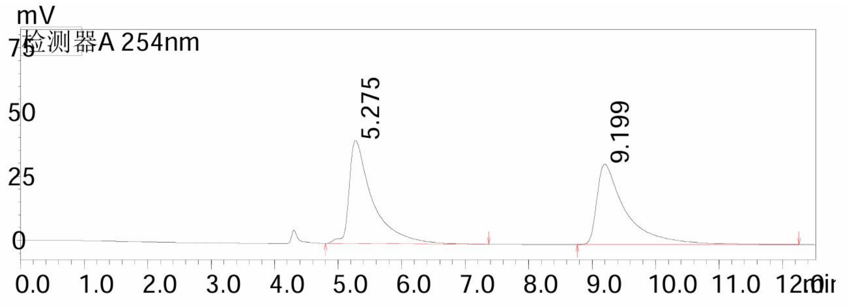

Peak#	Ret. Time	Area	Height	Area%	Height%
1	5.275	1030687	40185	50.989	56.236
2	9.199	990687	31273	49.011	43.764
Total		2021374	71458	100.000	100.000

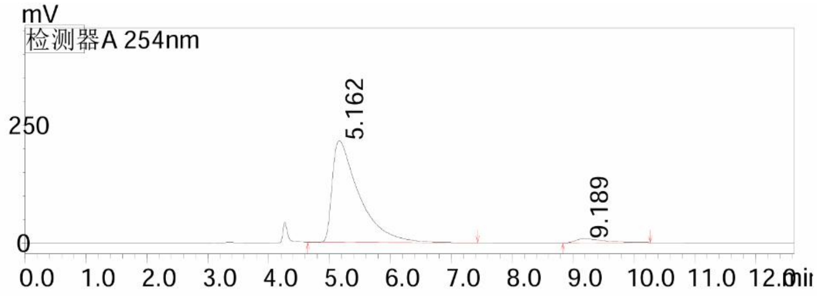

Peak#	Ret. Time	Area	Height	Area%	Height%
1	5.162	6258466	216138	91.032	94.235
2	9.189	692902	9062	8.968	5.765
Total		6951368	225200	100.000	100.000

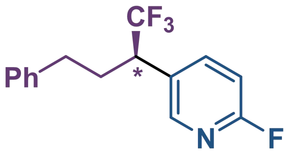

3o

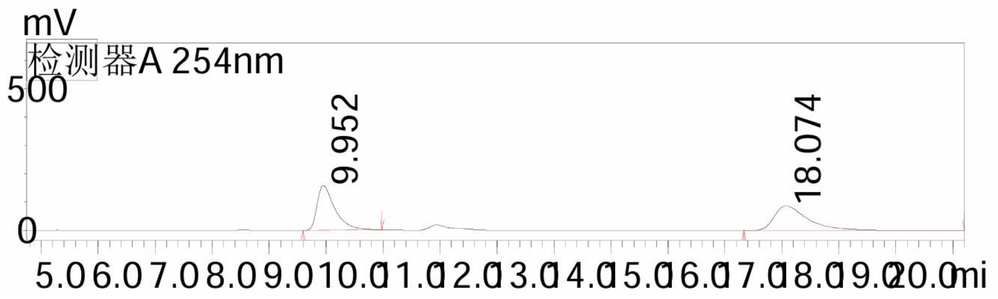

Peak#	Ret. Time	Area	Height	Area%	Height%
1	9.952	3411667	158114	49.265	64.666
2	18.074	3513481	86394	50.735	35.334
Total		6925147	244508	100.000	100.000

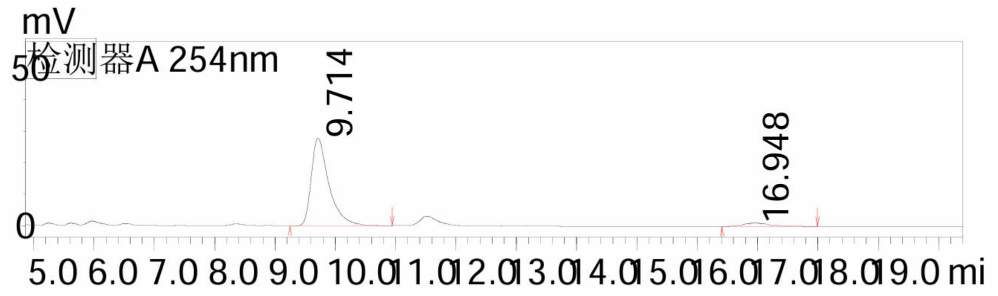

Peak#	Ret. Time	Area	Height	Area%	Height%
1	9.714	567097	27866	93.948	96.166
2	16.948	36531	1111	6.052	3.834
Total		603627	28976	100.000	100.000

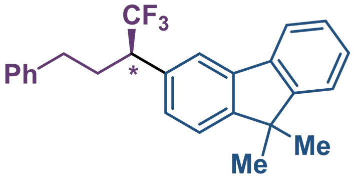

3p

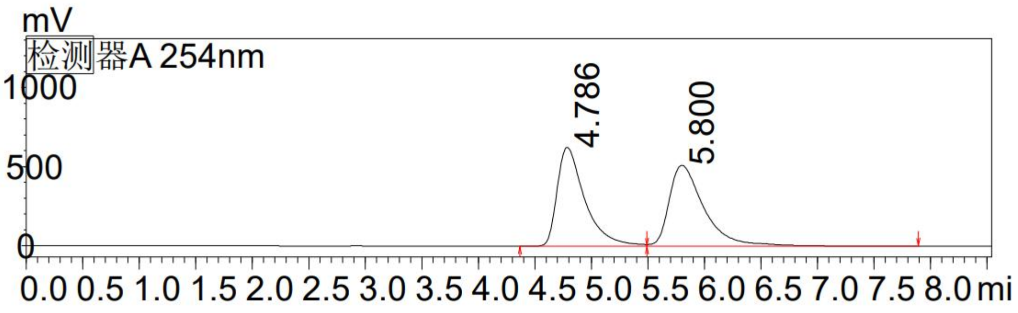

Peak#	Ret. Time	Area	Height	Area%	Height%
1	4.786	10565591	622637	49.258	54.993
2	5.800	10883805	509574	50.742	45.007
Total		21449396	1132211	100.000	100.000

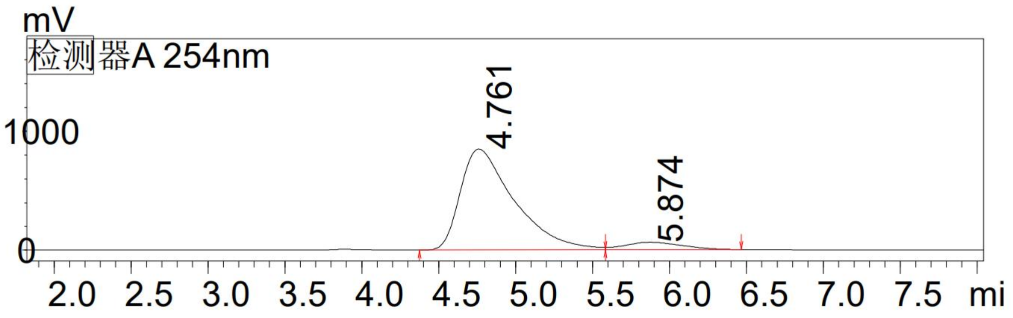

Peak#	Ret. Time	Area	Height	Area%	Height%
1	4.761	20670690	846785	92.873	93.211
2	5.874	1586146	61678	7.127	6.789
Total		22256835	908463	100.000	100.000

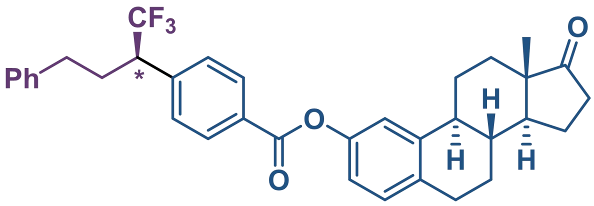

3q

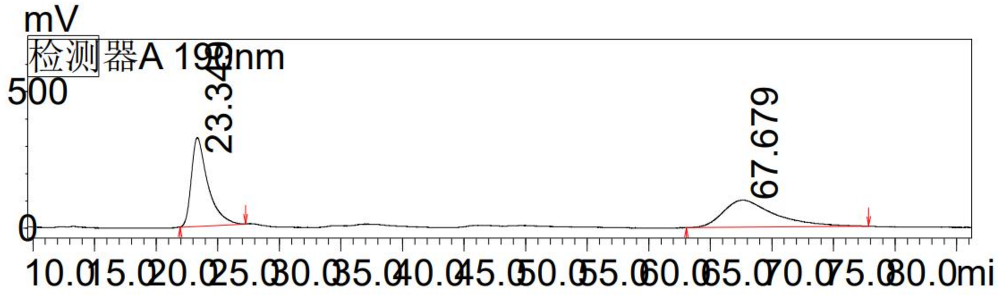

Peak#	Ret. Time	Area	Height	Area%	Height%
1	23.349	30224940	324725	50.963	76.533
2	67.679	29083077	99572	49.037	23.467
Total		59308017	424297	100.000	100.000

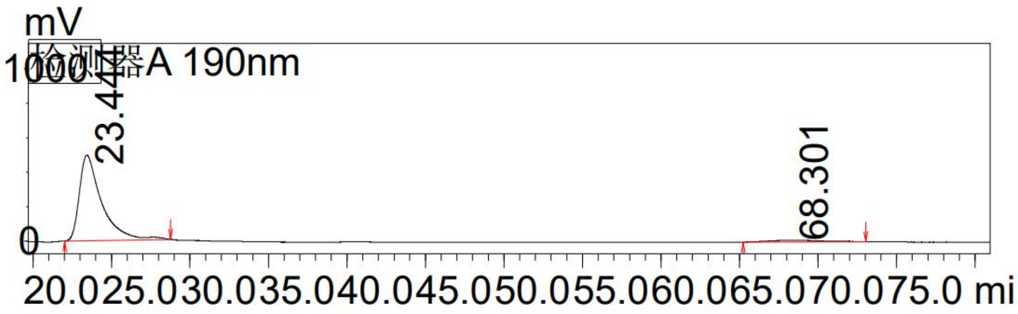

Peak#	Ret. Time	Area	Height	Area%	Height%
1	23.444	49244364	496570	95.515	97.964
2	68.301	2312188	10323	4.485	2.036
Total		51556552	506893	100.000	100.000

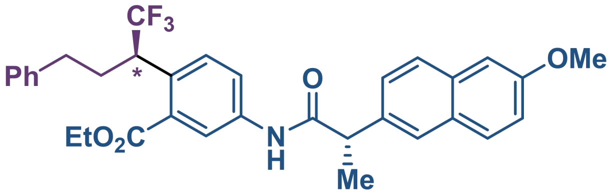

3r

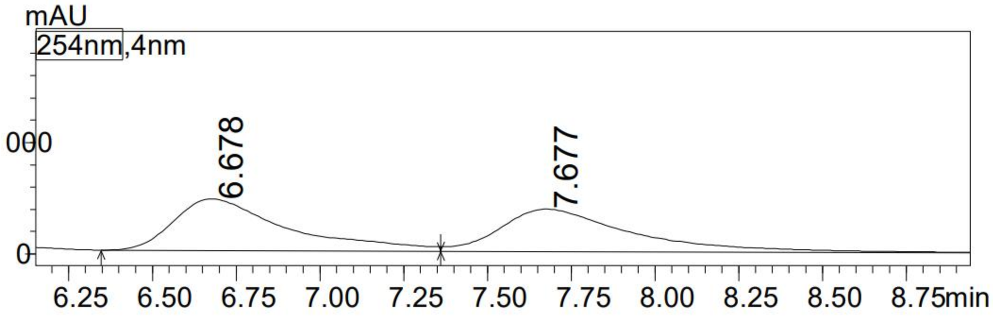

Peak#	Ret. Time	Area	Height	Area%	Height%
1	6.678	10849222	462187	51.158	54.921
2	7.677	10357866	379357	48.842	45.079
Total		21207088	841544	100.000	100.000

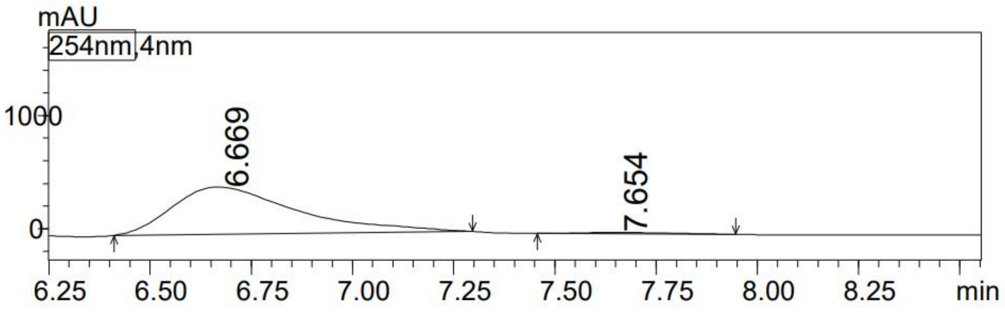

Peak#	Ret. Time	Area	Height	Area%	Height%
1	6.669	8957389	416899	97.666	96.761
2	7.654	214040	13958	2.334	3.239
Total		9171430	430856	100.000	100.000

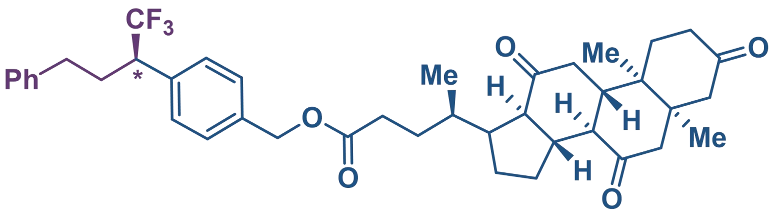

3s

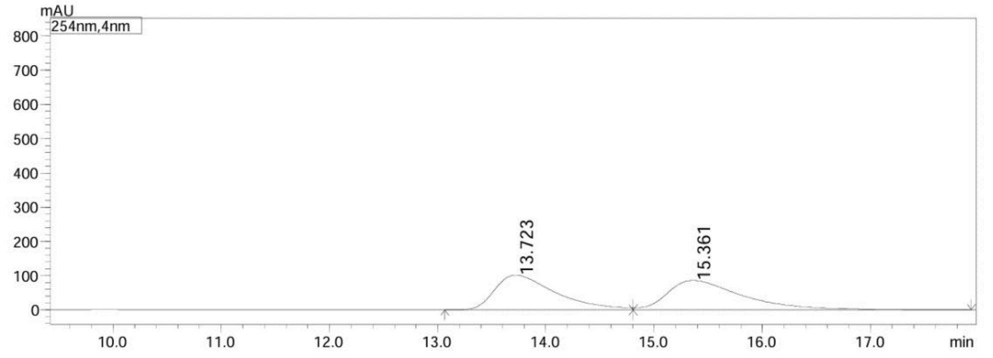

Peak#	Ret. Time	Area	Height	Area%	Height%
1	13.723	3960091	101410	48.888	54.117
2	15.361	4140293	85981	51.112	45.883
Total		8100384	187391	100.000	100.000

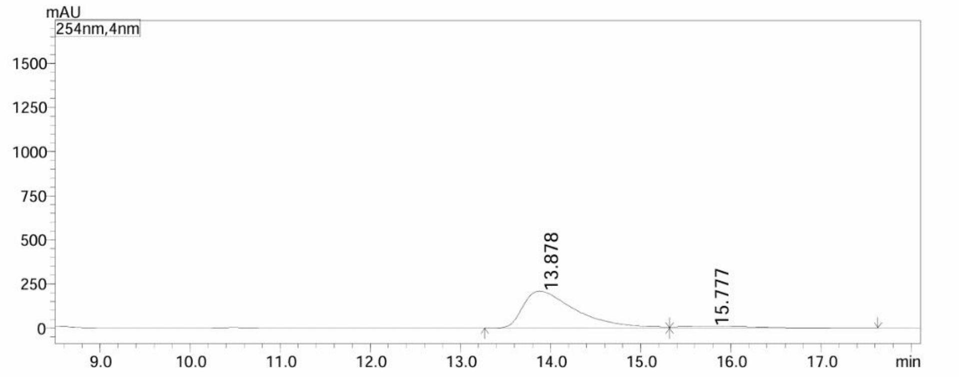

Peak#	Ret. Time	Area	Height	Area%	Height%
1	13.878	8519324	209790	93.442	94.742
2	15.777	597941	11642	6.558	5.258
Total		9117265	221432	100.000	100.000

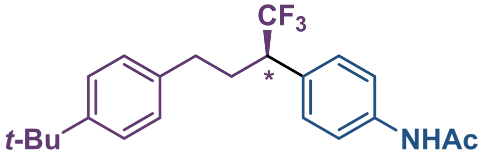

3t

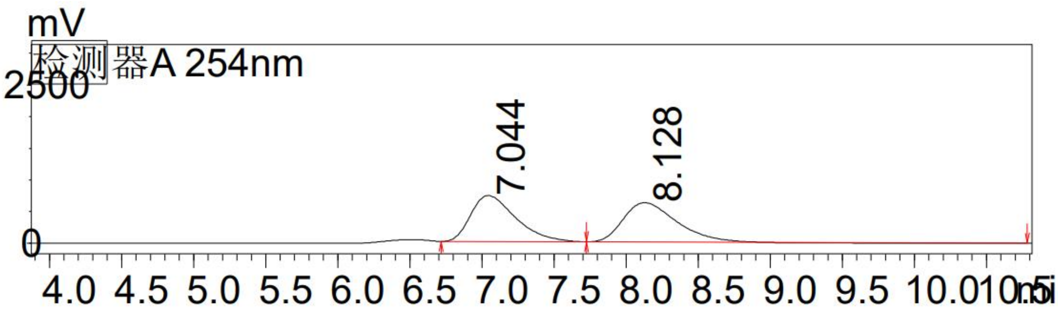

Peak#	Ret. Time	Area	Height	Area%	Height%
1	7.044	15699397	725421	50.079	53.864
2	8.128	15649673	621332	49.921	46.136
Total		31349070	1346753	100.000	100.000

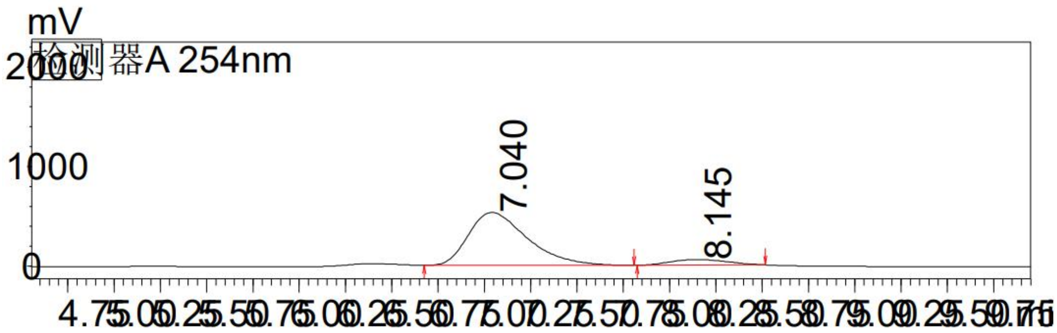

Peak#	Ret. Time	Area	Height	Area%	Height%
1	7.040	11731600	530453	90.781	90.358
2	8.145	1191429	56603	9.219	9.642
Total		12923030	587057	100.000	100.000

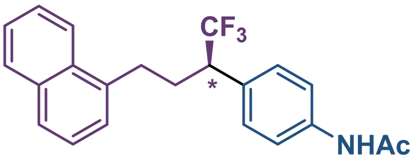

3u

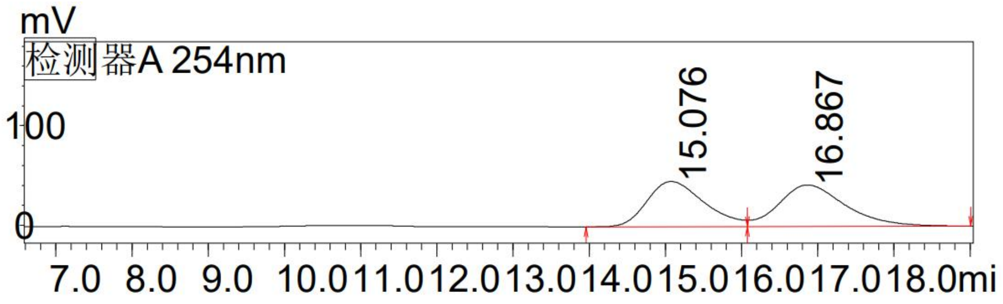

Peak#	Ret. Time	Area	Height	Area%	Height%
1	15.076	2380556	45479	48.249	52.098
2	16.867	2553386	41816	51.751	47.902
Total		4933942	87295	100.000	100.000

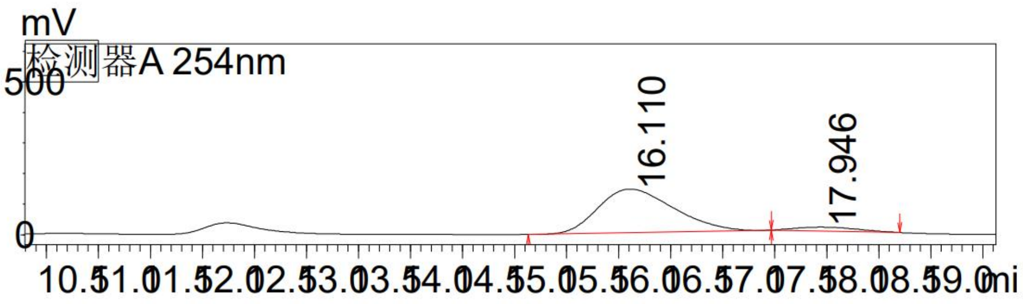

Peak#	Ret. Time	Area	Height	Area%	Height%
1	16.110	7175377	142597	93.032	91.622
2	17.946	537399	13039	6.968	8.378
Total		7712777	155636	100.000	100.000

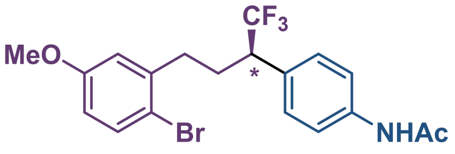

3v

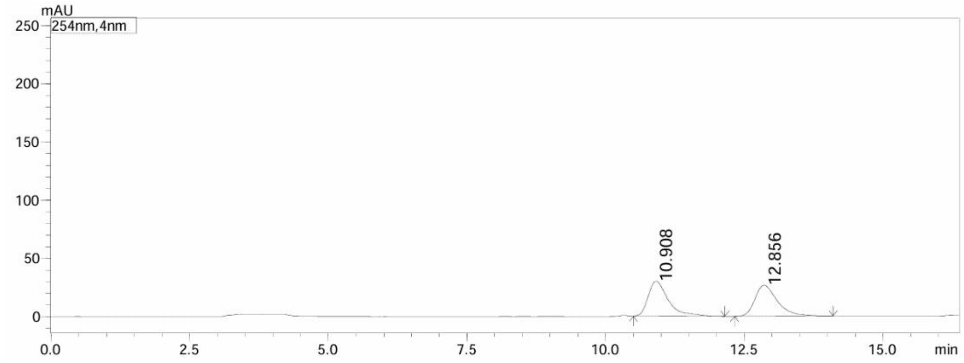

Peak#	Ret. Time	Area	Height	Area%	Height%
1	10.908	746137	29935	50.274	53.023
2	12.856	738010	26522	49.726	46.977
Total		1484146	56457	100.000	100.000

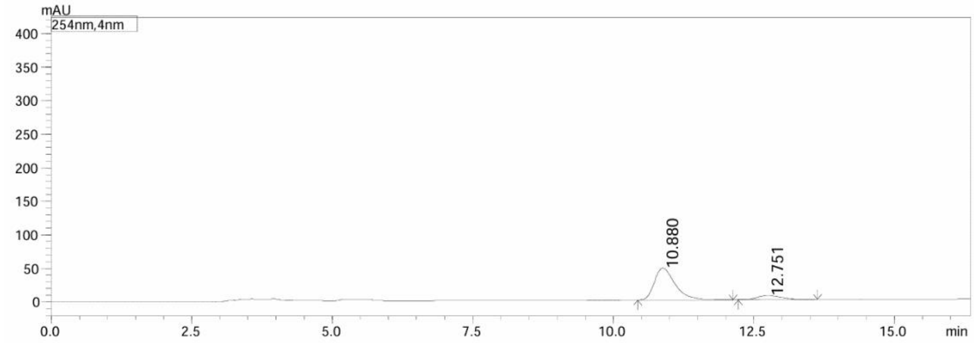

Peak#	Ret. Time	Area	Height	Area%	Height%
1	10.880	1218393	47560	86.512	88.365
2	12.751	189961	6262	13.488	11.635
Total		1408355	53822	100.000	100.000

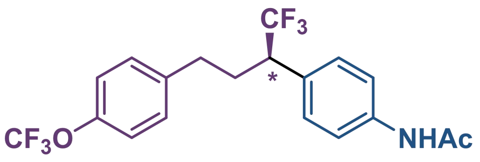

3w

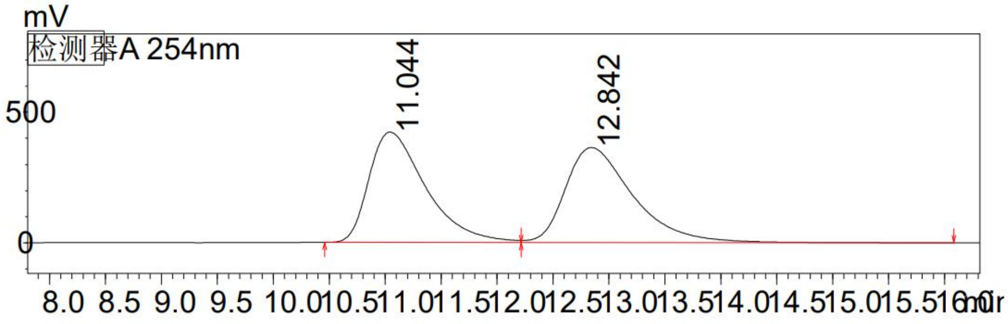

Peak#	Ret. Time	Area	Height	Area%	Height%
1	11.044	14865572	421887	49.238	53.704
2	12.842	15325585	363685	50.762	46.296
Total		30191157	785573	100.000	100.000

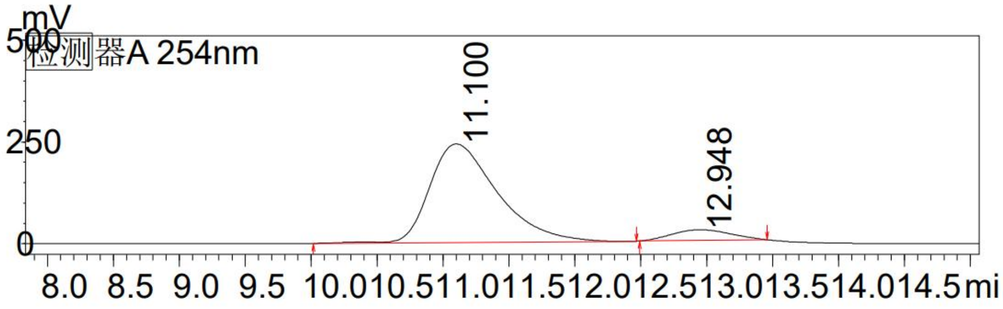

Peak#	Ret. Time	Area	Height	Area%	Height%
1	11.100	8630566	242068	91.432	90.238
2	12.948	808730	26188	8.568	9.762
Total		9439296	268256	100.000	100.000

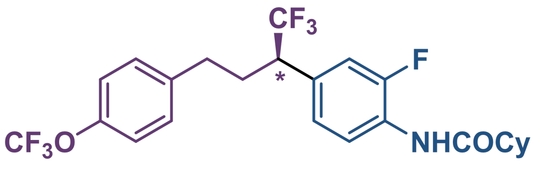

3x

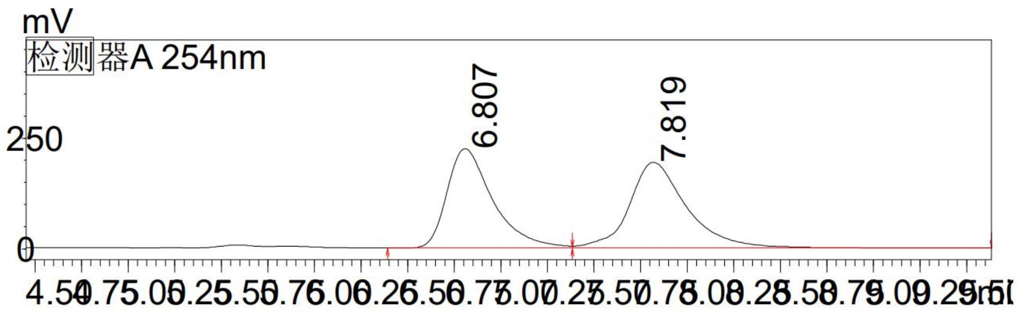

Peak#	Ret. Time	Area	Height	Area%	Height%
1	6.807	3831395	224030	47.940	53.664
2	7.819	4160694	193440	52.060	46.336
Total		7992089	417470	100.000	100.000

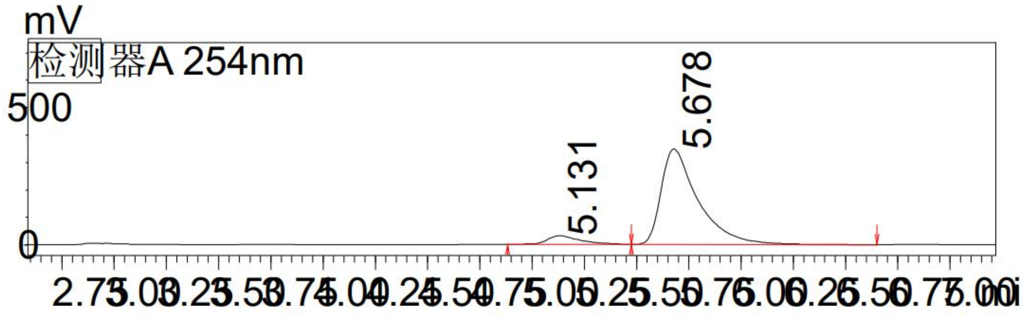

Peak#	Ret. Time	Area	Height	Area%	Height%
1	5.131	361585	32090	7.720	8.421
2	5.678	4322030	348961	92.280	91.579
Total		4683615	381050	100.000	100.000

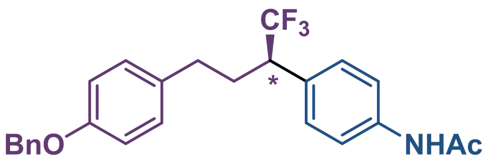

3y

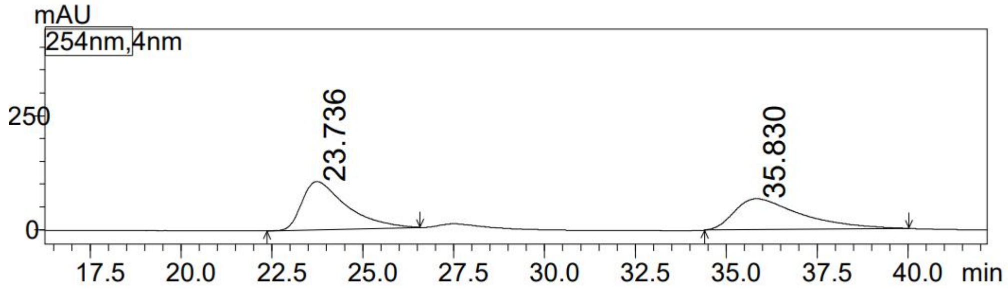

Peak#	Ret. Time	Area	Height	Area%	Height%
1	23.736	8895507	105098	50.671	60.826
2	35.830	8659881	67688	49.329	39.174
Total		17555388	172787	100.000	100.000

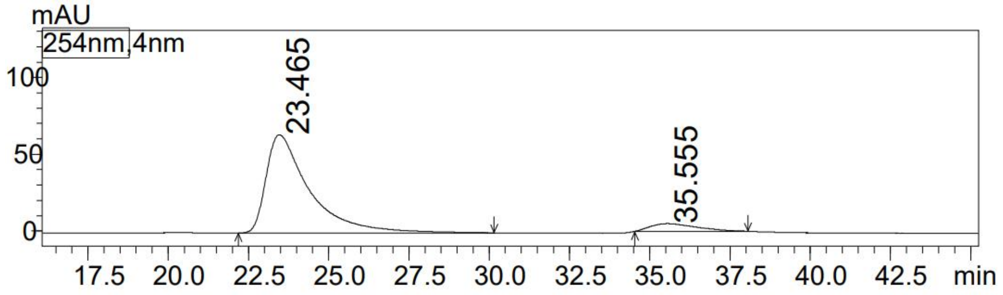

Peak#	Ret. Time	Area	Height	Area%	Height%
1	23.465	5996817	63936	92.242	92.756
2	35.555	504371	4994	7.758	7.244
Total		6501188	68929	100.000	100.000

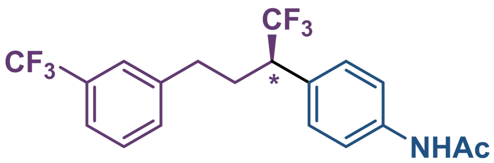

3z

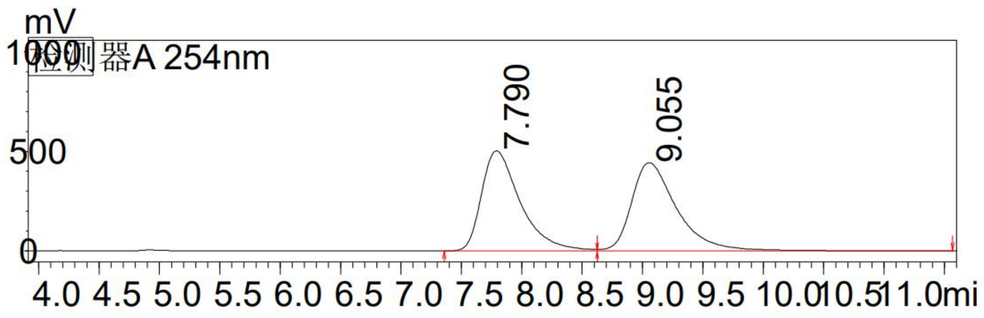

Peak#	Ret. Time	Area	Height	Area%	Height%
1	7.790	11193724	501894	49.336	53.133
2	9.055	11494920	442710	50.664	46.867
Total		22688644	944604	100.000	100.000

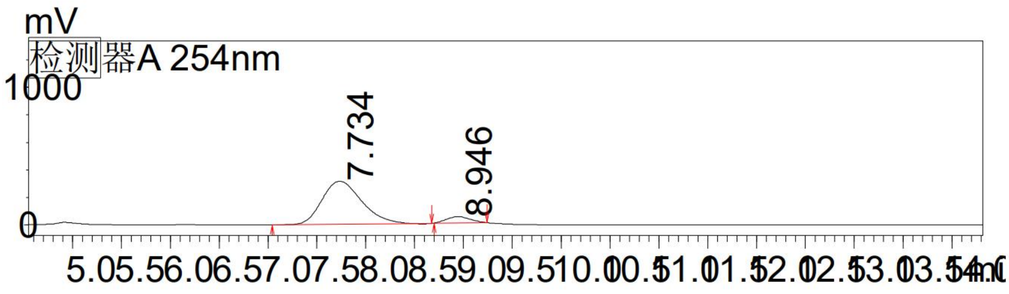

Peak#	Ret. Time	Area	Height	Area%	Height%
1	7.734	8847468	313413	92.182	87.582
2	8.946	750341	44440	7.818	12.418
Total		9597810	357852	100.000	100.000

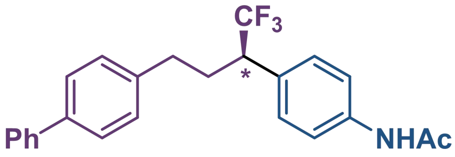

3aa

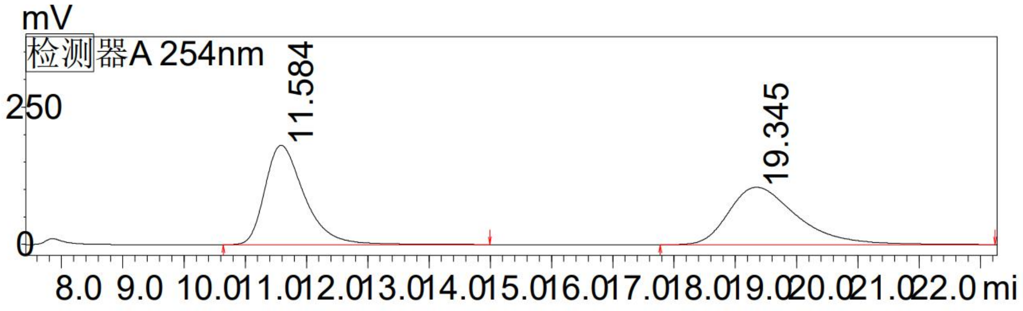

Peak#	Ret. Time	Area	Height	Area%	Height%
1	11.584	8079120	180694	49.888	63.355
2	19.345	8115334	104514	50.112	36.645
Total		16194454	285208	100.000	100.000

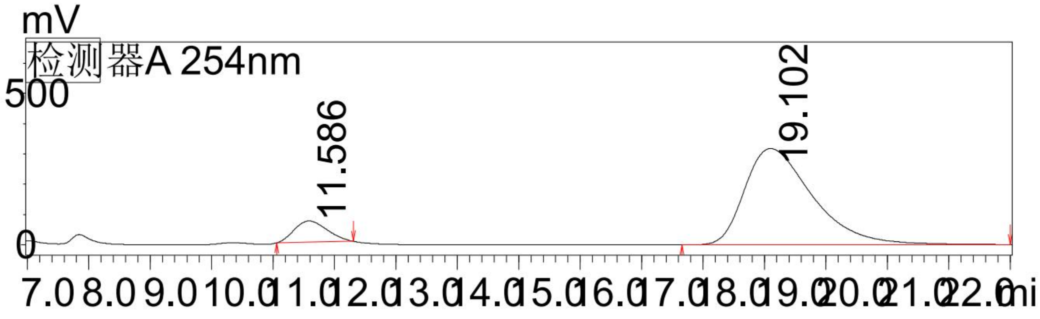

Peak#	Ret. Time	Area	Height	Area%	Height%
1	11.586	2607974	69920	9.666	18.028
2	19.102	24372994	317913	90.334	81.972
Total		26980968	387833	100.000	100.000

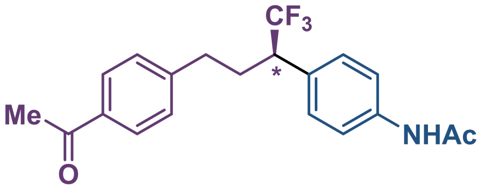

3ab

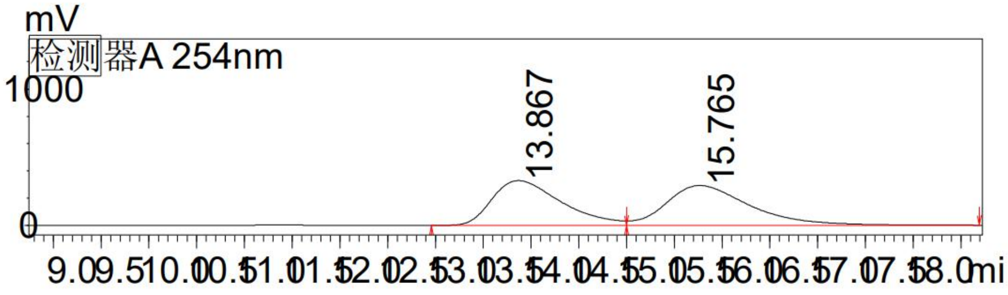

Peak#	Ret. Time	Area	Height	Area%	Height%
1	13.867	16516511	327911	48.279	52.965
2	15.765	17693736	291193	51.721	47.035
Total		34210246	619104	100.000	100.000

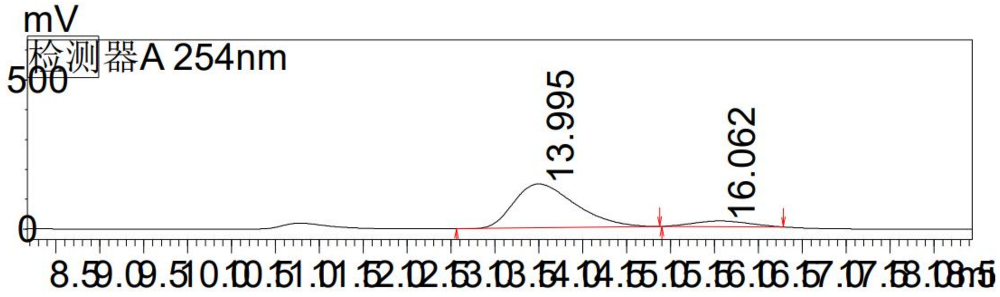

Peak#	Ret. Time	Area	Height	Area%	Height%
1	13.995	7165702	147127	89.528	88.637
2	16.062	838165	18861	10.472	11.363
Total		8003867	165988	100.000	100.000

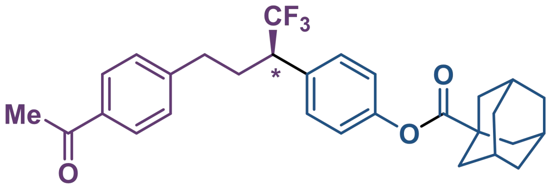

3ac

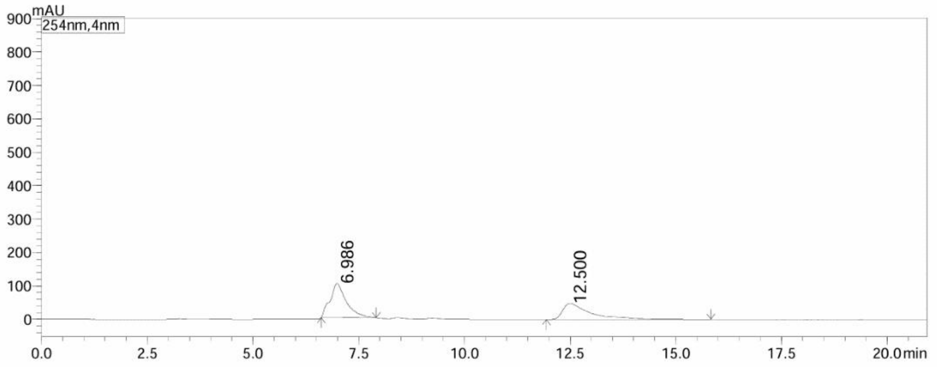

Peak#	Ret. Time	Area	Height	Area%	Height%
1	6.986	2757844	102157	54.948	67.897
2	12.500	2261133	48302	45.052	32.103
Total		5018976	150458	100.000	100.000

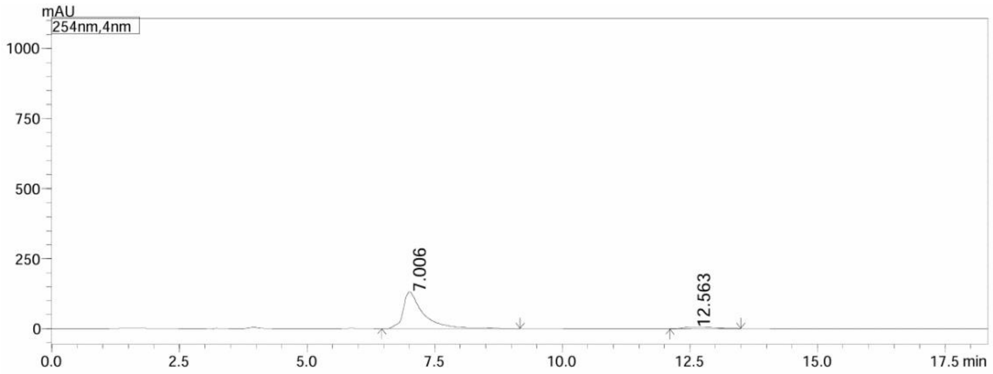

Peak#	Ret. Time	Area	Height	Area%	Height%
1	7.006	3574563	130823	94.215	95.890
2	12.563	219491	5608	5.785	4.110
Total		3794054	136430	100.000	100.000

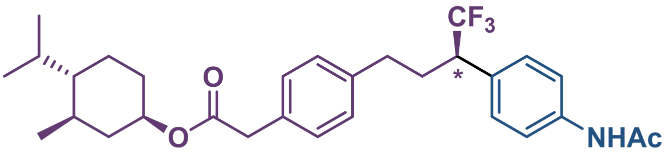

3ad

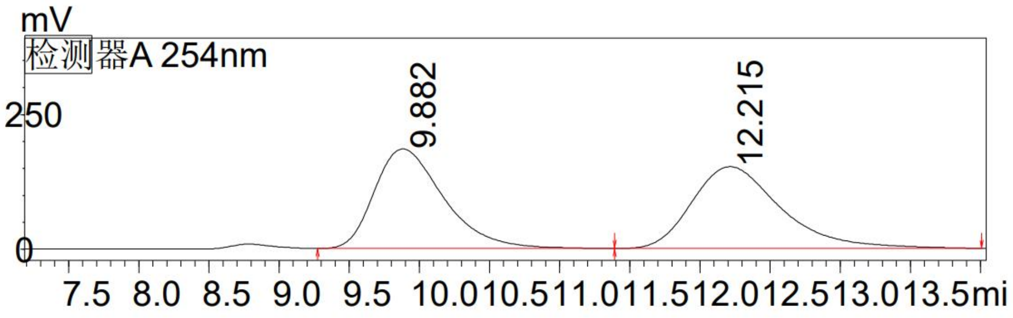

Peak#	Ret. Time	Area	Height	Area%	Height%
1	9.882	6361227	184691	48.663	54.904
2	12.215	6710728	151699	51.337	45.096
Total		13071955	336390	100.000	100.000

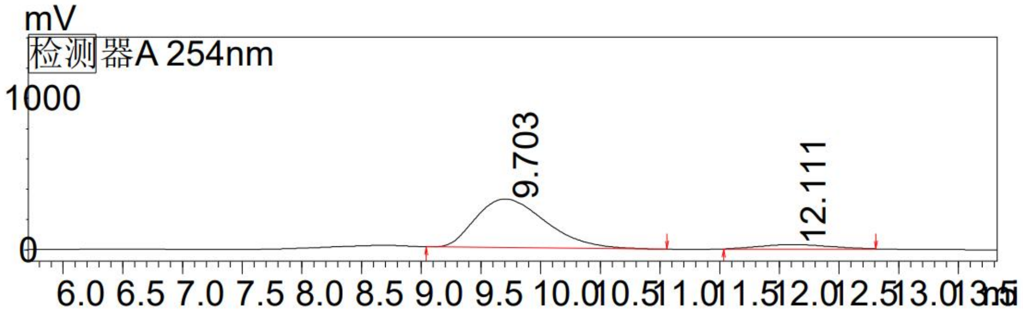

Peak#	Ret. Time	Area	Height	Area%	Height%
1	9.703	13146713	320180	92.087	91.993
2	12.111	1129735	27870	7.913	8.007
Total		14276448	348050	100.000	100.000

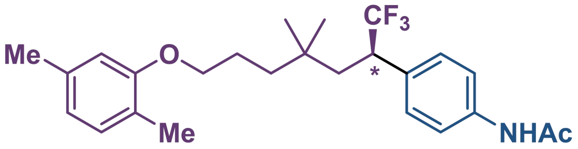

3ae

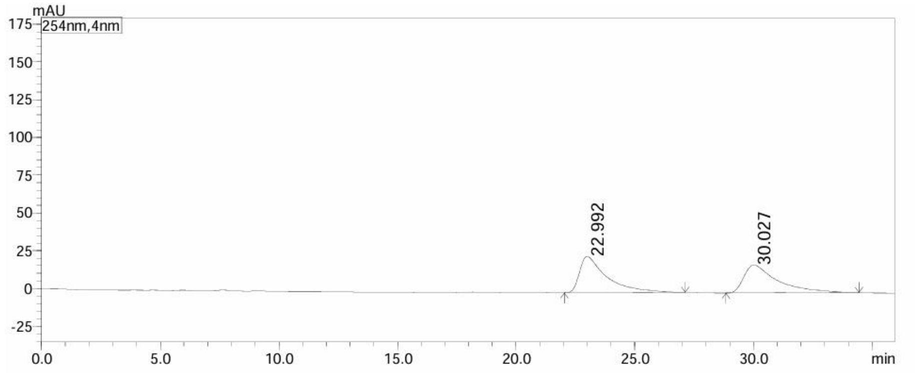

Peak#	Ret. Time	Area	Height	Area%	Height%
1	22.992	1914039	23922	51.215	56.522
2	30.027	1823217	18402	48.785	43.478
Total		3737255	42324	100.000	100.000

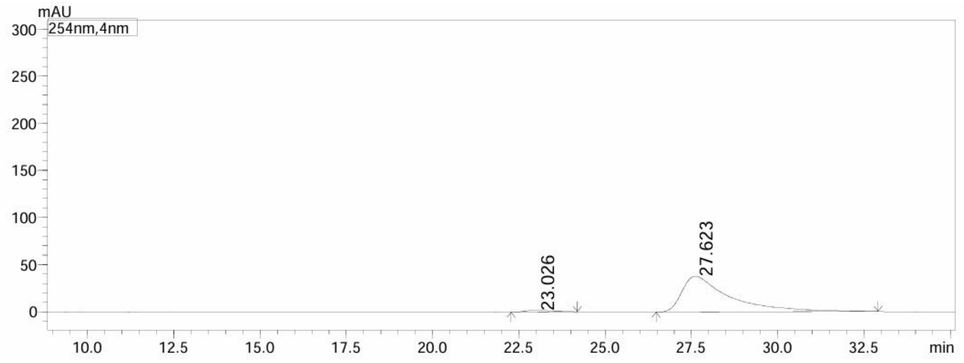

Peak#	Ret. Time	Area	Height	Area%	Height%
1	23.026	88638	1538	2.379	3.901
2	27.623	3637027	37895	97.621	96.099
Total		3725665	39433	100.000	100.000

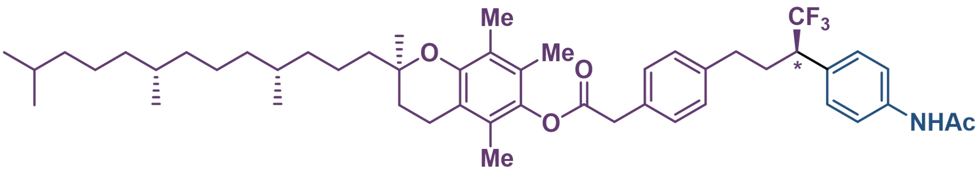

3af

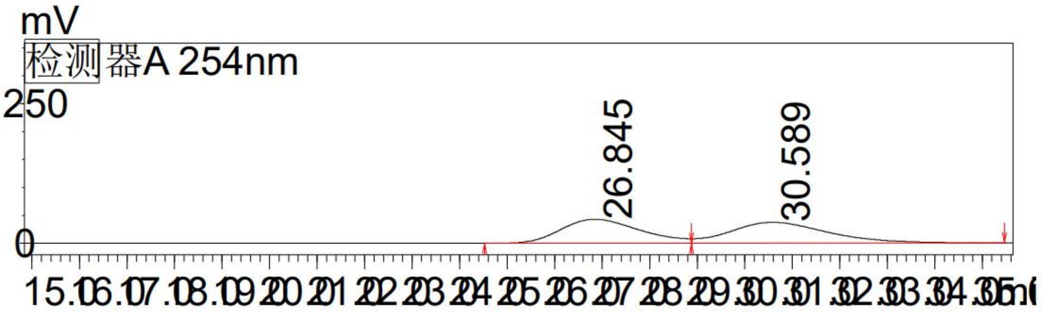

Peak#	Ret. Time	Area	Height	Area%	Height%
1	26.845	4974030	42902	48.325	53.511
2	30.589	5318748	37272	51.675	46.489
Total		10292779	80174	100.000	100.000

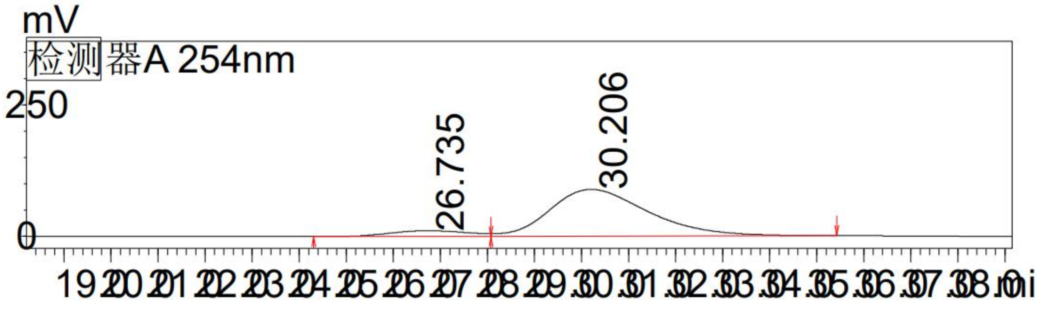

Peak#	Ret. Time	Area	Height	Area%	Height%
1	26.735	1265677	11064	8.896	11.069
2	30.206	12961181	88891	91.104	88.931
Total		14226858	99955	100.000	100.000

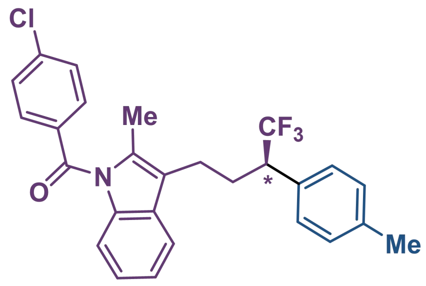

3ag

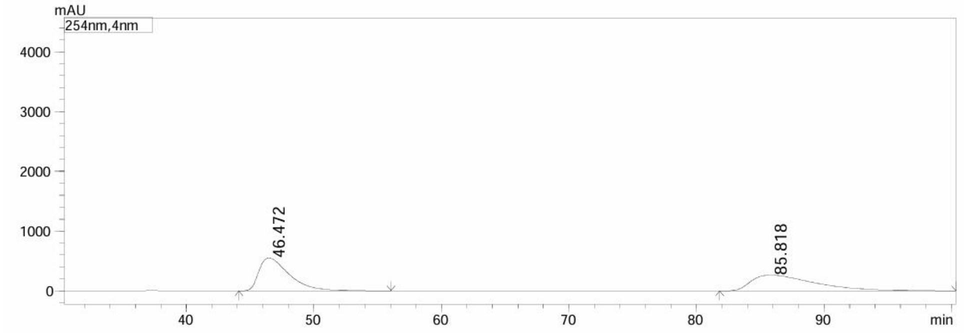

Peak#	Ret. Time	Area	Height	Area%	Height%
1	46.472	90406122	550768	50.052	67.535
2	85.818	90218358	264760	49.948	32.465
Total		180624480	815528	100.000	100.000

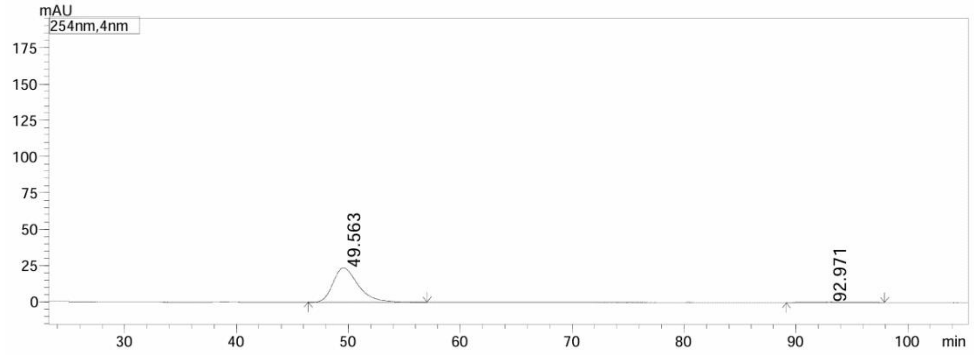

Peak#	Ret. Time	Area	Height	Area%	Height%
1	49.563	3678622	23600	97.078	98.234
2	92.971	110730	424	2.922	1.766
Total		3789352	24024	100.000	100.000

NMR Spectra

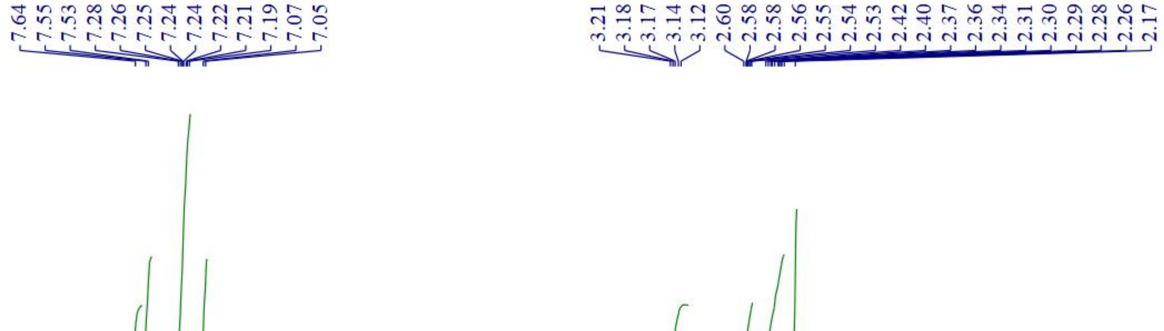

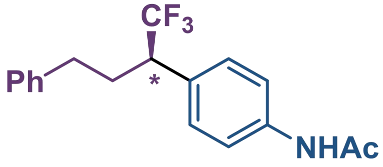

^{1}H NMR of **3a** (400 MHz, Chloroform-*d*, 298 K)

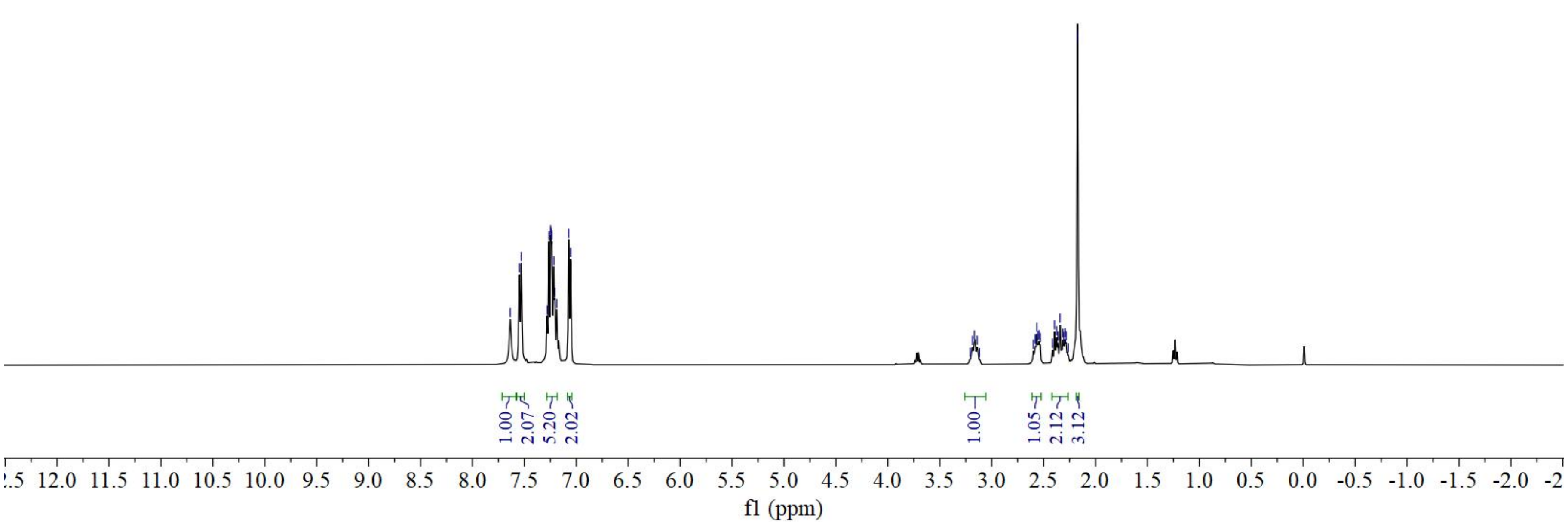

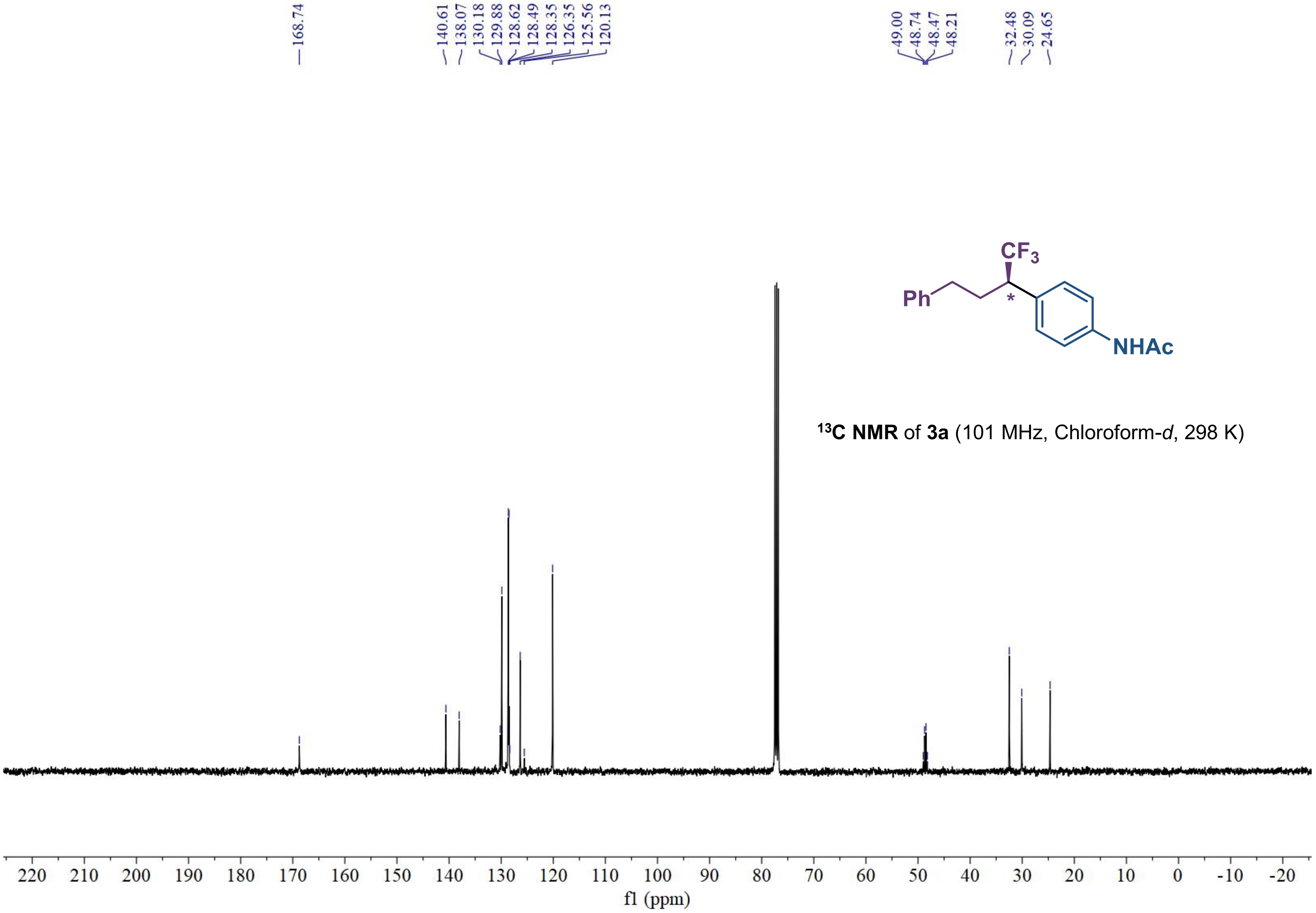

^{13}C NMR of **3a** (101 MHz, Chloroform-*d*, 298 K)

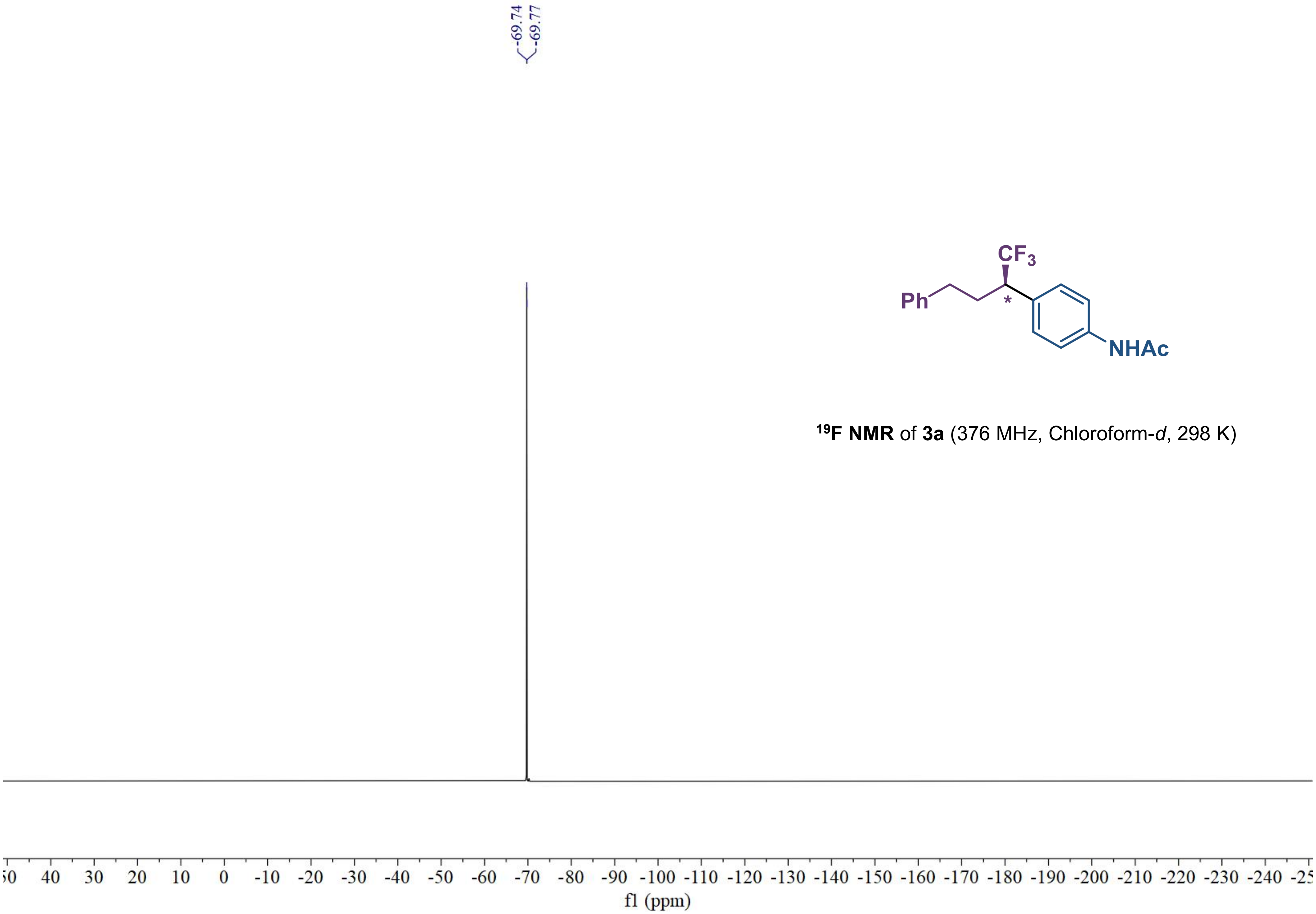

^{19}F NMR of **3a** (376 MHz, Chloroform-*d*, 298 K)

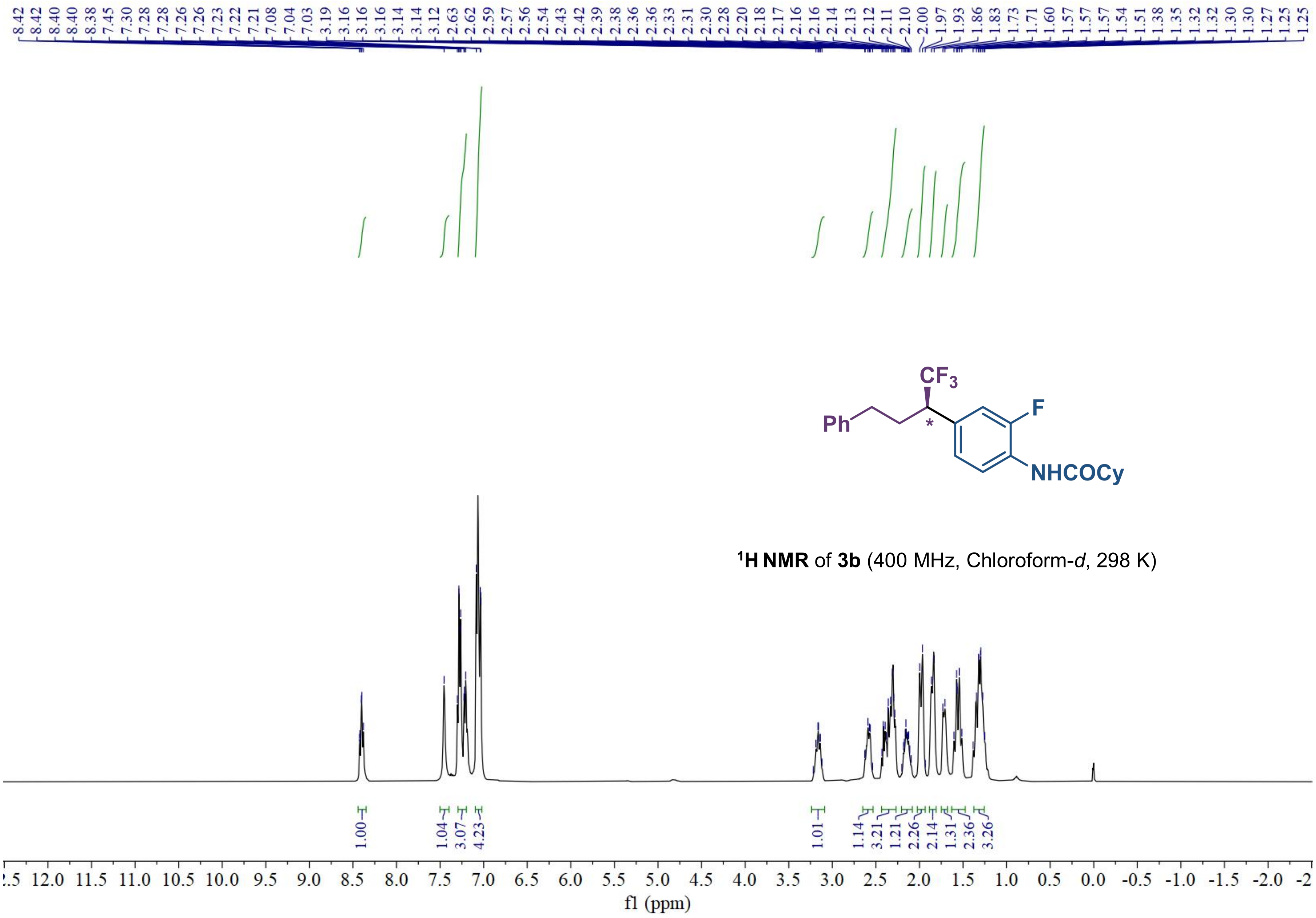

^{1}H NMR of **3b** (400 MHz, Chloroform-*d*, 298 K)

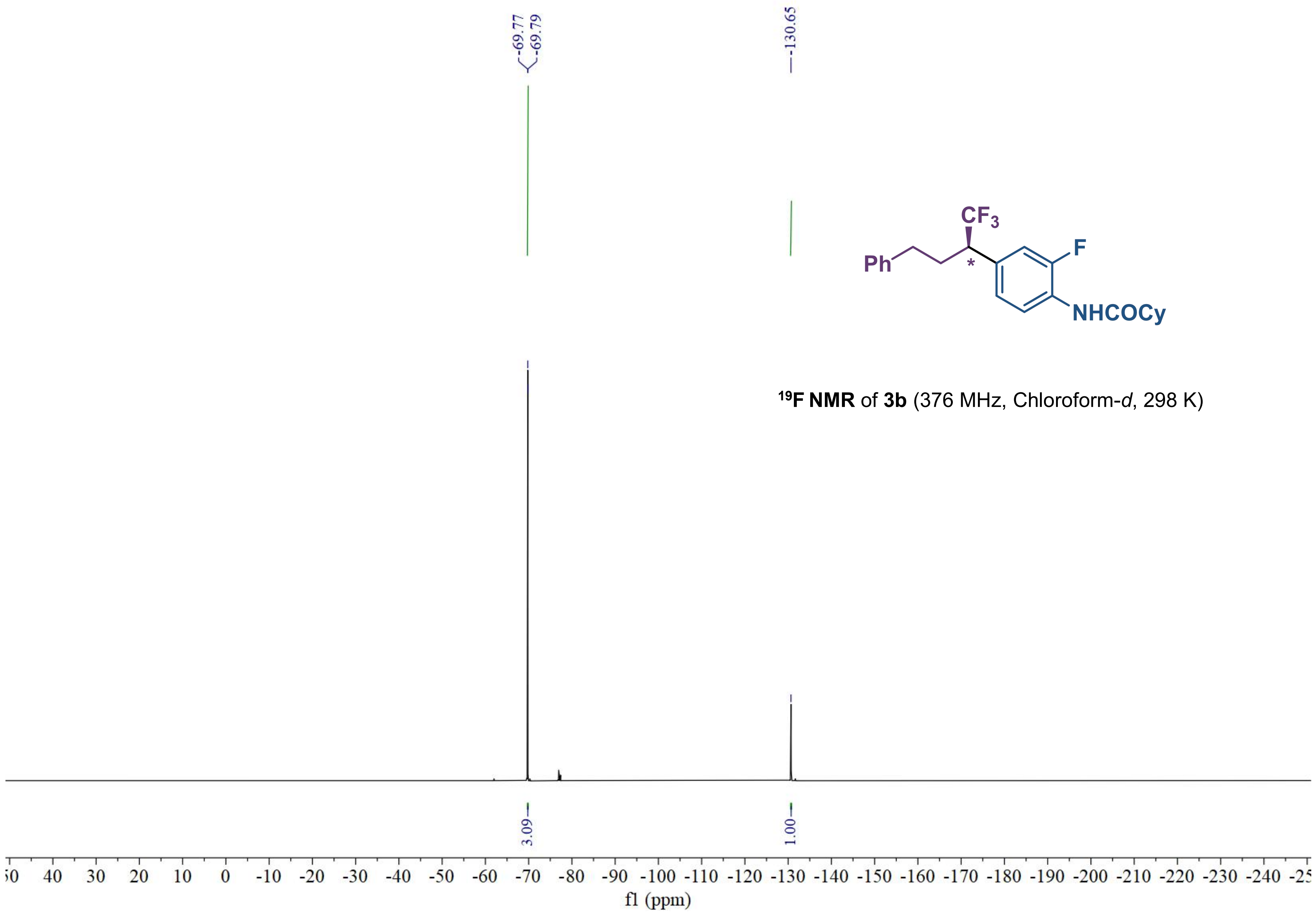

^{19}**F NMR** of **3b** (376 MHz, Chloroform-*d*, 298 K)

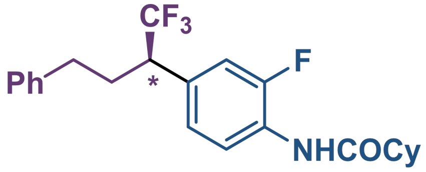

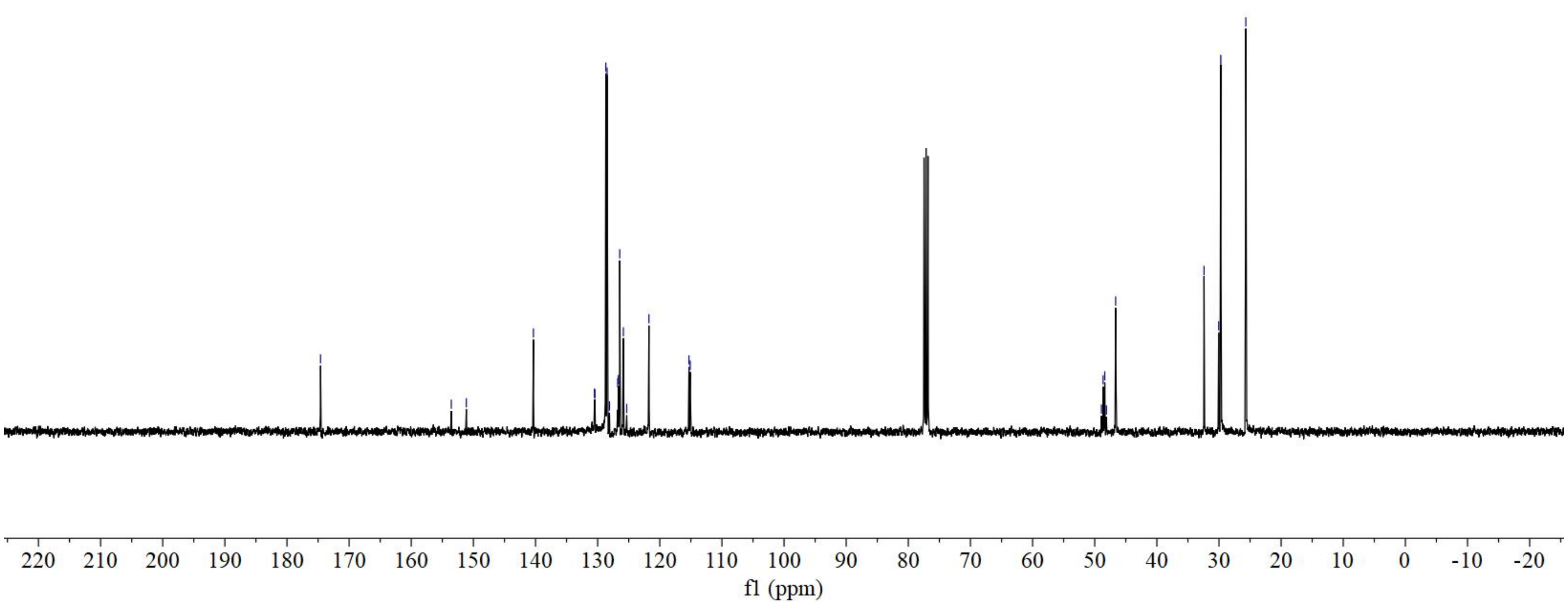

^{13}C NMR of **3b** (101 MHz, Chloroform-*d*, 298 K)

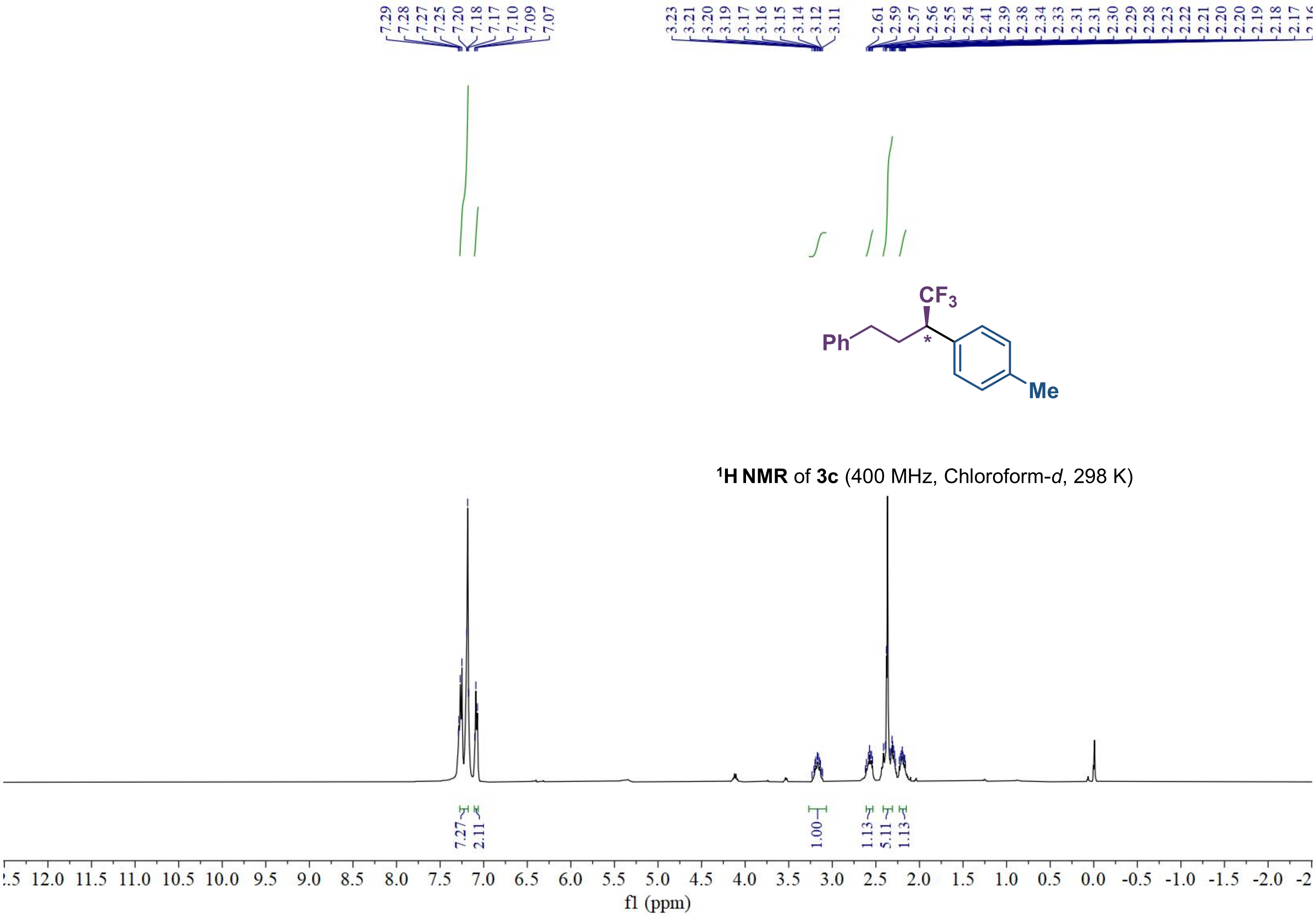

^{1}H NMR of **3c** (400 MHz, Chloroform-*d*, 298 K)

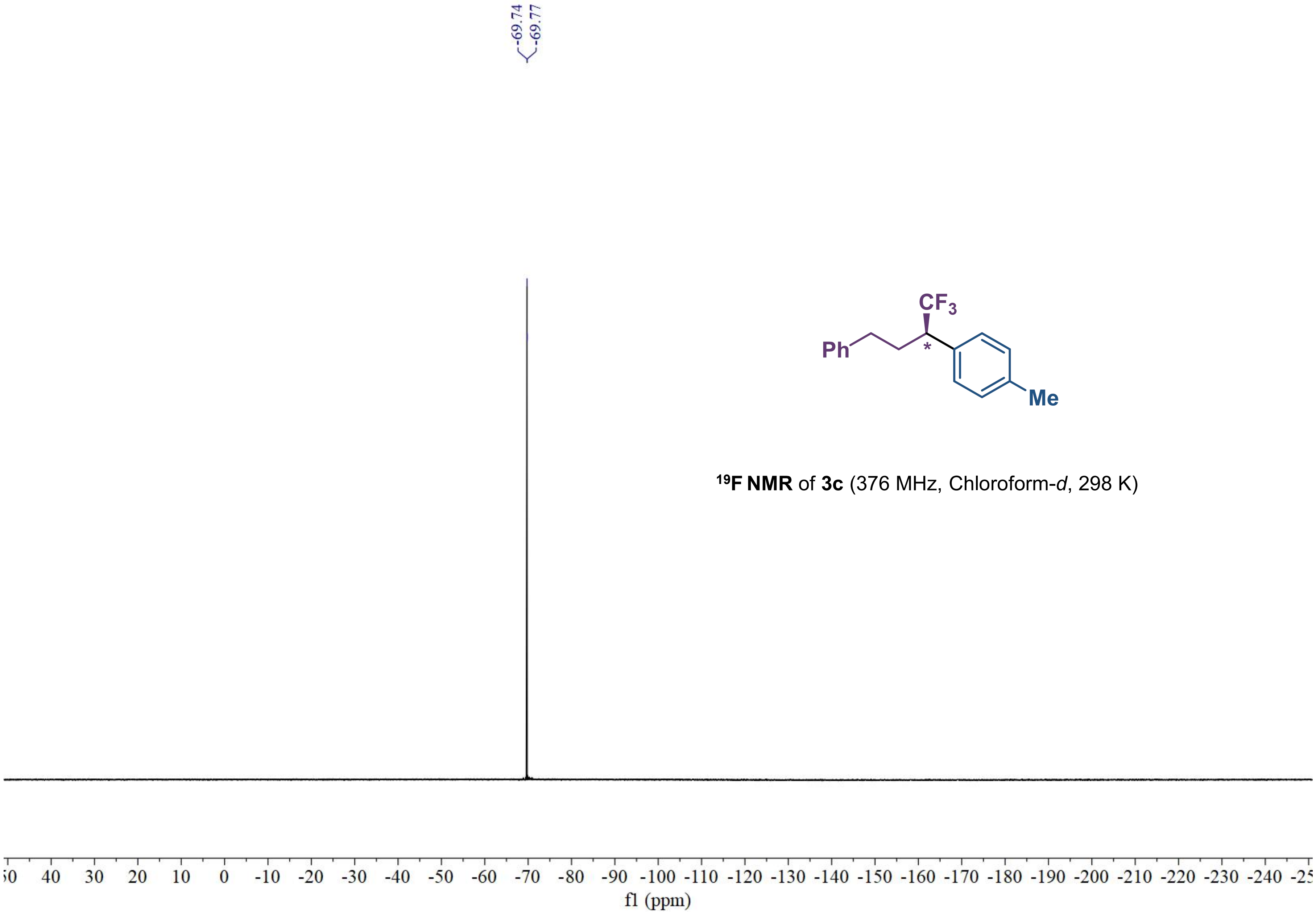

^{19}F NMR of **3c** (376 MHz, Chloroform-*d*, 298 K)

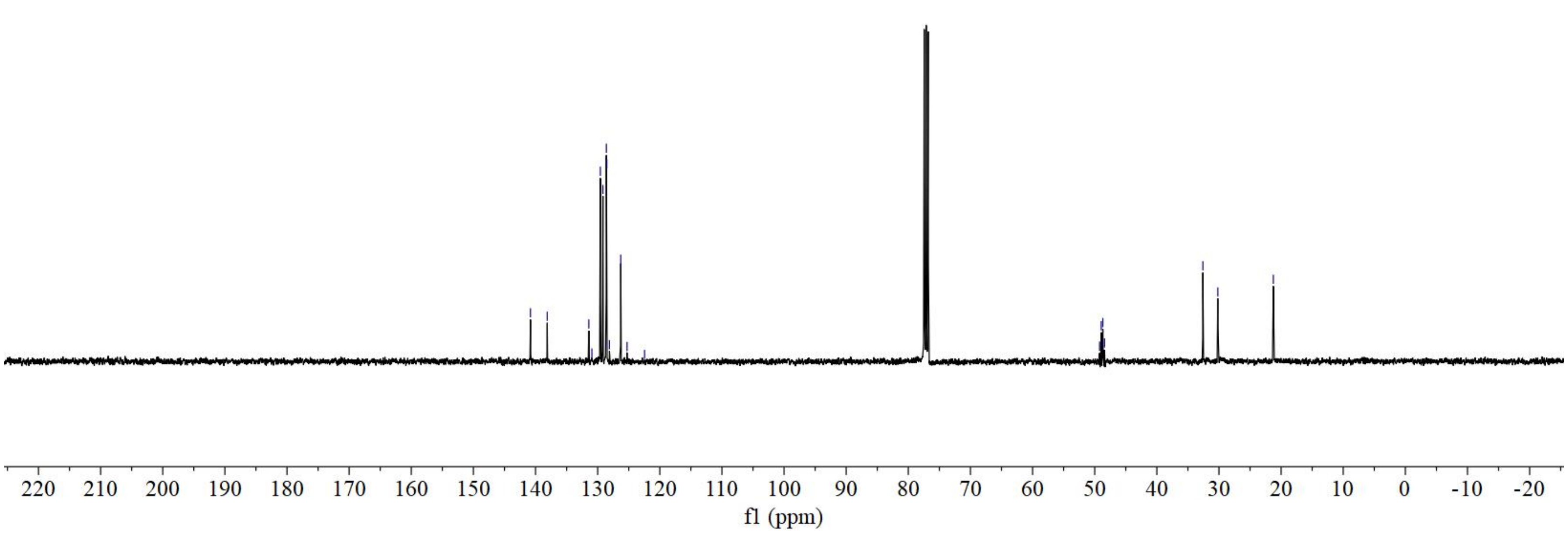

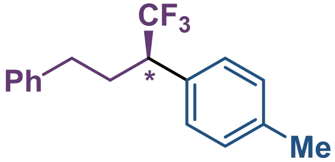

^{13}**C NMR** of **3c** (101 MHz, Chloroform-*d*, 298 K)

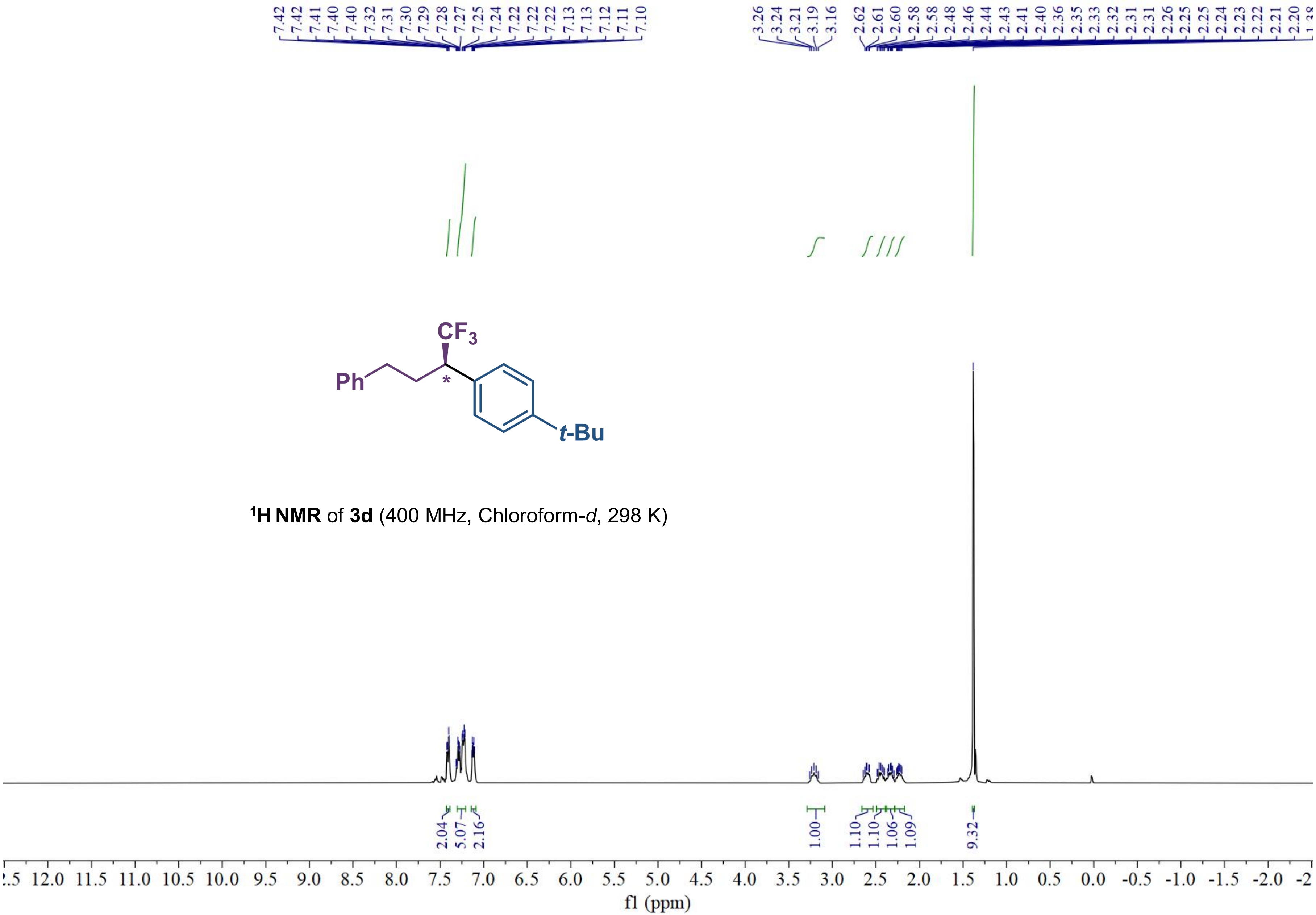

^{1}H NMR of **3d** (400 MHz, Chloroform-*d*, 298 K)

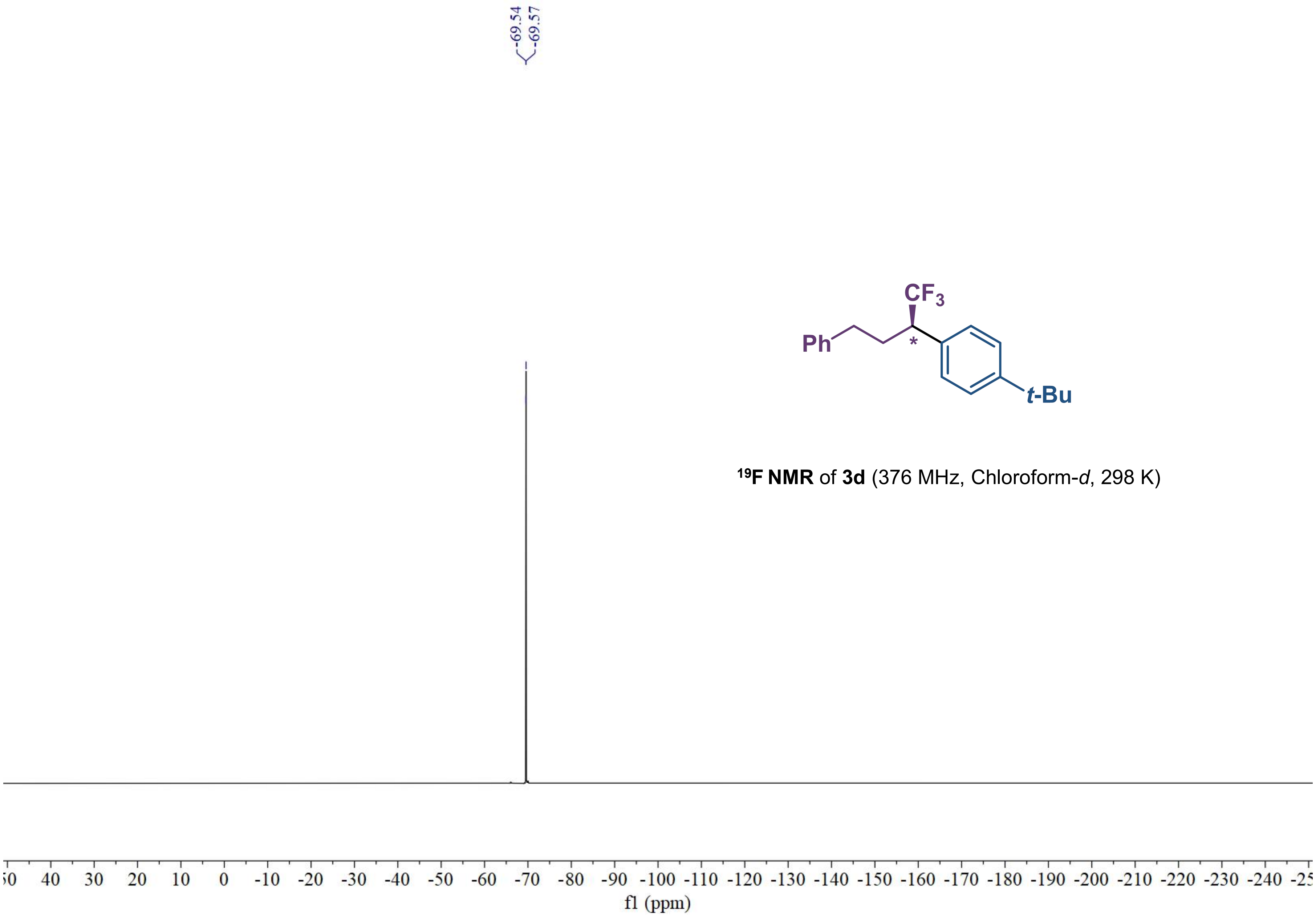

^{19}F NMR of **3d** (376 MHz, Chloroform-*d*, 298 K)

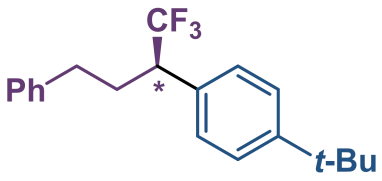

^{13}C NMR of **3d** (101 MHz, Chloroform-*d*, 298 K)

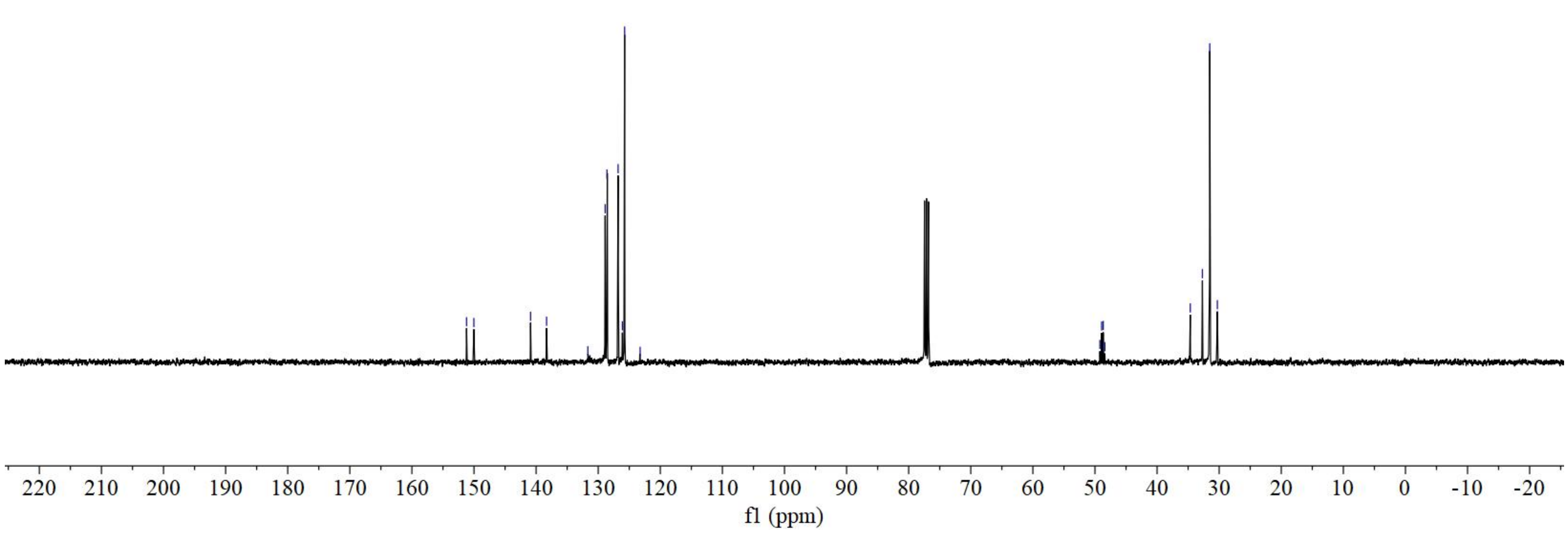

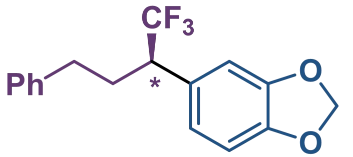

^{1}**H NMR** of **3e** (400 MHz, Chloroform-*d*, 298 K)

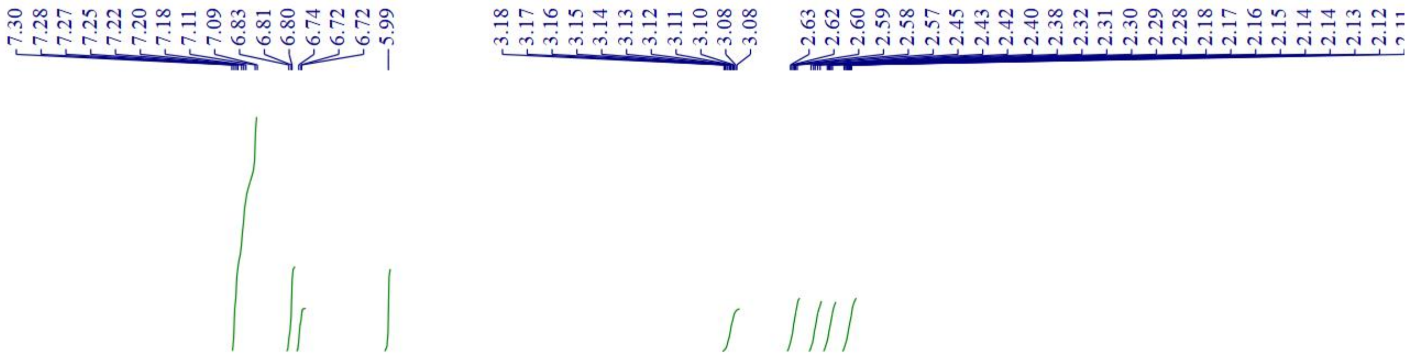

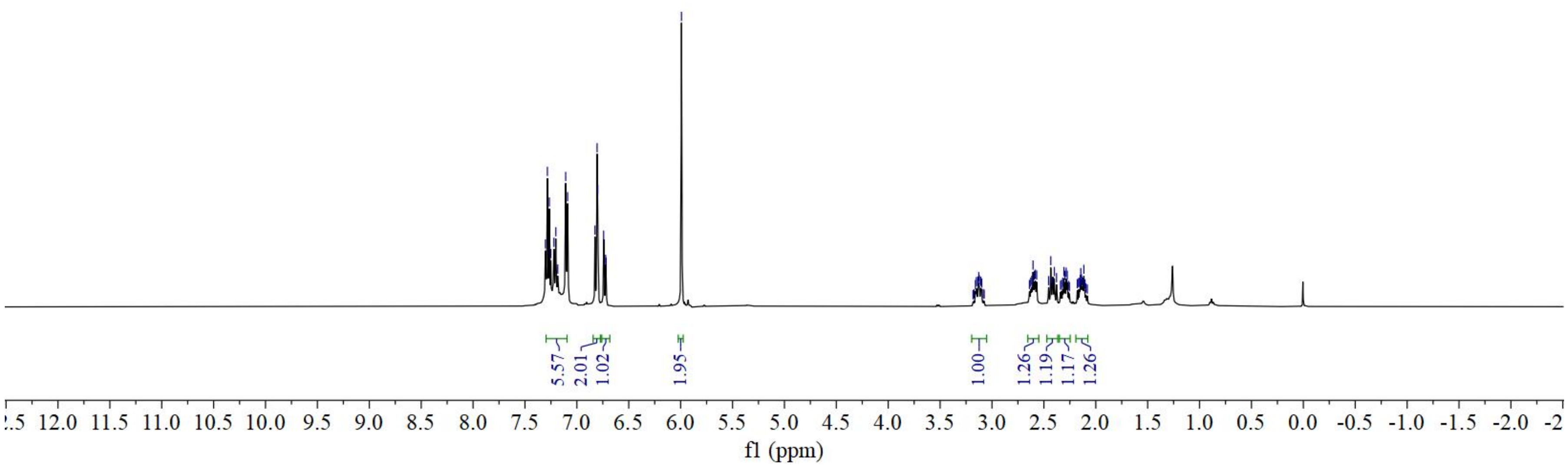

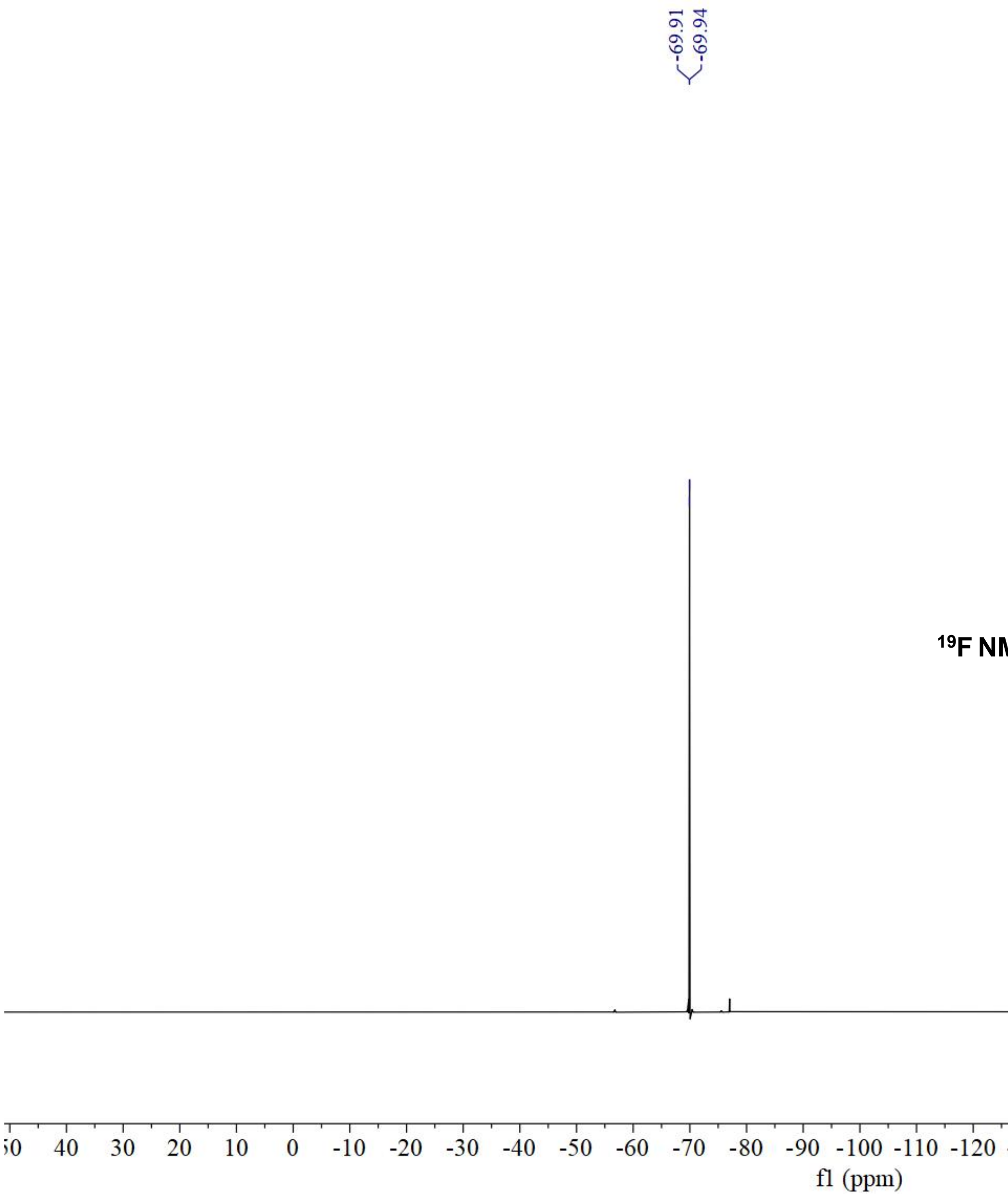

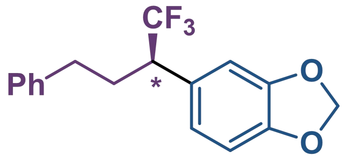

^{19}**F NMR** of **3e** (376 MHz, Chloroform-*d*, 298 K)

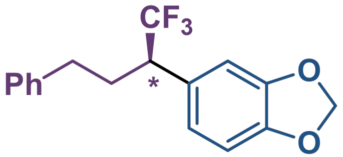

^{13}C **NMR** of **3e** (101 MHz, Chloroform-*d*, 298 K)

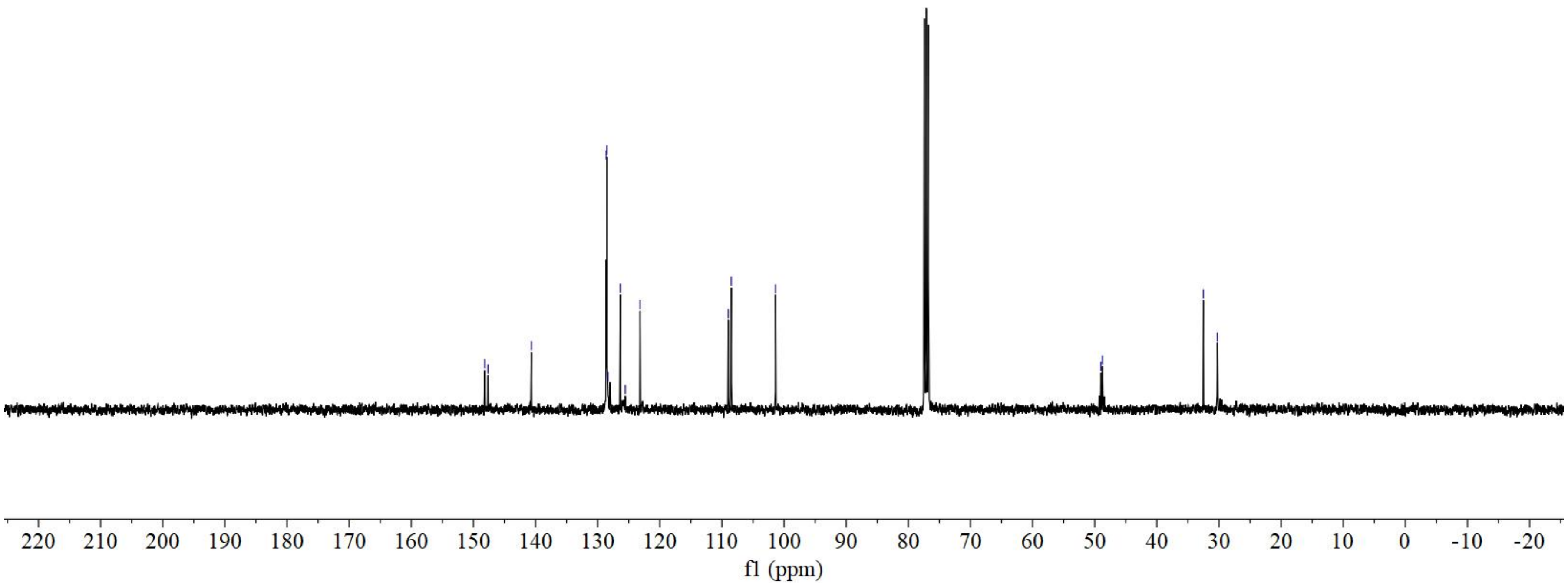

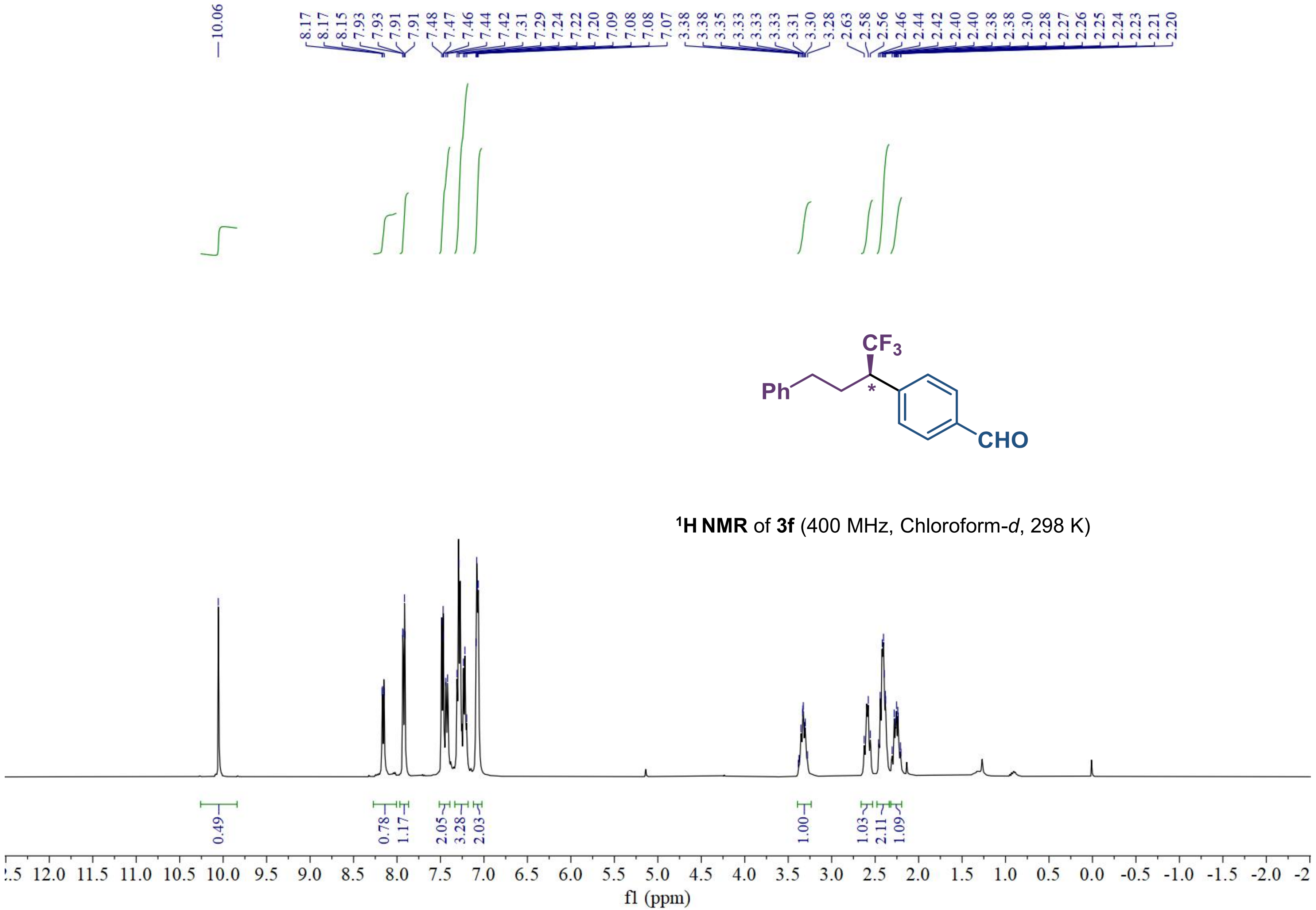

¹H NMR of **3f** (400 MHz, Chloroform-*d*, 298 K)

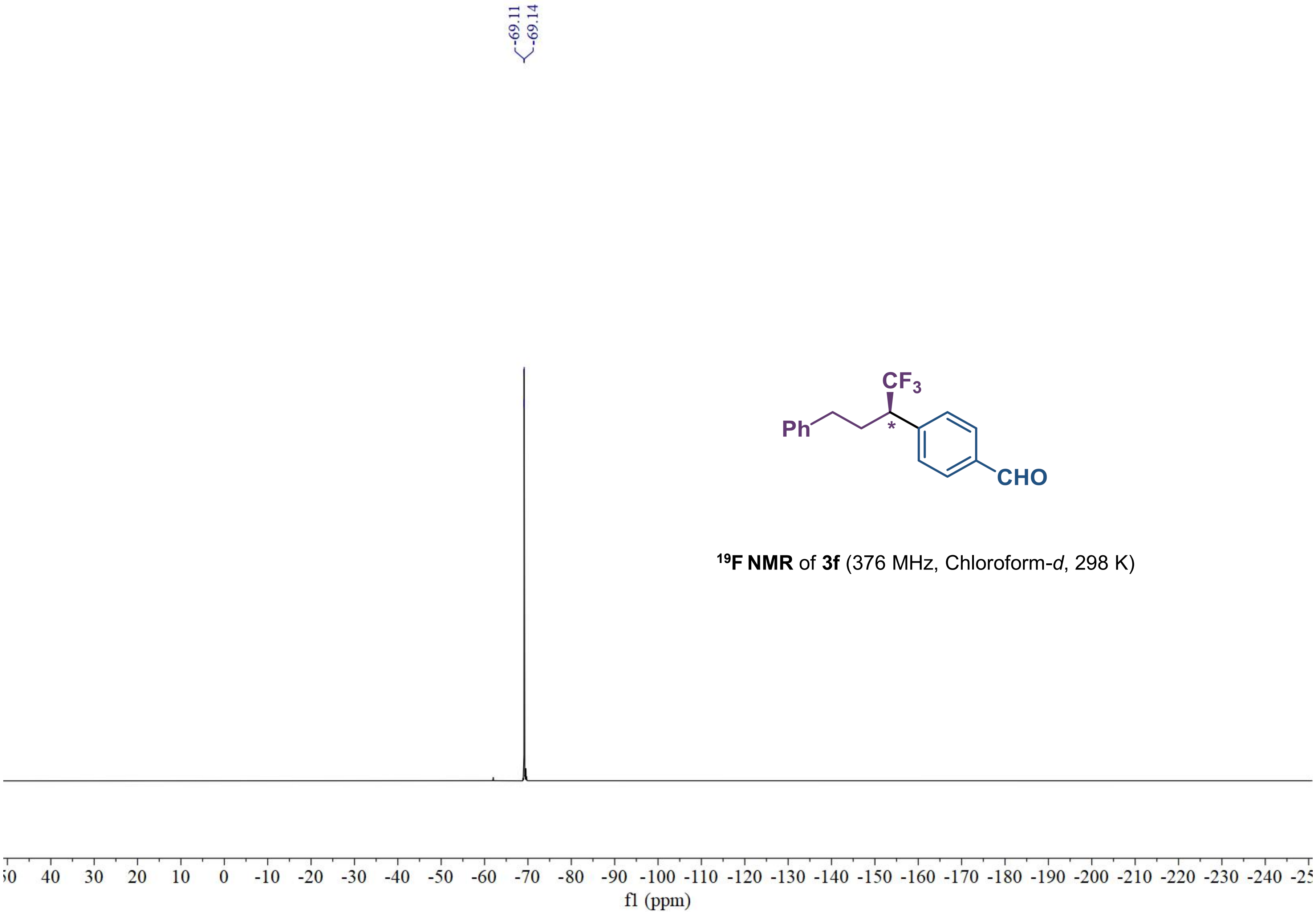

^{19}F NMR of **3f** (376 MHz, Chloroform-*d*, 298 K)

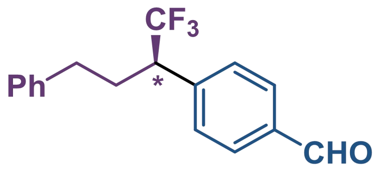

^{13}**C NMR** of **3f** (101 MHz, Chloroform-*d*, 298 K)

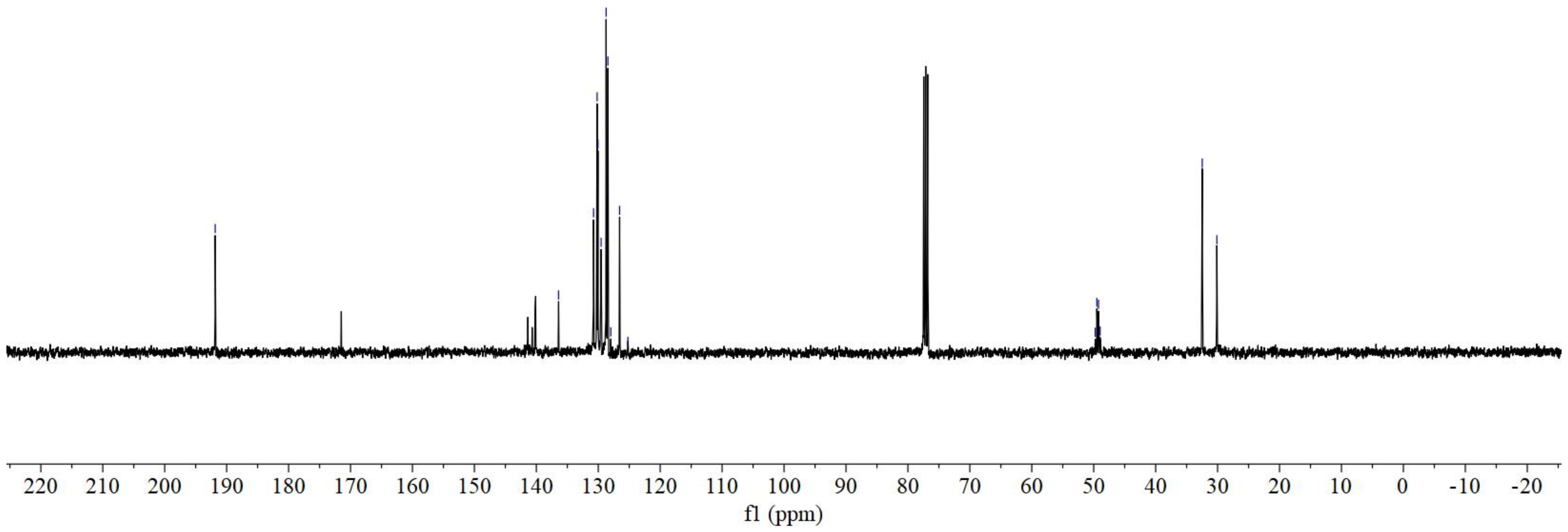

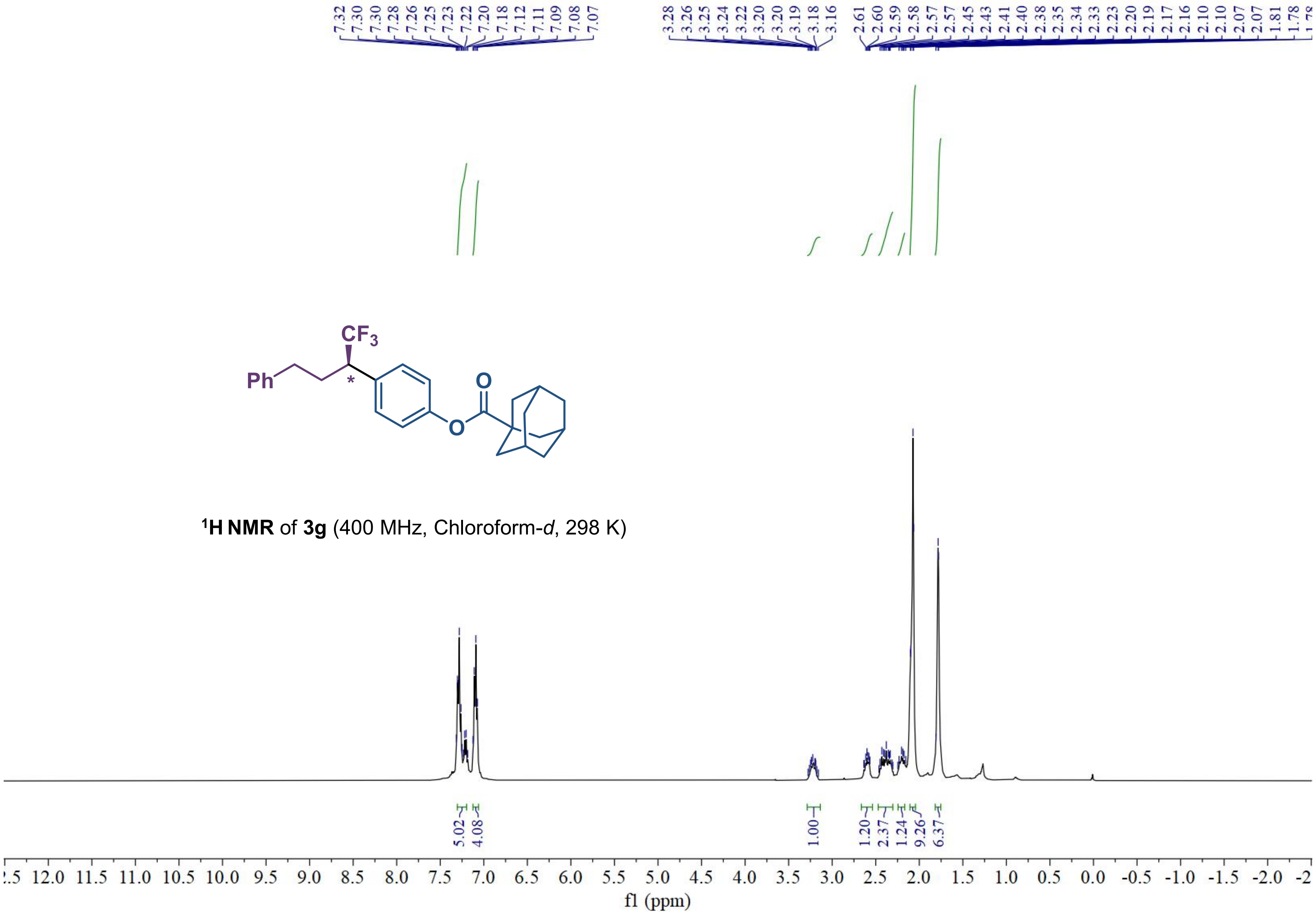

^{1}**H NMR** of **3g** (400 MHz, Chloroform-*d*, 298 K)

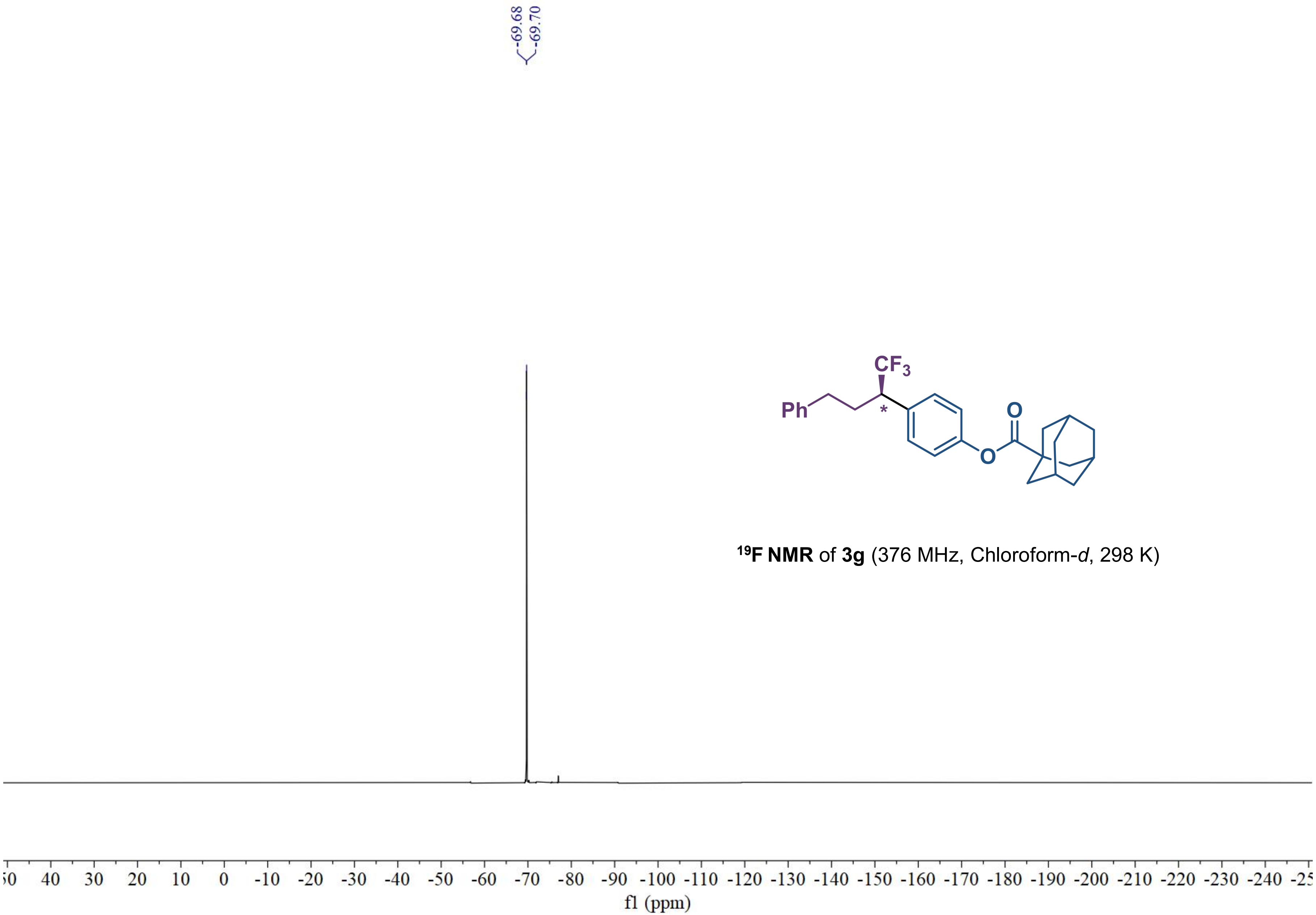

^{19}F NMR of **3g** (376 MHz, Chloroform-*d*, 298 K)

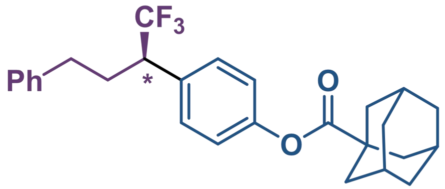

^{13}C NMR of **3g** (101 MHz, Chloroform-*d*, 298 K)

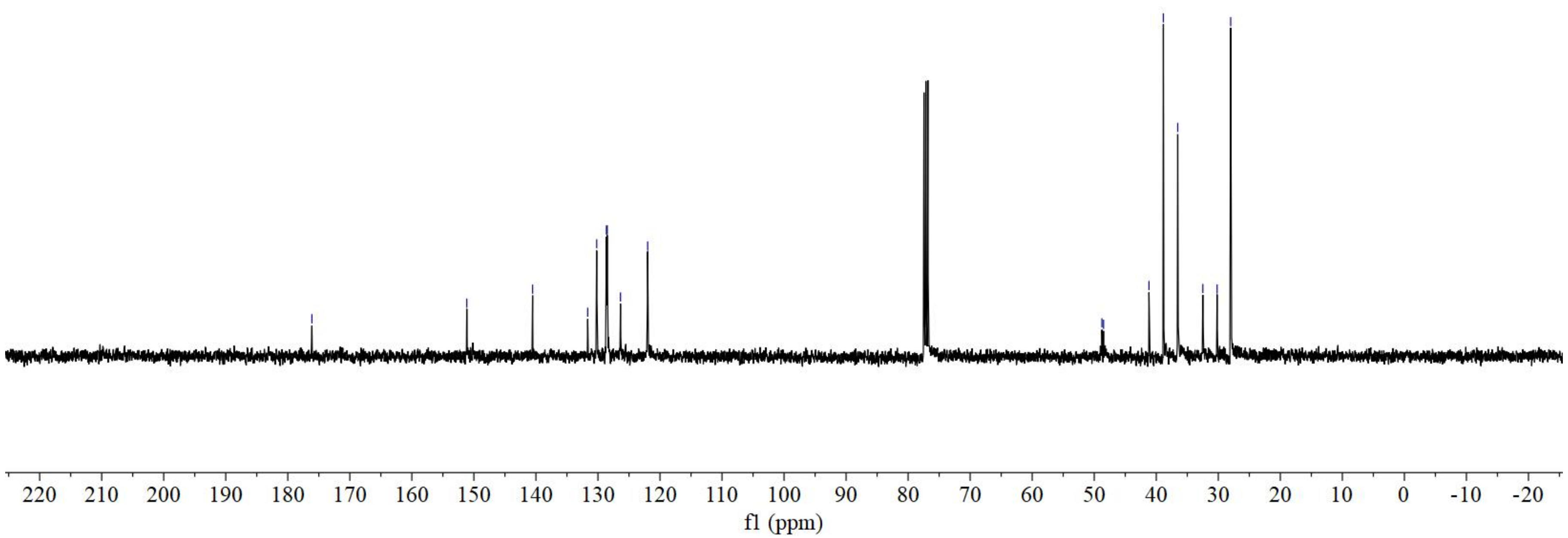

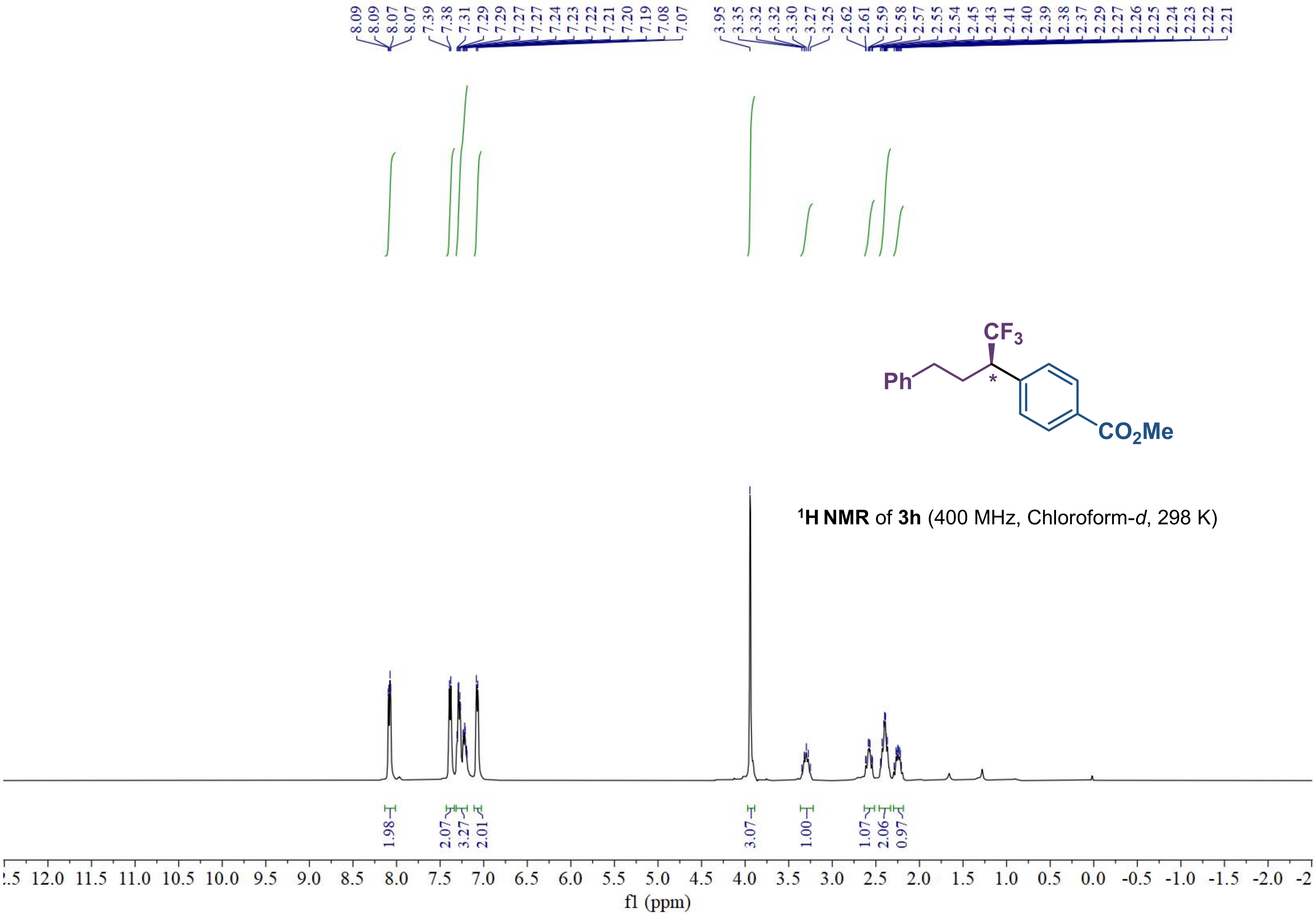

^{1}H NMR of **3h** (400 MHz, Chloroform-*d*, 298 K)

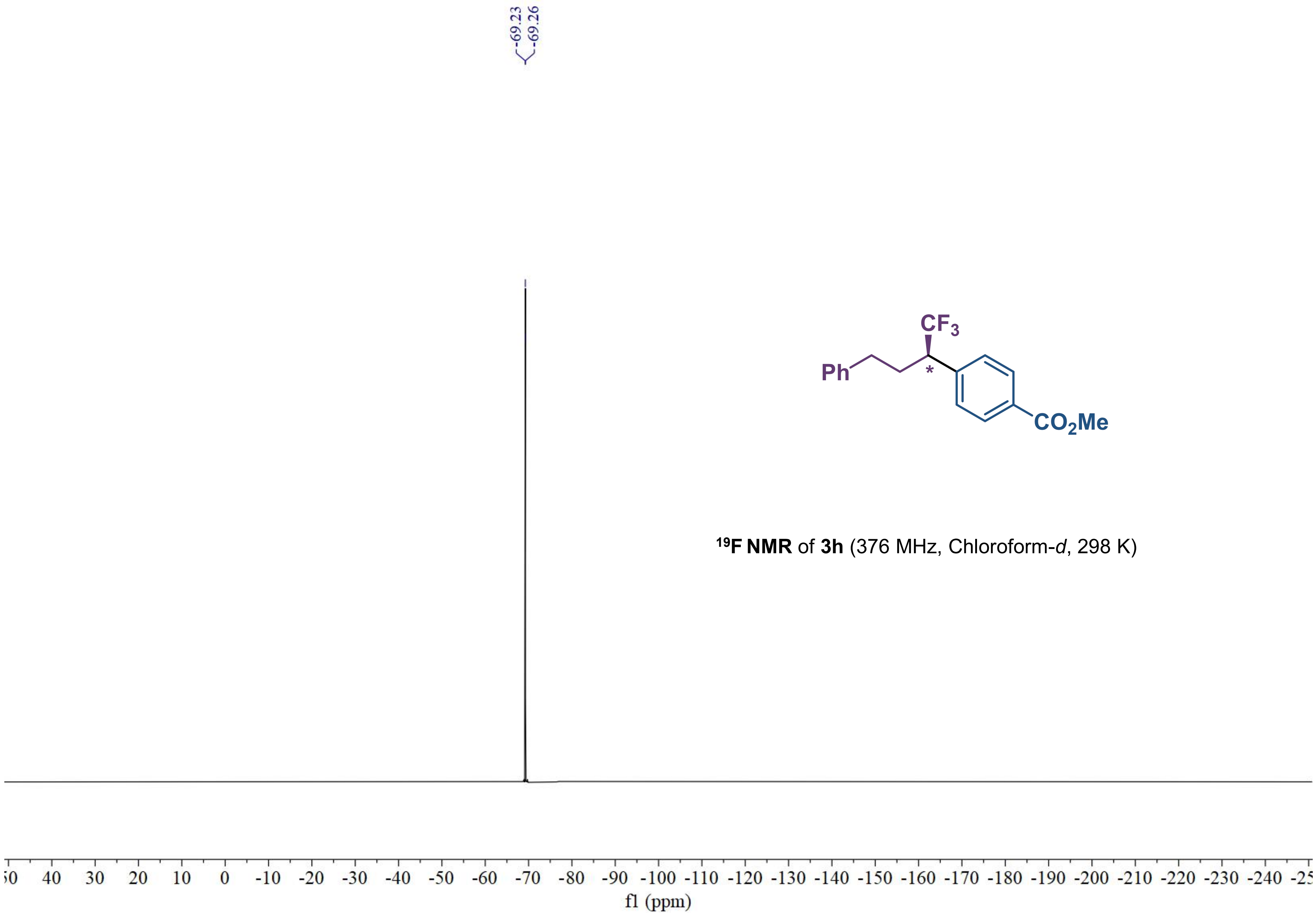

^{19}F NMR of **3h** (376 MHz, Chloroform-*d*, 298 K)

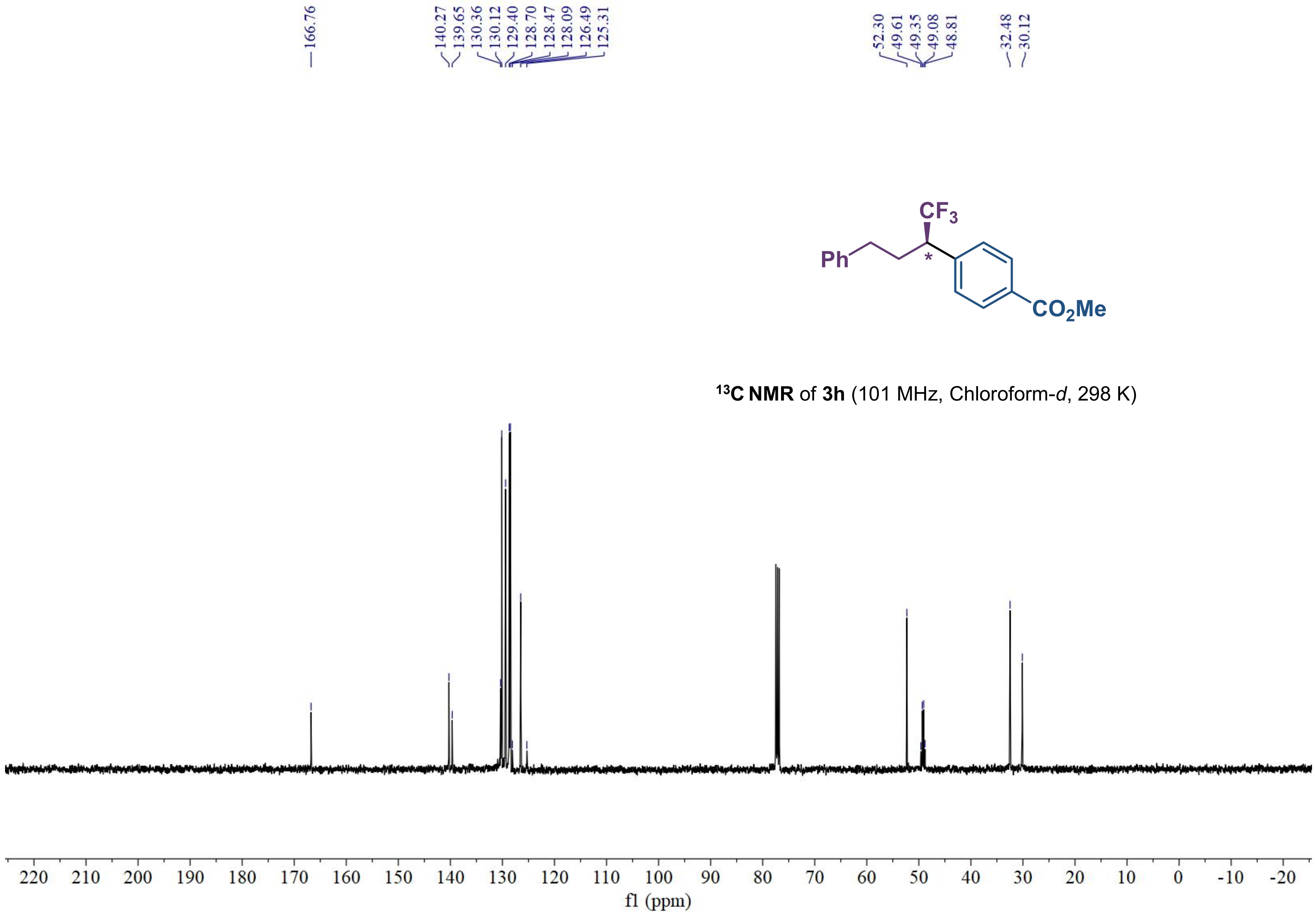

^{13}**C NMR** of **3h** (101 MHz, Chloroform-*d*, 298 K)

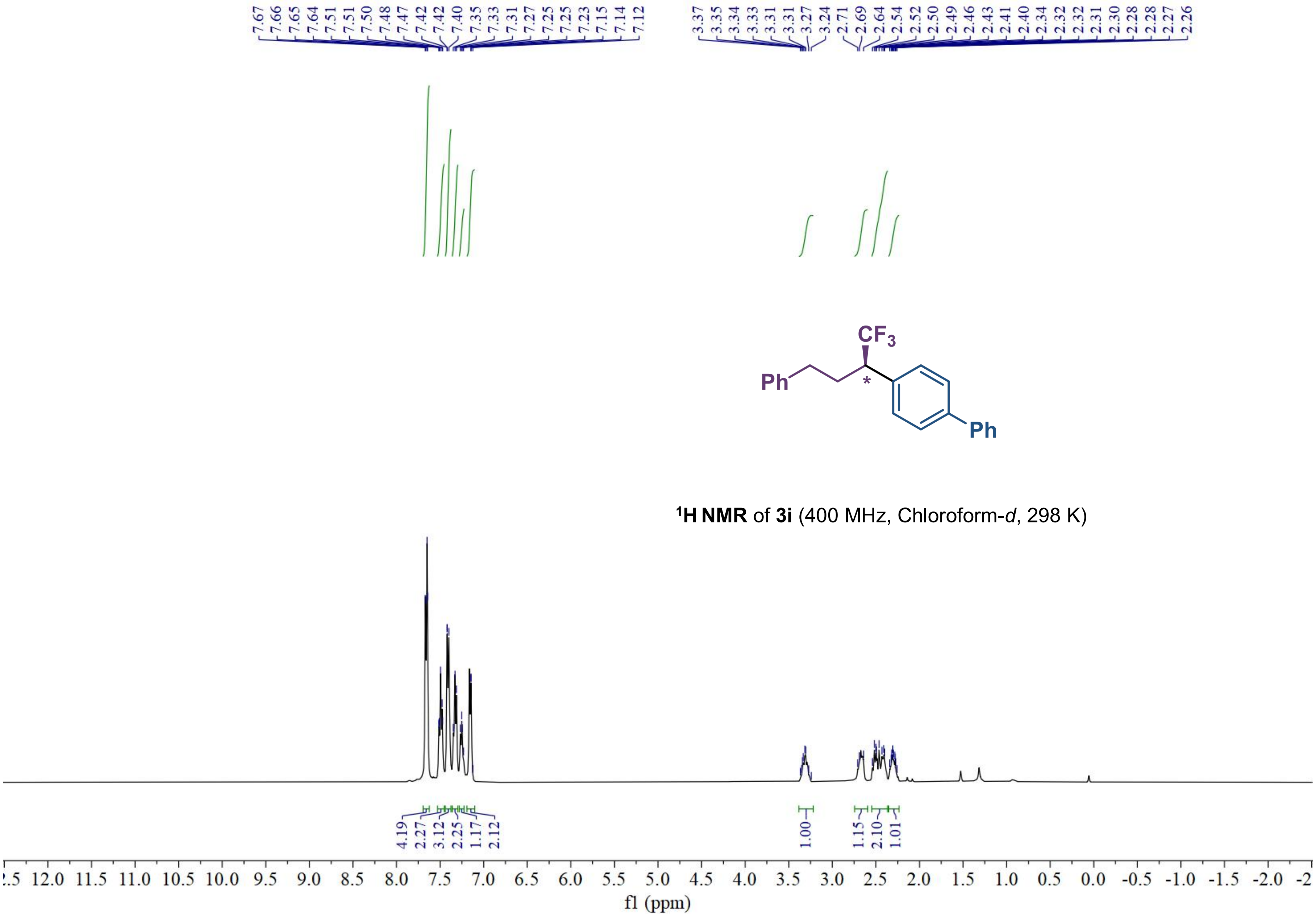

^{1}H NMR of **3i** (400 MHz, Chloroform-*d*, 298 K)

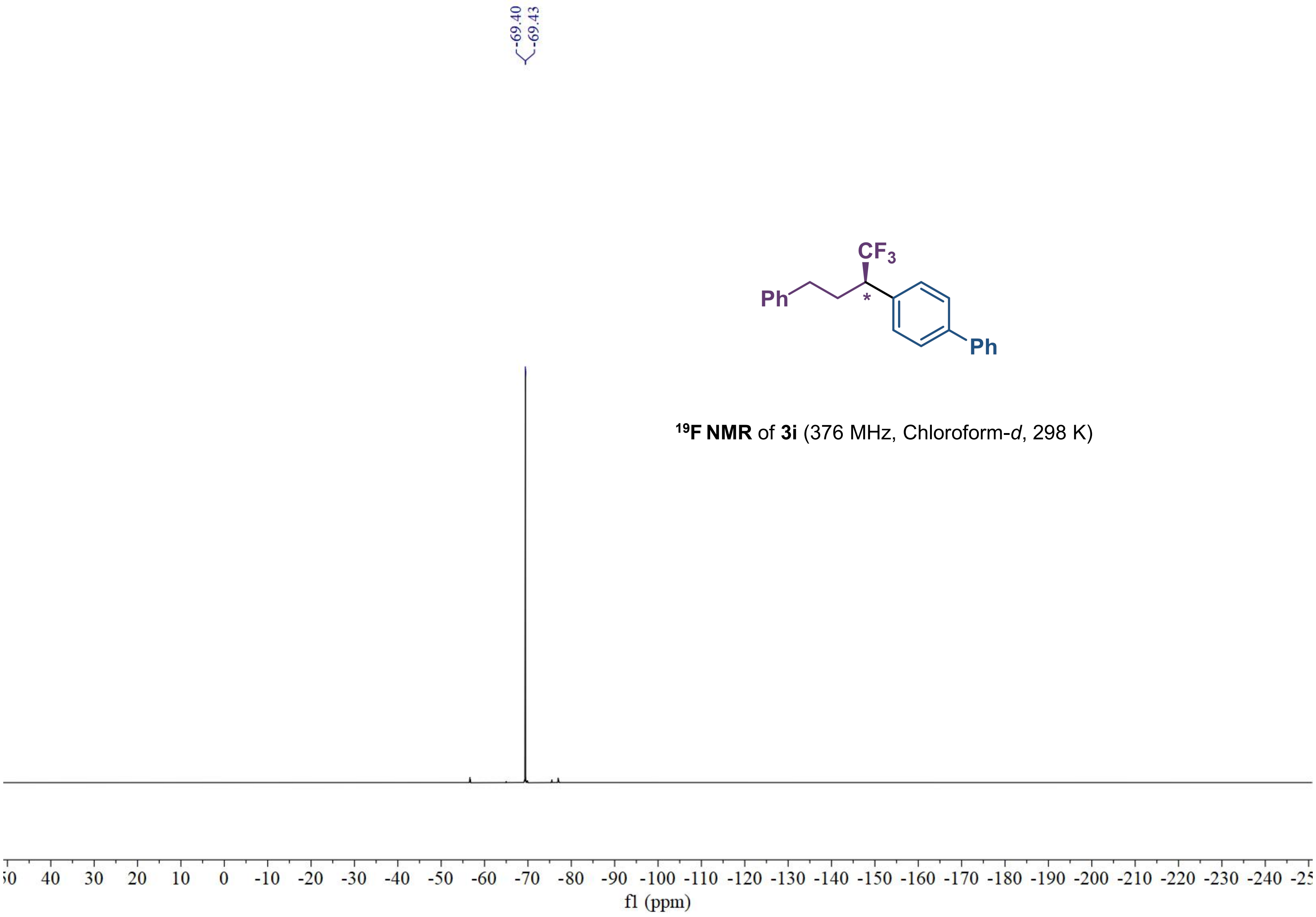

^{19}F NMR of **3i** (376 MHz, Chloroform-*d*, 298 K)

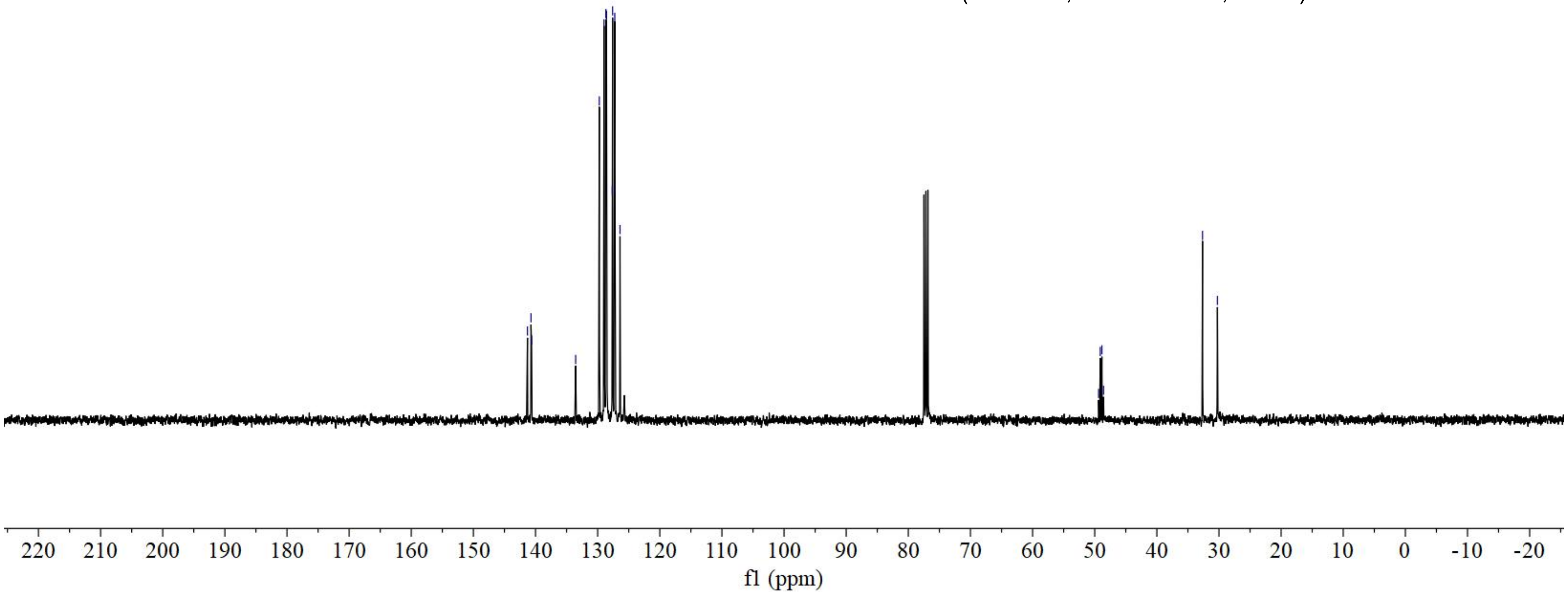

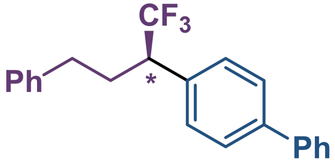

^{13}C NMR of **3i** (101 MHz, Chloroform-*d*, 298 K)

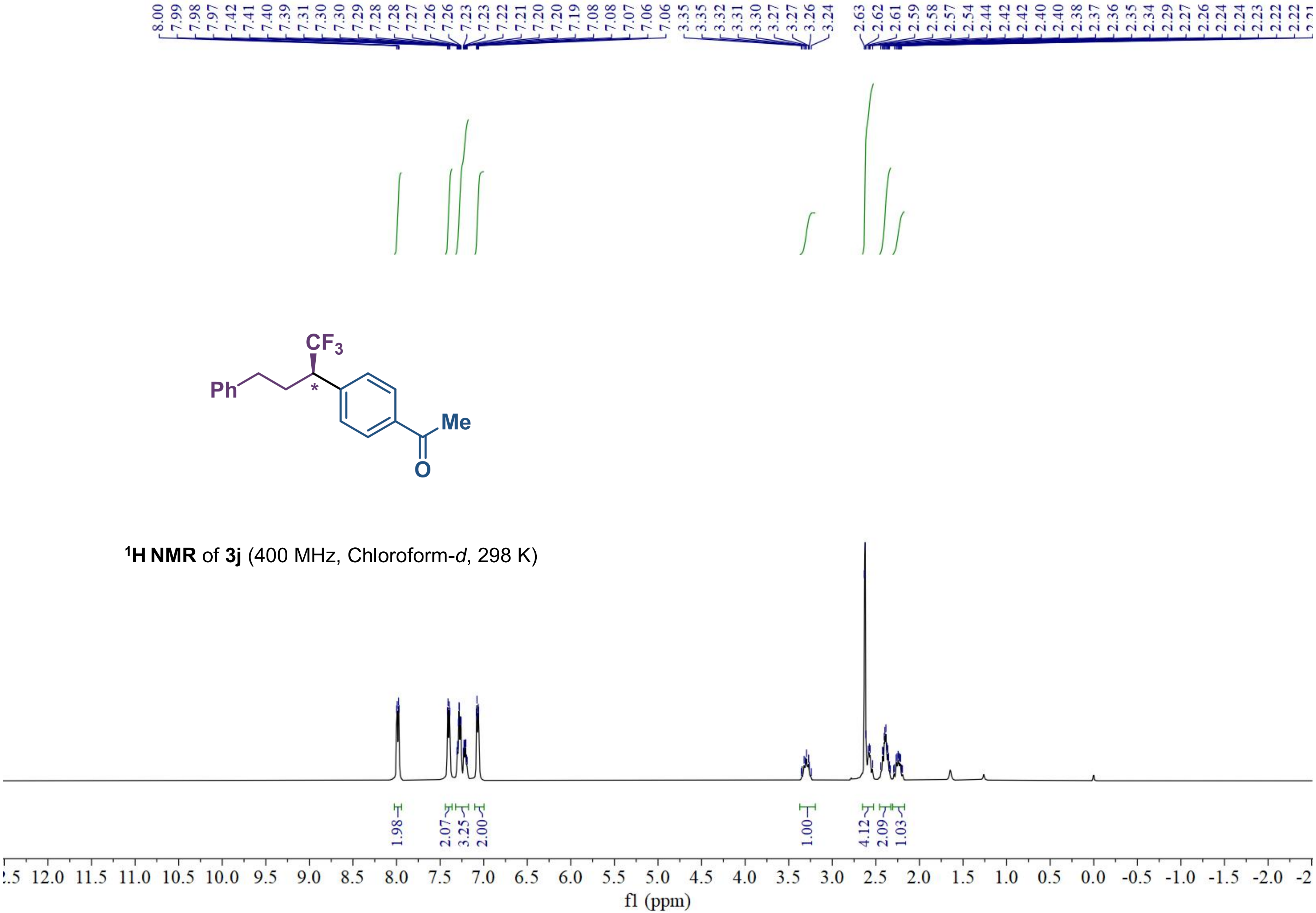

^{1}H NMR of **3j** (400 MHz, Chloroform-*d*, 298 K)

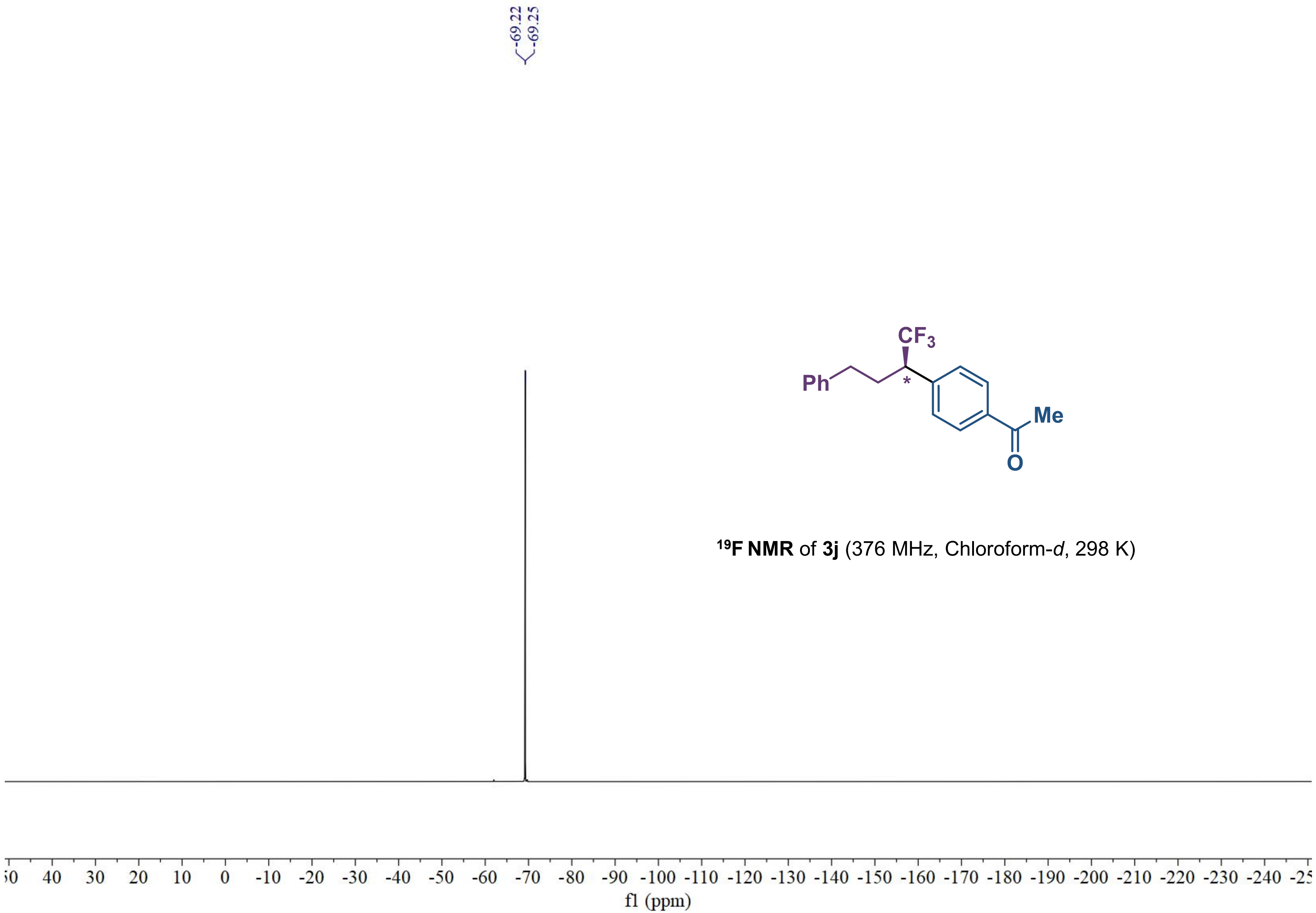

^{19}F NMR of **3j** (376 MHz, Chloroform-*d*, 298 K)

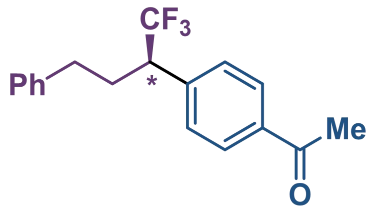

^{13}**C NMR** of **3j** (101 MHz, Chloroform-*d*, 298 K)

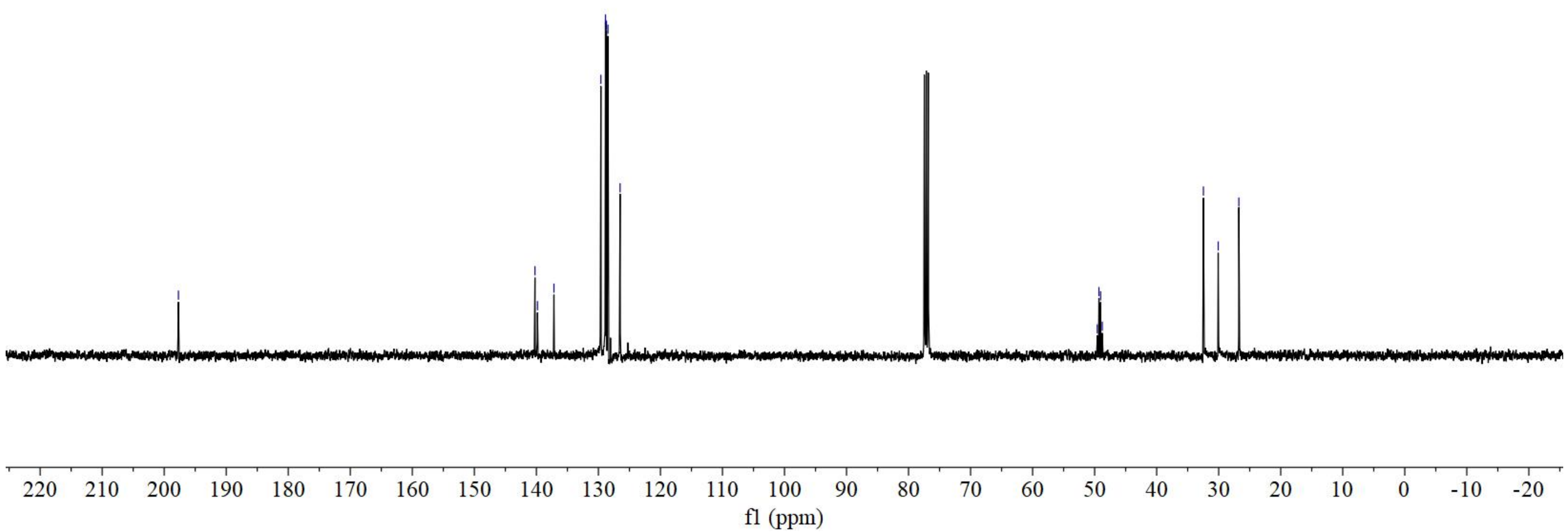

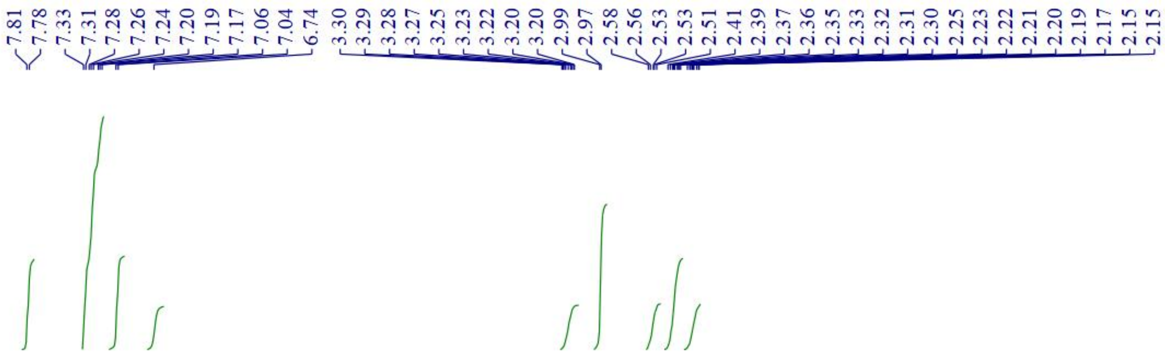

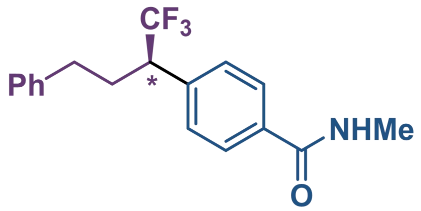

^{1}H NMR of **3k** (400 MHz, Chloroform-*d*, 298 K)

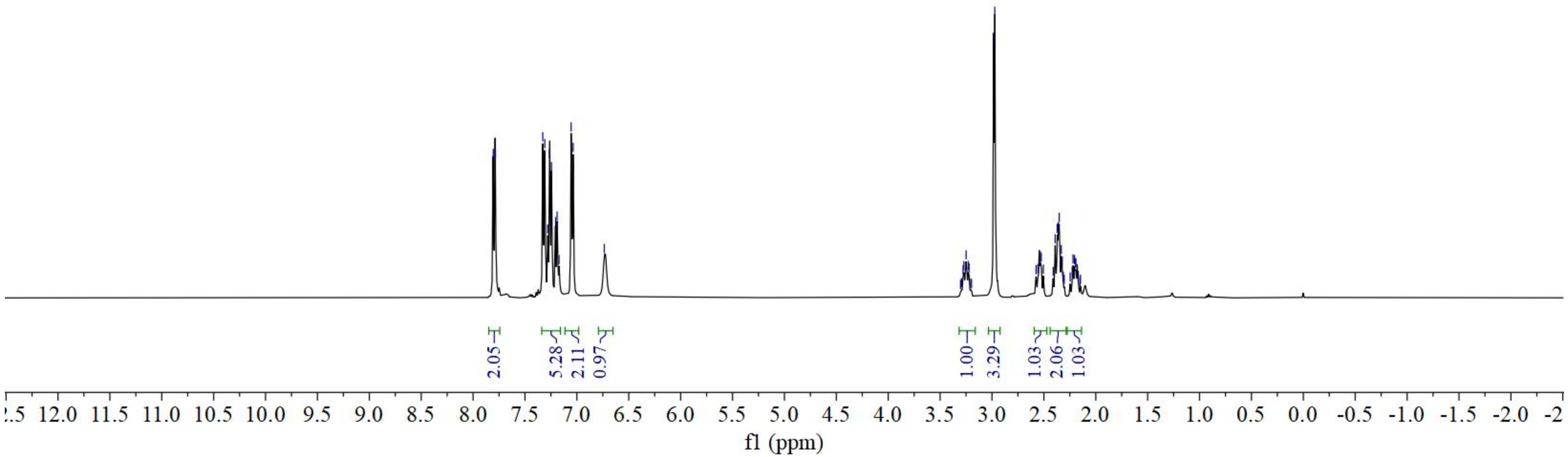

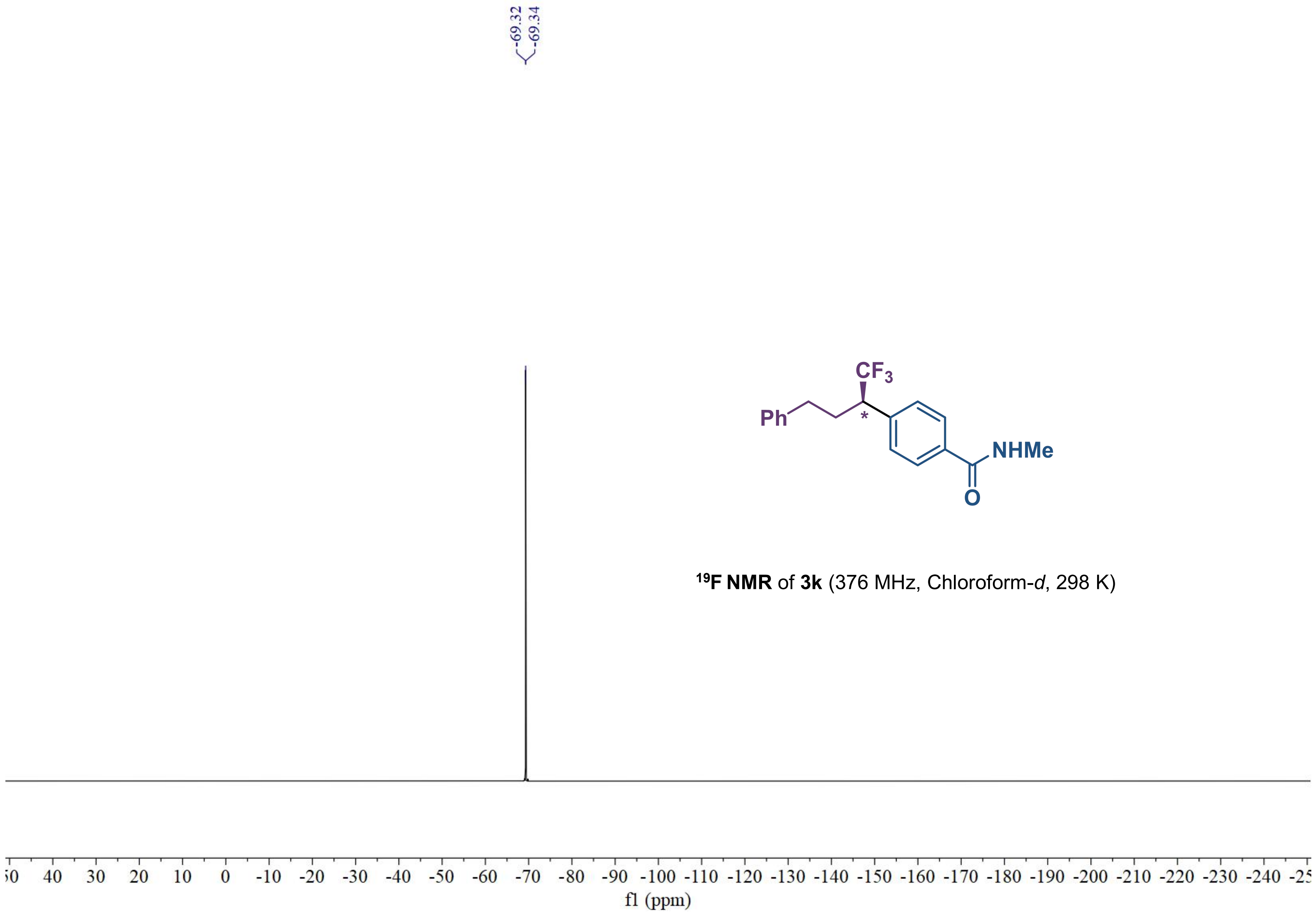

^{19}F NMR of **3k** (376 MHz, Chloroform-*d*, 298 K)

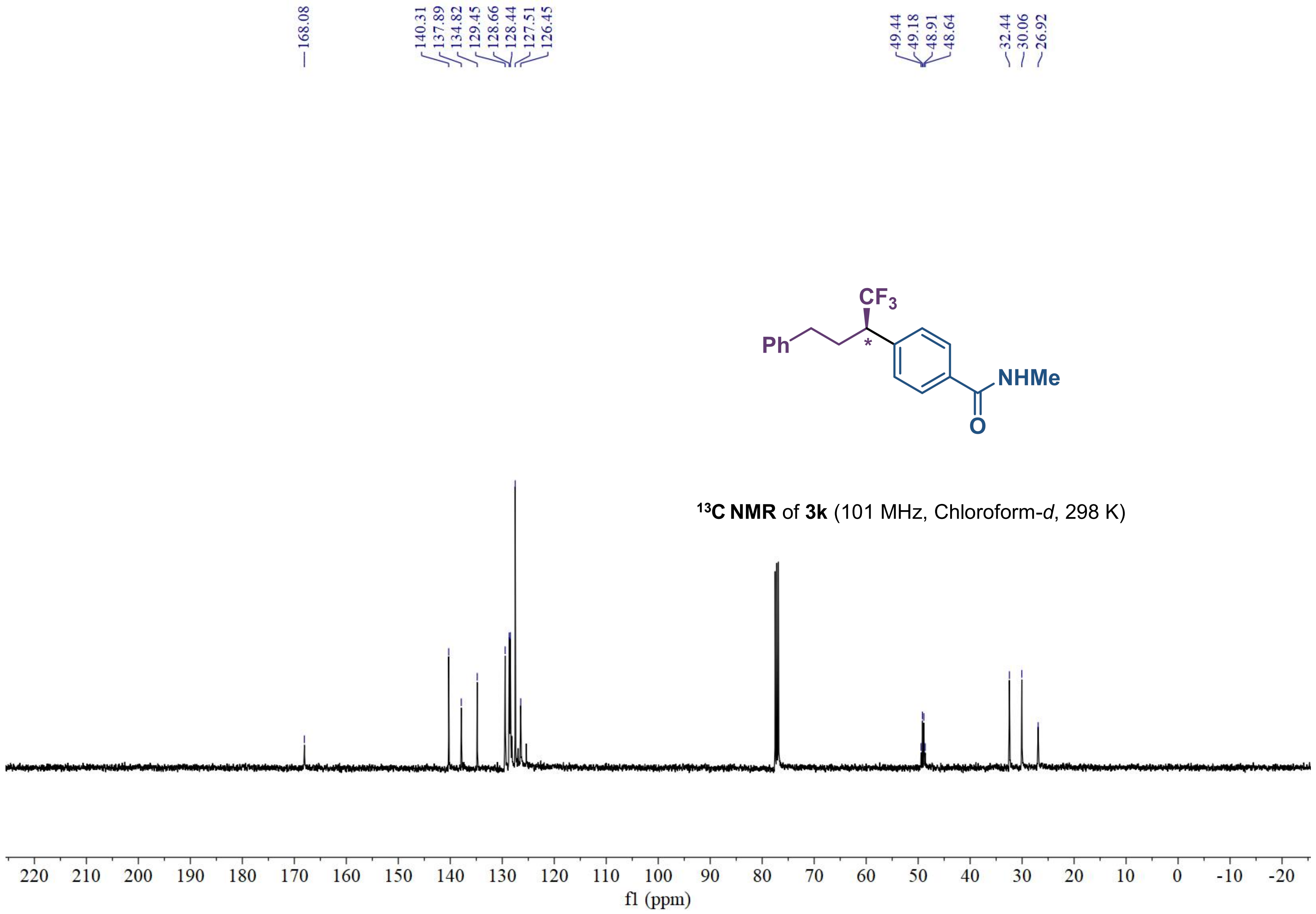

^{13}**C NMR** of **3k** (101 MHz, Chloroform-*d*, 298 K)

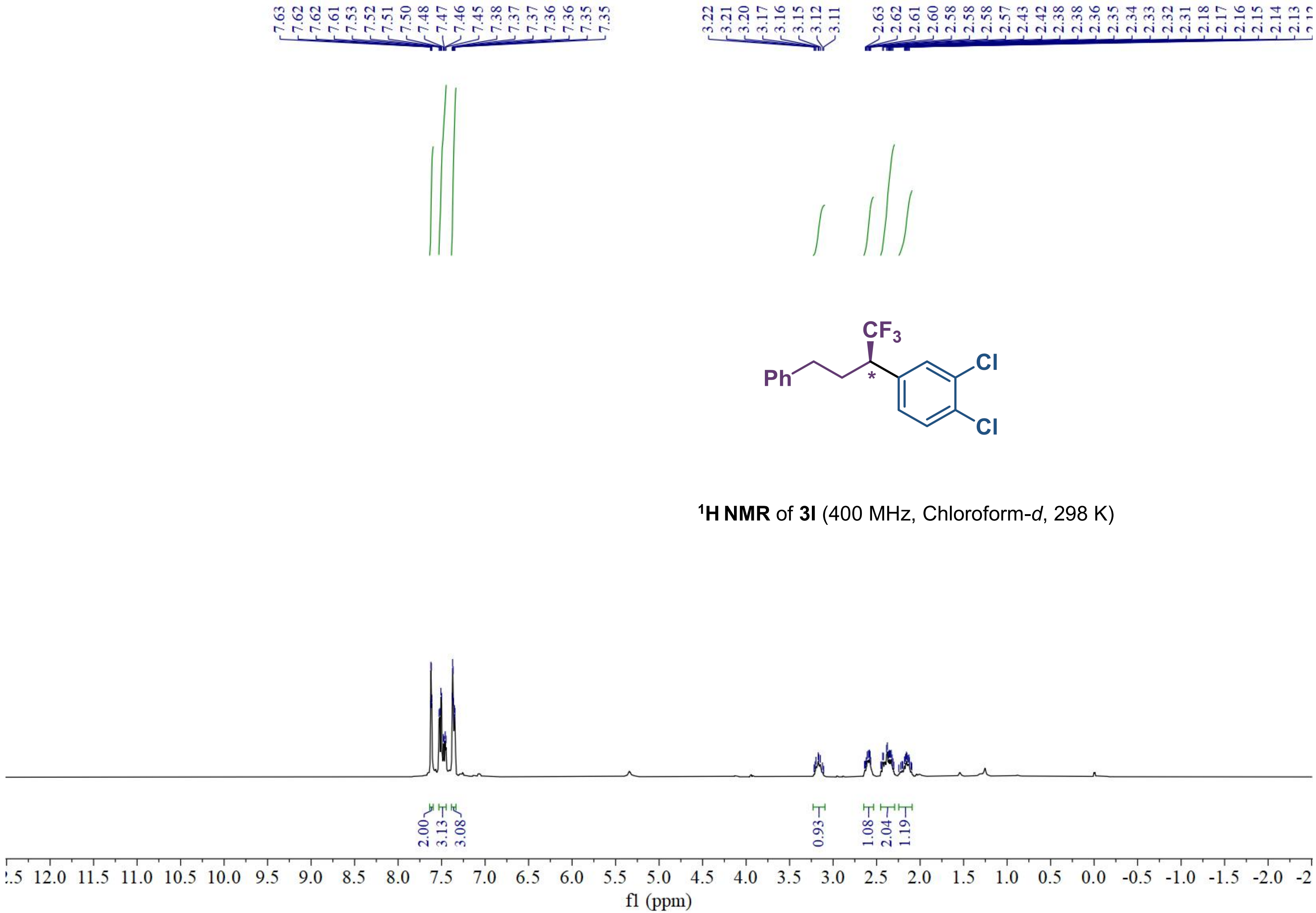

^{1}H NMR of **3l** (400 MHz, Chloroform-*d*, 298 K)

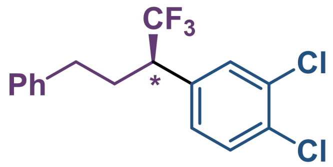

^{19}F NMR of **3l** (376 MHz, Chloroform-*d*, 298 K)

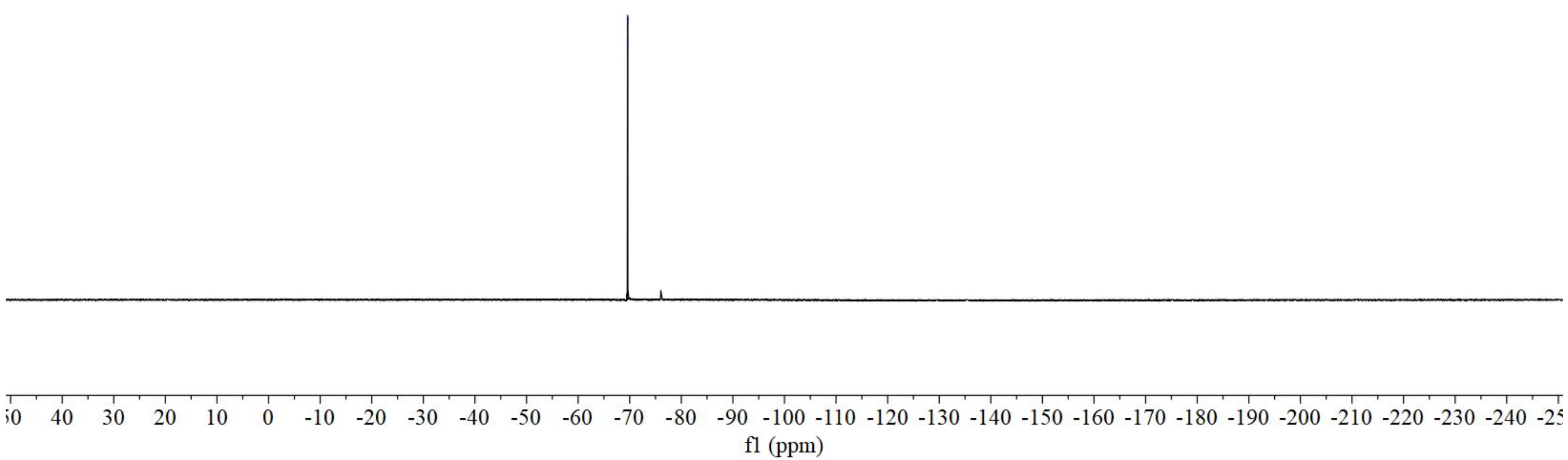

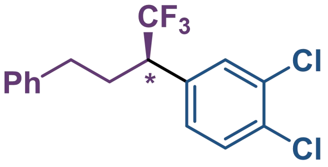

^{13}**C NMR** of **3l** (101 MHz, Chloroform-*d*, 298 K)

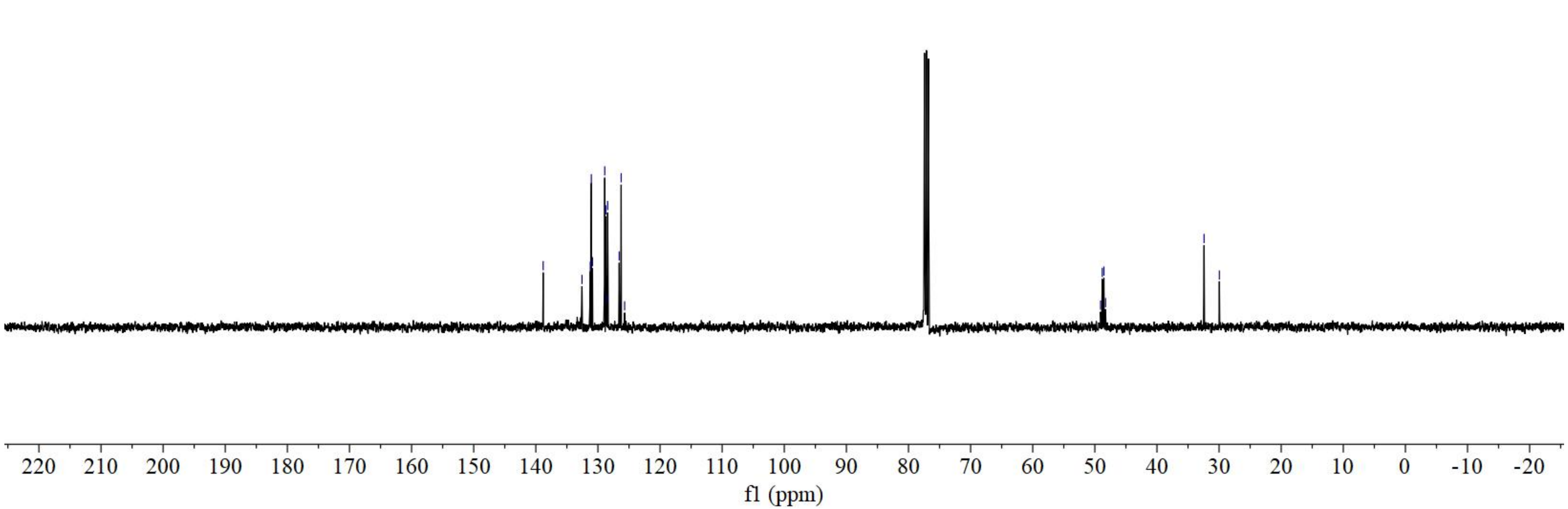

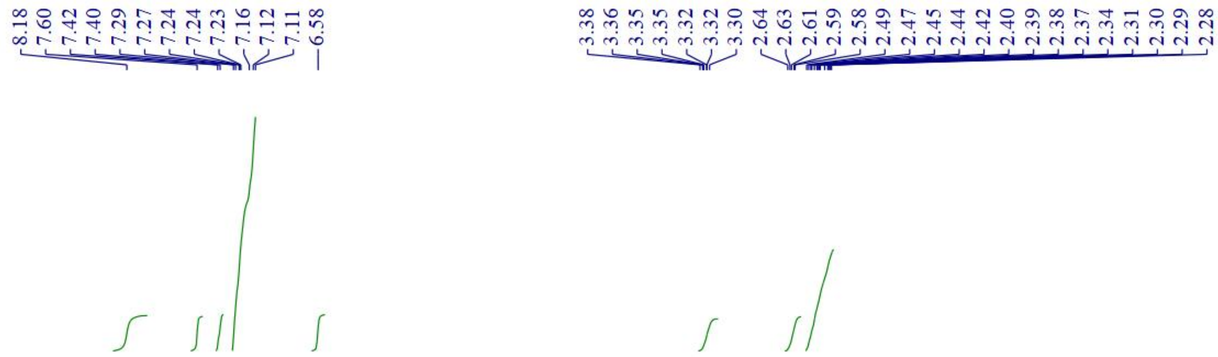

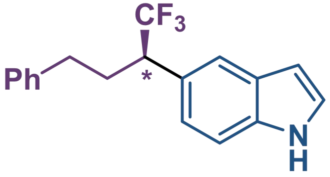

^{1}H NMR of **3m** (400 MHz, Chloroform-*d*, 298 K)

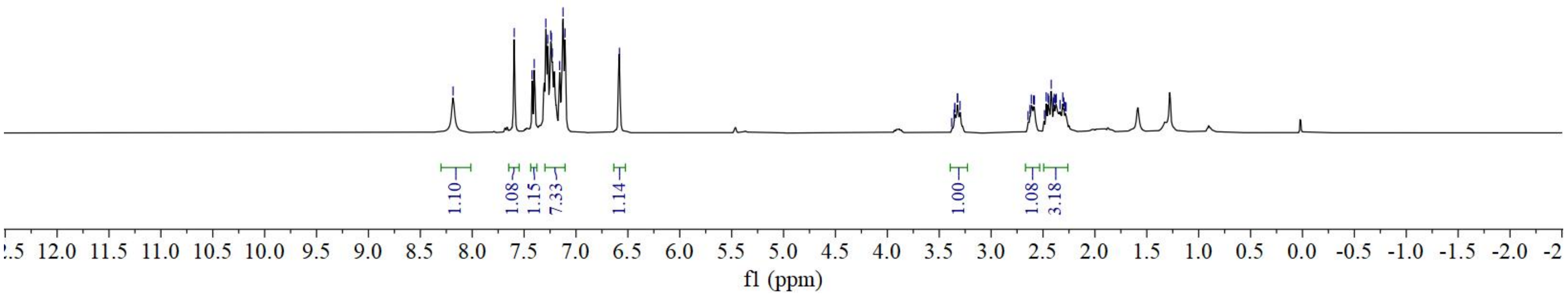

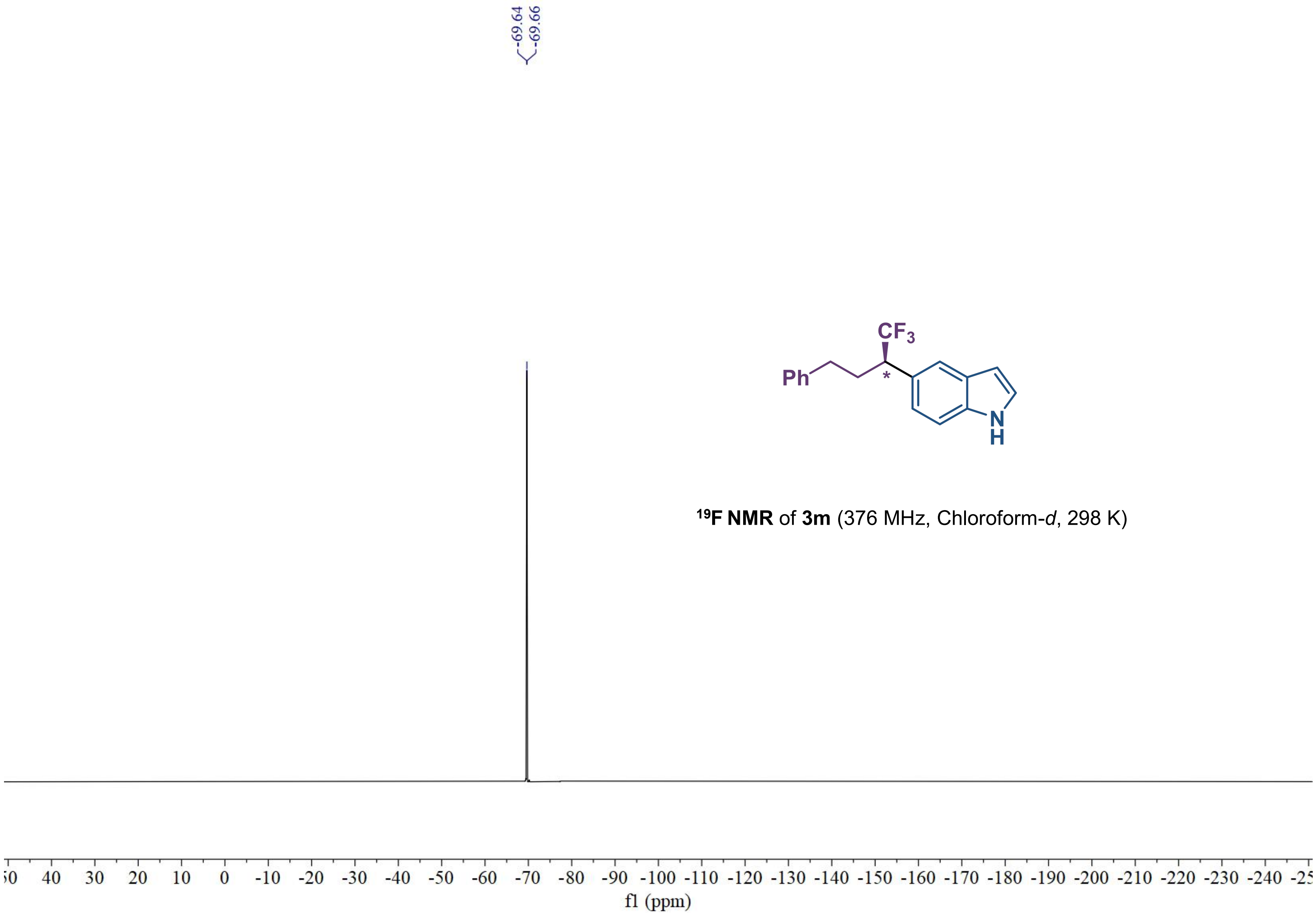

^{19}F NMR of **3m** (376 MHz, Chloroform-*d*, 298 K)

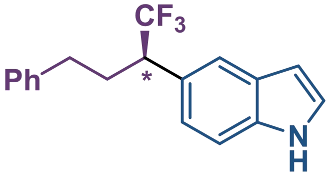

^{13}C NMR of **3m** (101 MHz, Chloroform-*d*, 298 K)

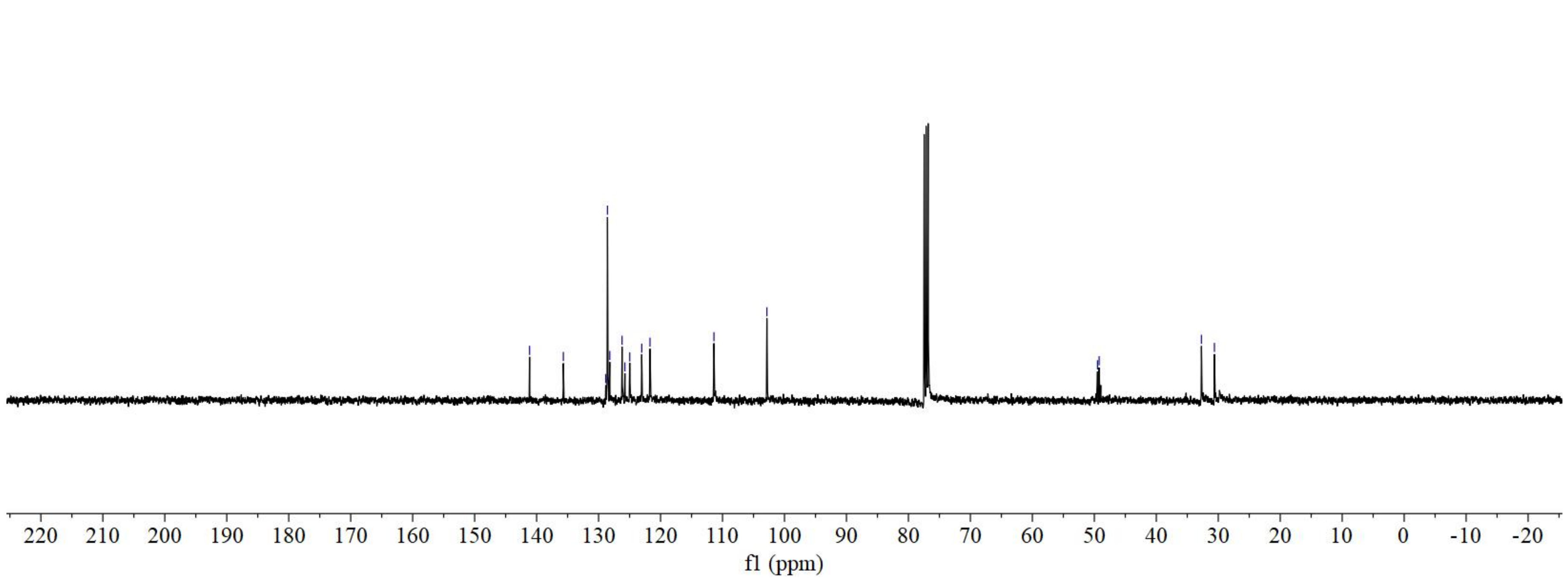

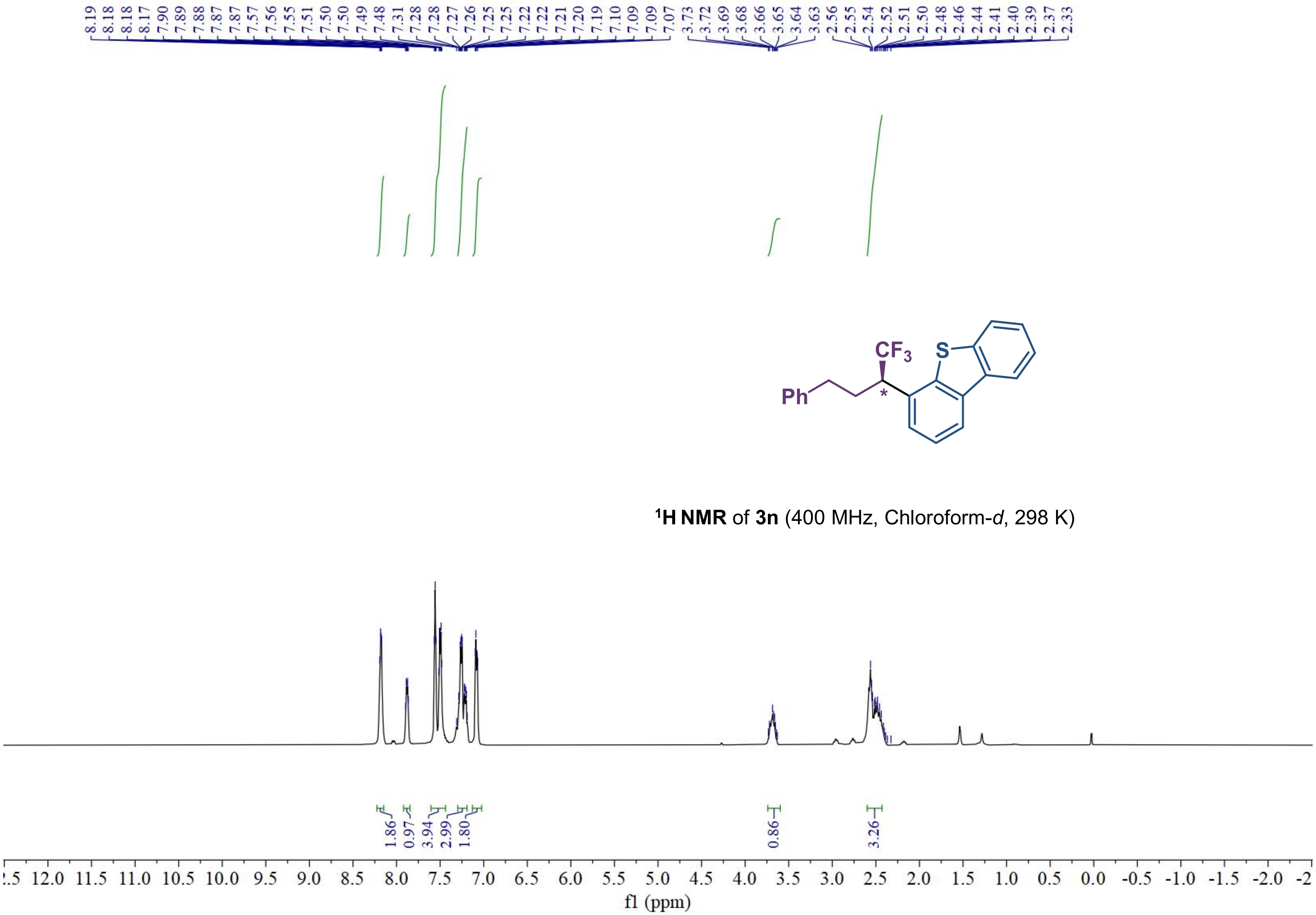

^{1}H NMR of **3n** (400 MHz, Chloroform-*d*, 298 K)

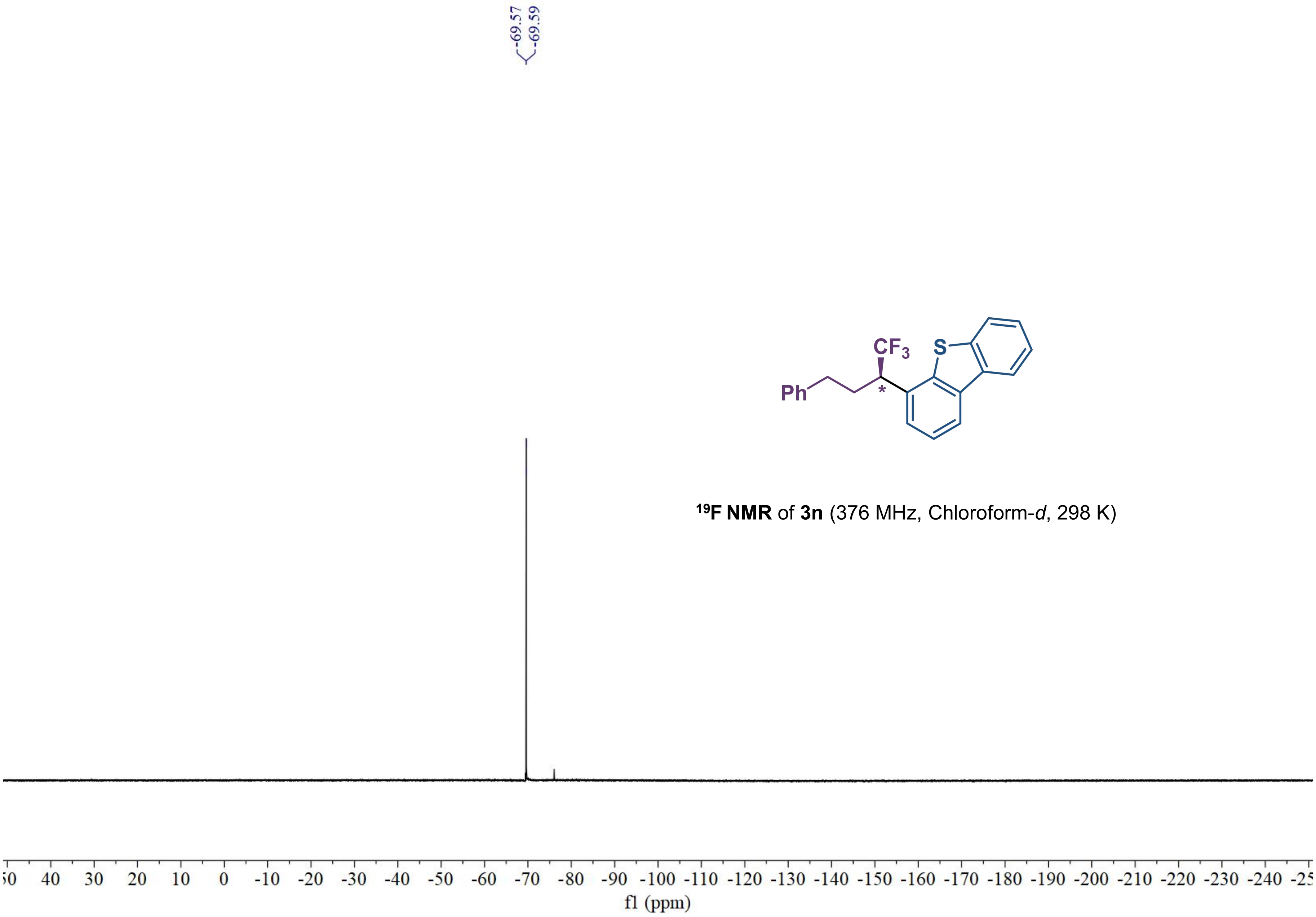

^{19}**F NMR** of **3n** (376 MHz, Chloroform-*d*, 298 K)

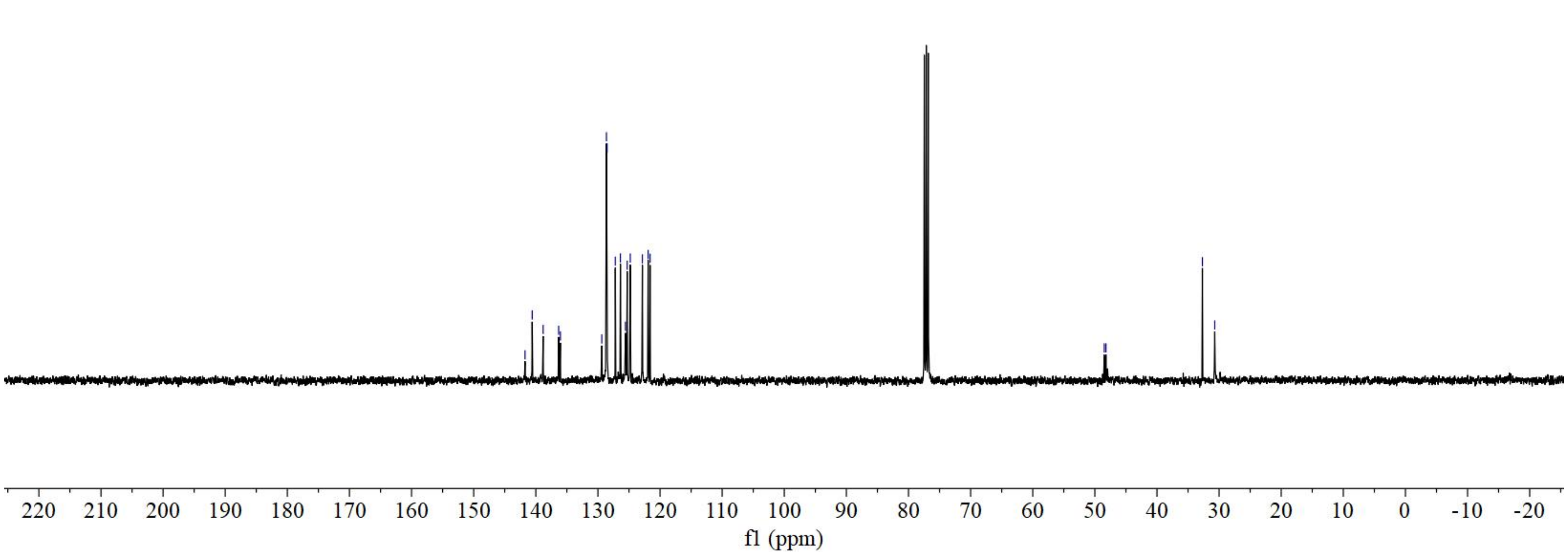

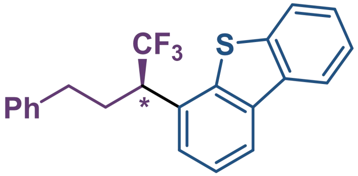

^{13}C NMR of **3n** (101 MHz, Chloroform-*d*, 298 K)

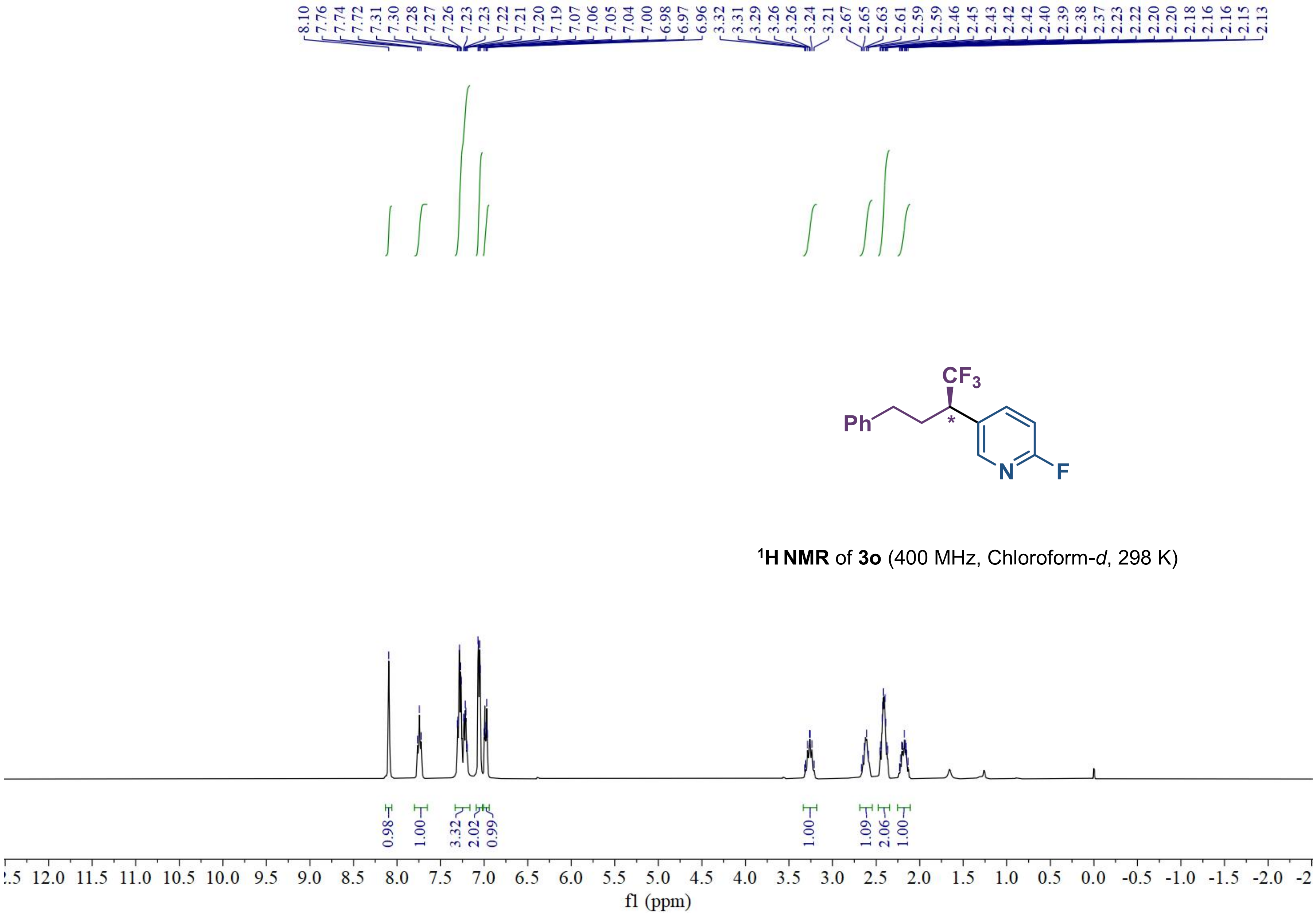

^{1}H NMR of **3o** (400 MHz, Chloroform-*d*, 298 K)

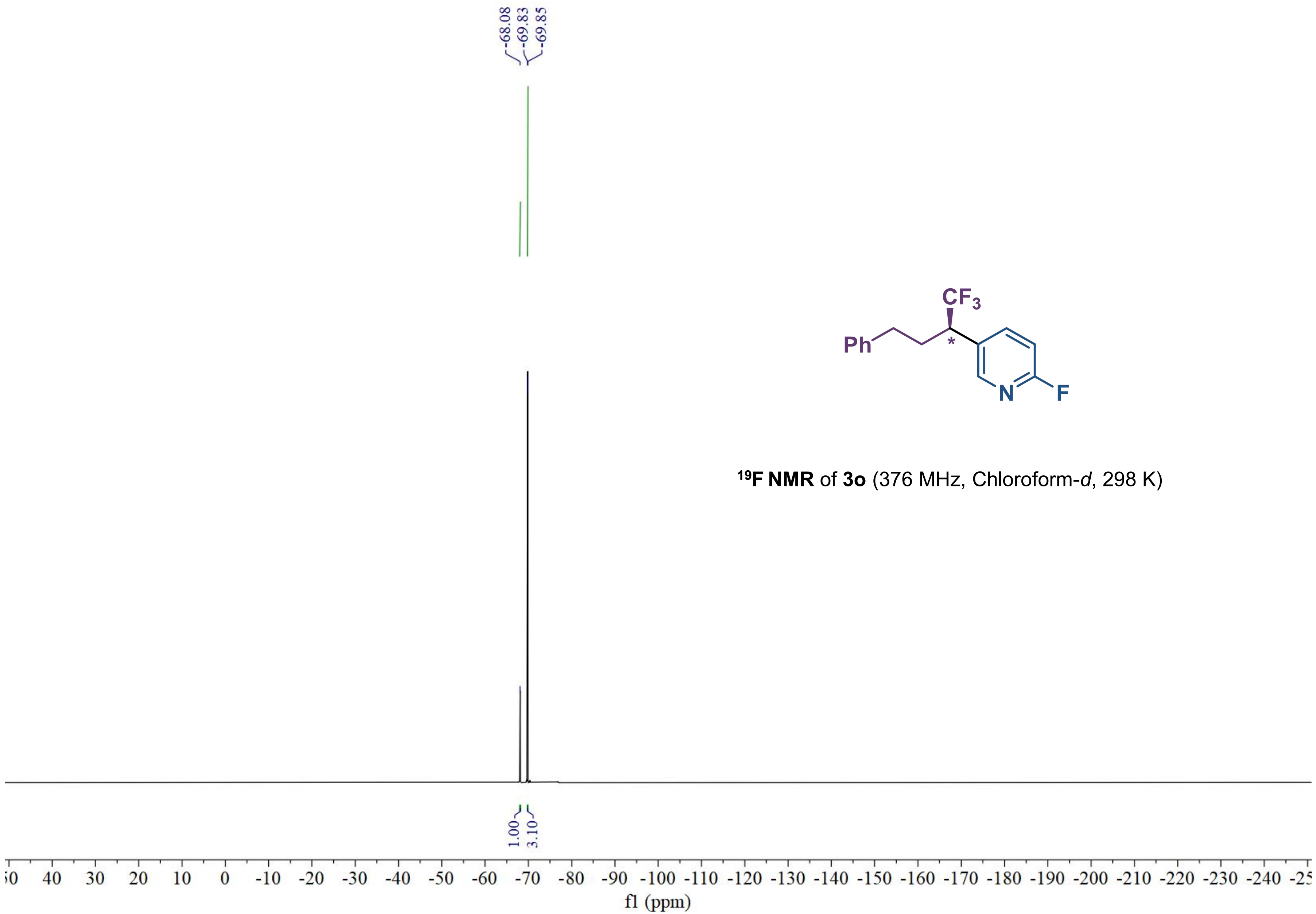

^{19}F NMR of **3o** (376 MHz, Chloroform-*d*, 298 K)

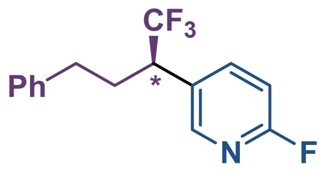

^{13}**C NMR** of **3o** (101 MHz, Chloroform-*d*, 298 K)

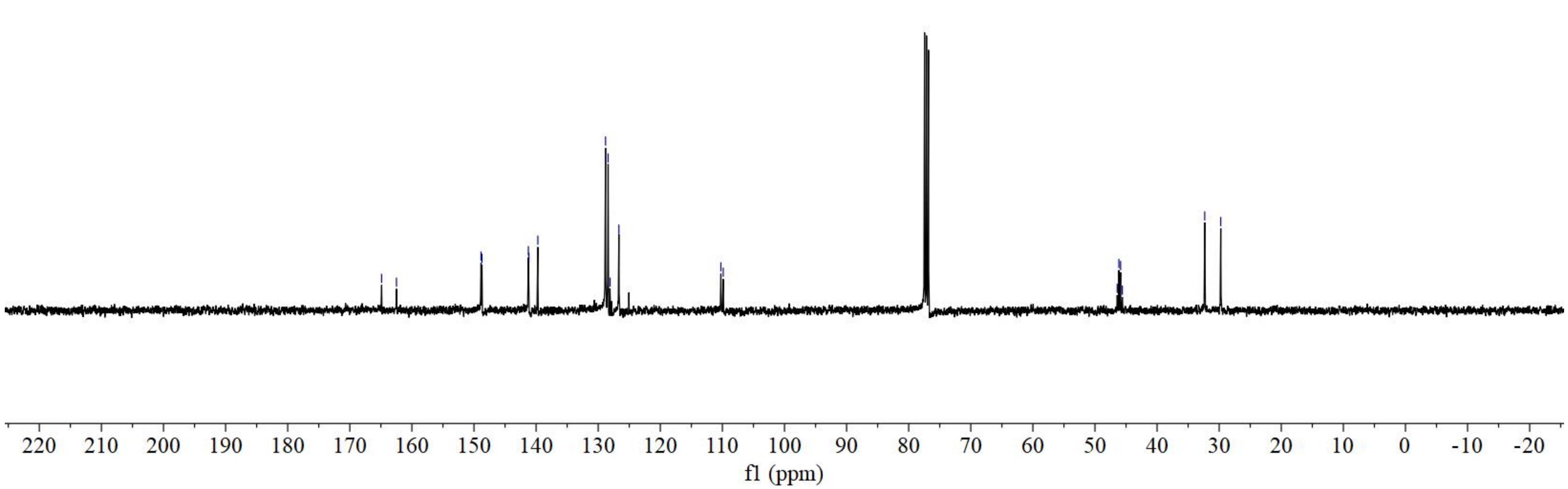

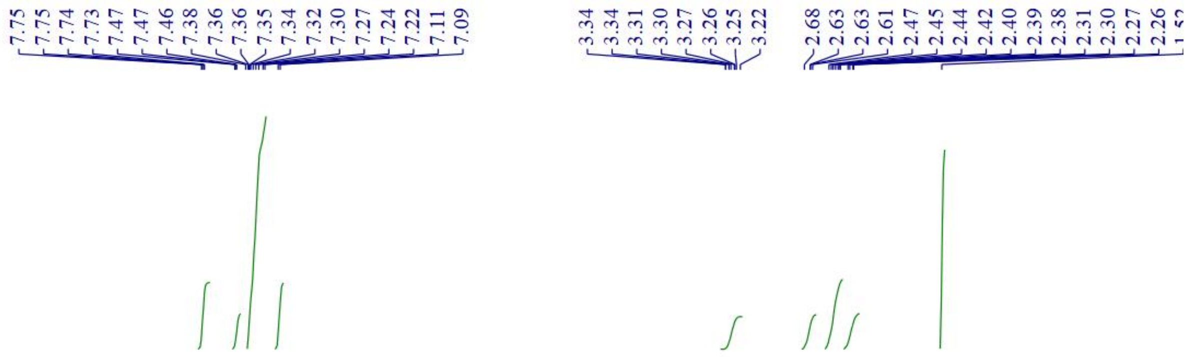

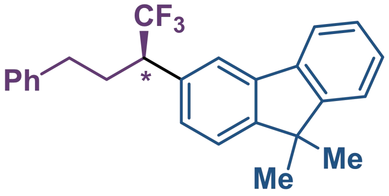

^{1}H NMR of **3p** (400 MHz, Chloroform-*d*, 298 K)

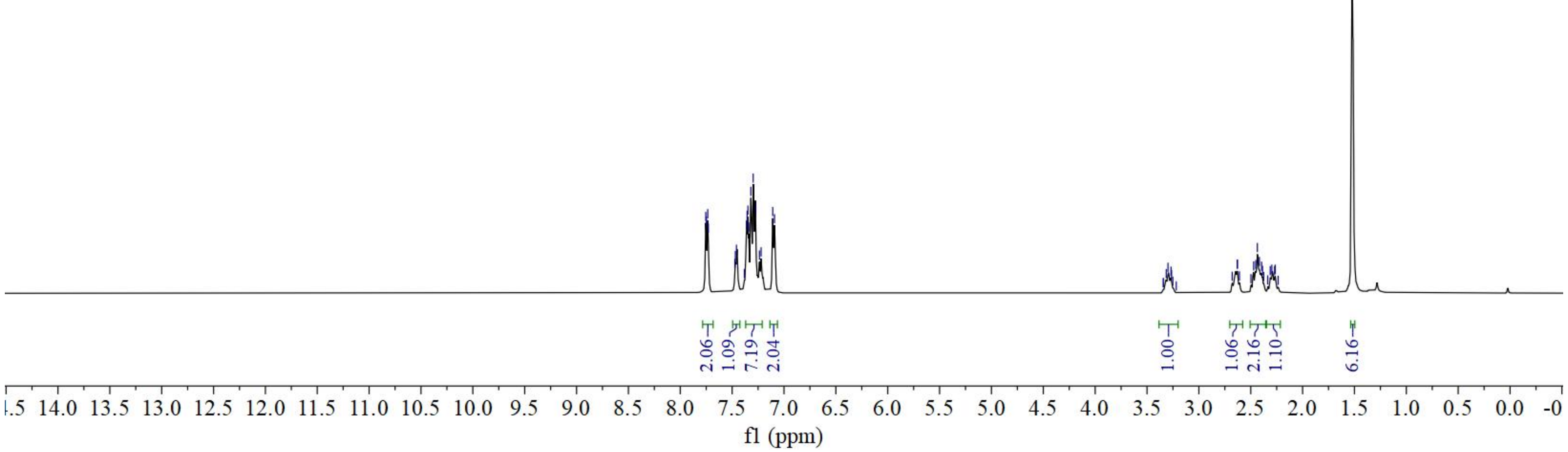

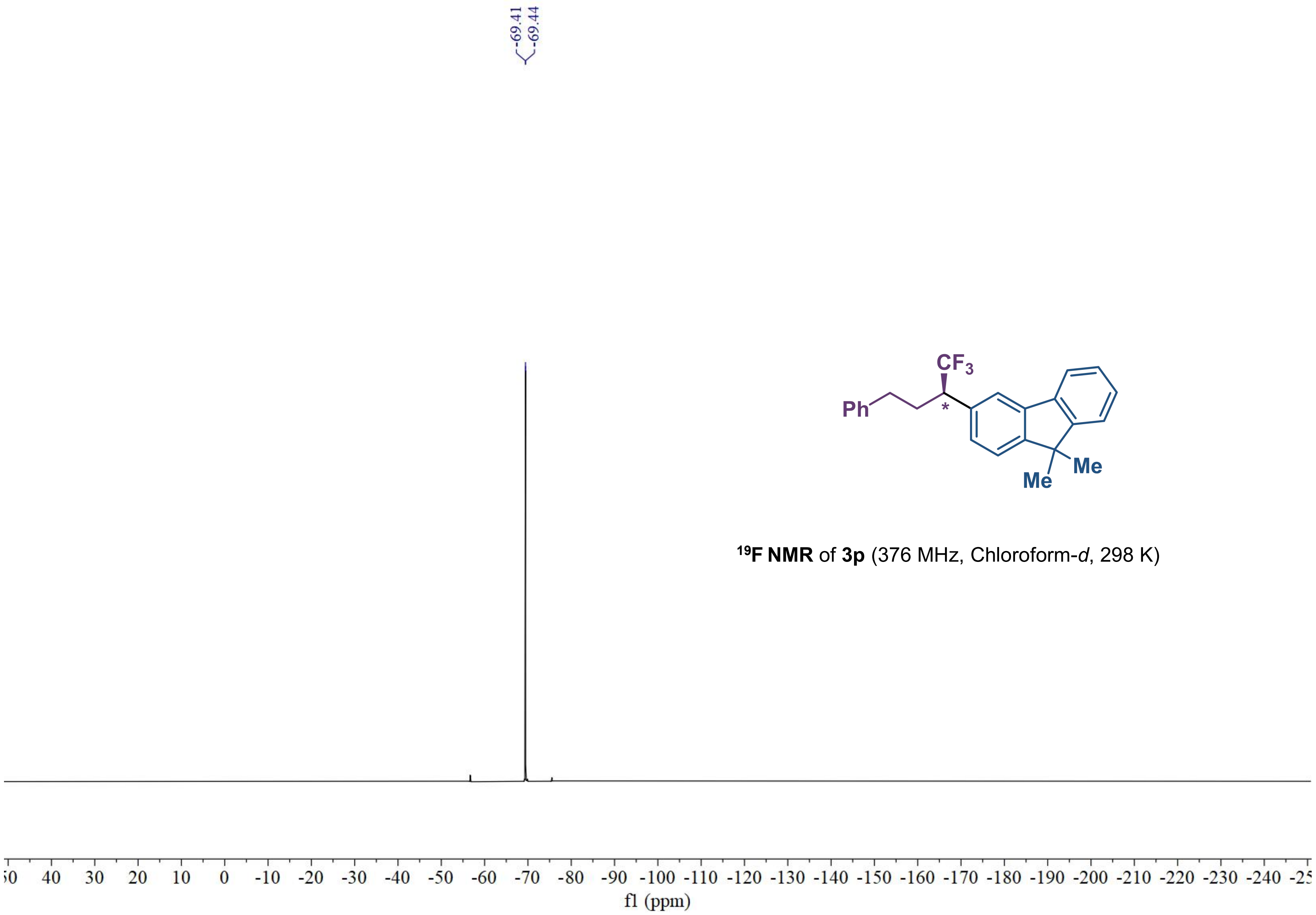

^{19}F NMR of **3p** (376 MHz, Chloroform-*d*, 298 K)

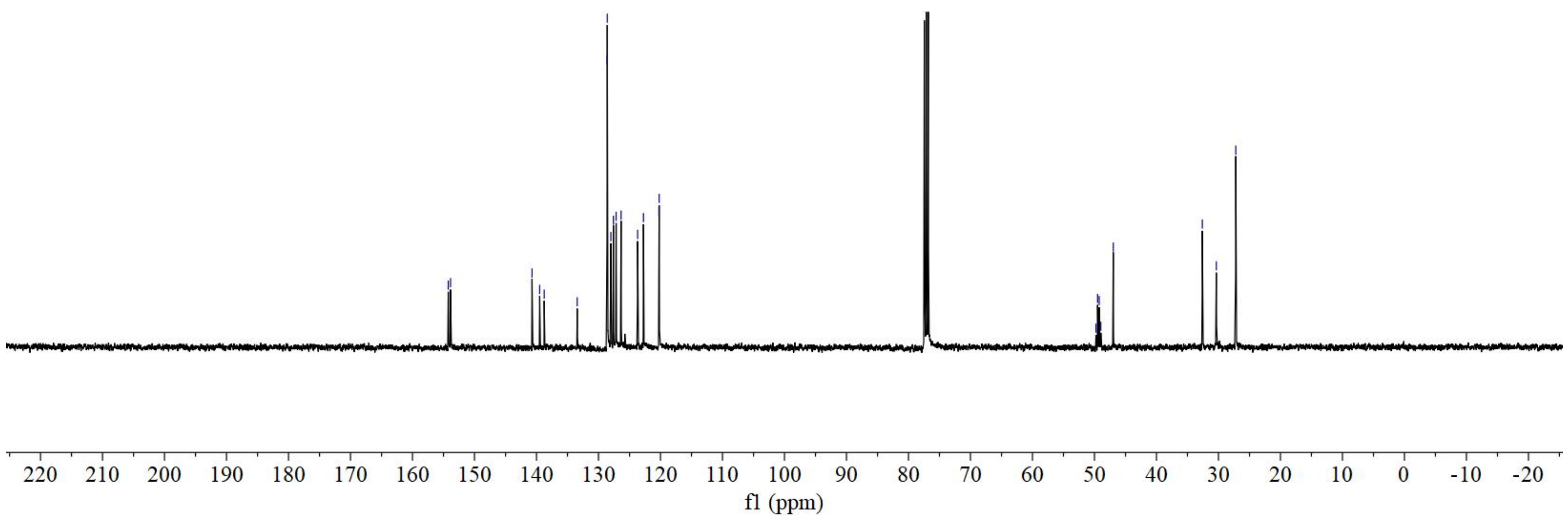

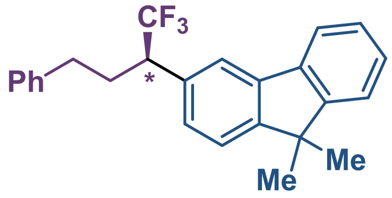

^{13}C NMR of **3p** (101 MHz, Chloroform-*d*, 298 K)

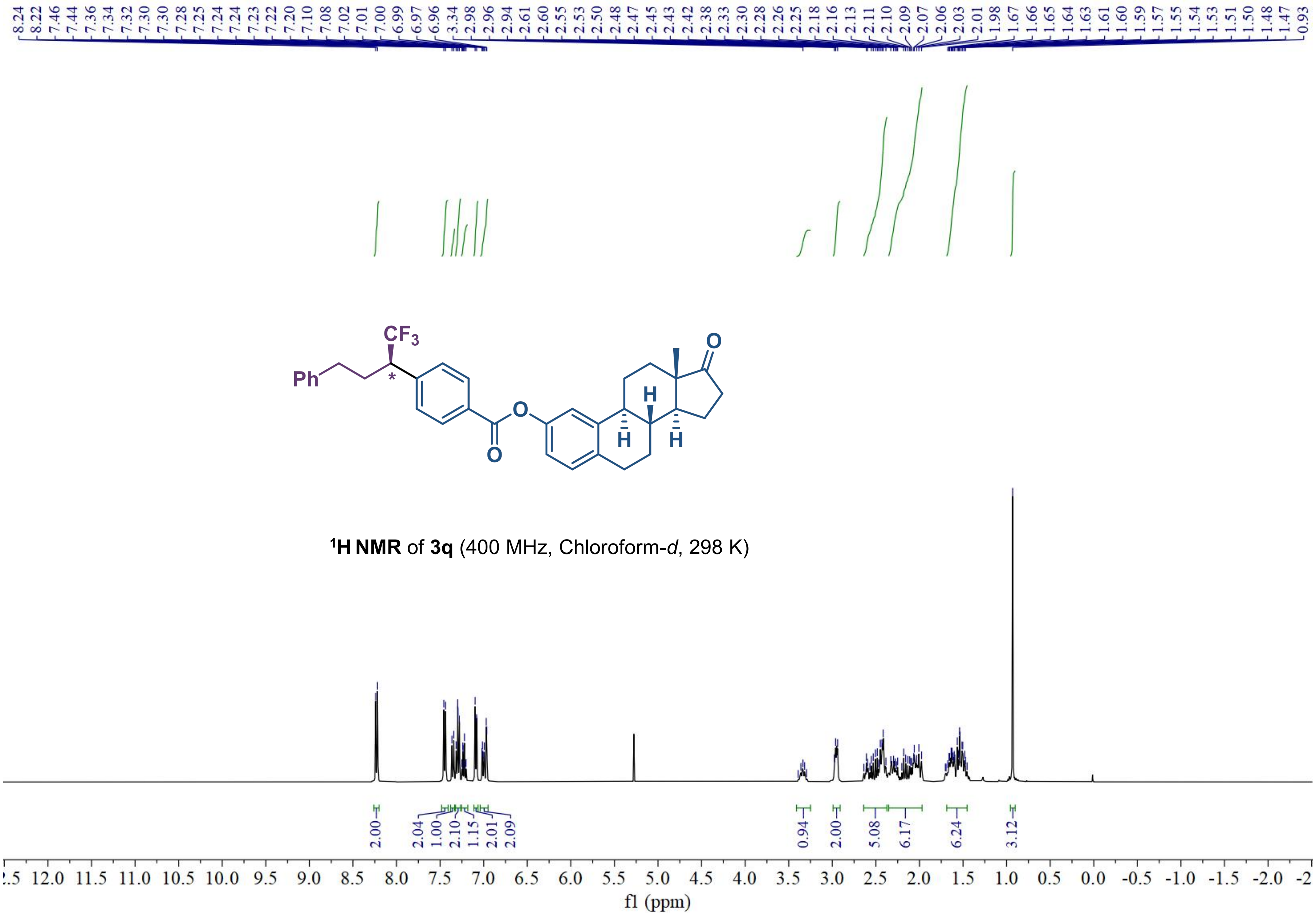

^{1}H NMR of **3q** (400 MHz, Chloroform-*d*, 298 K)

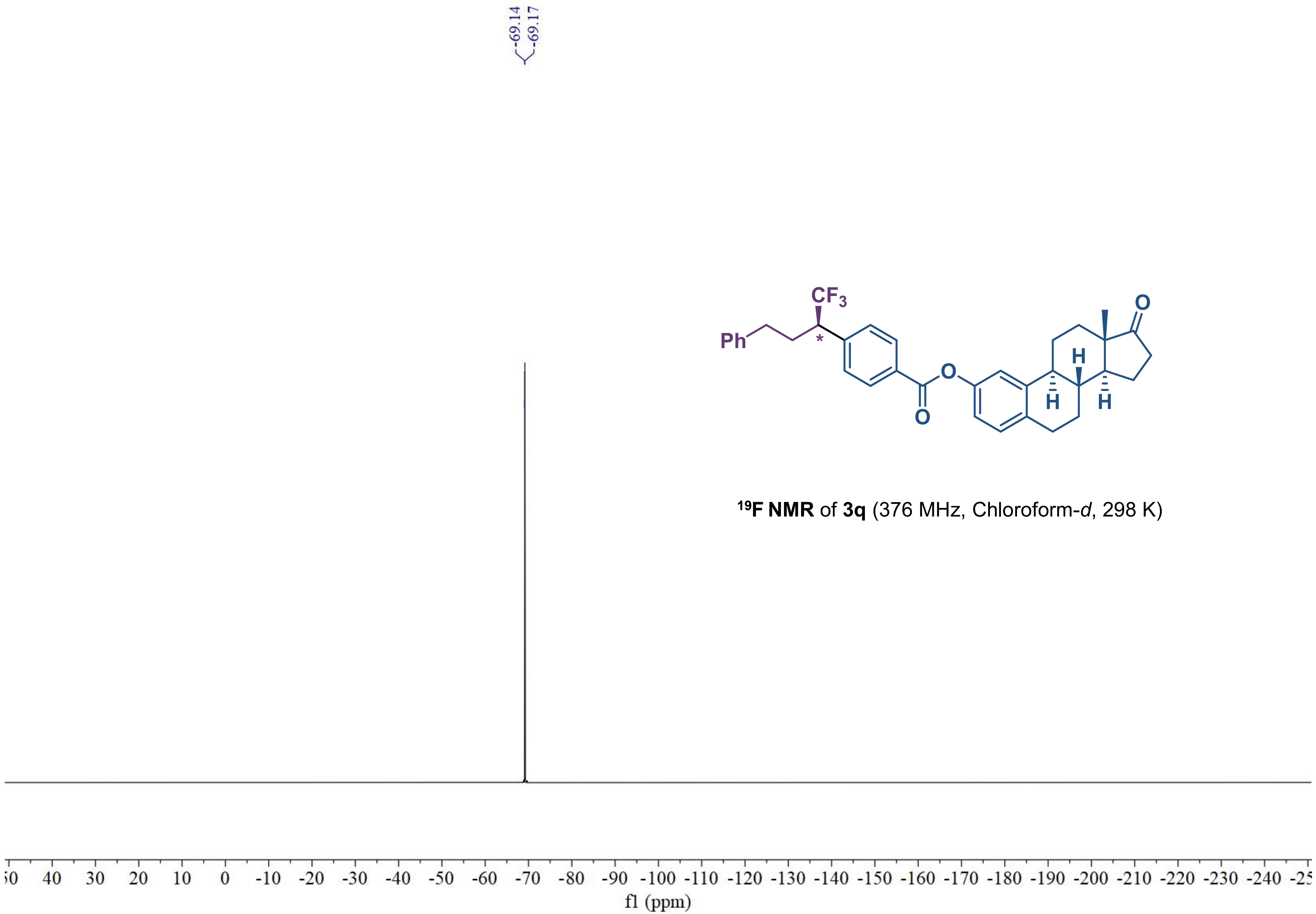

19F NMR of **3q** (376 MHz, Chloroform-*d*, 298 K)

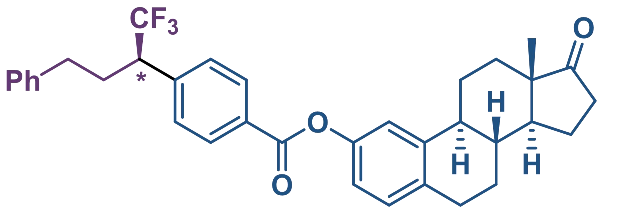

^{13}C NMR of **3q** (101 MHz, Chloroform-*d*, 298 K)

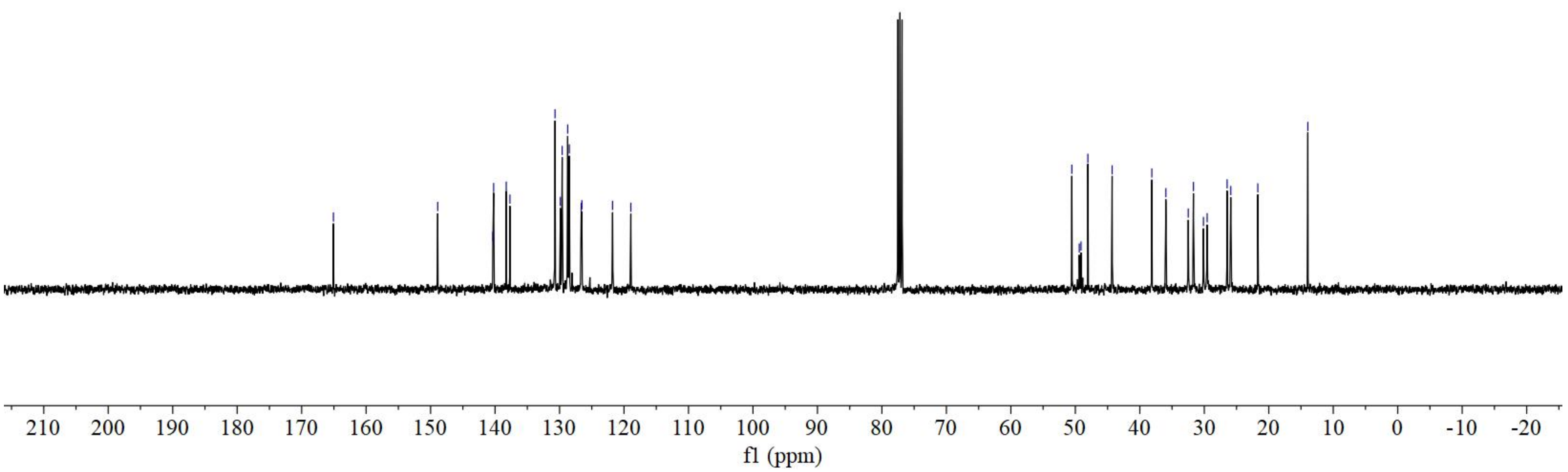

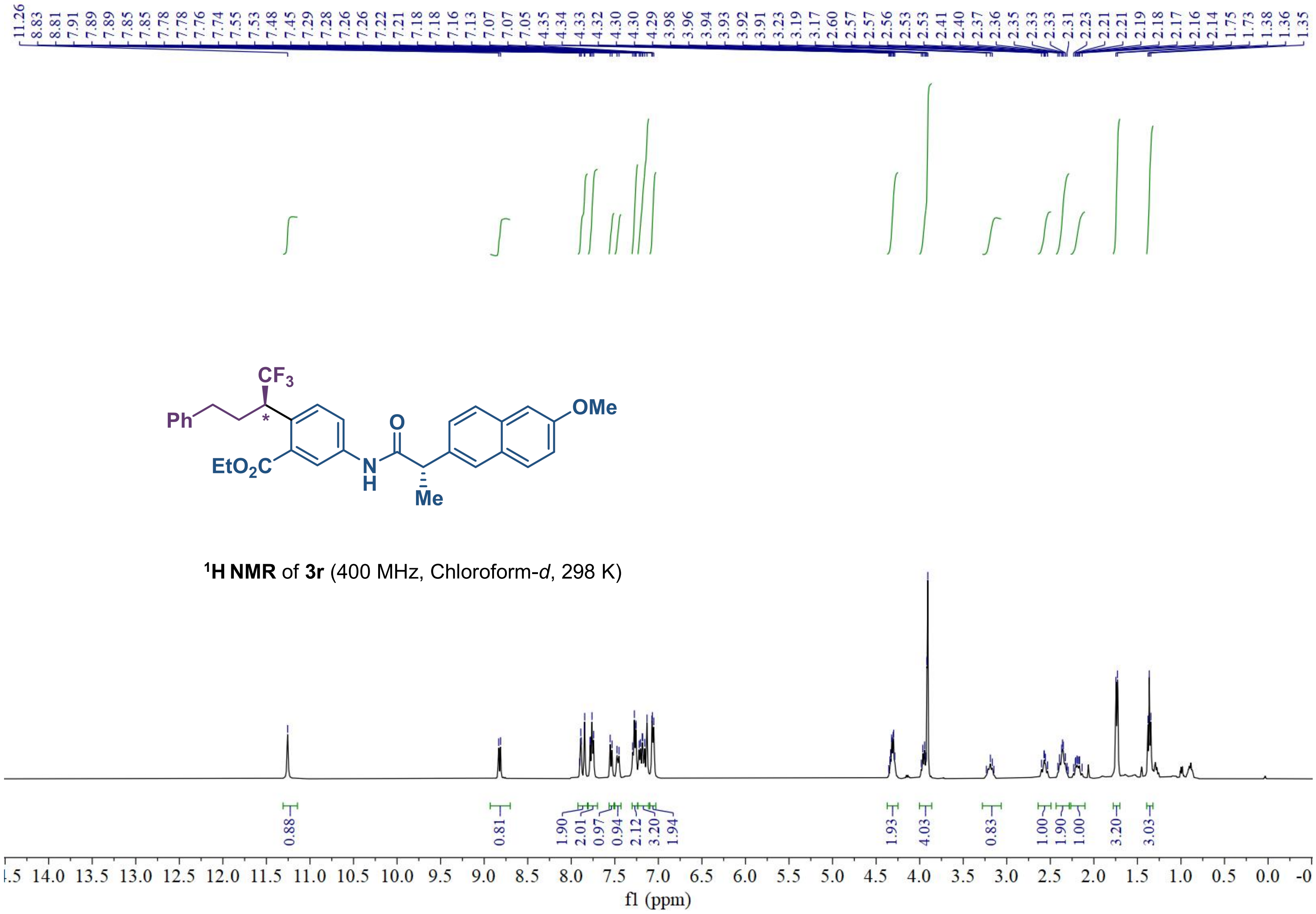

^{1}H NMR of **3r** (400 MHz, Chloroform-*d*, 298 K)

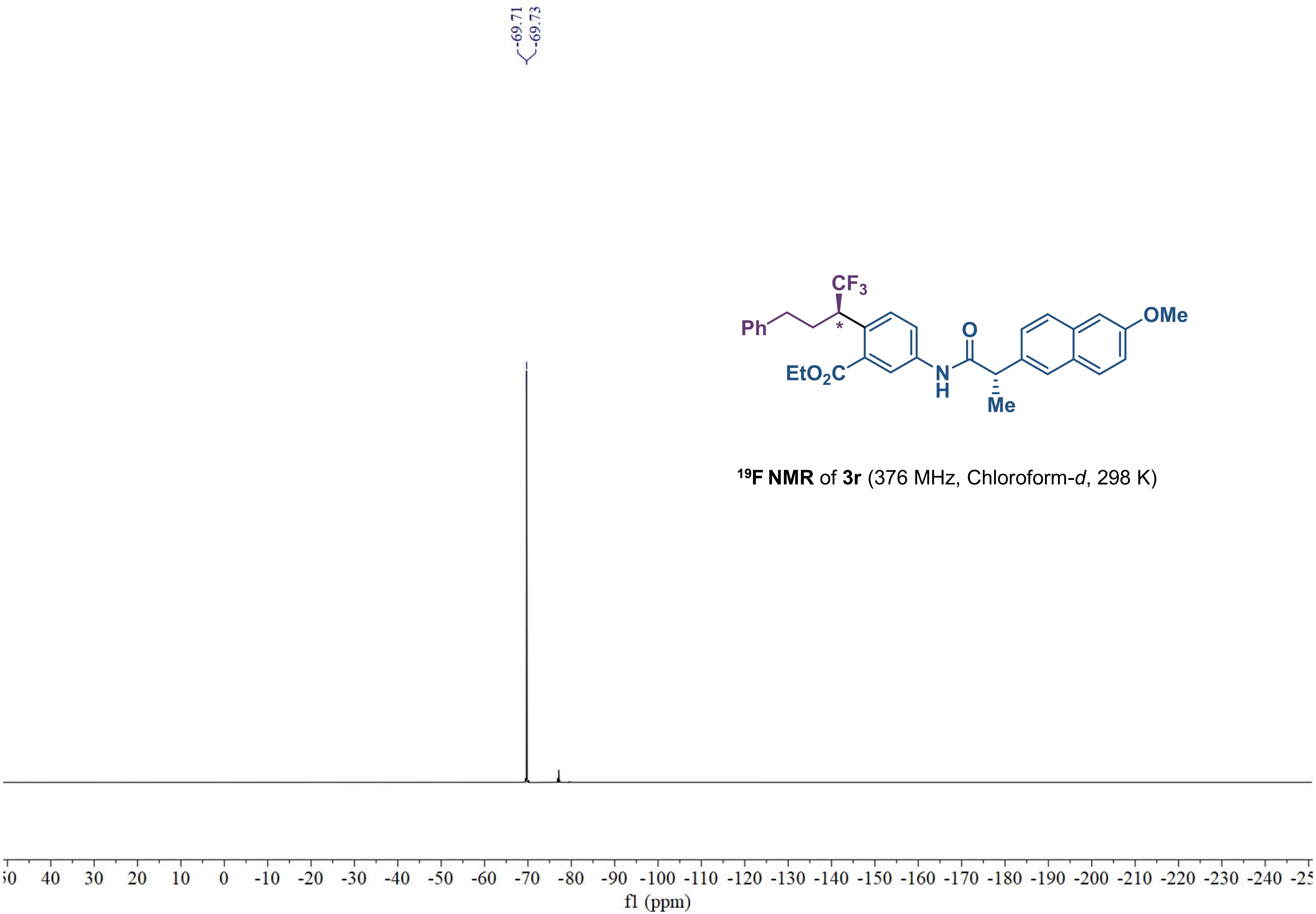

^{19}F NMR of **3r** (376 MHz, Chloroform-*d*, 298 K)

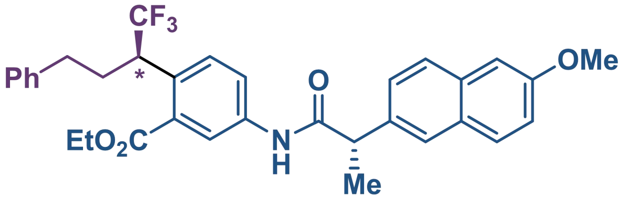

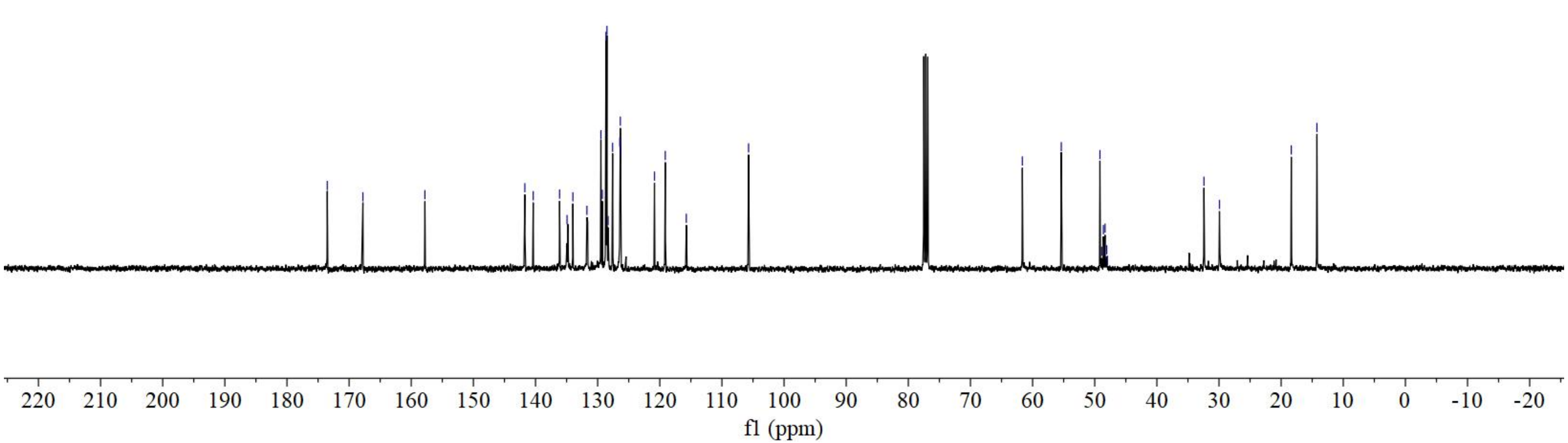

^{13}C NMR of **3r** (101 MHz, Chloroform-*d*, 298 K)

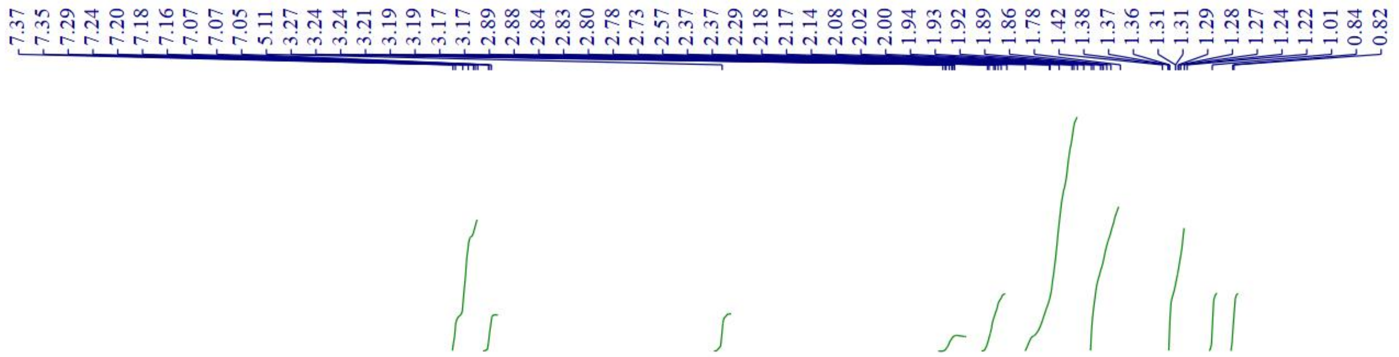

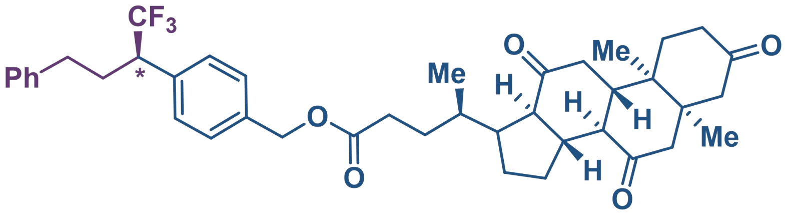

^{1}H NMR of **3s** (400 MHz, Chloroform-*d*, 298 K)

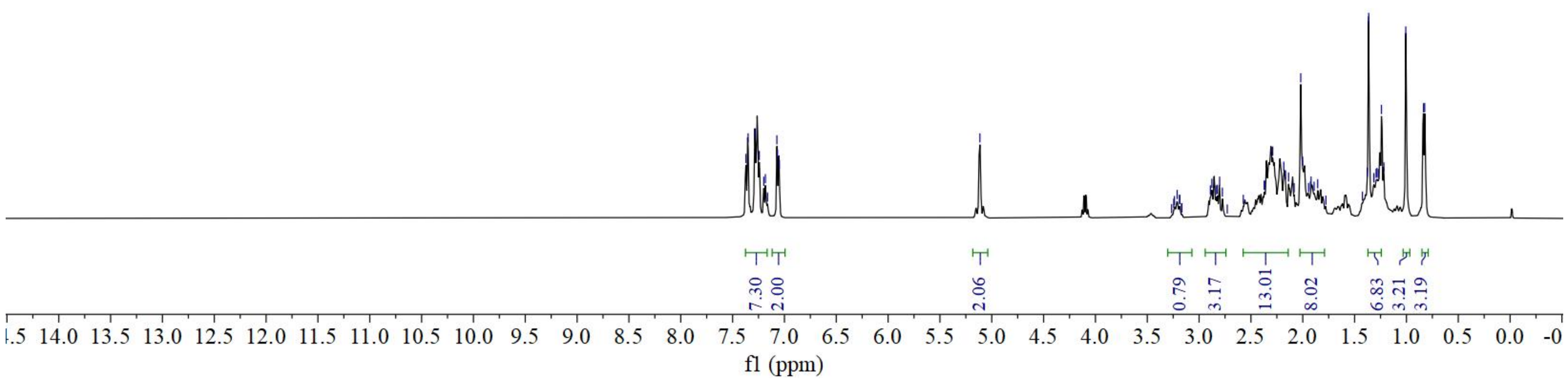

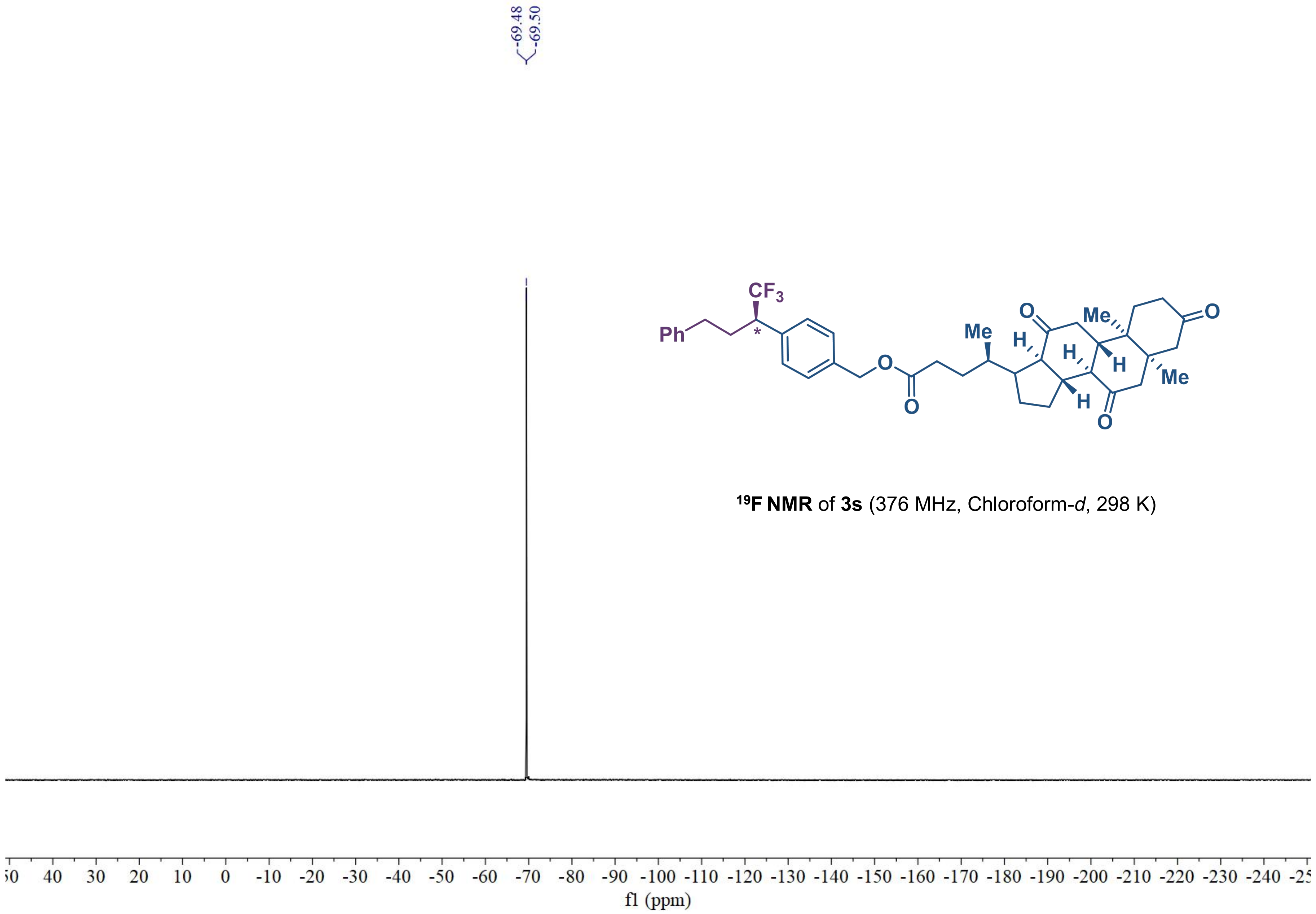

^{19}**F NMR** of **3s** (376 MHz, Chloroform-*d*, 298 K)

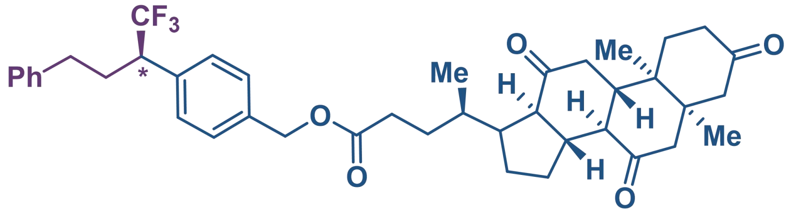

^{13}**C NMR** of **3s** (101 MHz, Chloroform-*d*, 298 K)

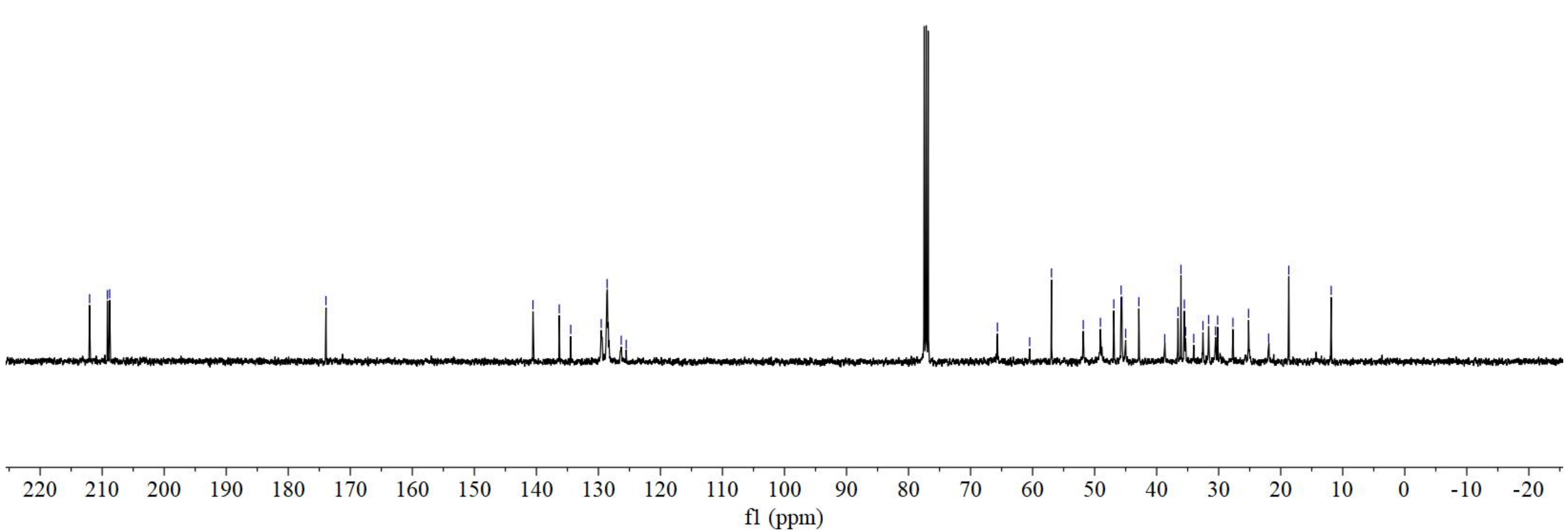

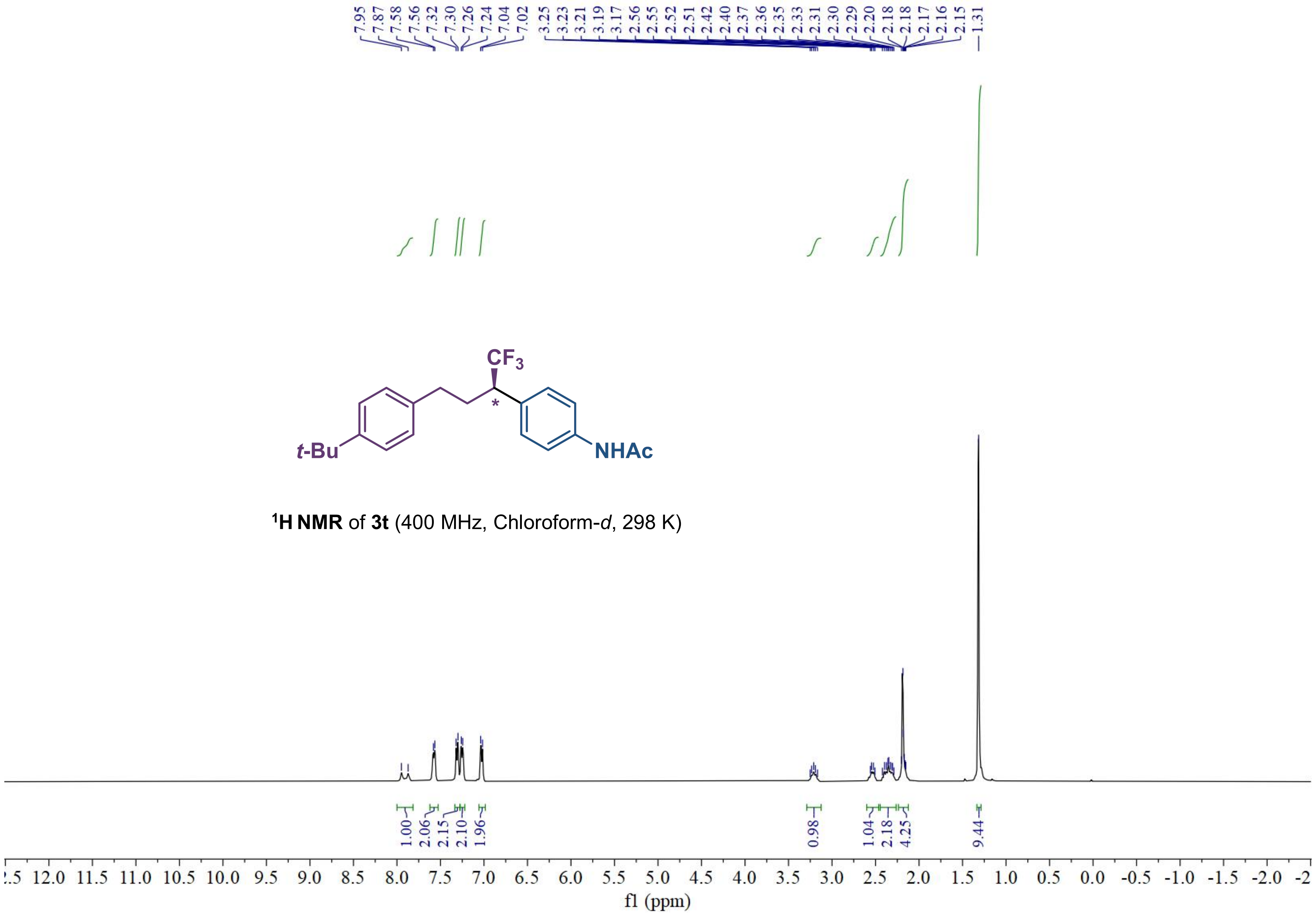

^{1}H NMR of 3t (400 MHz, Chloroform-d, 298 K)

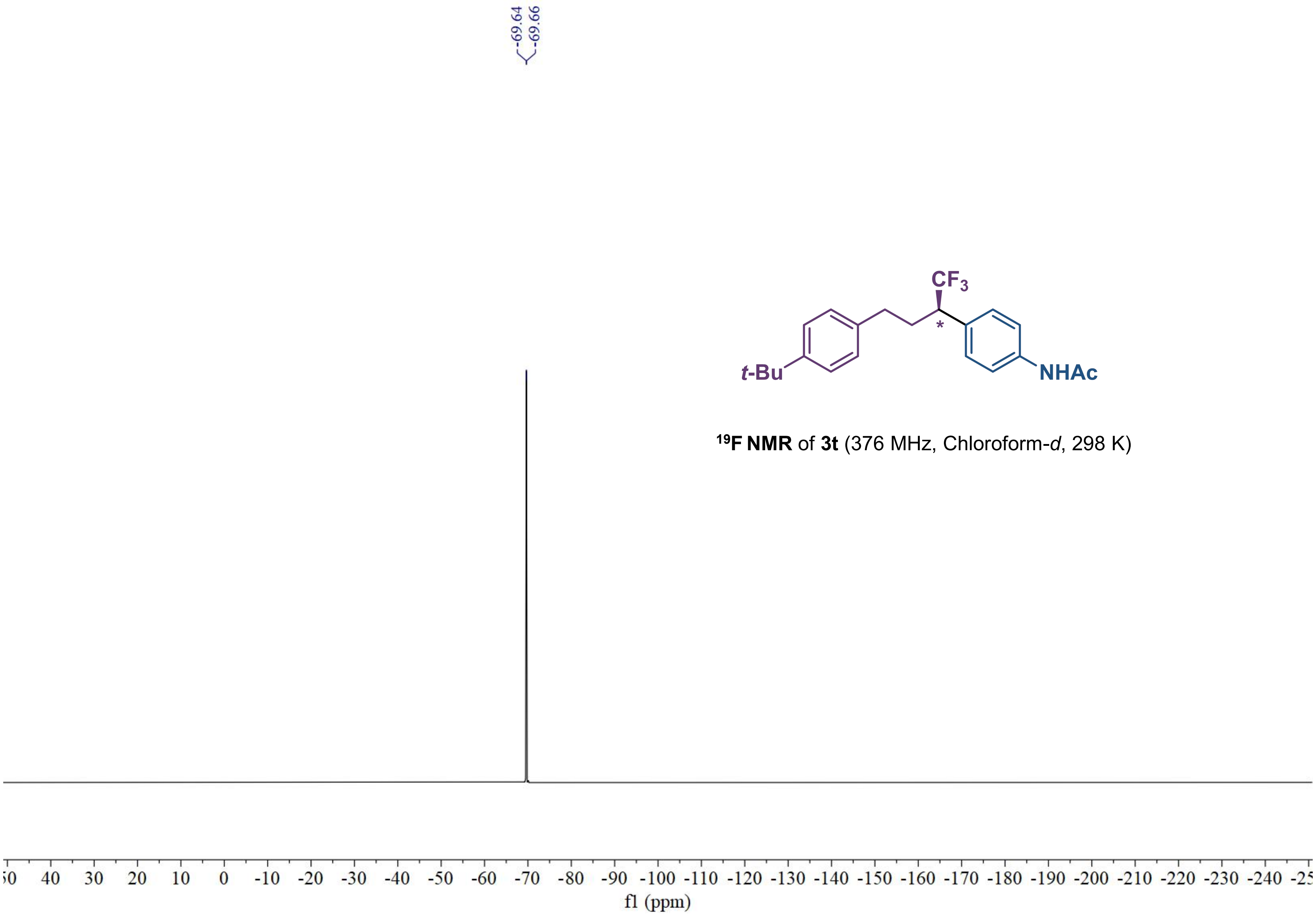

^{19}**F NMR** of **3t** (376 MHz, Chloroform-*d*, 298 K)

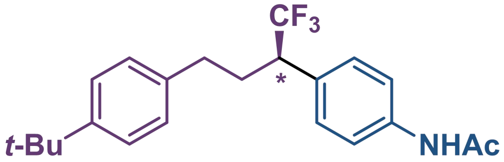

^{13}C NMR of **3t** (101 MHz, Chloroform-*d*, 298 K)

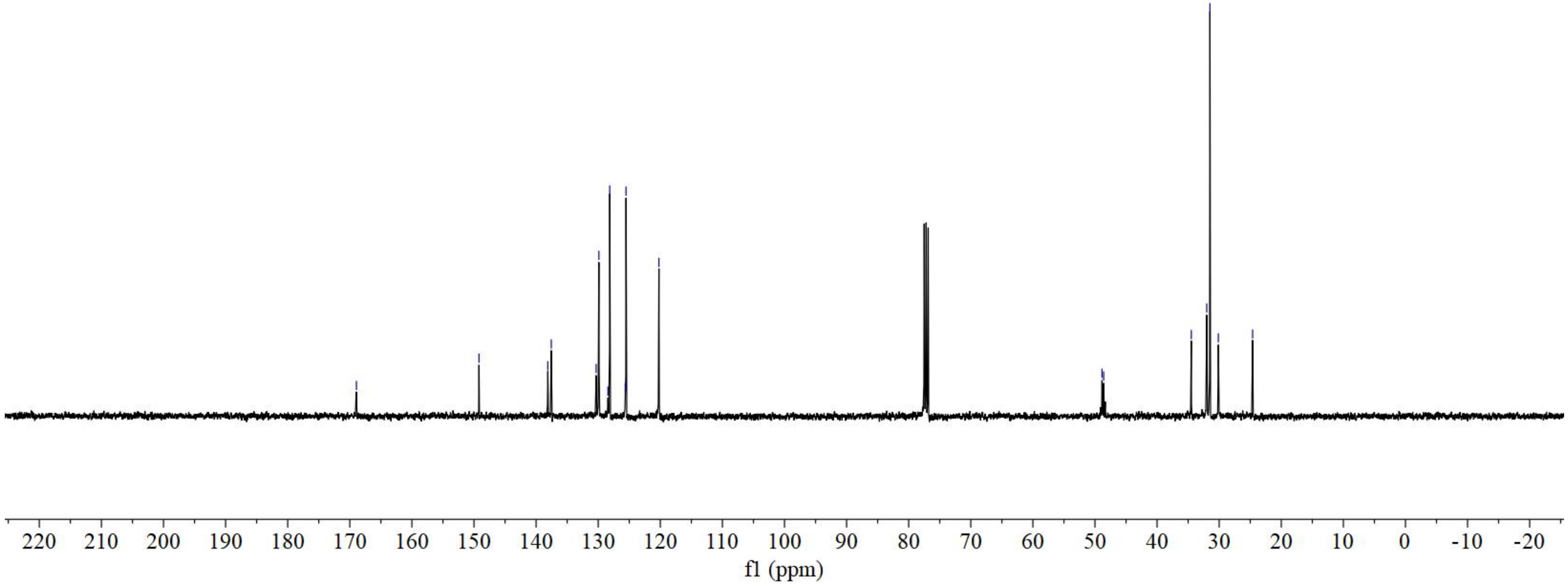

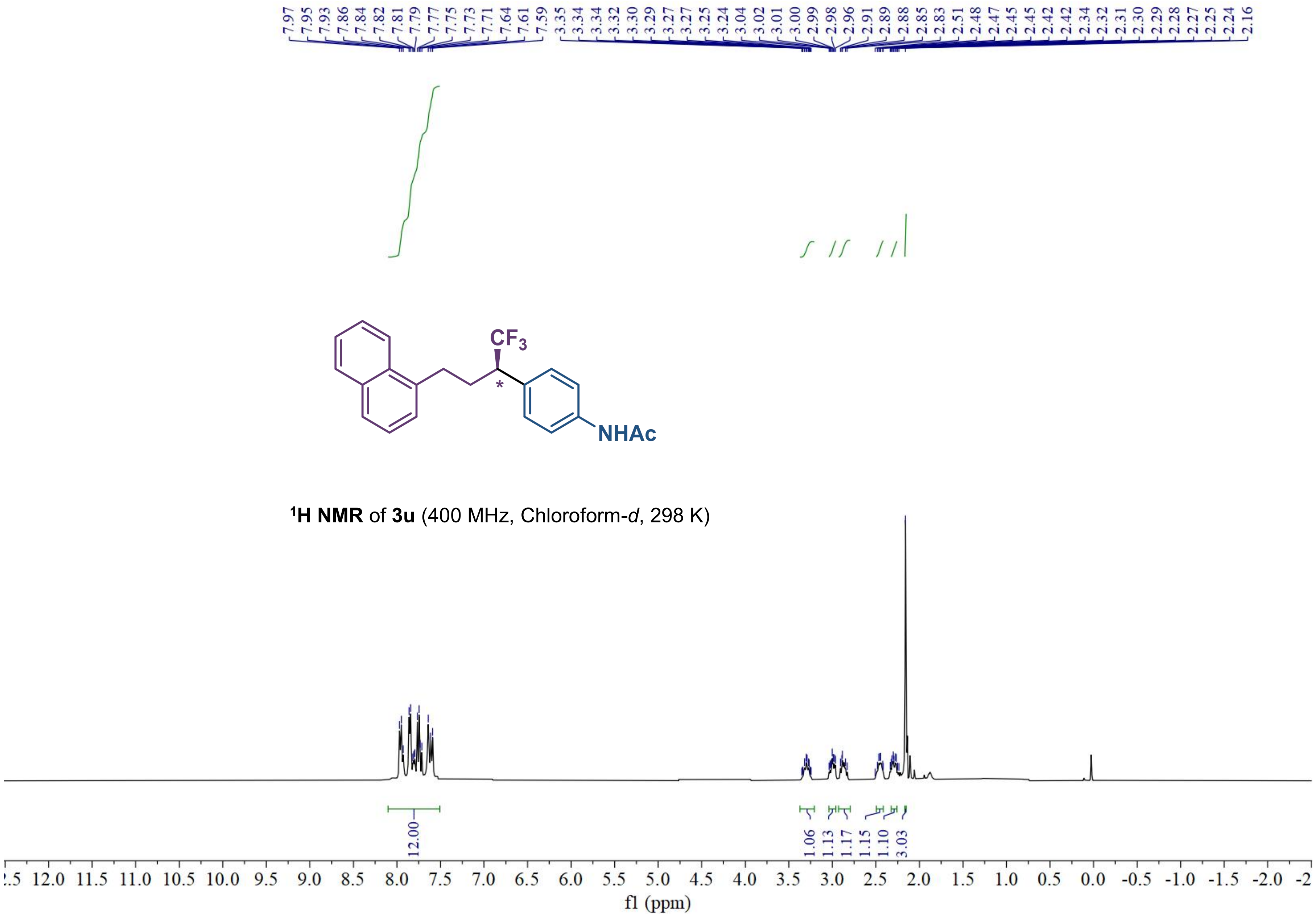

^{1}H NMR of **3u** (400 MHz, Chloroform-*d*, 298 K)

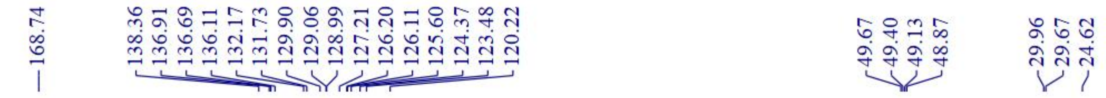

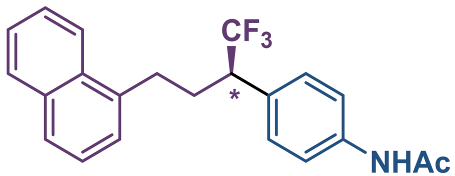

^{13}C NMR of **3u** (101 MHz, Chloroform-*d*, 298 K)

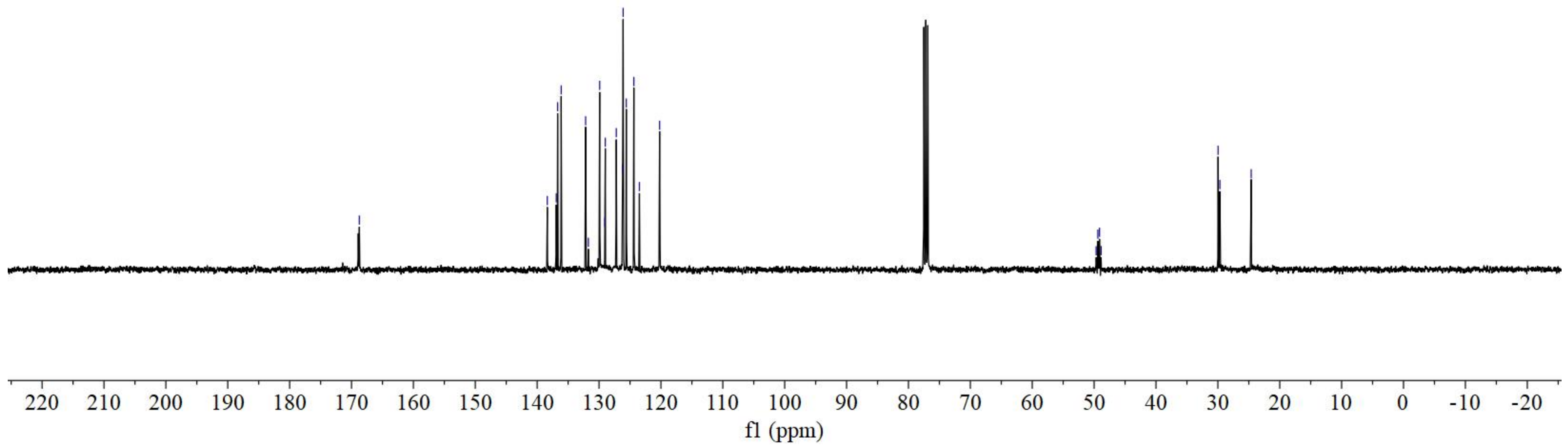

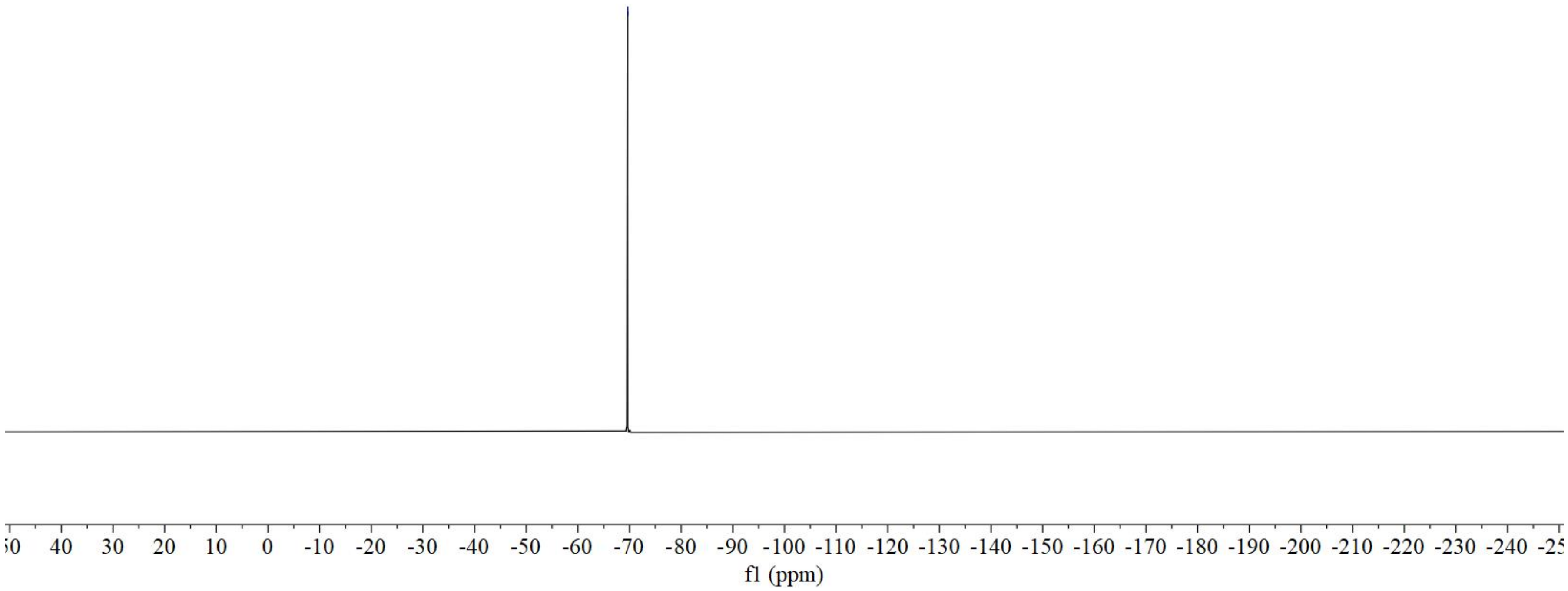

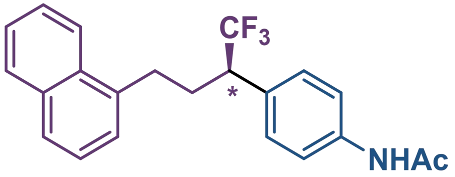

^{19}F NMR of **3u** (376 MHz, Chloroform-*d*, 298 K)

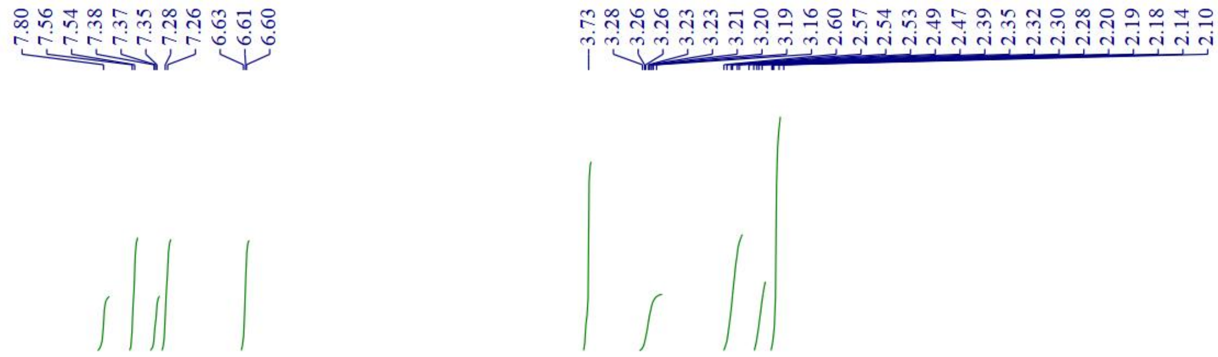

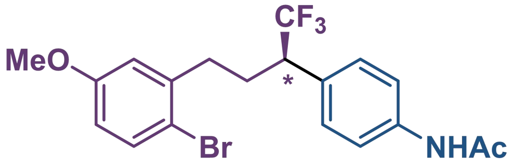

^{1}**H NMR** of **3v** (400 MHz, Chloroform-*d*, 298 K)

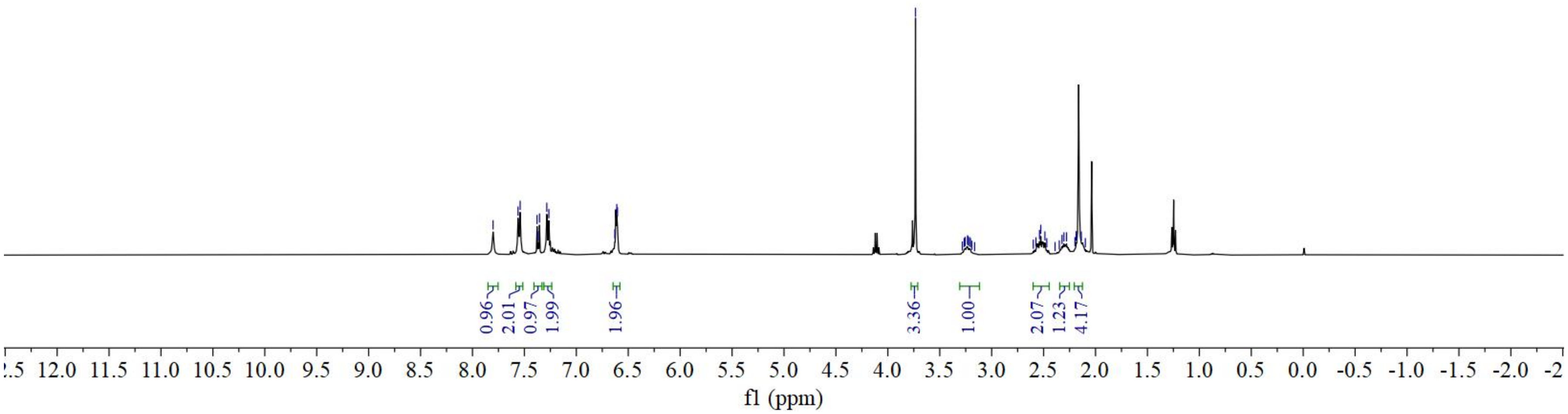

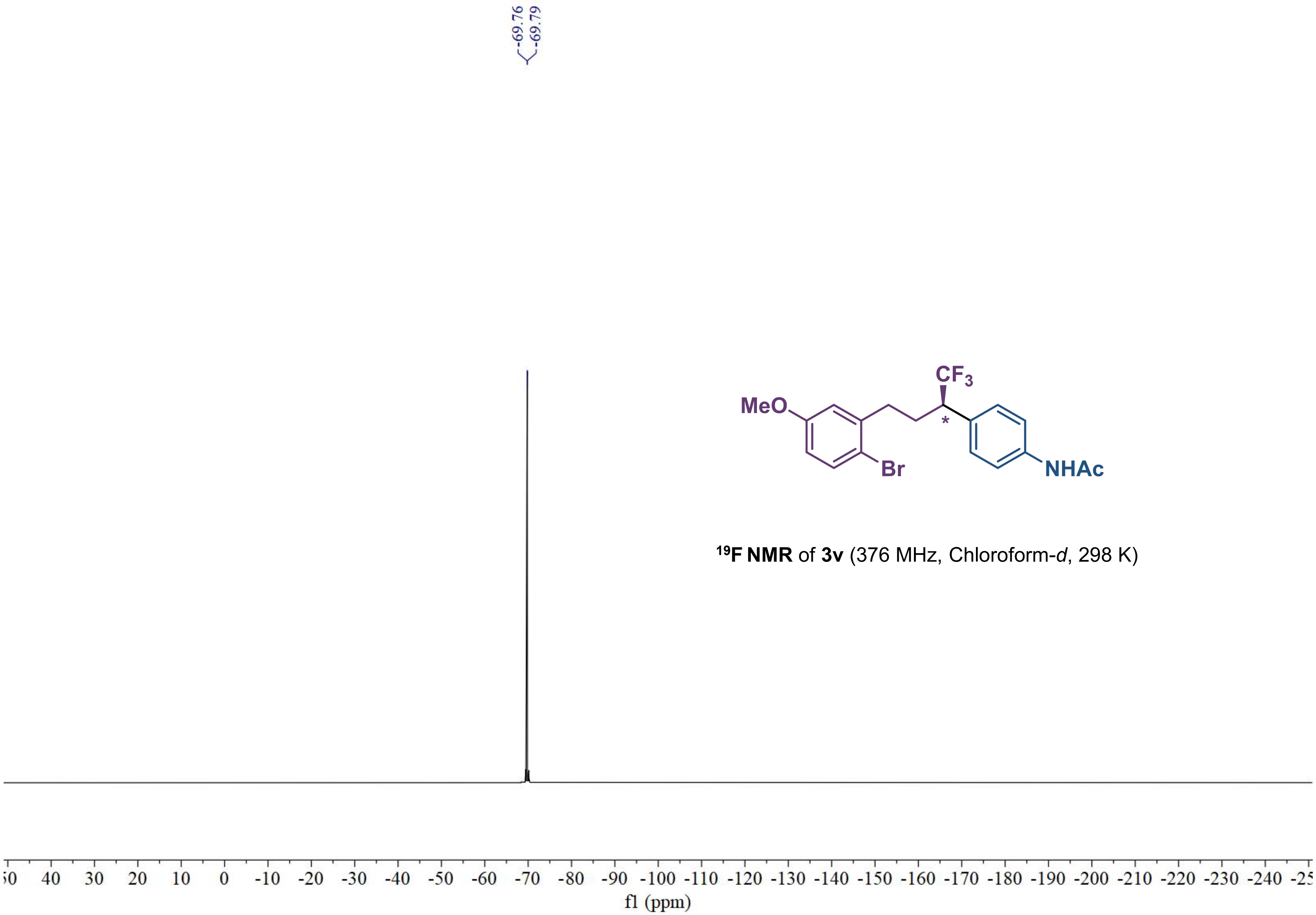

^{19}F NMR of **3v** (376 MHz, Chloroform-*d*, 298 K)

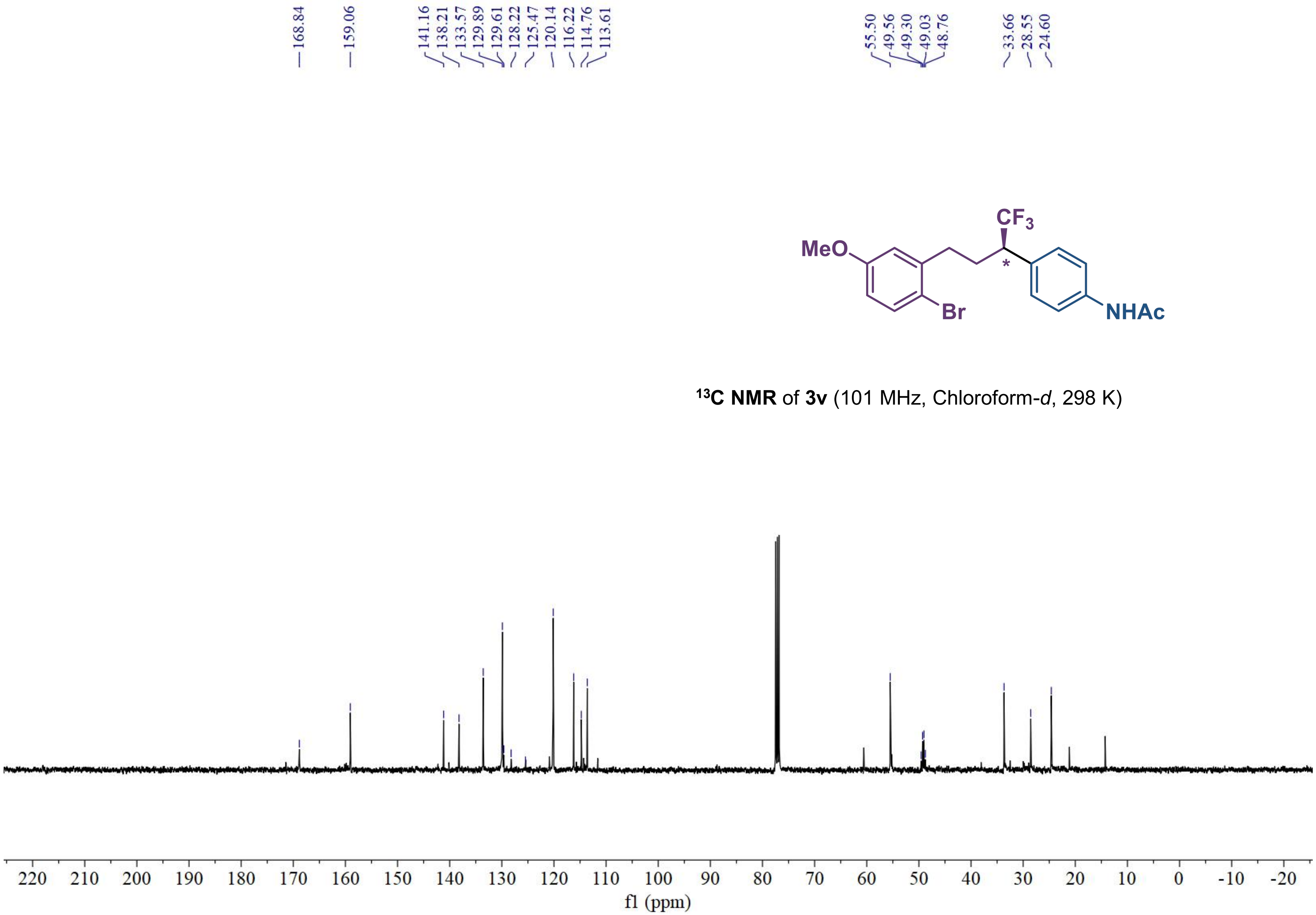

^{13}C NMR of **3v** (101 MHz, Chloroform-*d*, 298 K)

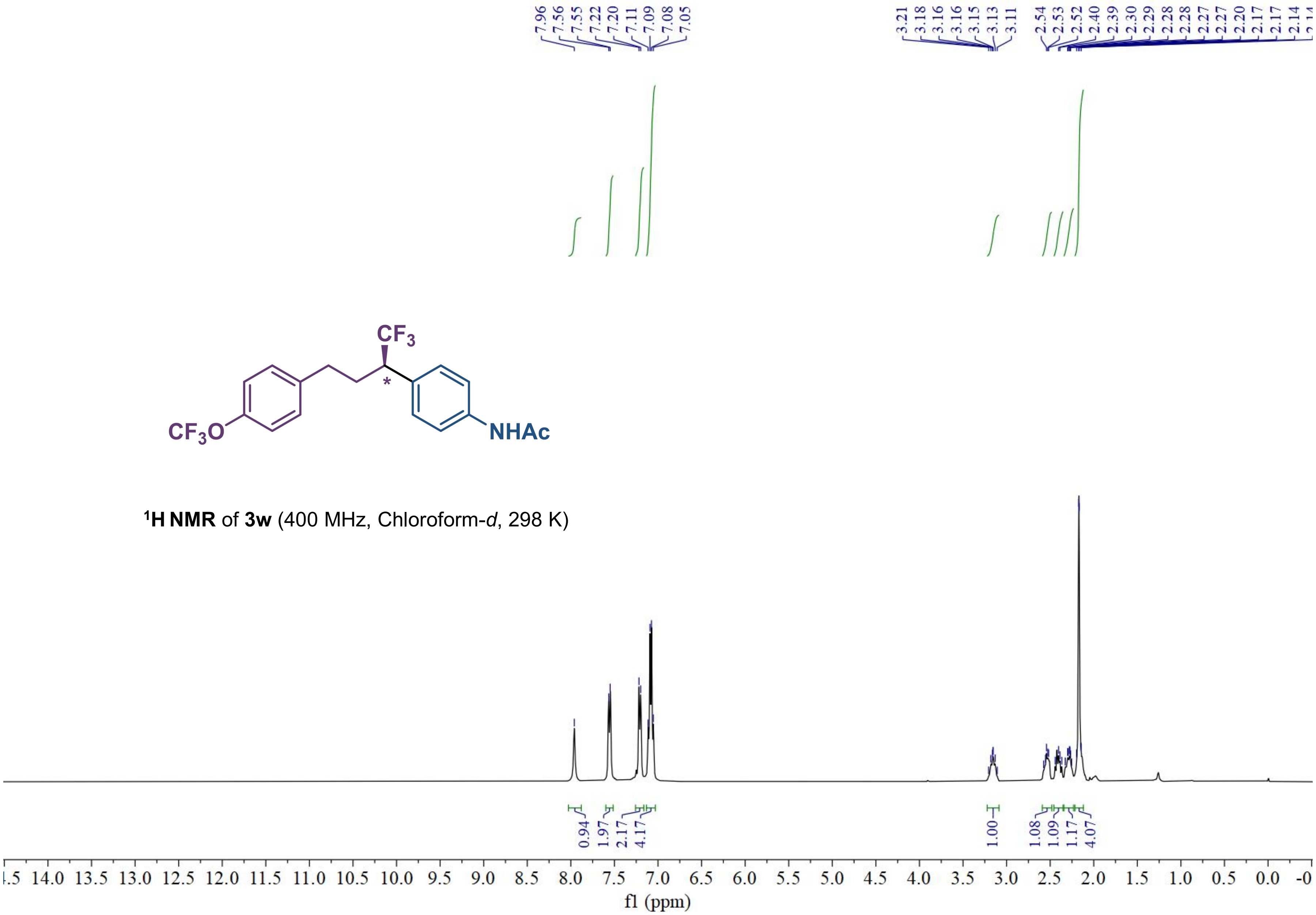

^{1}**H NMR** of **3w** (400 MHz, Chloroform-*d*, 298 K)

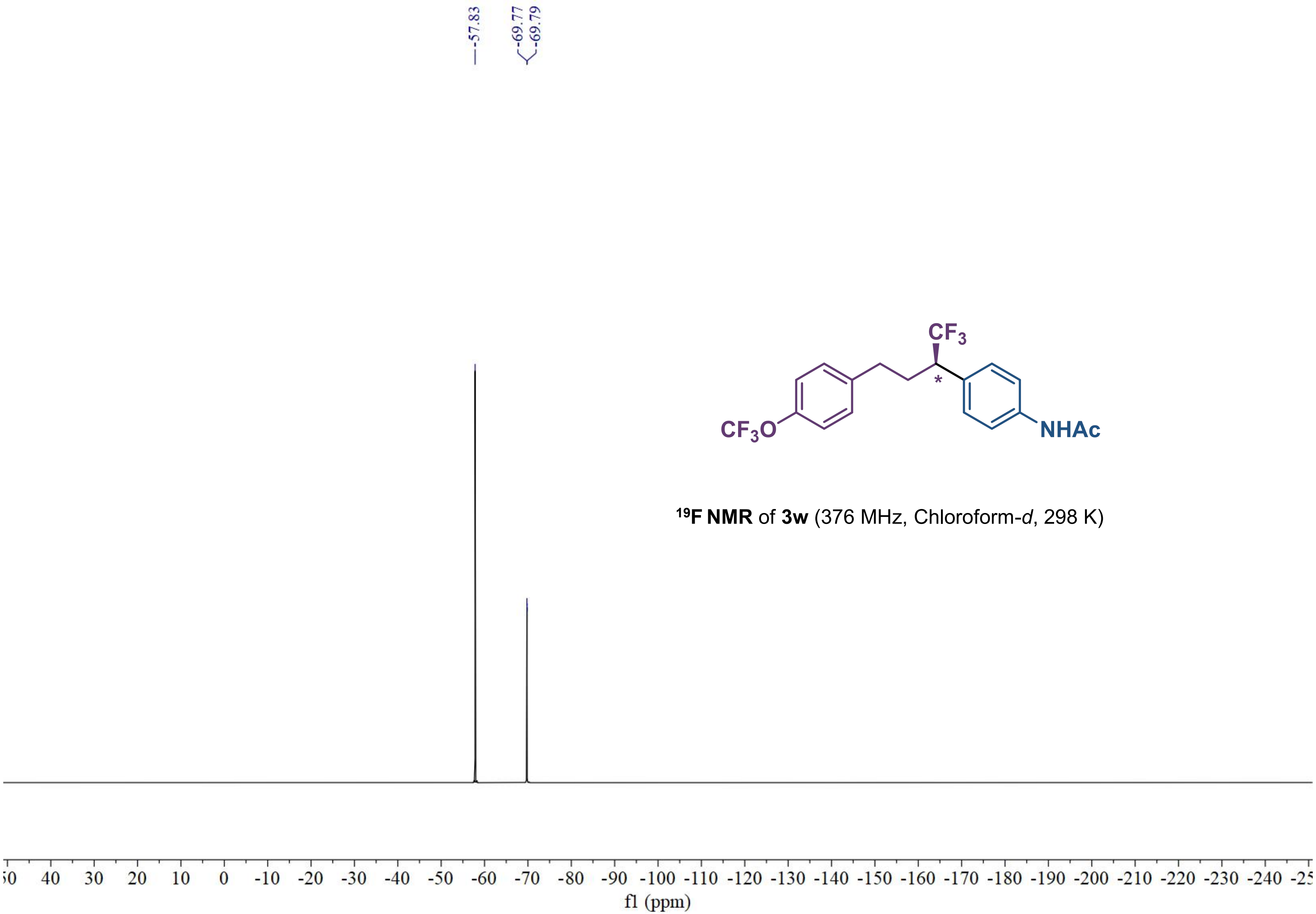

^{19}F NMR of **3w** (376 MHz, Chloroform-*d*, 298 K)

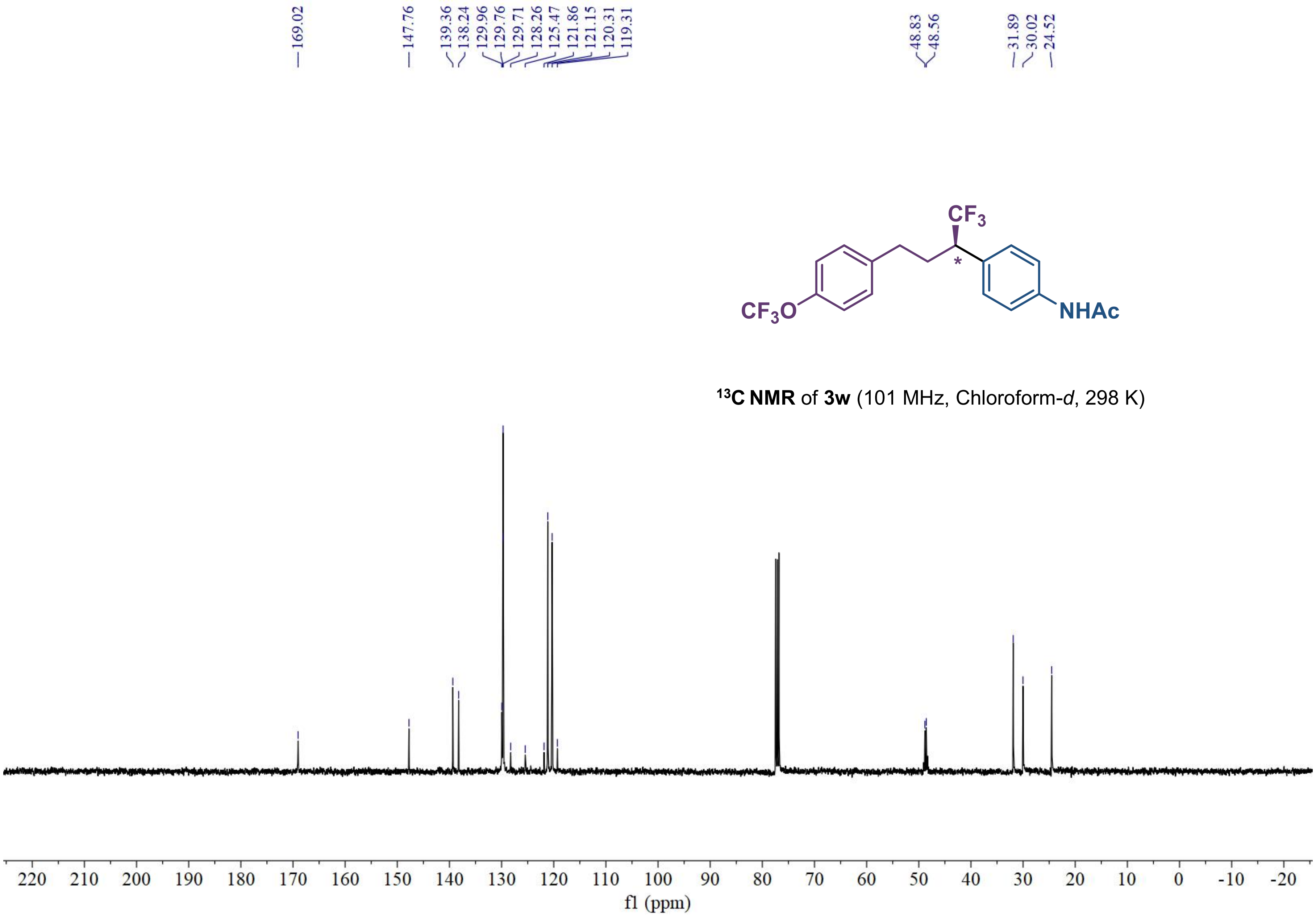

^{13}**C NMR** of **3w** (101 MHz, Chloroform-*d*, 298 K)

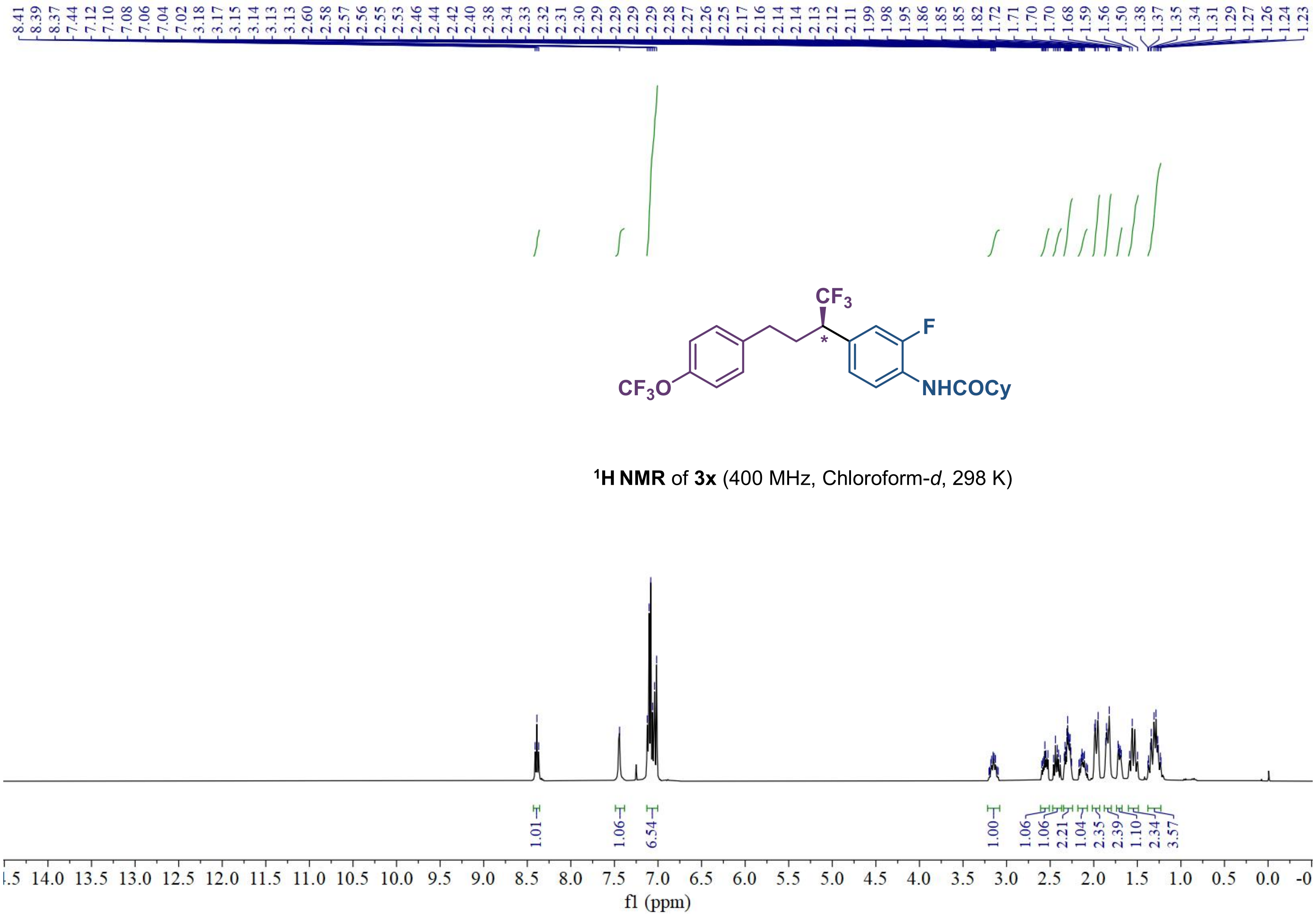

1H NMR of **3x** (400 MHz, Chloroform-*d*, 298 K)

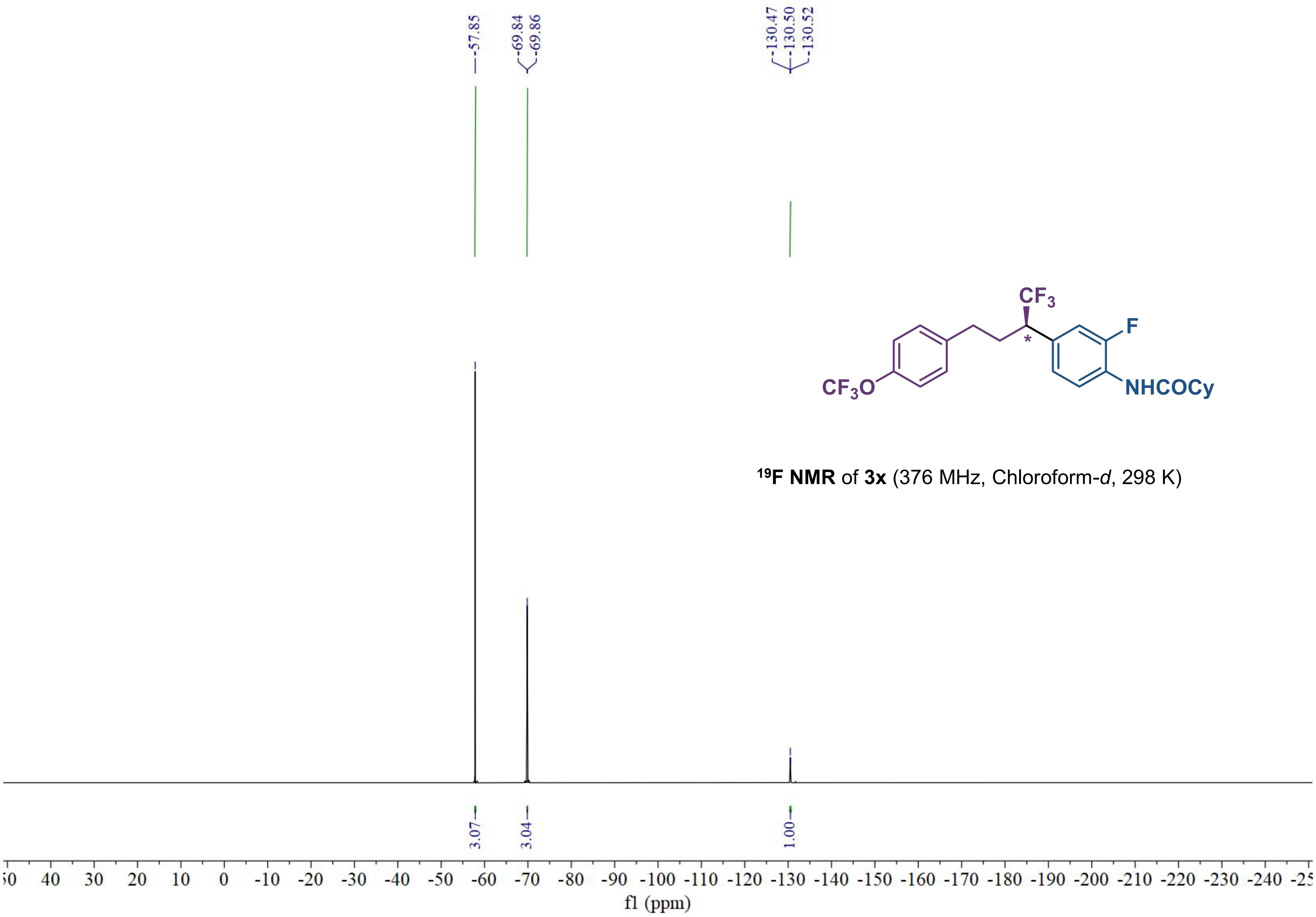

19F NMR of **3x** (376 MHz, Chloroform-*d*, 298 K)

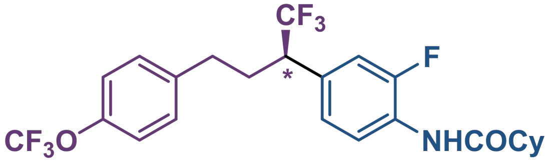

^{13}C NMR of 3x (101 MHz, Chloroform-*d*, 298 K)

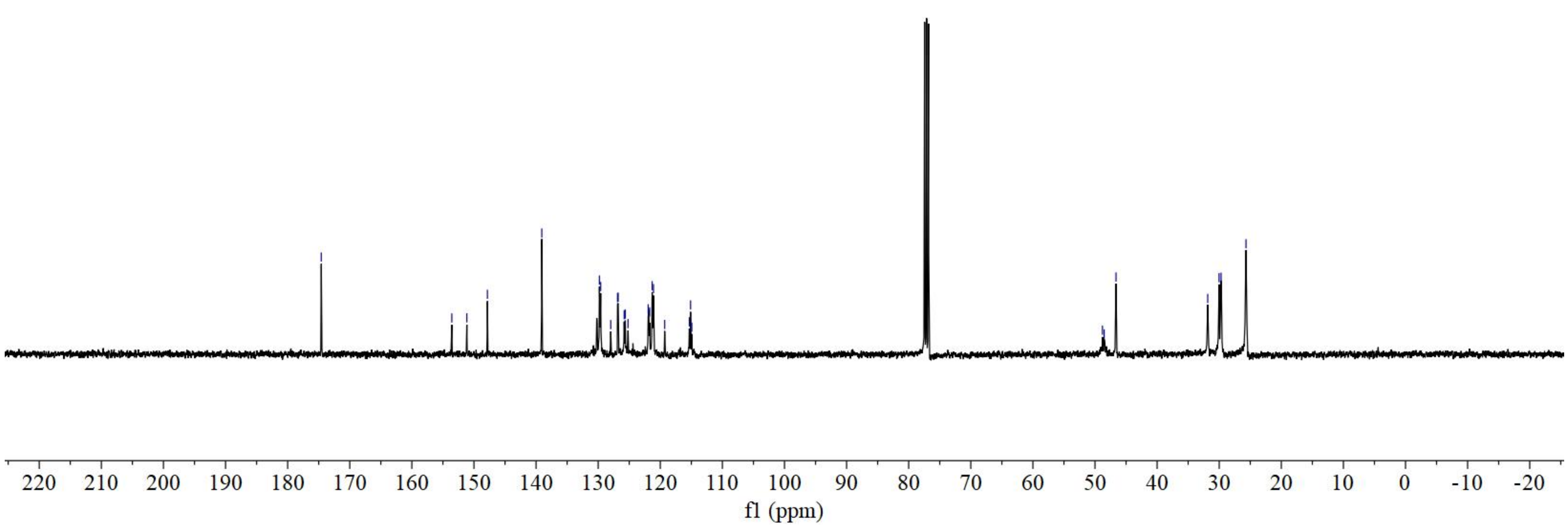

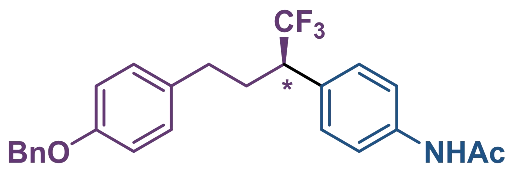

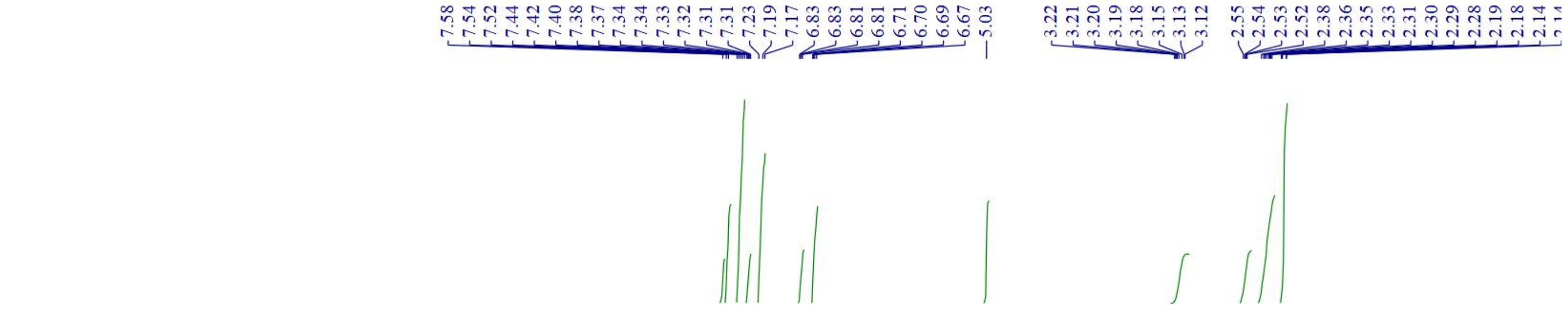

^{1}H NMR of **3y** (400 MHz, Chloroform-*d*, 298 K)

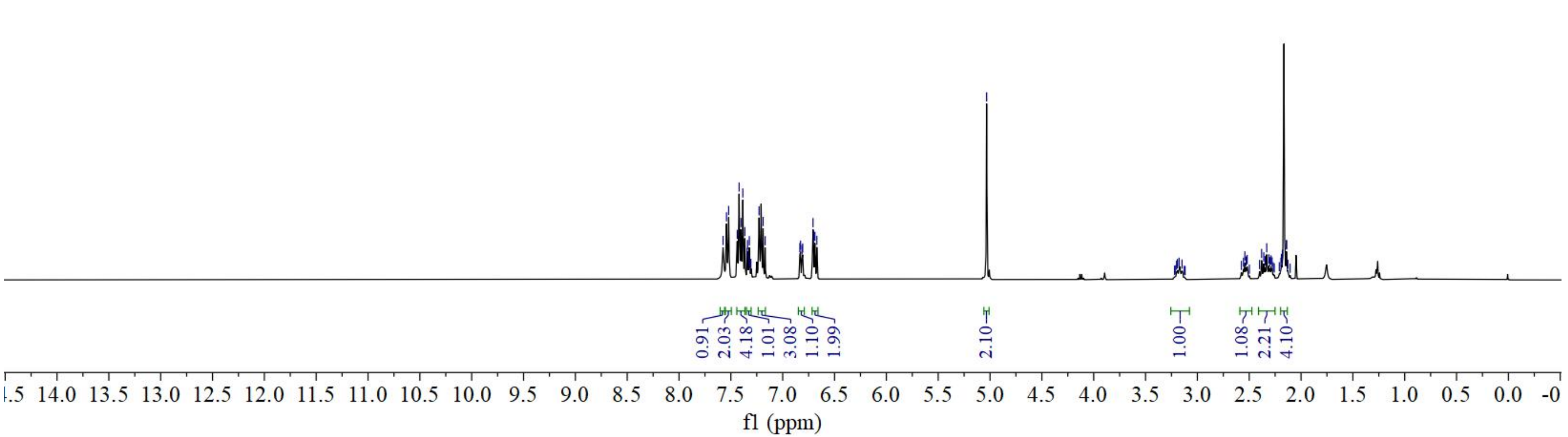

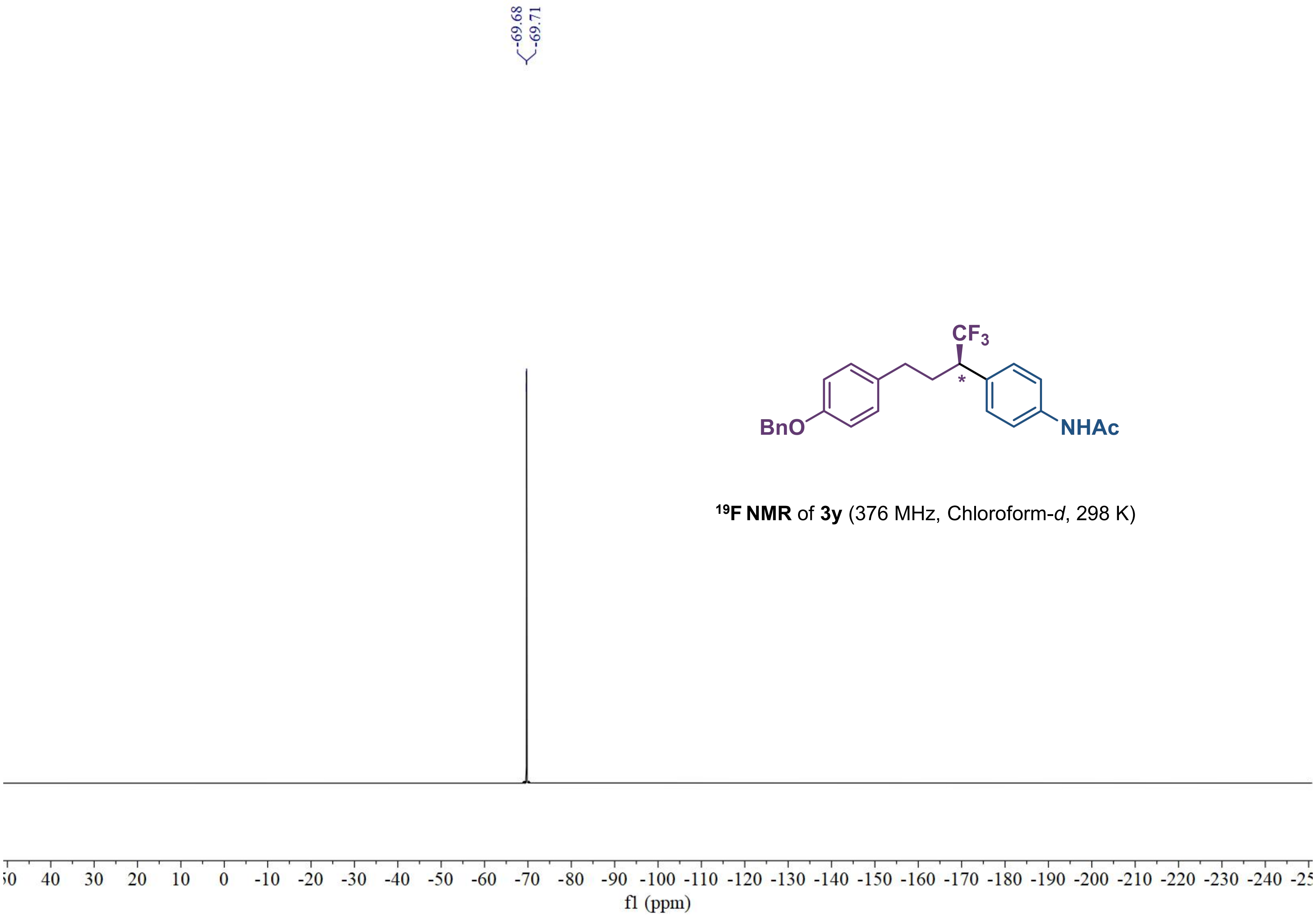

^{19}F NMR of **3y** (376 MHz, Chloroform-*d*, 298 K)

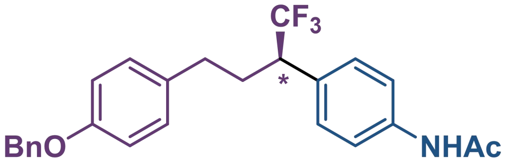

13C NMR of **3y** (101 MHz, Chloroform-*d*, 298 K)

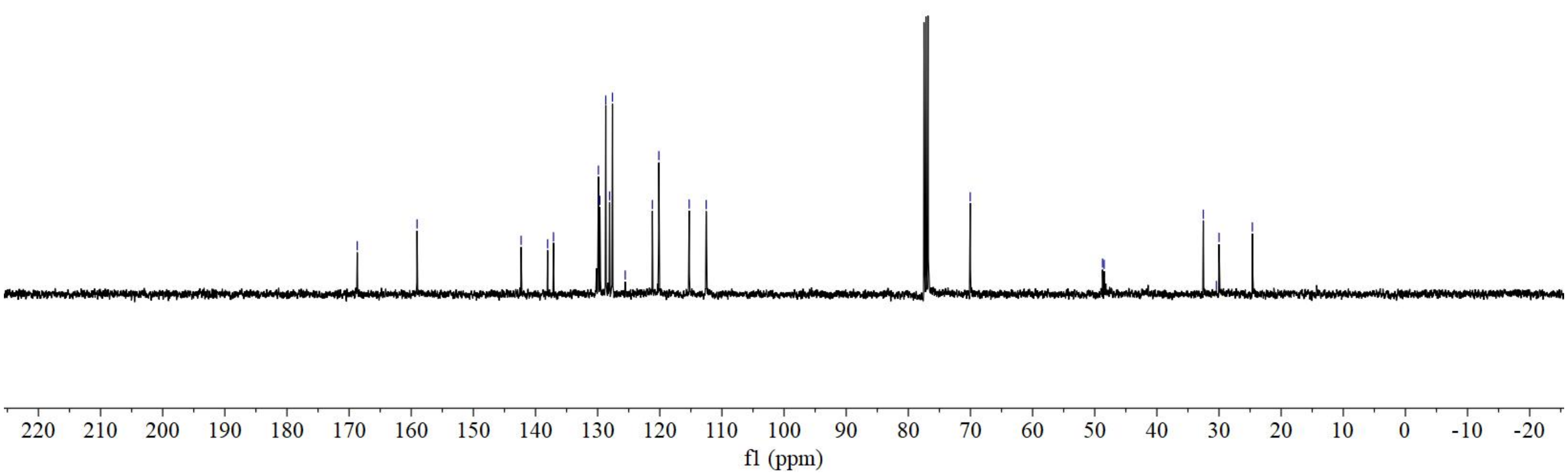

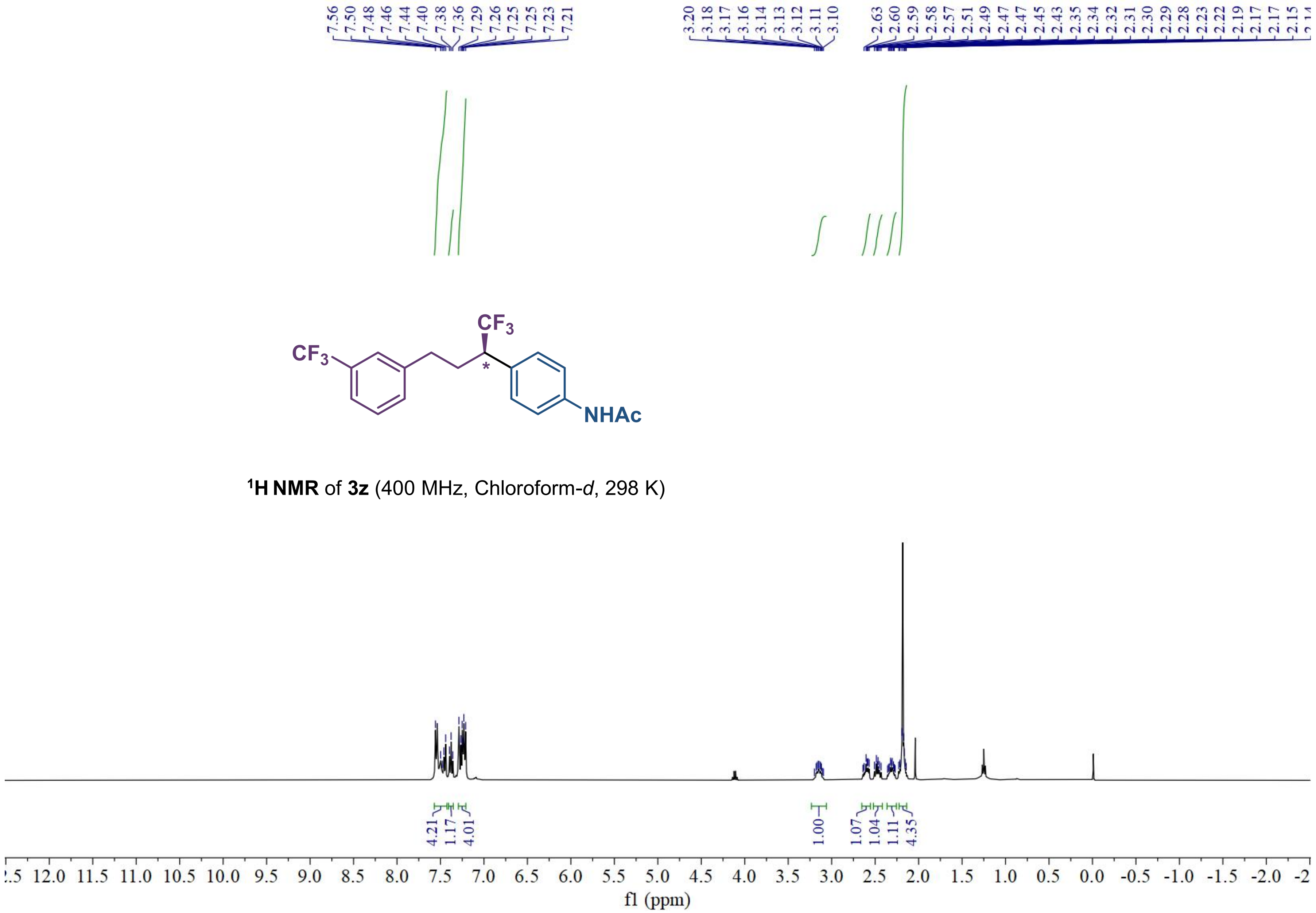

^{1}H NMR of **3z** (400 MHz, Chloroform-*d*, 298 K)

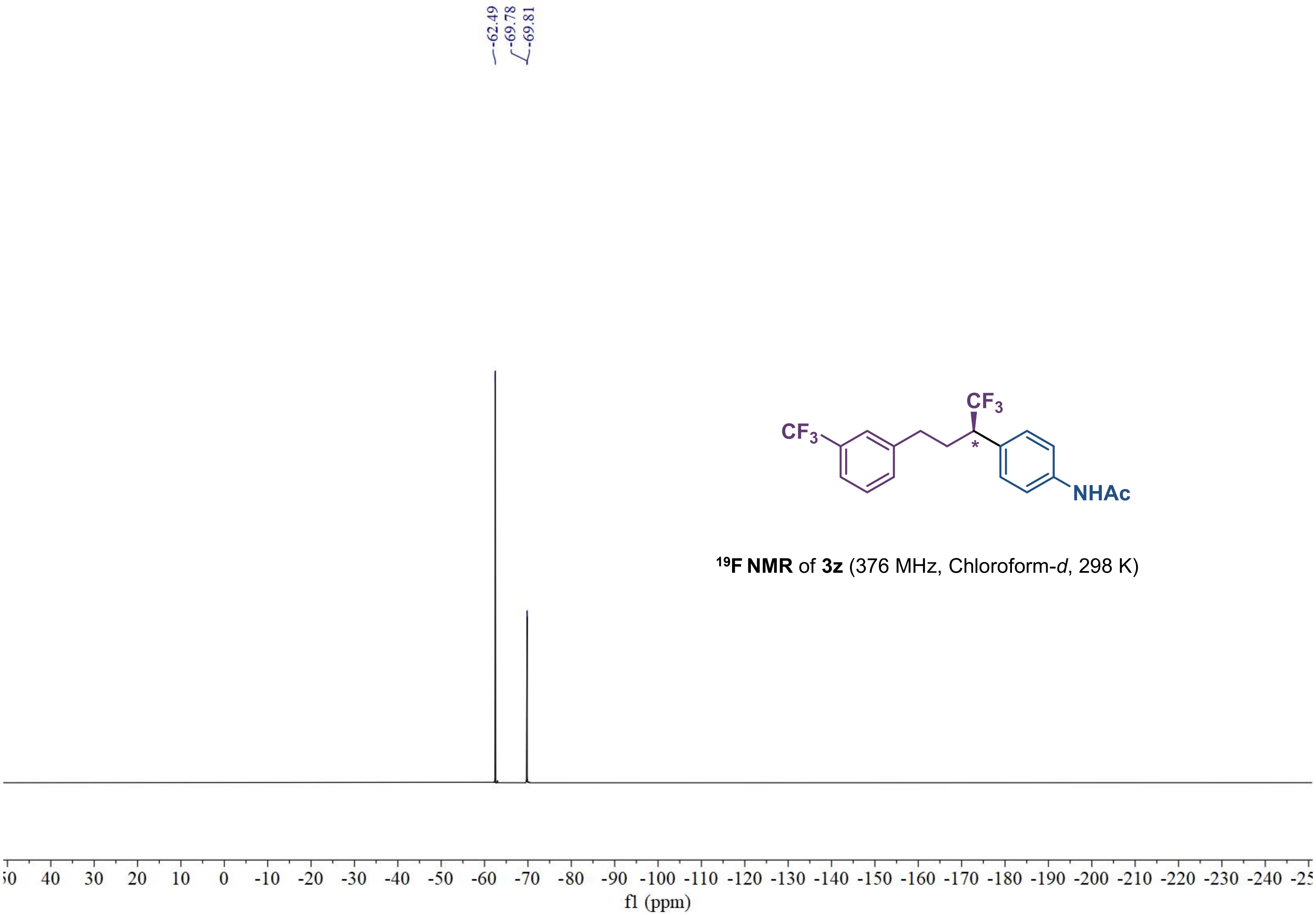

^{19}F NMR of **3z** (376 MHz, Chloroform-*d*, 298 K)

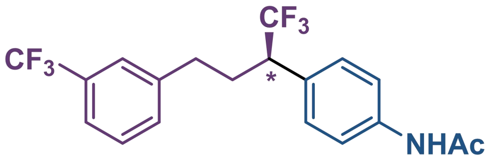

^{13}**C NMR** of **3z** (101 MHz, Chloroform-*d*, 298 K)

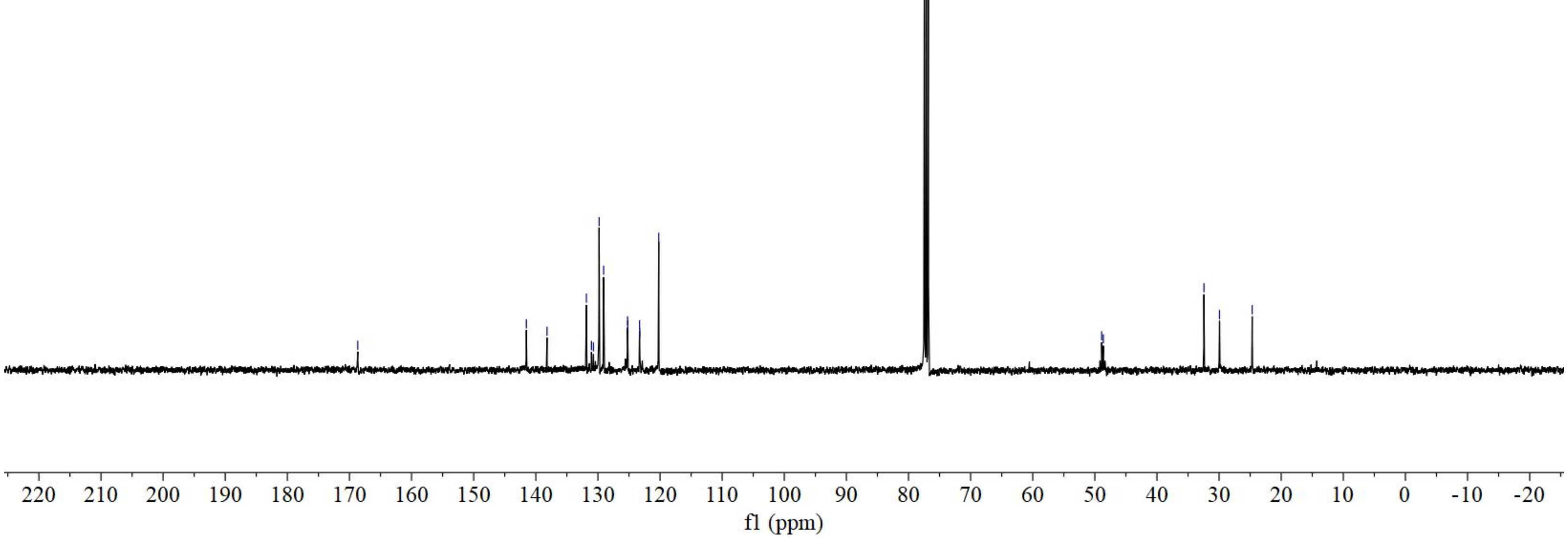

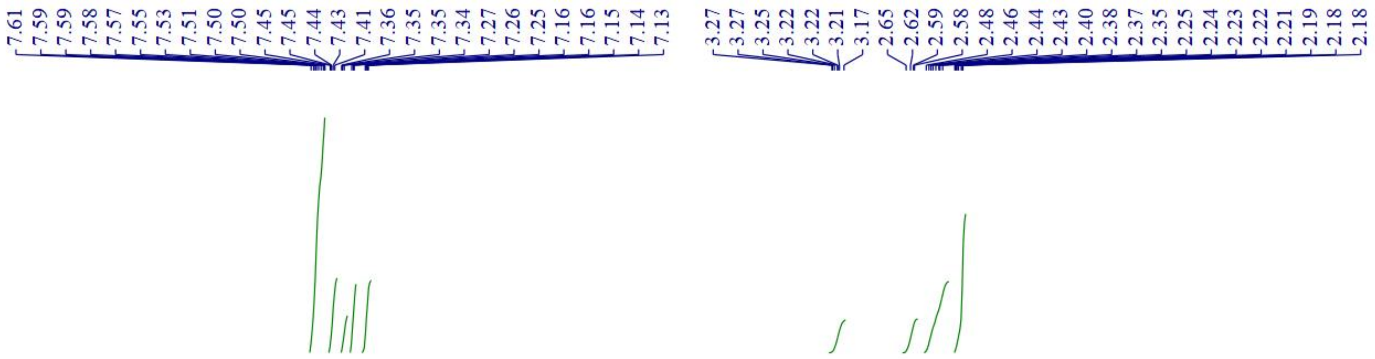

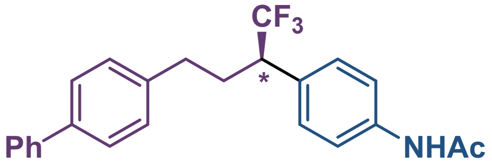

^{1}H NMR of **3aa** (400 MHz, Chloroform-*d*, 298 K)

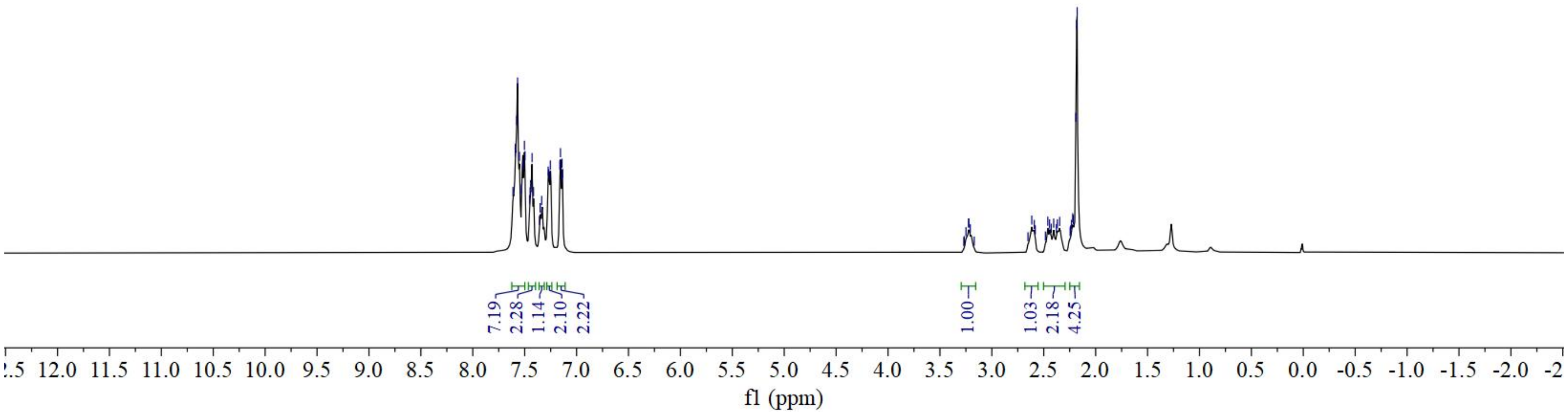

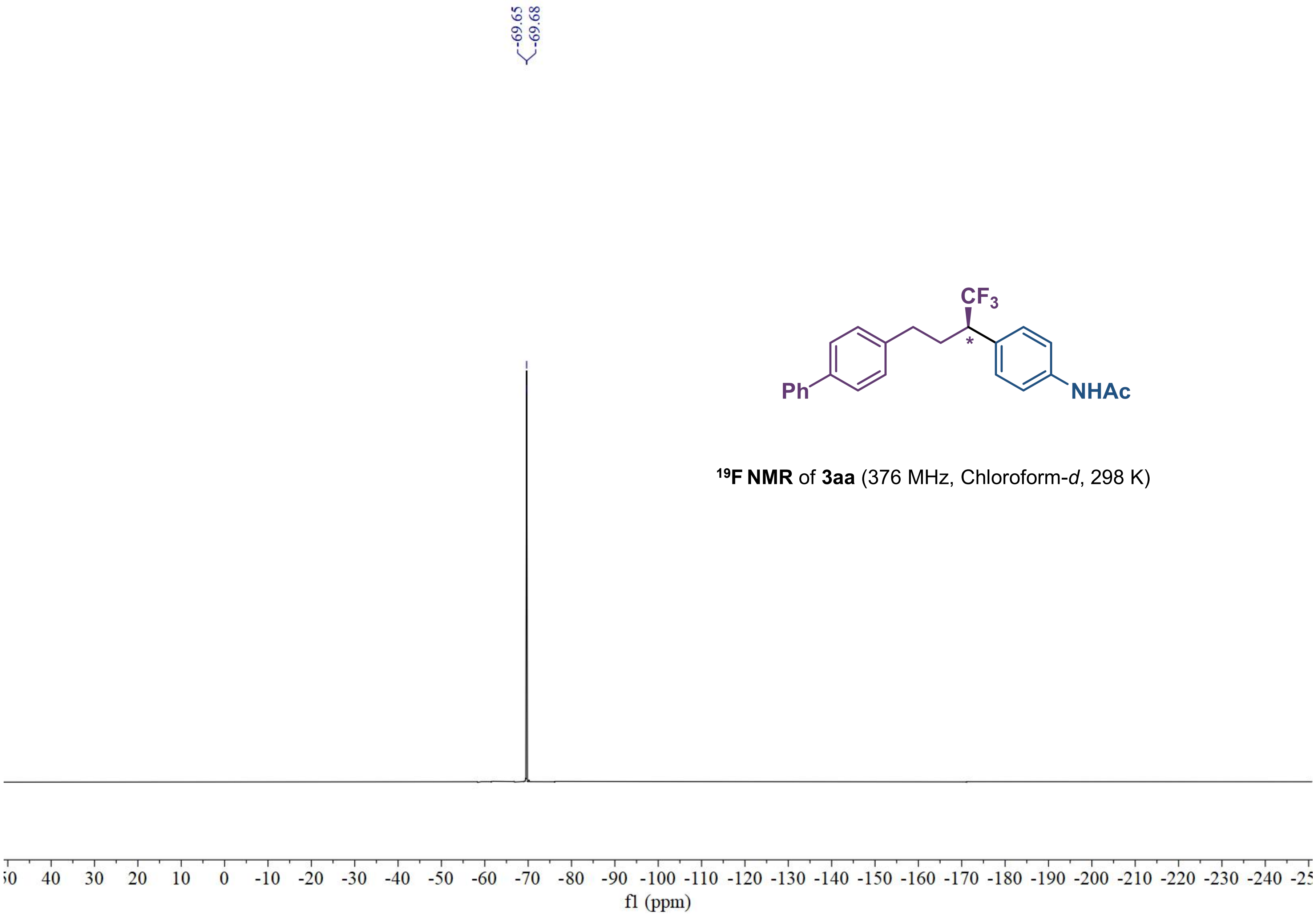

^{19}F NMR of **3aa** (376 MHz, Chloroform-*d*, 298 K)

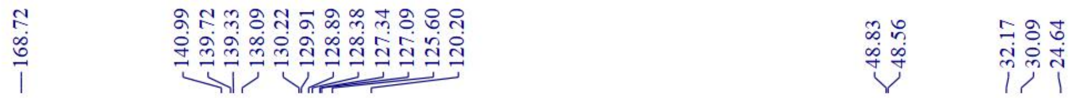

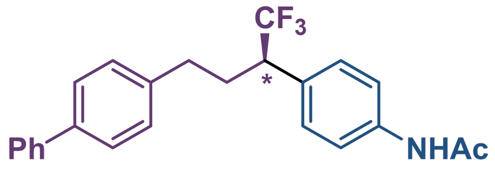

^{13}C NMR of **3aa** (101 MHz, Chloroform-*d*, 298 K)

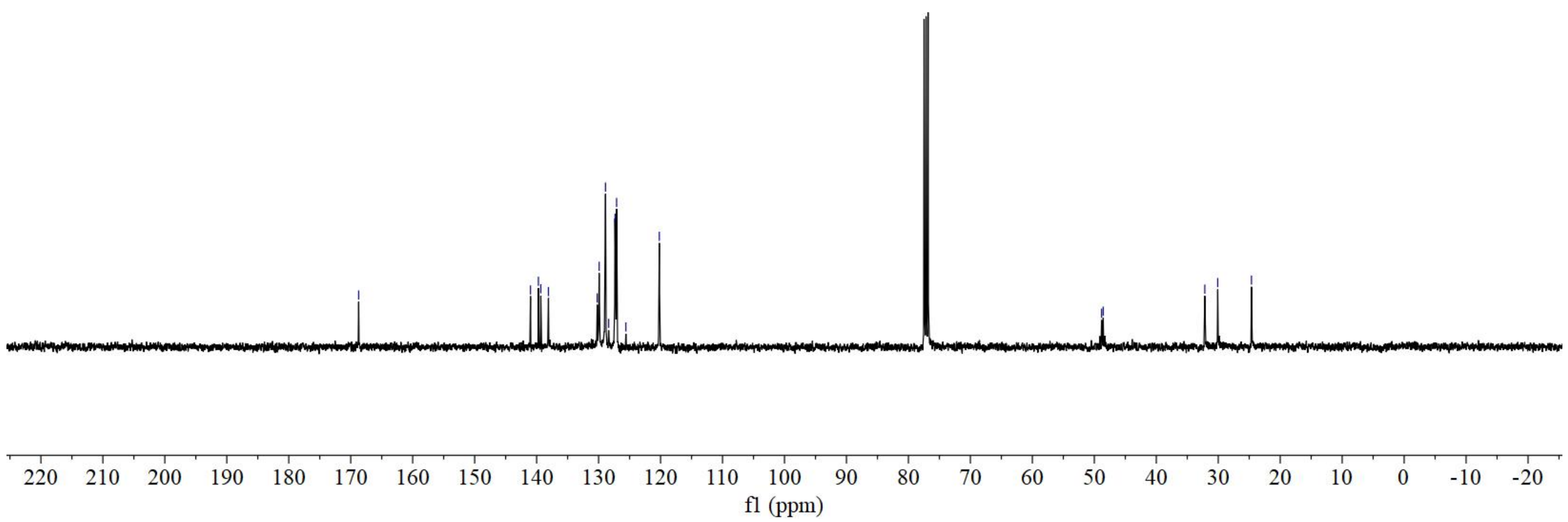

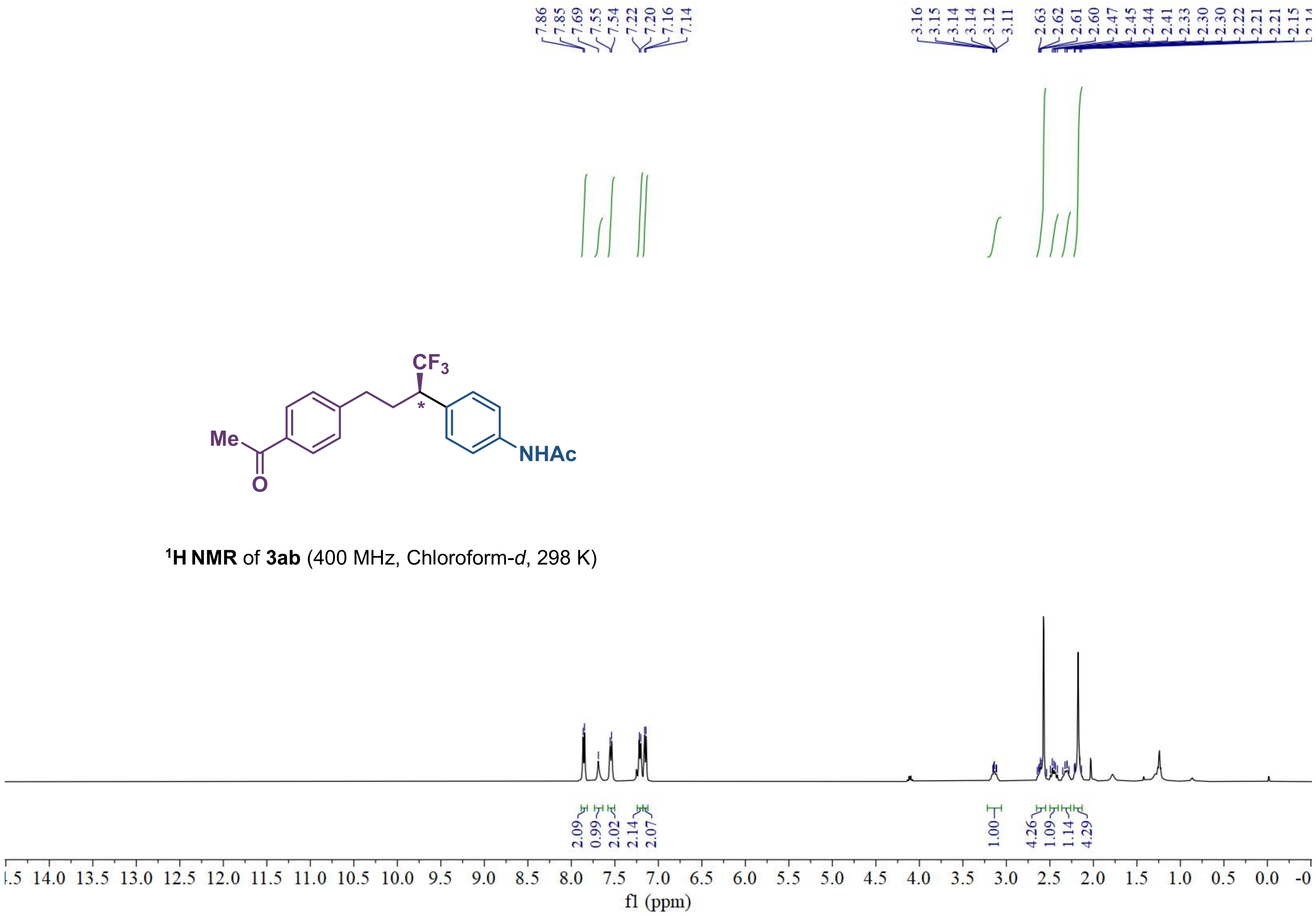

^{1}H NMR of **3ab** (400 MHz, Chloroform-*d*, 298 K)

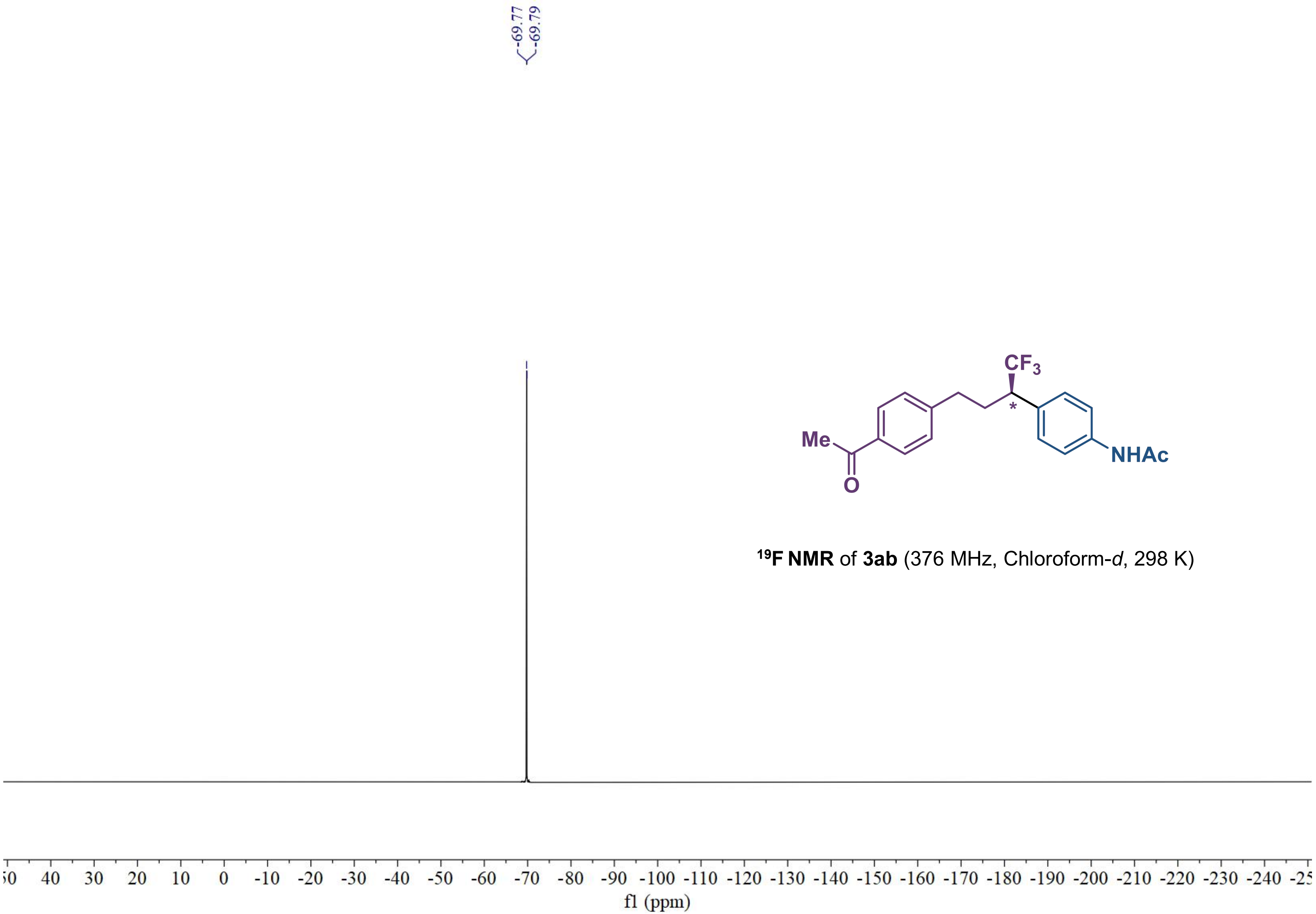

[19]**F NMR** of **3ab** (376 MHz, Chloroform-*d*, 298 K)

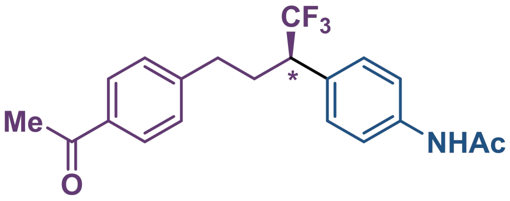

^{13}**C NMR** of **3ab** (101 MHz, Chloroform-*d*, 298 K)

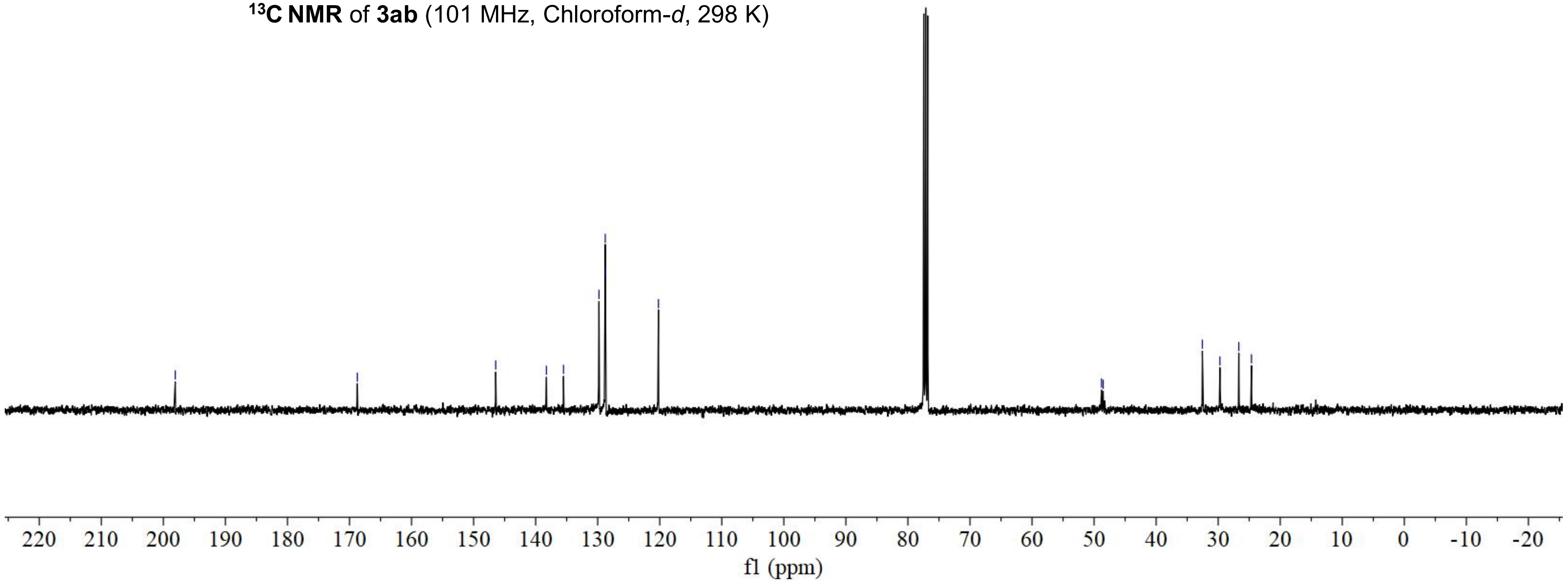

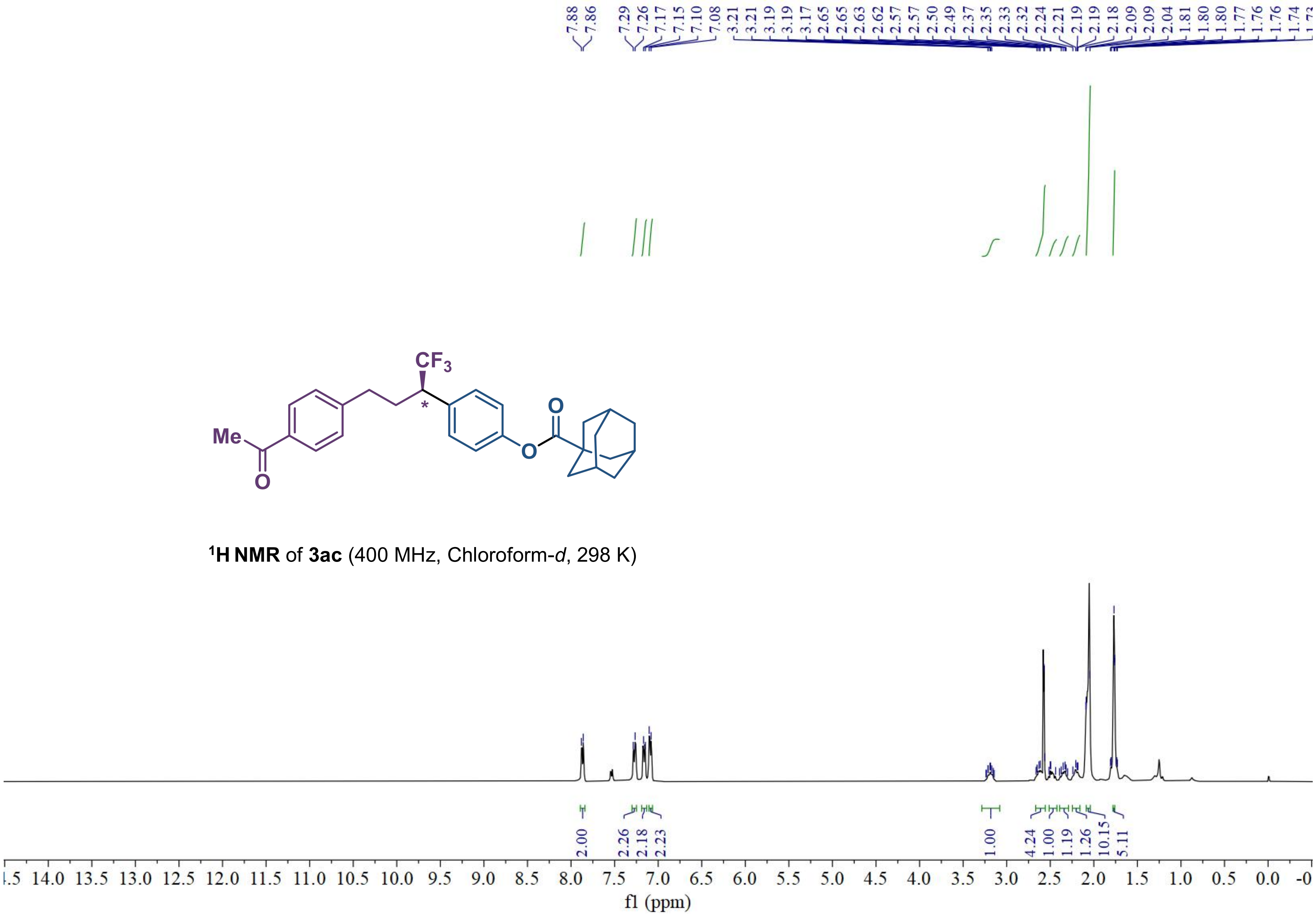

^{1}H NMR of **3ac** (400 MHz, Chloroform-*d*, 298 K)

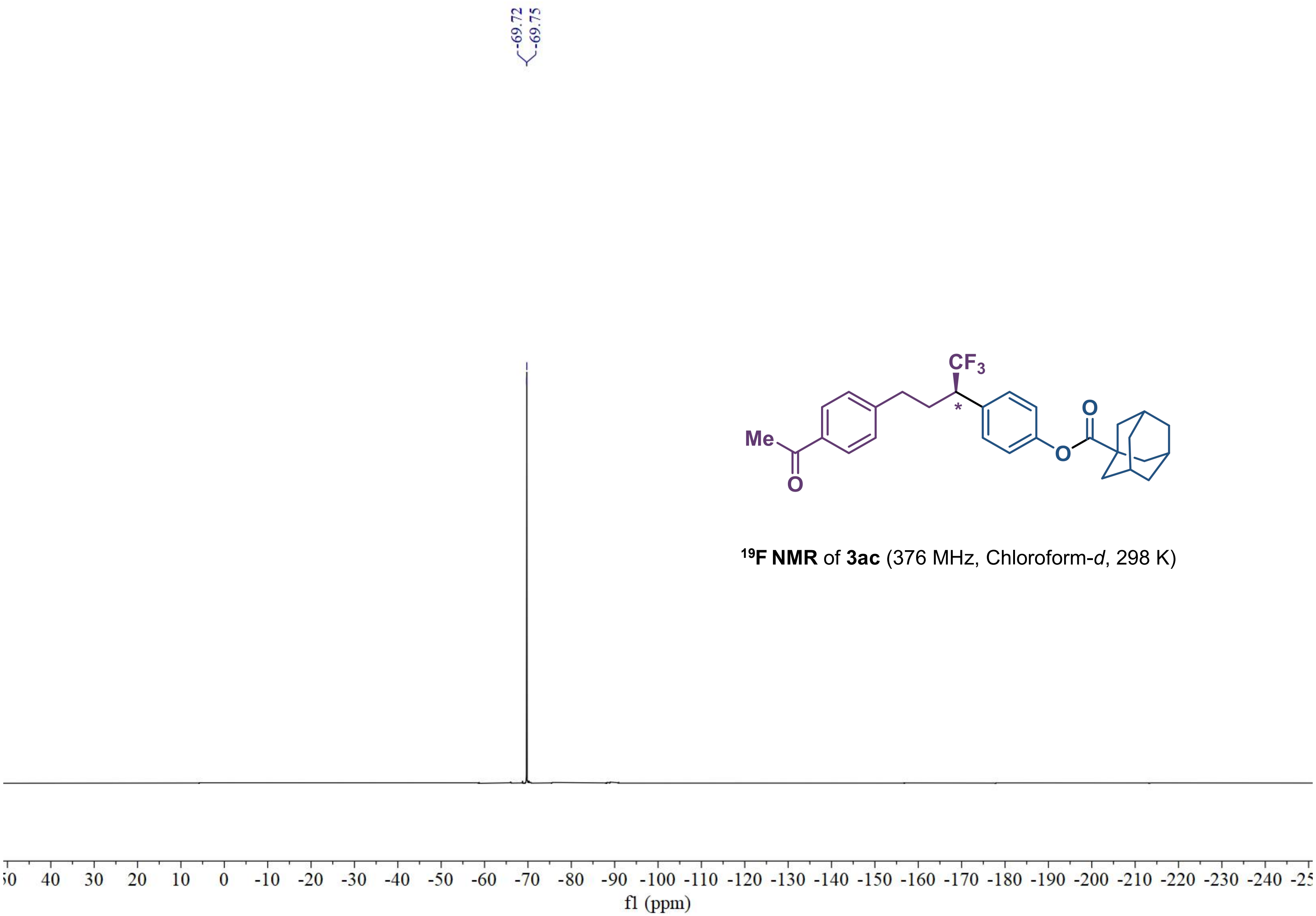

^{19}F NMR of **3ac** (376 MHz, Chloroform-*d*, 298 K)

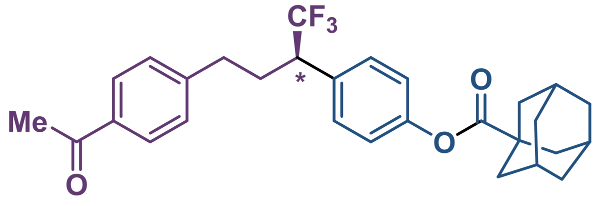

^{13}C NMR of **3ac** (101 MHz, Chloroform-*d*, 298 K)

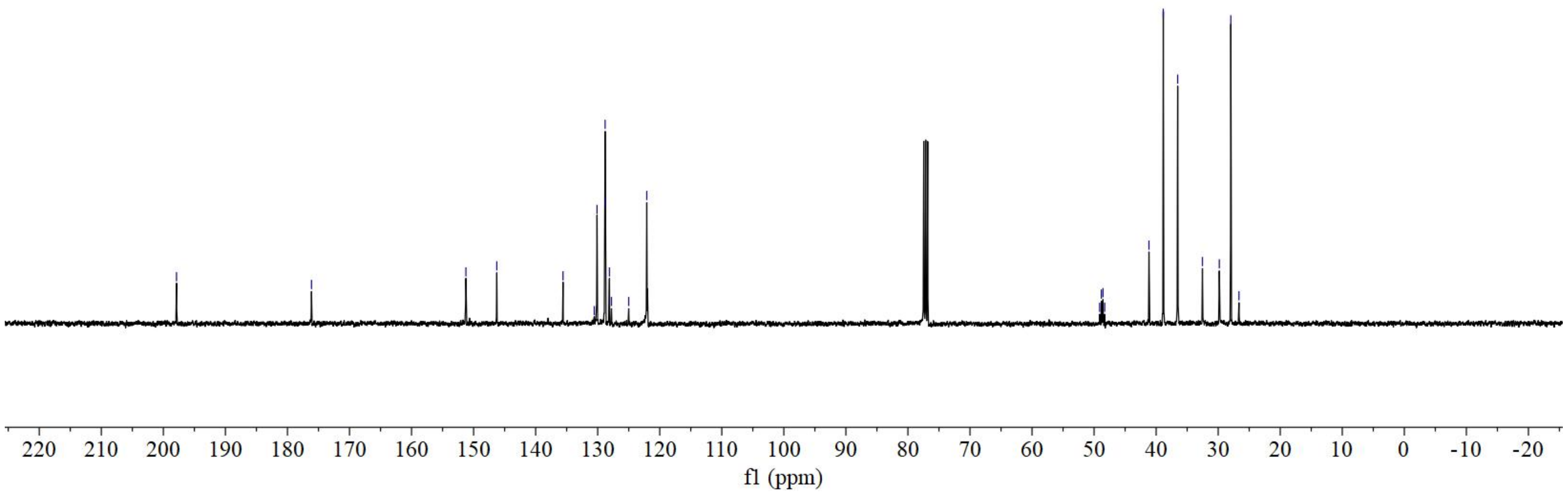

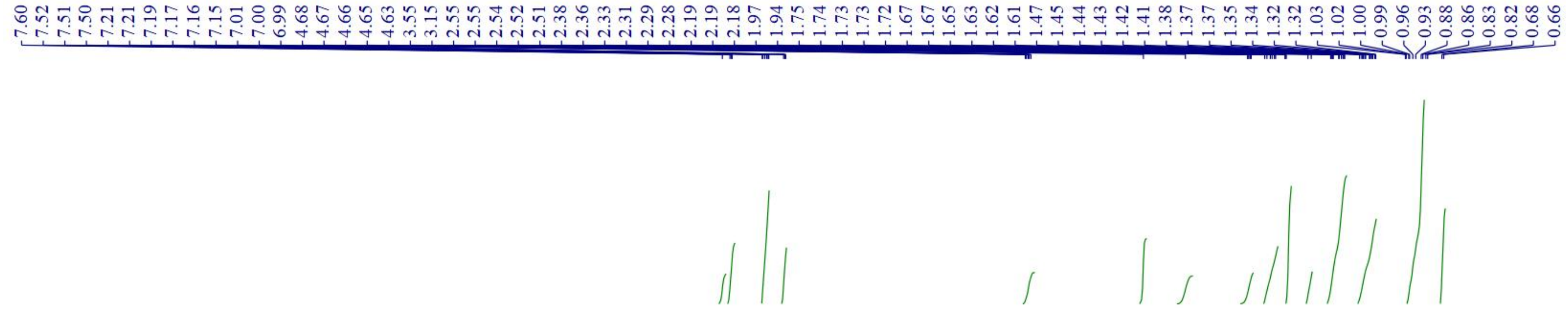

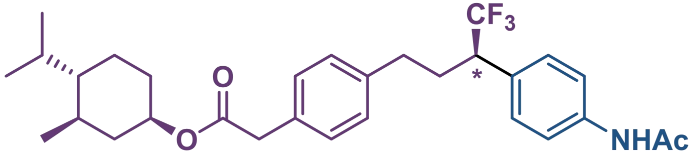

¹H NMR of **3ad** (400 MHz, Chloroform-*d*, 298 K)

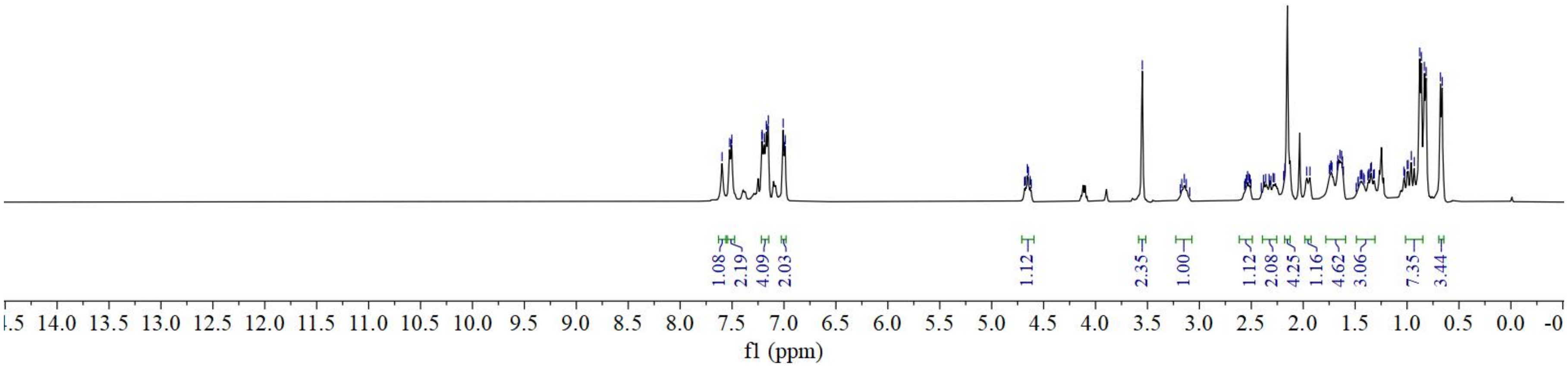

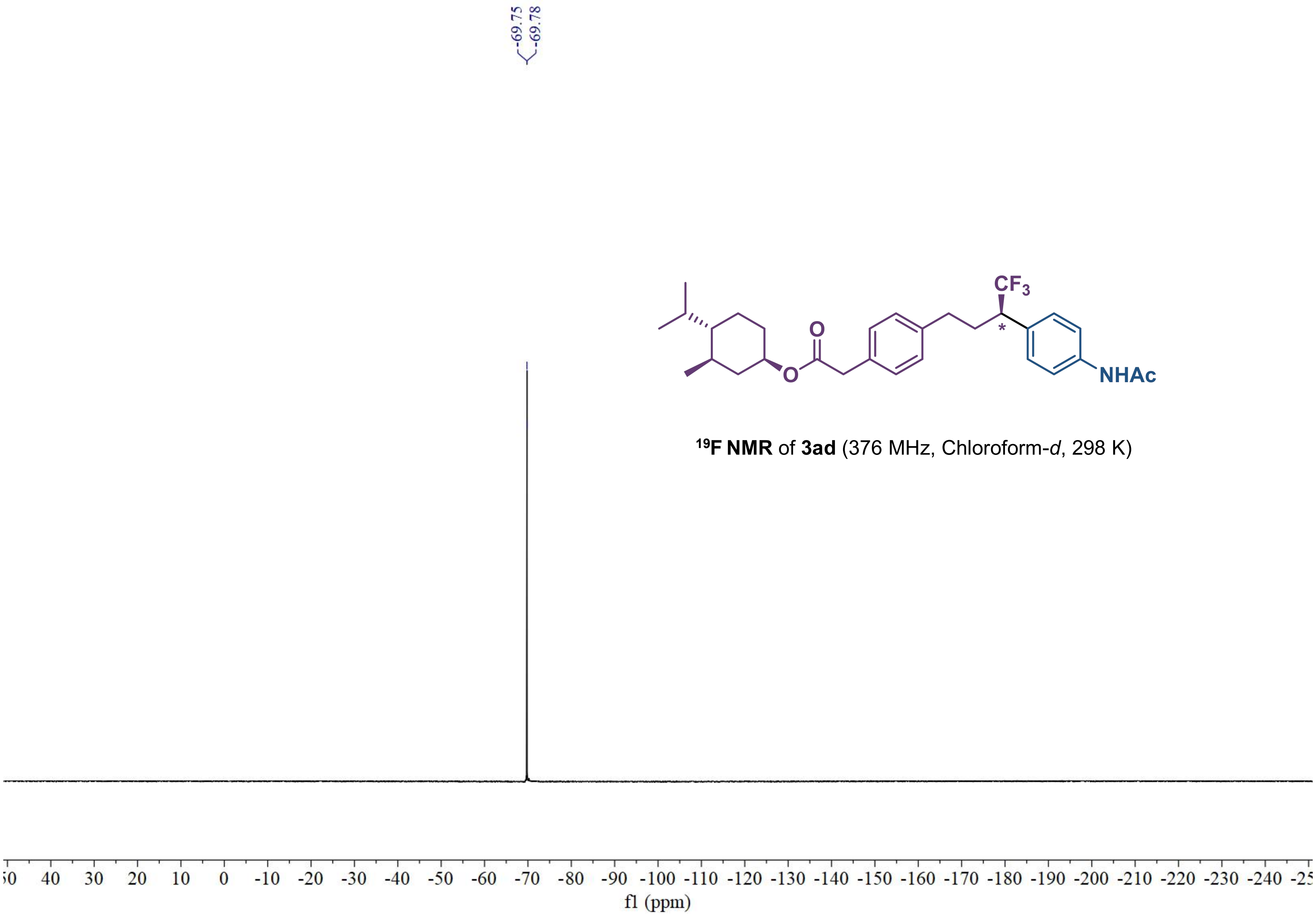

^{19}F NMR of **3ad** (376 MHz, Chloroform-*d*, 298 K)

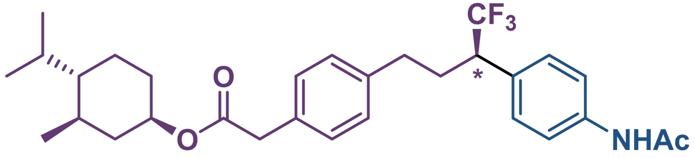

^{13}C NMR of **3ad** (101 MHz, Chloroform-*d*, 298 K)

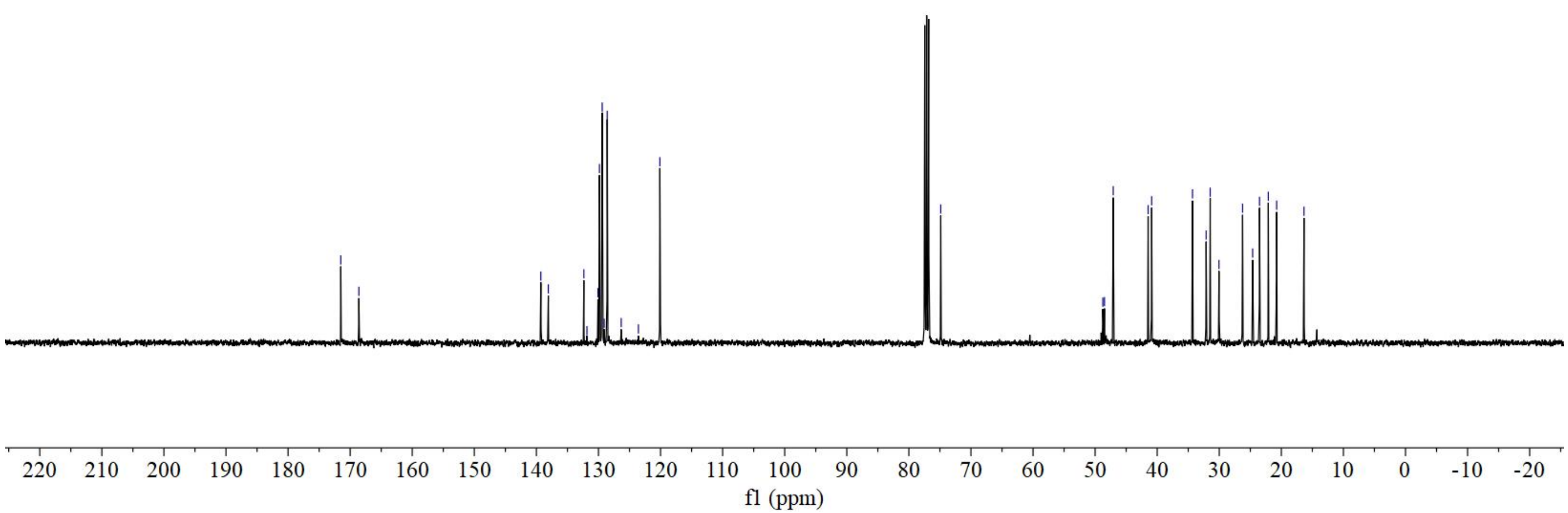

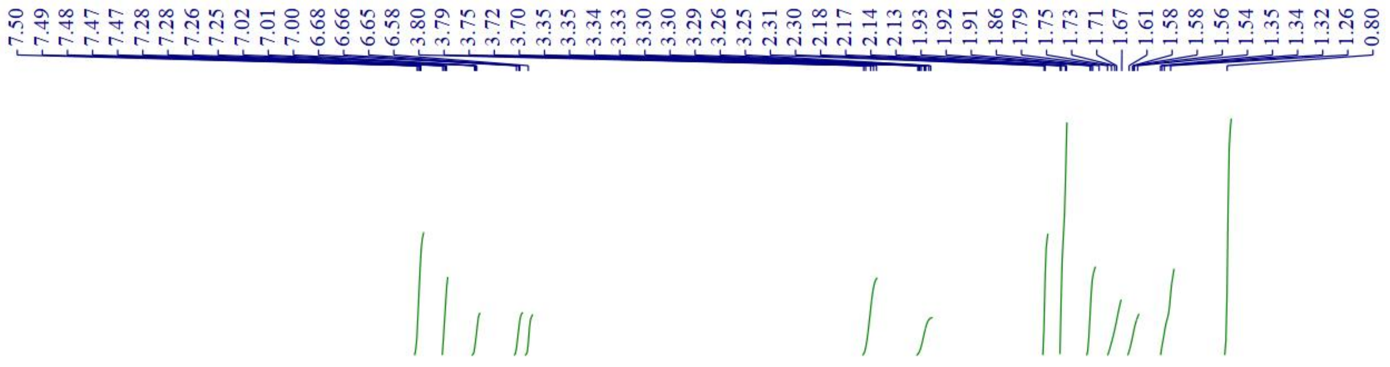

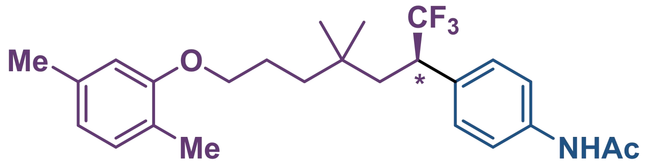

^{1}**H NMR** of **3ae** (400 MHz, Chloroform-*d*, 298 K)

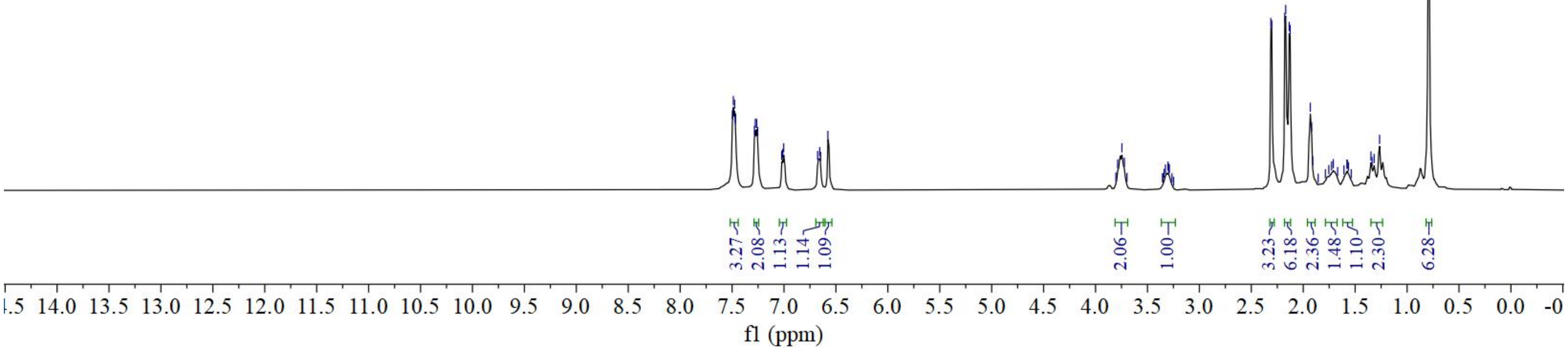

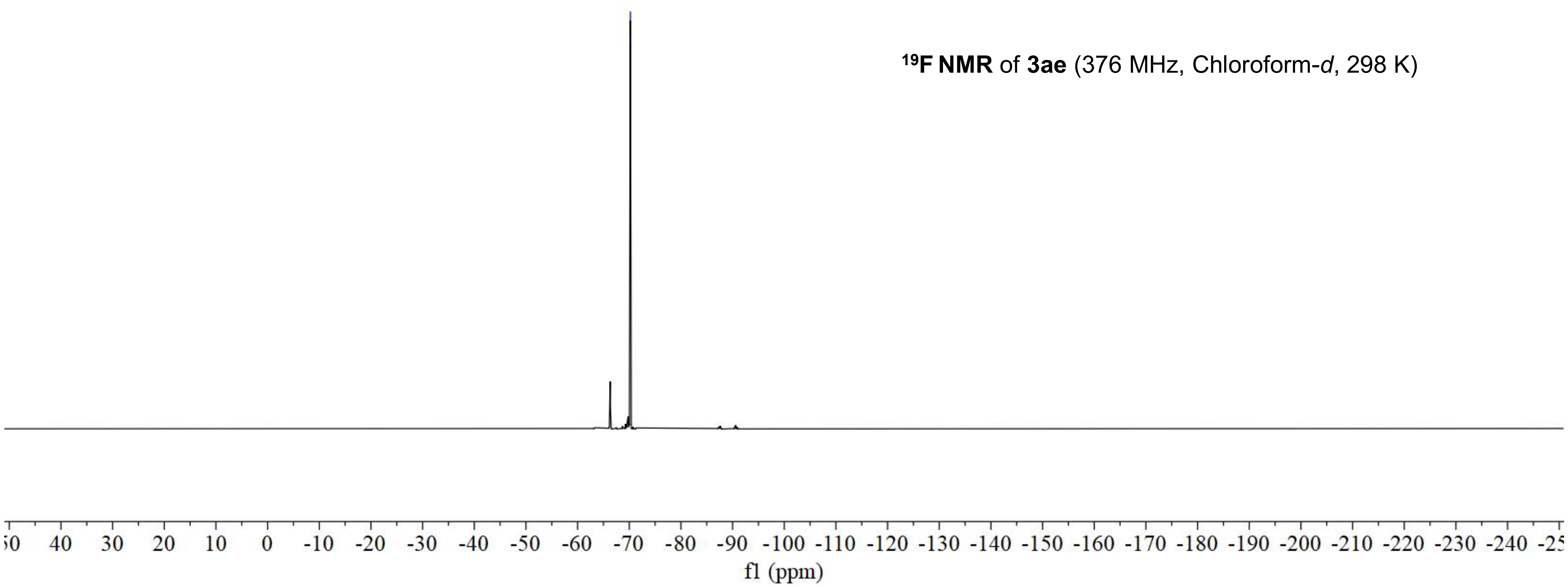

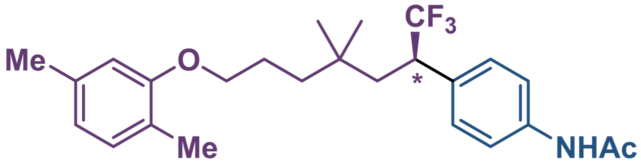

^{19}F NMR of **3ae** (376 MHz, Chloroform-*d*, 298 K)

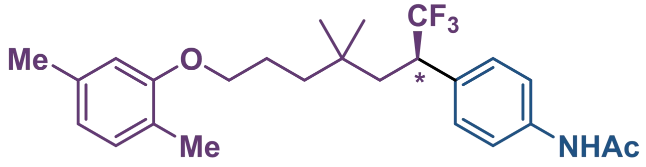

^{13}C NMR of **3ae** (101 MHz, Chloroform-*d*, 298 K)

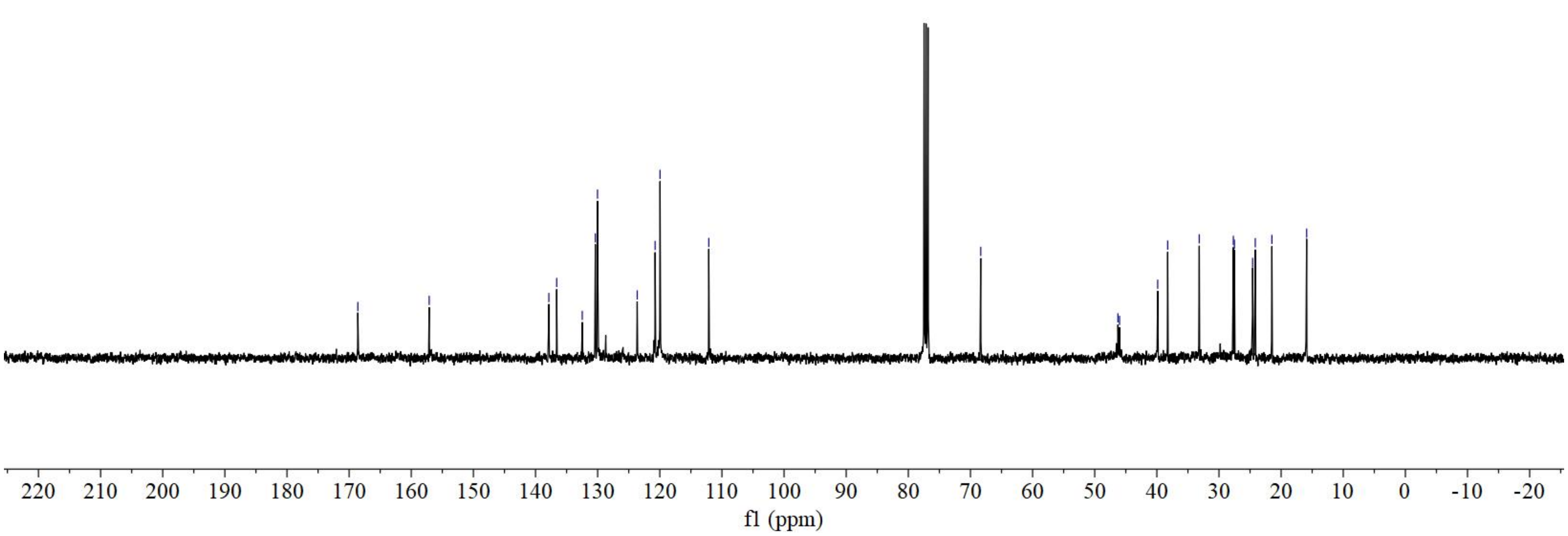

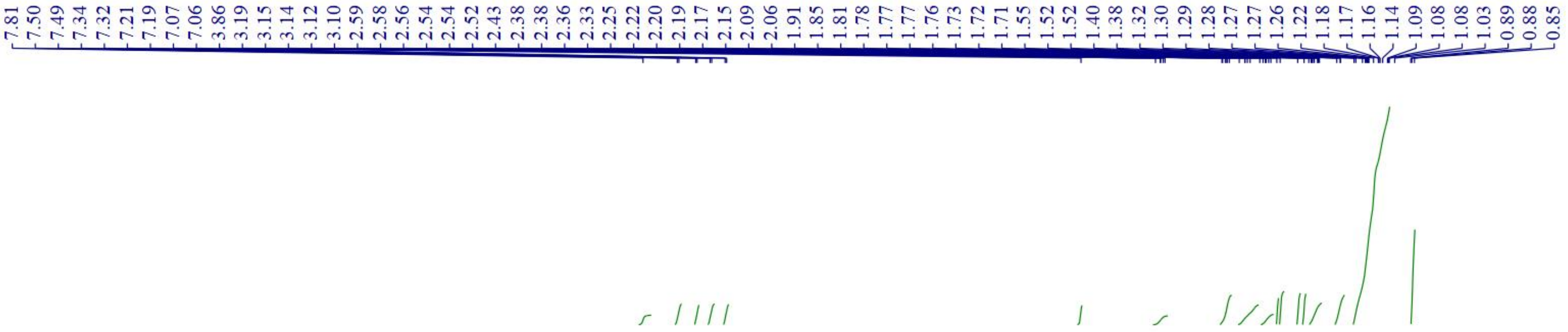

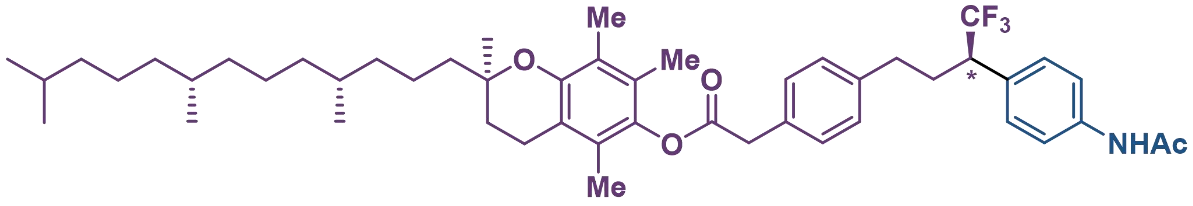

^{1}H NMR of **3af** (400 MHz, Chloroform-*d*, 298 K)

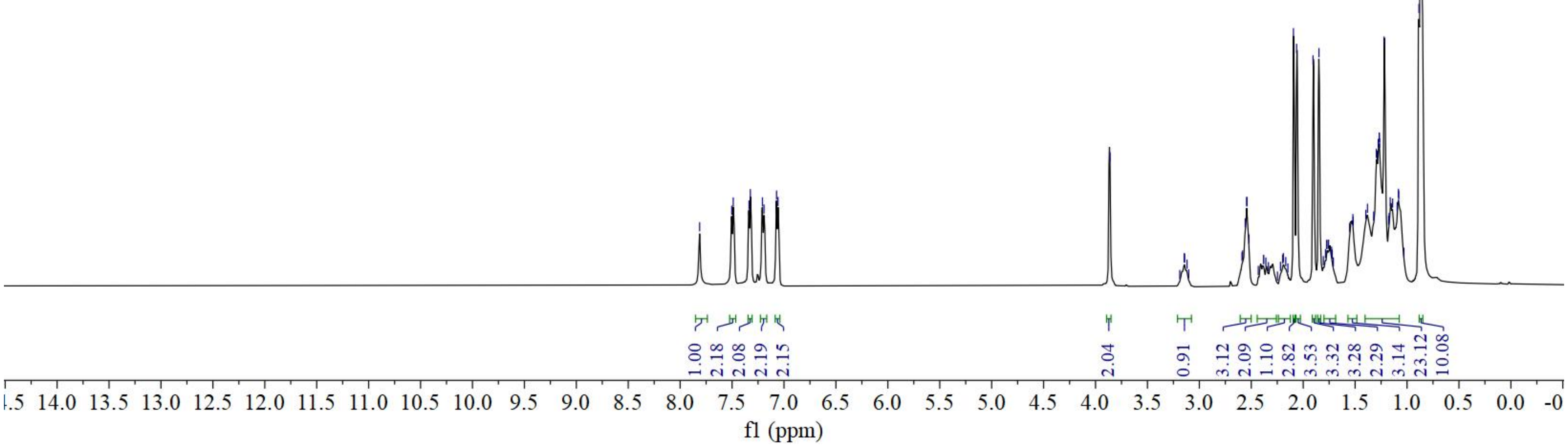

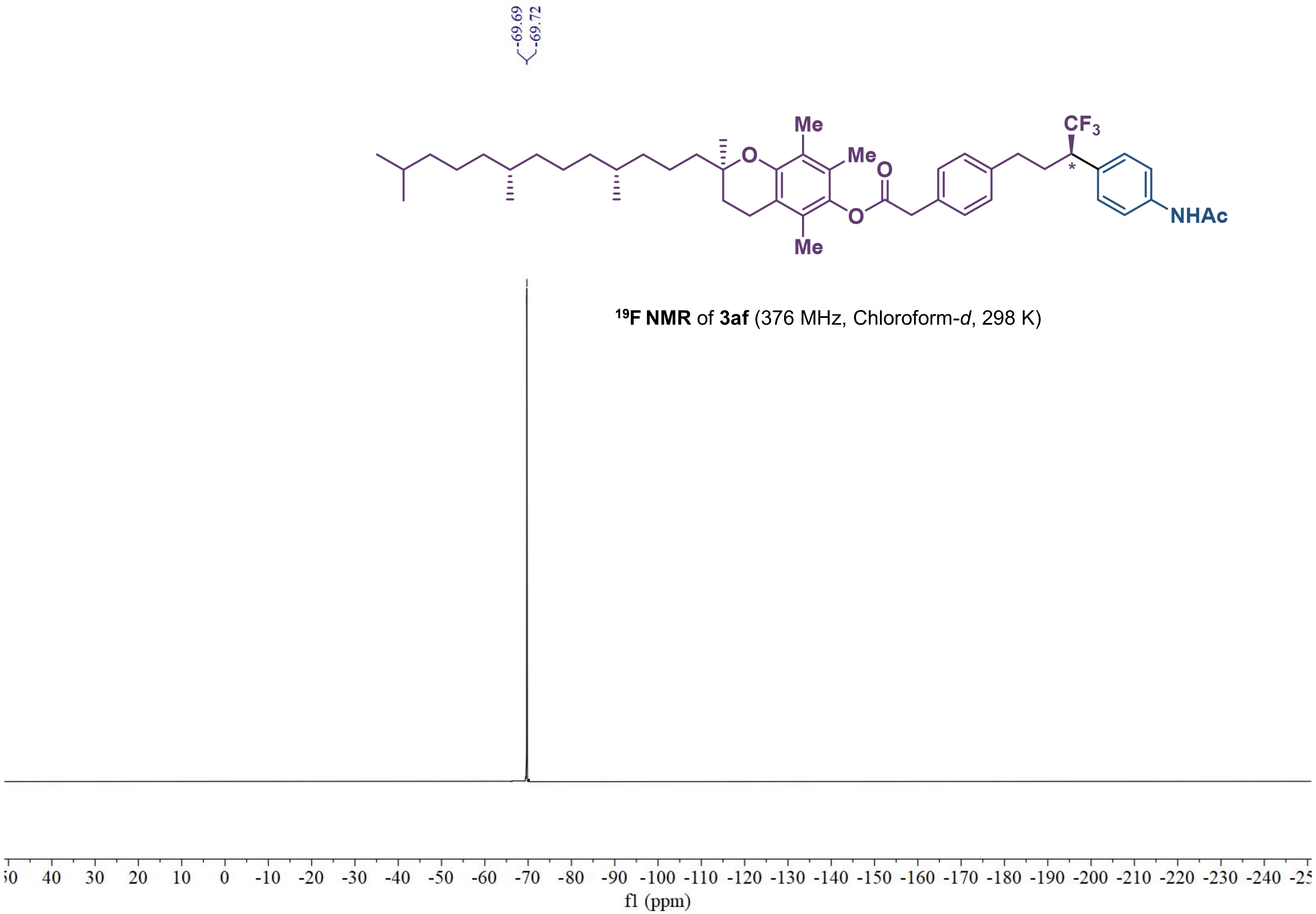

^{19}F NMR of **3af** (376 MHz, Chloroform-*d*, 298 K)

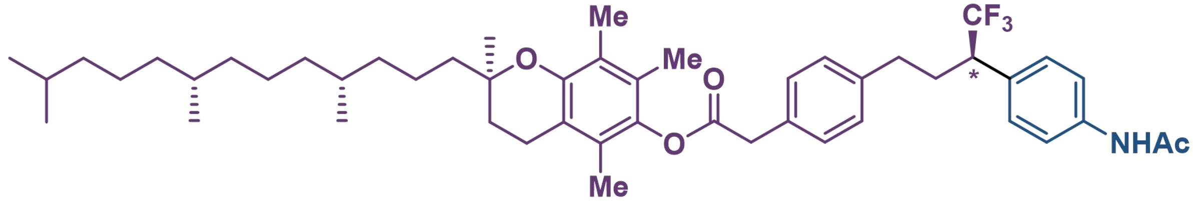

^{13}C NMR of **3af** (101 MHz, Chloroform-*d*, 298 K)

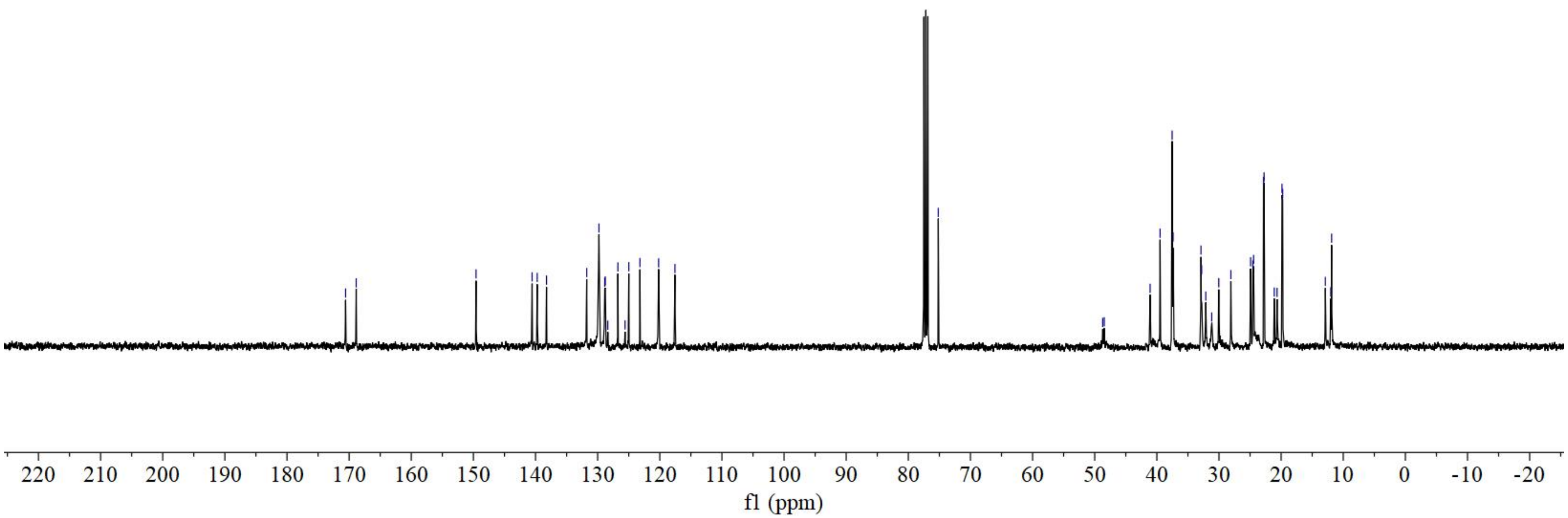

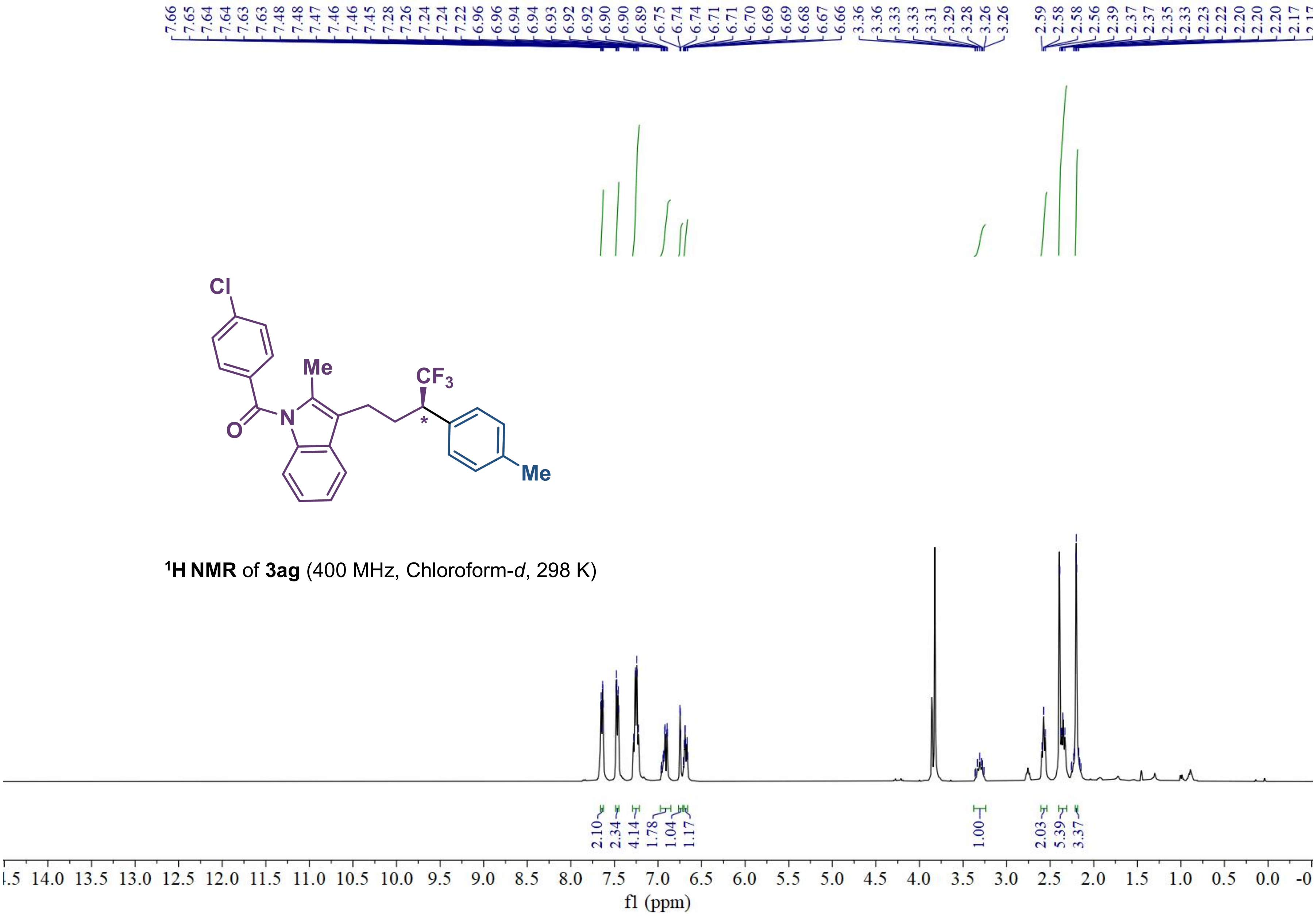

¹H NMR of **3ag** (400 MHz, Chloroform-*d*, 298 K)

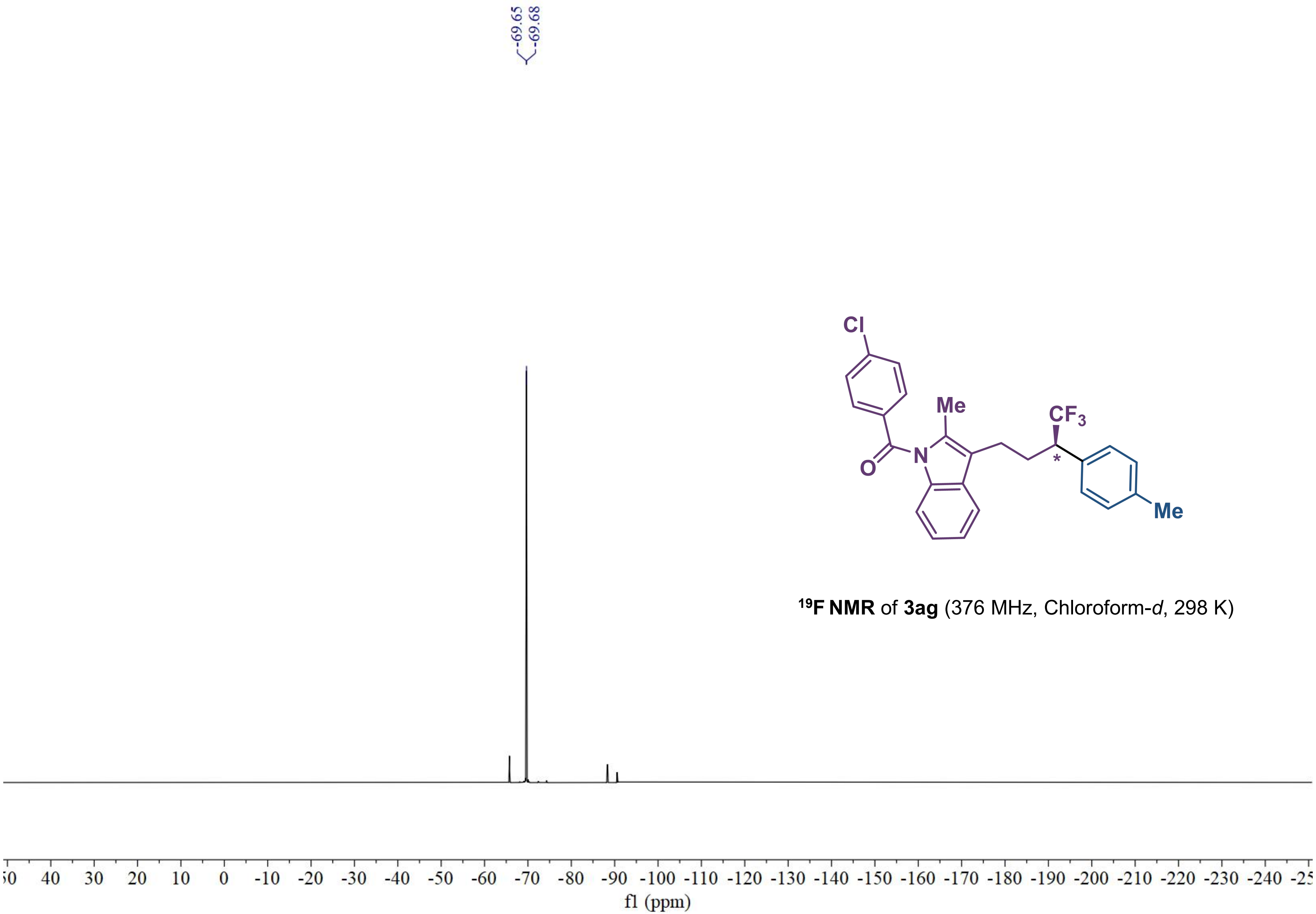

^{19}**F NMR** of **3ag** (376 MHz, Chloroform-*d*, 298 K)

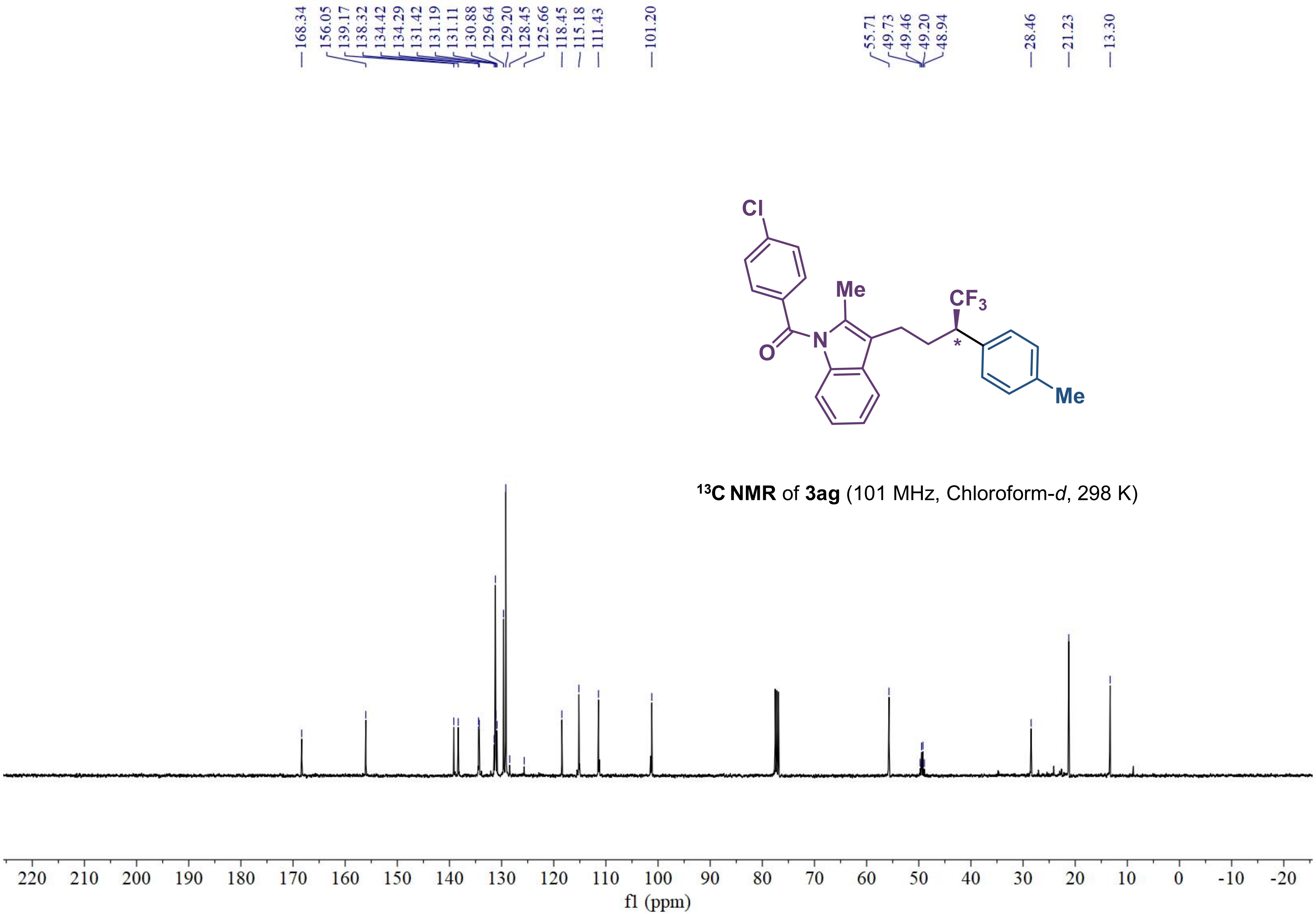

^{13}**C NMR** of **3ag** (101 MHz, Chloroform-*d*, 298 K)

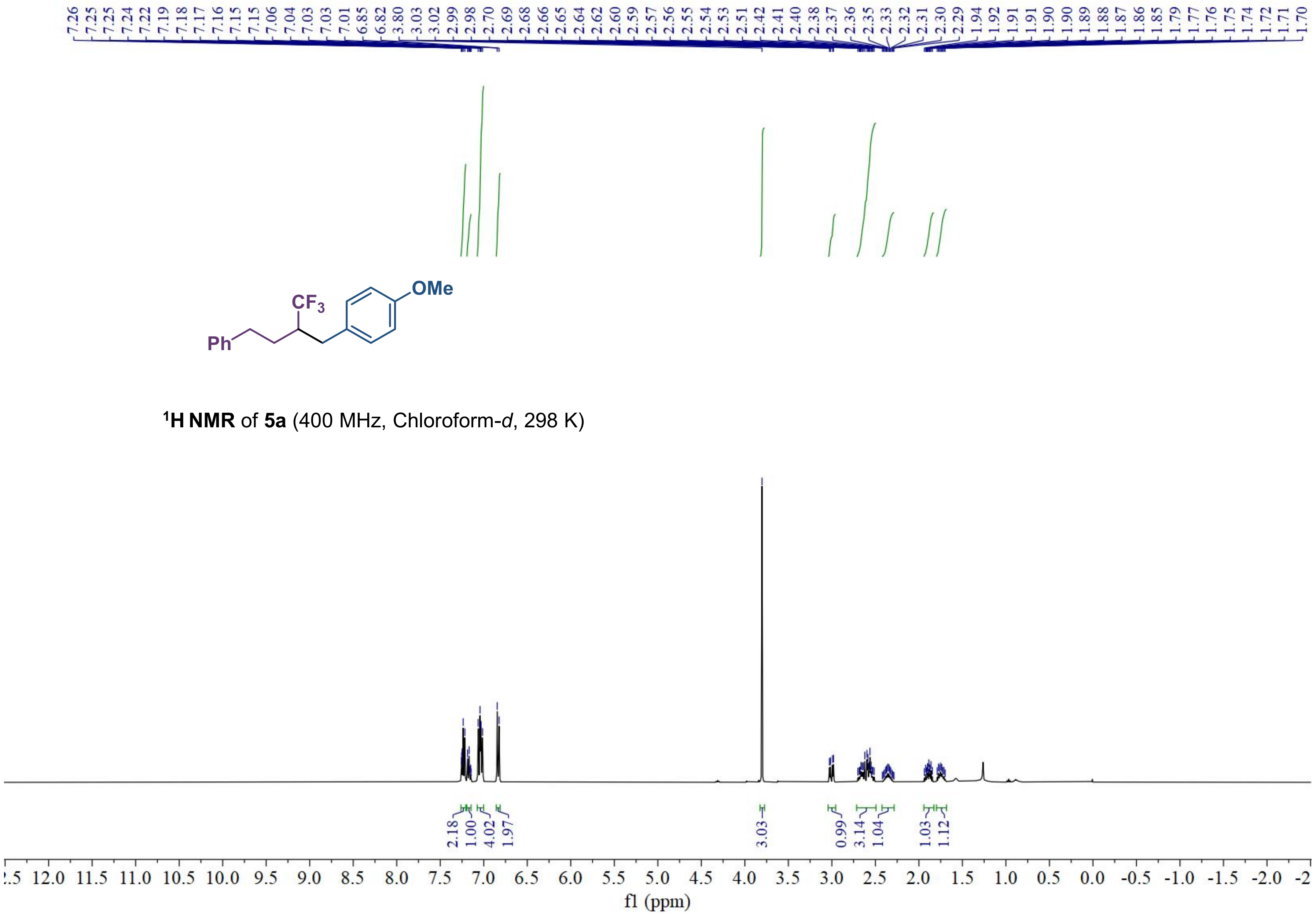

^{1}H NMR of **5a** (400 MHz, Chloroform-*d*, 298 K)

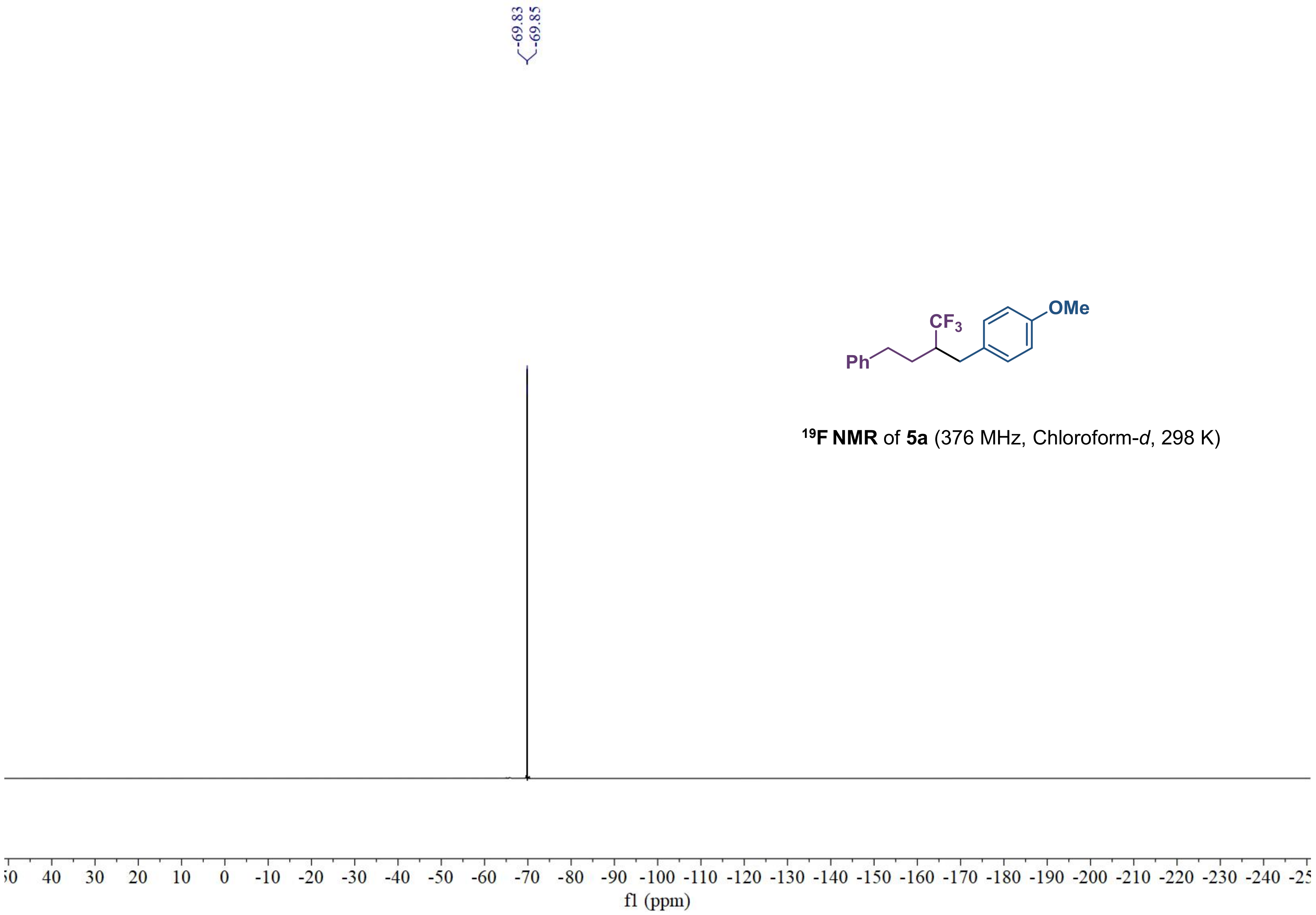

^{19}F NMR of **5a** (376 MHz, Chloroform-*d*, 298 K)

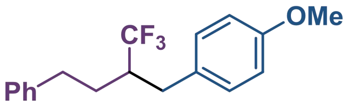

^{13}C NMR of **5a** (101 MHz, Chloroform-*d*, 298 K)

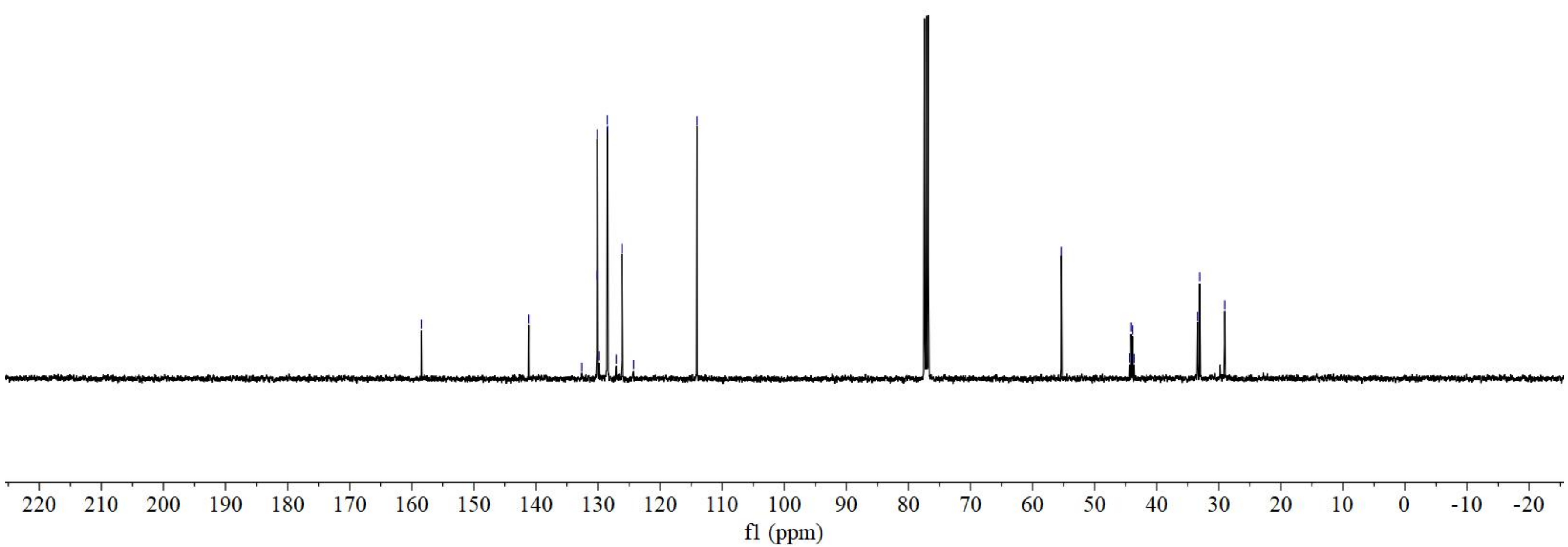

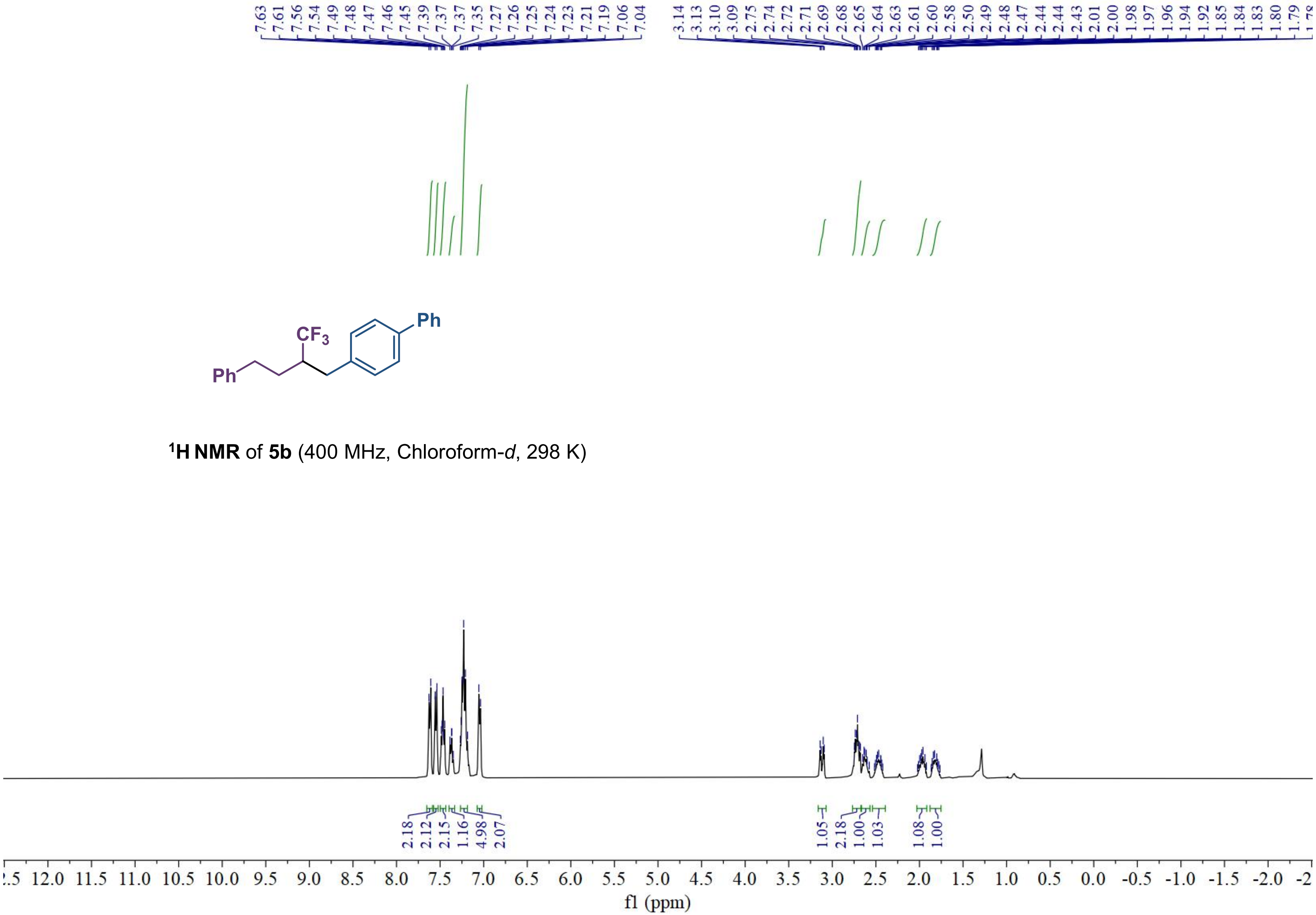

^{1}H NMR of **5b** (400 MHz, Chloroform-*d*, 298 K)

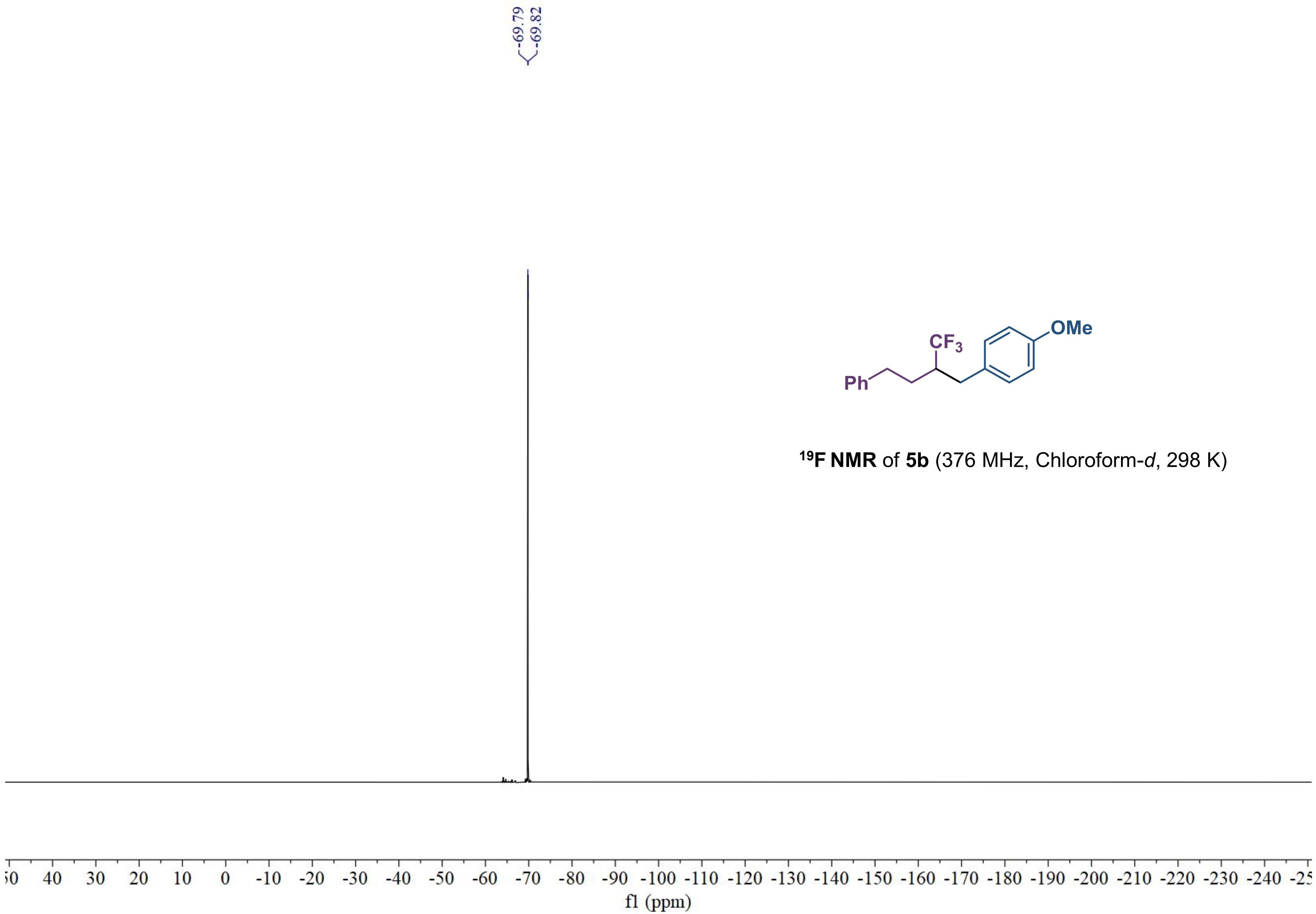

^{19}F NMR of **5b** (376 MHz, Chloroform-*d*, 298 K)

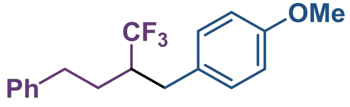

^{13}C NMR of **5b** (101 MHz, Chloroform-*d*, 298 K)

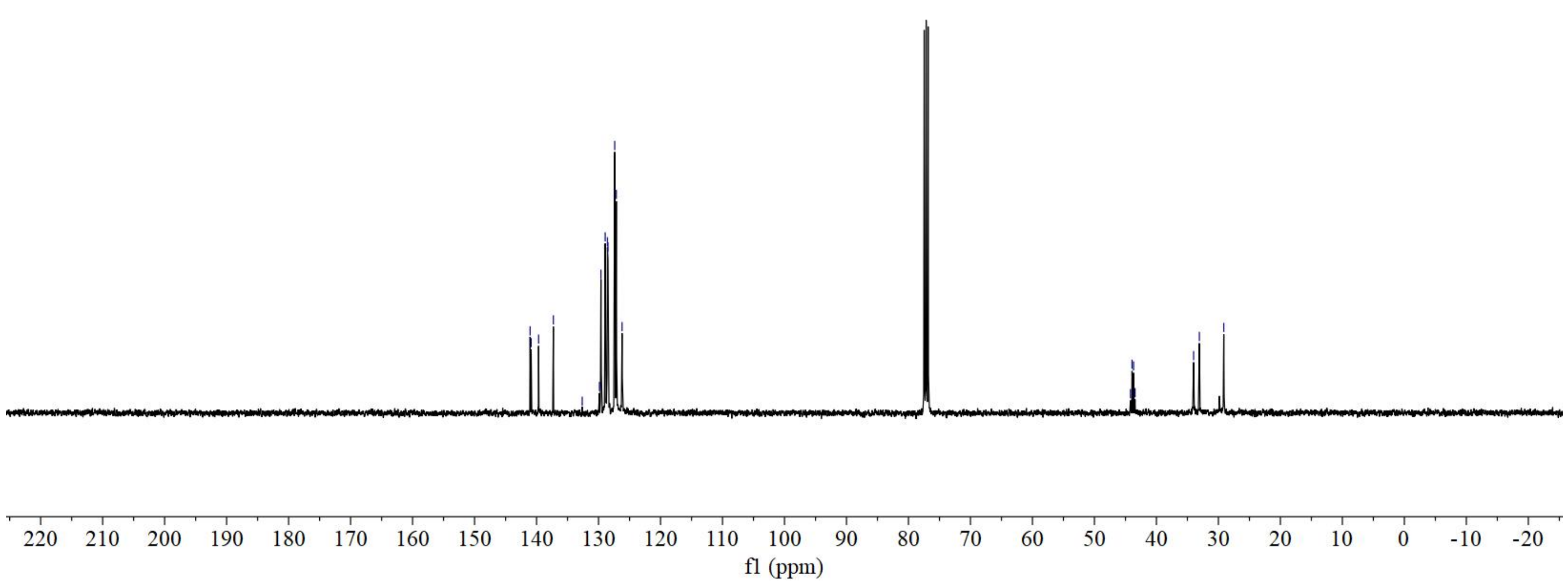

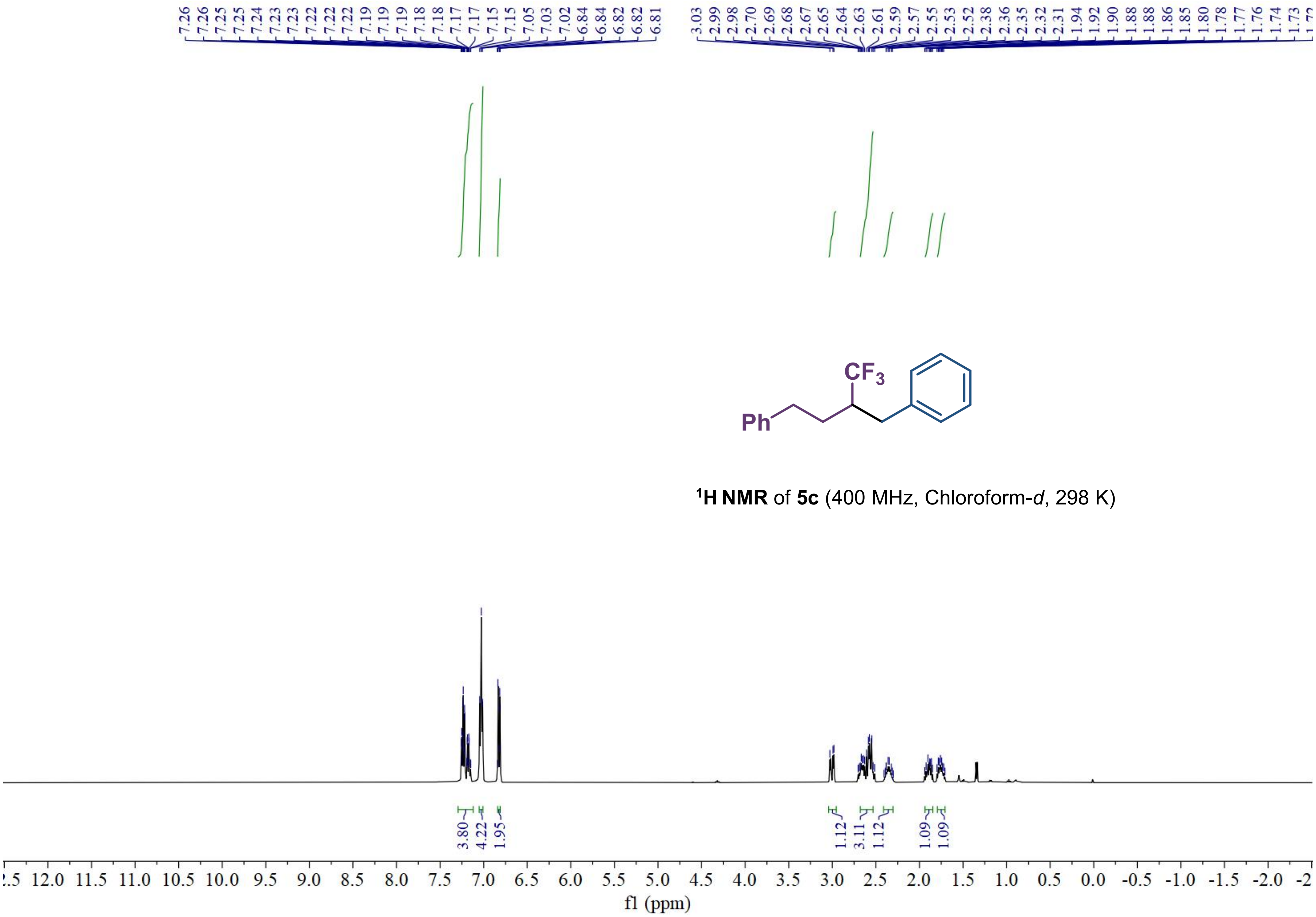

¹H NMR of **5c** (400 MHz, Chloroform-*d*, 298 K)

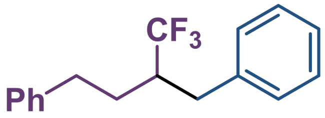

13C NMR of **5c** (100 MHz, Chloroform-*d*, 298 K)

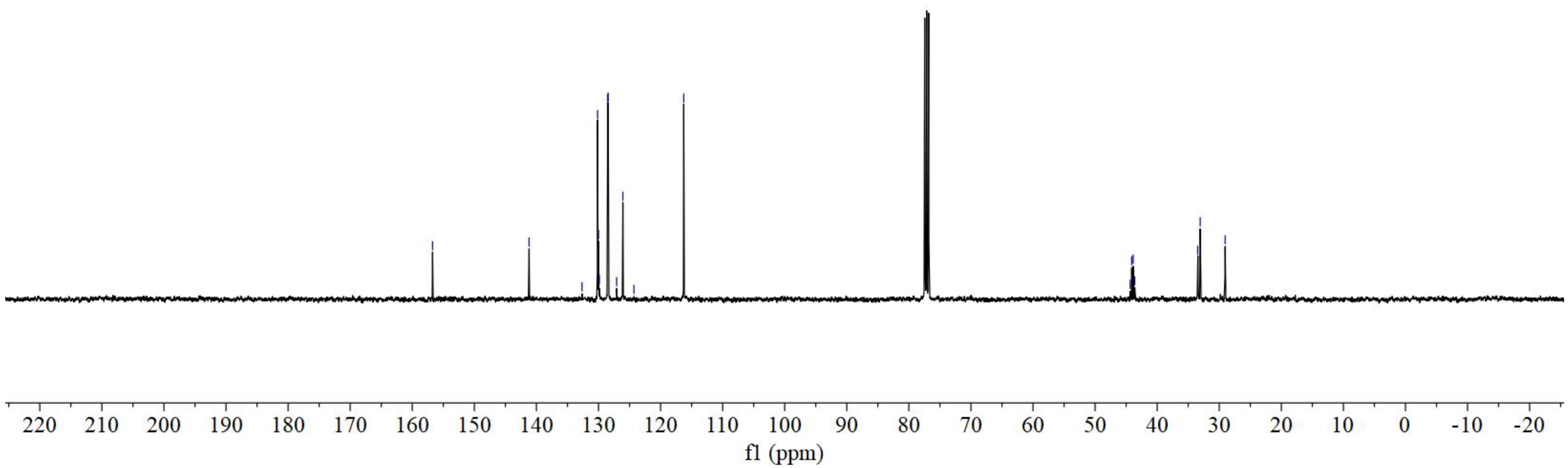

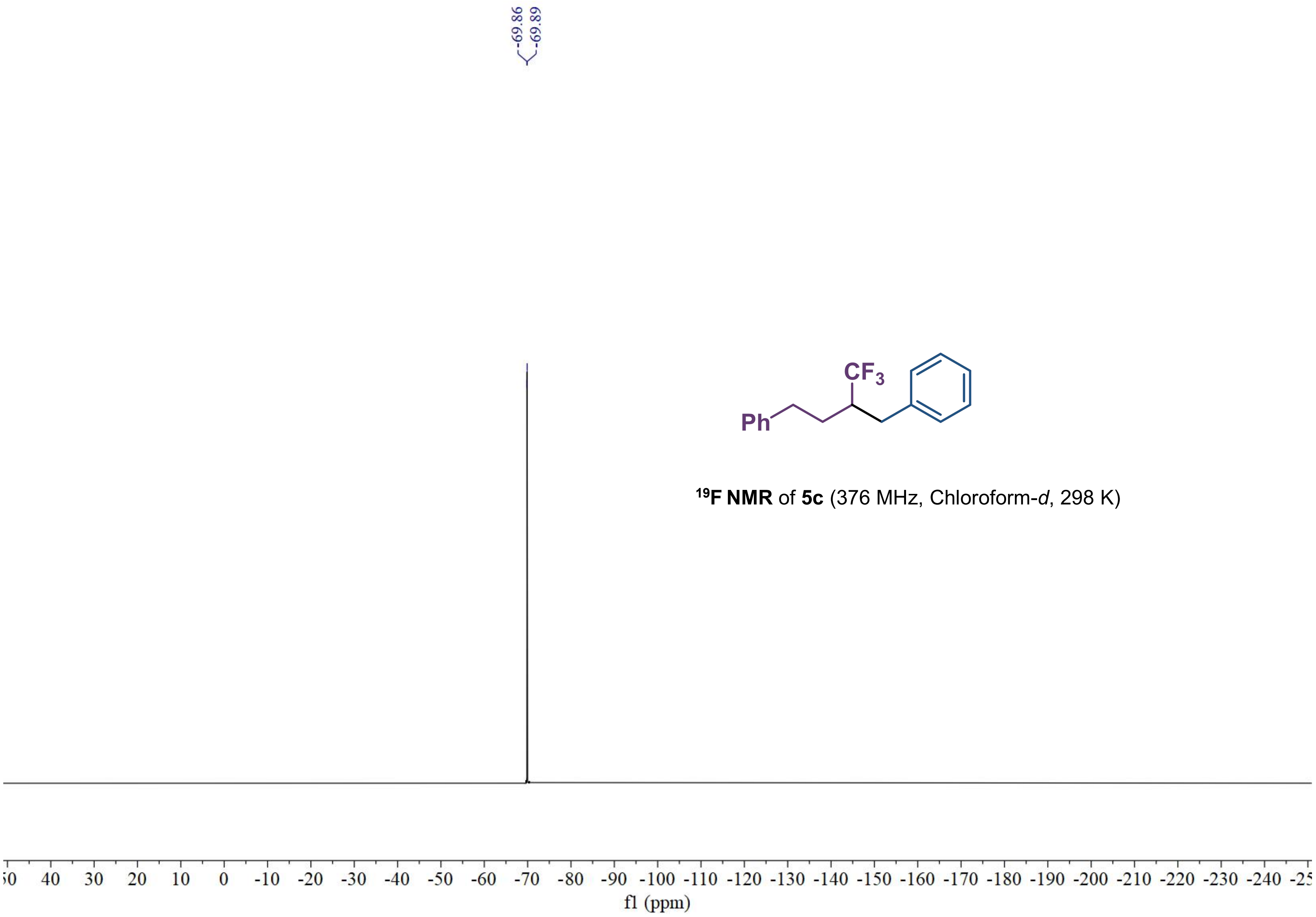

^{19}**F NMR** of **5c** (376 MHz, Chloroform-*d*, 298 K)

REFERENCRS

1. L. Candish, M. Teders, F. Glorius, Transition-Metal-Free, Visible-Light-Enabled Decarboxylative Borylation of Aryl N-Hydroxyphthalimide Esters. *J. Am. Chem. Soc.* **2017**, *139*, 7440-7443.
2. L. Huang, A. M. Olivares, D. J. Weix, Reductive Decarboxylative Alkynylation of N-Hydroxyphthalimide Esters with Bromoalkynes. *Angew. Chem. Int. Ed.*, **2017**, *56*, 11901-11905.
3. Y. Liang, G. C. Fu, Stereoconvergent Negishi Arylations of Racemic Secondary Alkyl Electrophiles: Differentiating between a CF_3 and an Alkyl Group. *J. Am. Chem. Soc.* **2015**, *137*, 9523-9526.
4. P. Xu, W. Fan, P. Chen, G. Liu, Enantioselective Radical Trifluoromethylation of Benzylic C-H Bonds via Cooperative Photoredox and Copper Catalysis. *J. Am. Chem. Soc.* **2022**, *144*, 13468-13474.
5. J. Qi, J. Xu, H. T. Ang, B. Wang, N. K. Gupta, S. R. Dubbaka, P. O'Neill, X. Mao, Y. Lum, J. Wu, Electrophotochemical Synthesis Facilitated Trifluoromethylation of Arenes Using Trifluoroacetic Acid. *J. Am. Chem. Soc.* **2023**, *145*, 24965-24971.
6. K. J. Bian, Y. C. Lu, D. Nemoto Jr, S. C. Kao, X. Chen, J. G. West, Photocatalytic hydrofluoroalkylation of alkenes with carboxylic acids. *Nat. Chem.* **2023**, *15*, 1683-1692.
7. S. F. García, V. O. Chantzakou, F. J. Hernández. Direct Decarboxylation of Trifluoroacetates Enabled by Iron Photocatalysis. *Angew. Chem. Int. Ed.* **2024**, *63*, e202311984.
8. Y. Min, J. Sheng, J. L. Yu, S. X. Ni, G. Ma, H. Gong, X. S. Wang, Diverse Synthesis of Chiral Trifluoromethylated Alkanes via Nickel-Catalyzed Asymmetric Reductive Cross-Coupling Fluoroalkylation. *Angew. Chem., Int. Ed.* **2021**, *60*, 9947-9952.
9. Y. Liang, G. C. Fu, Stereoconvergent Negishi Arylations of Racemic Secondary Alkyl Electrophiles: Differentiating between a CF_3 and an Alkyl Group. *J. Am. Chem. Soc.* **2015**, *137*, 9523-9526.
